# Kappa-alpha plot derived structural alphabet and BLOSUM-like substitution matrix for rapid search of protein structure database

**DOI:** 10.1186/gb-2007-8-3-r31

**Published:** 2007-03-03

**Authors:** Chi-Hua Tung, Jhang-Wei Huang, Jinn-Moon Yang

**Affiliations:** 1 Institute of Bioinformatics, National Chiao Tung University, 75 Po-Ai Street, Hsinchu, 30050, Taiwan; 2 Department of Biological Science and Technology, National Chiao Tung University, 75 Po-Ai Street, Hsinchu, 30050, Taiwan; 3 Core Facility for Structural Bioinformatics, National Chiao Tung University, 75 Po-Ai Street, Hsinchu, Taiwan

## Abstract

We present a novel protein structure database search tool, 3D-BLAST, that is useful for analyzing novel structures and can return a ranked list of alignments. This tool has the features of BLAST (for example, robust statistical basis, and effective and reliable search capabilities) and employs a kappa-alpha (κ, α) plot derived structural alphabet and a new substitution matrix. 3D-BLAST searches more than 12,000 protein structures in 1.2 s and yields good results in zones with low sequence similarity.

## Abstract 2

3D BLAST, a novel protein structure database search tool, is a useful tool for analysing novel structures, capable of returning a list of aligned structures ordered according to E-values.

## Background

A major challenge facing structural biology research in the postgenomics era is to discover the biologic functions of genes identified by large-scale sequencing efforts. As protein structures increasingly become available and structural genomics research provides structural models in genome-wide strategies [1], proteins with unassigned functions are accumulating, and the number of protein structures in the Protein Data Bank (PDB) is rapidly rising [2]. The current structure-function gap highlights the need for powerful bioinformatics methods with which to elucidate the structural homology or family of a query protein by known protein sequences and structures.

Numerous sequence alignment methods (for instance BLAST, SSEARCH [3], SAM [4], and PSI-BLAST [5]) and structure alignment methods (for instance, DALI [6], CE [7], and MAMMOTH [8]) have been demonstrated to identify homologs of newly determined structures. Sequence alignment methods are rapid but frequently unreliable in detecting the remote homologous relationships that can be suggested by structural alignment tools; also, although the latter may be useful, they are slow at scanning homologous structures in large structure databases such as PDB [2]. Various tools including ProtDex2 [9], YAKUSA [10], TOPSCAN [11], and SA-Search [12] have recently been developed to search protein structures quickly. TOPSCAN, SA-Search, and YAKUSA describe protein structures as one-dimensional sequences and then use specific sequence alignment methods to replace BLAST for aligning two structures, because BLAST needs a specific substitution matrix for a new alphabet. Many of these methods have been evaluated based on the performance of two structure alignments but not on the performance of the database search. Additionally, none of these methods provides a function analogous to the *E *value of BLAST (which is probably the most adopted database search tool by biologists) for investigating the statistical significance of an alignment 'hit'.

The three-state secondary elements, namely α-helix, β-sheet, and coils, are rather crude for predicting protein structure, and it is not possible to make use of these elements in three-dimensional (3D) reconstruction without additional information. Many approaches have been proposed to replace three-state secondary structure descriptions with various local structural fragments, also known as a 'structural alphabet' [13-19], which can redefine not only regular periodic structures but also their capping areas. Such studies have described local protein structures according to various geometric descriptors (for example, C_α _coordinates, C_α _distances, α or φ, and ψ dihedral angles) and algorithms (for example, hierarchical clustering, empirical functions, and hidden Markov models [HMMs] [12]). Many of these methods involve protein structure prediction; an exception is the SA-Search tool [12], which is based on C_σ _coordinates and C_α _distances, and which adopts a structural alphabet and a suffix tree approach for rapid protein structure searching.

To address the above issues, we have developed a novel kappa-alpha (κ, α) plot derived structural alphabet and a novel BLOSUM-like substitution matrix, called SASM (structural alphabet substitution matrix), for BLAST [5], which searches in a structural alphabet database (SADB). This structural alphabet is valuable for reconstructing protein structures from just a small number of structural fragments and for developing a fast structure database search method called 3D-BLAST. This tool is as fast as BLAST and provides the statistical significance (*E *value) of an alignment, indicating the reliability of a hit protein structure. For the purposes of scanning a large protein structure database, 3D-BLAST is fast and accurate and is useful for the initial scan for similar protein structures, which can be refined by detailed structure comparison methods (for example, CE and MAMMOTH).

To the best of our knowledge, 3D-BLAST is the first tool that permits rapid protein structure database searching (and provides an *E *value) by using BLAST, which searches a SADB database with a SAMS matrix. The SADB database and the SASM matrix improve the ability of BLAST to search for structural homology of a query sequence to a known protein structure or a family of proteins. This tool searches for the structural alphabet high-scoring segment pairs (SAHSPs) that exist between a query structure and each structure in the database. Experimental results reveal that the search accuracy of 3D-BLAST is significantly better than that of PSI-BLAST [5] at 25% sequence identity or less.

## Results and discussion

### (κ, α) Plot and structural alphabet

**Figure 1:**
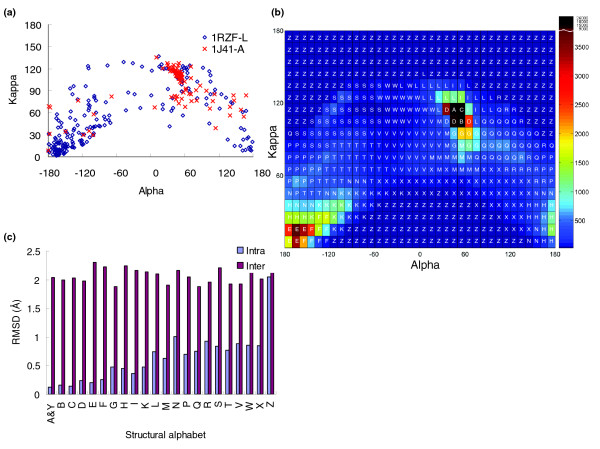
The (κ, α) plot and the distribution of the 23-state structural alphabet. **(a) **The typical (κ, α) plots of an all-α protein (Protein Data Bank [PDB] code 1J41-A; red) and an all-β protein (PDB code 1RZF-L; blue). **(b) **The distribution of accumulated (κ, α) plot of 225,523 segments derived from the pair database with 1,348 proteins. This plot, which comprises 648 cells (36 × 18), is clustered into 23 groups, and each cell is assigned a structure letter. **(c) **The average intrasegment (blue) and intersegment root mean square deviation (rmsd) values of the 23-state structural alphabet.

A pair database comprising 674 structural pairs (Additional data file 1), each with a high structural similarity and low sequence identity, was derived from the SCOP classification database [20] for the (κ, α) plot (Figure 1a,b). Each structure in this database (1,348 proteins) was divided into a series of 3D protein fragments (225,523 fragments), each five residues long, using κ and α angles. The angle κ, ranging from 0° to 180°, of residue i is a bond angle formed by three C_α _atoms of residues i - 2, i, and i + 2. The angle α, ranging from -180° to 180°, of a residue i is a dihedral angle formed by the four C_α _atoms of residues i - 1, i, i + 1, and i + 2. Each structure has a specific (κ, α) plot (Figure 1a) when governed by these two angles. For instance, a typical (κ, α) plot (blue diamond) of an all-β protein (human anti-HIV-1 GP120-reactive antibody E51, PDB code 1RZF-L [21]) is significantly different from that (red cross) of an all-α protein (human hemoglobin, PDB code 1J41-A [22]). Conversely, two similar protein structures have similar (κ, α) plots.

An accumulated (κ, α) plot (Figure 1b) consisting of 225,523 protein fragments was obtained from this pair database. The plot is split into 648 cells (36 × 18) when the angles of κ and α are divided by 10°. In the accumulated (κ, α) plot, most of the α-helix segments are located on four cells in which the α angle ranges from 40° to 60°, and the κ angle ranges from 100° to 120°. In contrast, the κ angle of most of the β-strand segments ranges from 0° to 30°, and the α angle ranges from -180° to -120°, or 160° to 180°. The number of 3D segments in each cell ranges from 0 to 22,310, and the color bar on the right side presents the distribution scale. Based on the definitions in the DSSP program [23] the numbers of α-helix and β-strand segments are 82,482 (36.6%) and 52,371 (23.3%), respectively. Most 3D segments in the same cell in this plot have similar 3D shapes, that is, a root mean square deviation (rmsd) below 0.3 Å on five contiguous C_α _atom coordinates. Moreover, the conformations of 3D segments located in adjacent cells are often encoded into similar structural letters which have more similar 3D structures than those in distant cells (Figures 1b,c). Hence, the (κ, α) plot is helpful for clustering these 3D segments to determine a representative segment for each cluster.

**Figure 2:**
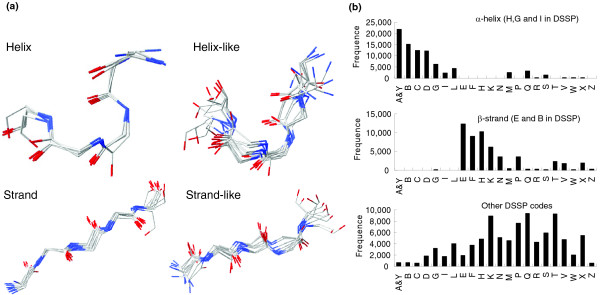
The relationship between the 23-state structural alphabet and three-state secondary elements. **(a) **The three-dimensional (3D) segment conformations of the five main classes of the 23-state structural alphabet, including helix letter (A, Y, B, C, and D), helix-like letters (G, I, and L), strand letters (E, F, and H), strand-like letters (K and N), and others (Additional data file 2). The shapes of the segments in the same category are similar to each other. **(b) **The distributions of the 23-state structural alphabet on 82,482 α-helix segments, 52,371 β-strand segments, and the 66,503 coil segments defined by the DSSP program.

Based on the (κ, α) plot and a new nearest neighbor clustering (see Materials and methods, below), a new 23-state structural alphabet was derived to represent the profiles of most 3D fragments, and was roughly categorized into five groups (Figure 2a and Additional data file 2): helix letters (A, Y, B, C, and D), helix-like letters (G, I, and L), strand letters (E, F, and H), strand-like letters (K and N), and others. The 3D shapes of representative segments in the same category are similar; conversely, the shapes of different categories are significantly different. For instance, the shapes of representative 3D segments in the helix letters are similar to each other, as are those in strand alphabets. In contrast, the shapes of helix letters and strand letters obviously differ. The average structural distance (determined from the rmsd value of five continuous C_α _atom positions between a pair of 5-mer segments) of intersegments in both helix and strand letters is less than 0.4 Å (Figure 1c), and is much less that those of other letters in the structural alphabet. Additionally, most α-helix secondary structures based on the definition of the DSSP program are encoded as helix or helix-like alphabets, and none are encoded as strand or strand-like alphabets (Figure 2b). Conversely, most β-strand segments are encoded as strand or strand-like letters (Additional data file 3).

All residues were fairly restricted in their possibilities in the (κ, α) plot (Figure 1b). The proportion of cells with 0 segments, which were encoded as structural letter 'Z', was 28.2% (183 cells among 648). Additionally, the numbers of cells and segments with structural letter 'Z' were 272 (42.0% [272/648]) and 989 (0.4% [989/225,523]), respectively. Restated, only 0.44% segments were widely distributed in 41.98% of cells. If the segments of a new protein structure are located on these 41.98% cells, then they may be regarded as poor structural segments. Conversely, five helix letters (A, Y, B, C, and D) and three strand letters (E, F, and H) were located in seven and 30 cells (Figure 1b), respectively. The total number of segments located in these 37 (4.4%) cells was 75,477 (33.5%).

The (κ, α) plot is similar to a Ramachandran plot, based on the following observations. First, the α-helices are located in very restricted areas, in which α ranges from 40° to 60°, and κ ranges from 100° to 120°. Additionally, β-sheet segments are restricted to some regions in the (κ, α) plot. All residues are fairly restricted in their possibilities in both plots. Second, angles φ and ψ in the Ramachandran plot, denoting a protein structure with a series of 3D positions of amino acids, are widely adopted to develop various structural segments (blocks). Here, the (κ, α) plot was utilized to develop a structural alphabet, which represents a protein structure as a series of 3D protein fragments, each of which are five residues long. The angles φ and ψ represent the position relationship of two contiguous amino acids, whereas the angles κ and α represent the position relationship of five amino acids. These observations indicate that the (κ, α) plot is an effective means of both developing short sequence structure motifs and assessing the quality of a protein structure.

### Reconstructing protein

**Figure 3:**
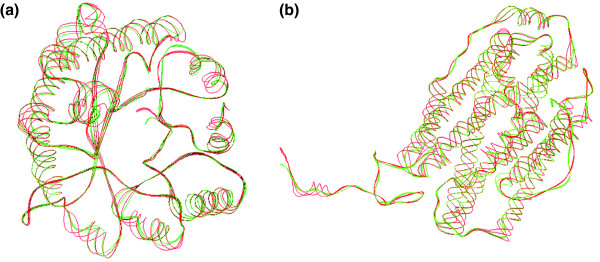
Reconstruction protein structures using the 23-state structural alphabet. Reconstruction of the **(a) **α-β-barrel protein (Protein Data Bank [PDB] code 1TIM-A [24]) and **(b) **ribonucleotide reductase (PDB code 1SYY-A [25]). The α-carbon root mean square deviation (rmsd) between the X-ray structures (red) and reconstructed proteins (green) are 0.80 Å (1TIM-A) and 0.63 Å (1SYY-A), respectively.

A greedy algorithm and the evaluation criteria (global-fit score) presented by Kolodny and coworkers [15] were applied to measure the performance of 23-state structural alphabet (structural segments) in reconstructing the α-β-barrel protein (PDB code 1TIM-A [15,24]) and 38 structures (Additional data file 4) selected from the SCOP-516 set, which comprises 516 proteins. This greedy algorithm reconstructs the protein in increasingly large segments using the best structural fragment, namely the one whose concatenation produces a structure with the minimum rmsd from the corresponding segment in the protein from 23 structural segments. No energy minimization procedure was utilized to optimize the reconstructing structures in this study. The global rmsd values were from 0.58 Å to 2.45 Å, and the average rmsd value was 1.15 Å for these 38 proteins. Figure 3a,b illustrate the reconstructed structures of the α-β-barrel protein and ribonucleotide reductase (PDB code 1SYY-A [25]), respectively. The C_α _carbon rmsd values were 0.80 Å (1TIM-A) and 0.63 Å (1SYY-A) between the X-ray structures (red) and reconstructed proteins (green). The reconstructed structures are frequently close to the X-ray structures on both α-helix and β-sheet segments, and the loop segments account for the main differences. If all representative segments (465 segments) of the non-zero cells in the (κ, α) plot were considered when reconstructing structures, then the global rmsd values would be in the range 0.35 to 2.32 Å, and the average rmsd value would be 0.94 Å.

The 23-state structural alphabet should be able to represent more biologic meaning than standard three-state secondary structural alphabets. First, the classic regular zones of three-state secondary structures are flexible structures. For instance, α-helices may be curved [26] and more than one-quarter of them are irregular [27], and the φ and ψ dihedral angles of β-sheets are widely dispersed. The proposed 23-state alphabet describes α-helices with eight segments (five helix letters and three helix-like letters) and β-sheets with five segments (Figure 2a). Figure 3 reveals that the 23 structural segments performed well in reconstructing protein structures, particularly in the structure segments of classic α-helices and β-sheets. Second, the three-state secondary structure cannot represent the large conformational variability of coils. Nonetheless, some similar structures can be identified for many of the protein fragments, such as β-turns [28], π-turns, and β-bulges [29]. Here, 10 structural segments in the 23-state alphabet were utilized to describe the loop conformations. An analysis using the PROMOTIF [30] tool reveals that most of the segments (>80%) in the letter 'W' are β-turns.

### Protein structure database search

In a structural database search, 3D-BLAST identifies the known homologous structures and determines the evolutionary classification of a query structure from an SADB database (Additional data file 5). Users input a PDB code with a protein chain (for example, 1GR3-A) or a domain structure with a SCOP identifier (for example, d1gr3a_). When the query has a new protein structure, the 3D-BLAST tool enables users to input the structure file in the PDB format. The tool returns a list of protein structures that are similar to the query, ordered by *E *values, within several seconds. When we searched databases such as SCOP [20] or CATH [31], which are based on structural classification schemes, the evolutionary classification (family/superfamily) of the query protein was based on the first structure in the 3D-BLAST hit list.

The main advantages of 3D-BLAST using BLAST as a search tool include robust statistical basis, effective and reliable database search capabilities, and established reputation in biology. However, the use of BLAST in protein structure search has several limitations, namely the need for an SADB database, a new SASM matrix, and a new *E *value threshold to show the statistical significance of an alignment hit. These issues are described in the following subsections.

#### SADB databases and test data sets

A SADB database was easily derived from a known protein structure database based on the (κ, α) plot and the structural alphabet. We created five SADB databases derived from the following protein structure databases PDB; a nonredundant PDB chain set (nrPDB); all domains of SCOP1.69 [20]; SCOP1.69 with under 40% identity to each other; and SCOP1.69 with under 95% identity to each other.

The SCOP-516 query protein set, which has a sequence identity below 95% selected from the SCOP database [20], was chosen to measure the utility of 3D-BLAST for the discovery of homologous proteins of a query structure. This set contains 516 query proteins that are in SCOP 1.69 but not in SCOP 1.67, and the search database was SCOP 1.67 (11,001 structures). The total number of alignments was 5,676,516 (516 × 11,001). For evolutionary classification, the first position of the hit list of a query was treated as the evolutionary family/superfamily of this query protein. For comparison with related work on rapid database searching, 3D-BLAST was also tested on a dataset of 108 query domains, termed SCOP-108 (Additional data file 6), proposed by Aung and Tan [9]. These queries, which have under 40% sequence homology to each other, were chosen from medium-sized families in SCOP. The search database (34,055 structures) represents most domains in SCOP 1.65. Finally, the utility of 3D-BLAST for 319 structural genomics targets was analyzed; the search database was SCOP 1.69, with under 95% identity to each other.

Here, several common metrics (precision, recall, and receiver operating characteristic [ROC] curve) were utilized to assess the predicted quality of a search method on database searching. Precision is defined as A_h_/T_h _and recall is defined as A_h_/A, where A_h _is the number of true hit domains in the hit list, T_h _is the total number of domain proteins in the hit list, and A is total number of true hits in the database. The ROC curve plots the sensitivity (recall) against 1.0 - specificity (false-positive rate). The average precision is defined as $(\sum_{i=1}^{A}i/T_{h}^{i})/A$, where $T_{h}^{i}$ is the number of compounds in a hit list containing i correct domains.

#### Structural alphabet substitution matrix (SASM)

**Figure 4:**
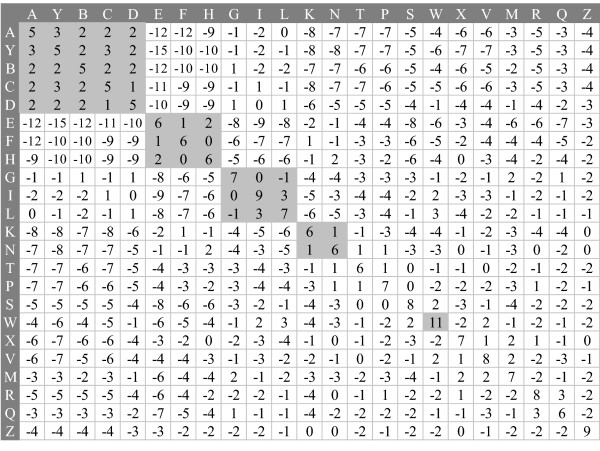
Structural alphabet substitution matrix (SASM). The SASM is a BLOSUM-like substitution matrix for determining aligned scores for the 3D-BLAST tool, as BLOSUM matrix used in BLAST. The scores are high if the same letters (those on the main diagonal) or letters in the same category (those near the main diagonal) are aligned. In contrast, the scores are low when two letters with different properties are aligned.

A substitution matrix is an essential component when BLAST is used to search a structural database quickly. A new BLOSUM-like substitution matrix, called SASM (Figure 4), was developed by using a method similar to that used to construct BLOSUM62 [32] based on the pair database. The SASM (23 × 23) provides insight into substitution preferences for 3D segments between homologous structures with low sequence identity.

The SASM matrix presents good relationships between biologic functions and protein structures. The highest substitution score in SASM represents the alignment of an alphabet 'W' with an alphabet 'W', in which the conformations of segments are similar β-turns. Substitution scores are high when two identical structural alphabets (for example, diagonal entries) are aligned. For instance, the alignment scores of aligning 'I' and 'S' to themselves are 9 and 8, respectively. Most substitution scores are positive when two structural alphabets in the same category, for example helix alphabets (A, Y, B, C, and D), are aligned. Conversely, the lowest substitution score (-15) in SASM is for the alignment of 'Y' (helix alphabet) with 'E' (strand alphabet). These scores are also low when helix alphabets (A, Y, B, C, and D) are aligned with strand alphabets (E, F, and H).

The SASM matrix and BLOSUM62 [32] were compared because they adopted BLAST as the search tool. The highest substitution score is 11 for both matrices. By contrast, the lowest score for SASM (-15) is much lower than that for BLOSUM62 (-4). This large difference occurs mainly because α-helices and β-strands constitute very different protein secondary structures, and the structural letters pertaining to these two structures are better conserved than those of amino acid sequences. Because the gap penalty is an important factor, various combinations of gap penalties were systematically tested for 3D-BLAST and the SASM matrix based on the pair database (1,348 proteins). Here, the optimal values for the open gap penalty and the extended one are 8 and 2, respectively.

#### Statistics of 3D-BLAST

A database search method should enable users to examine the statistical significance of an alignment in order to determine the reliability of the prediction. 3D-BLAST maintains the benefits of the BLAST tool in terms of ordering hit proteins by *E *value for rapid scanning of structural database. We used the theoretical result [33,34] to estimate the *E *value of an ungapped local alignment of two structural alphabet (SA) sequences A (query) and B (database sequence) with score S using the following steps. First, we computed statistical parameters λ and K according to the 23-state SA compositions of A and B and the SASM matrix (Figure 4). In a SA database search, we used the actual SA composition of A and an average SA composition for B. Second, we computed adjusted lengths L_A _and L_B _of A and B, where L_B _is the sum of lengths of all database sequences. Third, we obtained a normalized score S' = λS - ln(K) and calculated the *E *value = L_A_L_B_e^-S^. Although the theory referred to above has not been proved to be valid for gapped local alignments, computational experiments suggest that that it is [35,36]. The statistical parameters λ and K cannot be derived from theory; they must be estimated by simulation with random or real but unrelated sequences.

To evaluate the accuracy of the *E *values reported by 3D-BLAST, we submitted shuffled SA sequences as queries and found the number of match sequences with *E *values below various thresholds. For simplicity, we used the query set SCOP-516 and the respective shuffled queries (516 SA sequences) that represent protein structures, and the search database was SCOP 1.67. Shuffled queries mimic completely random SA sequences, which preserve only the composition basis of a protein structure, using the typical SA composition. The numbers of matches of 516 shuffled queries with *E*-values below e^-20^, e^-15^, and e^-10 ^are 0, 3, and 326, respectively. On the other hand, the numbers of matches of 516 queries in the SCOP-516 dataset with *E *values below e^-20^, e^-15 ^and e^-10 ^are 8,268, 18,700, and 64,440, respectively. Protein structures and the structural letters are more conserved than protein sequences; thus, as one would expect, the *E *values of 3D-BLAST are larger than those of BLAST when the reliable indicators are similar.

**Figure 5:**
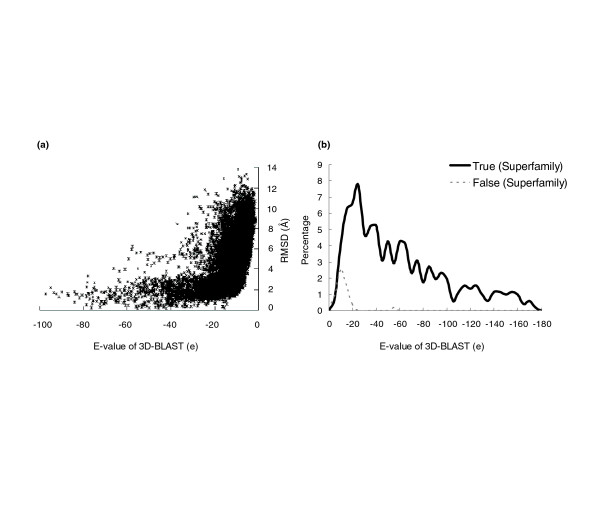
3D-BLAST performance with *E *values on the protein query set SCOP-516. **(a) **The relationship between 3D-BLAST *E *values and the root mean square deviation (rmsd) values of aligned residues. The average rmsd values with *E *value below e^-10^, e^-15^, e^-20^, and e^-25 ^are 3.57 Å, 2.85 Å, 2.37 Å and 2.25 Å, respectively, based on 22,415 protein structures randomly selected from the 516 returned lists. **(b) **The relationship between *E *values and the percentages of true (black) and false (gray) function assignment. The correct percentages of the superfamily assignments with *E *values below e^-10^, e^-15^, e^-20 ^and e^-25^, are 95.26%, 97.67%, 99.31%, and 99.75%, respectively. The coverage values of the function assignment are 98.06% (<e^-10^), 91.47% (<e^-15^), 83.72% (<e^-20^), and 76.74 (<e^-25^).

The proposed 3D-BLAST provides a threshold *E *value to identify a highly significant similarity with the query. The SASM matrix reveals that the biologic significance of the high-scoring structures can be inferred from the similarity score, and the proportion of true positives rises when a lower *E *value is utilized (Figure 5). Figure 5a shows that *E *values correlate strongly with the rmsd values of aligned residues between the query protein and the hit proteins. A total of 22,415 proteins were randomly chosen from the hit lists of 516 query proteins in the SCOP-516 dataset. Among these 22,415 proteins, 27.72% (6,215 structures) had rmsd values below 3.0 Å. If the *E *value was restricted to under e^-20^, then 83.52% of hit proteins (2,130 proteins from among 2,549 proteins) had rmsd values less than 3.0 Å, and the average rmsd was 2.37 Å. When the *E *value was restricted to under e^-15 ^and under e^-10^, then 72.65% (3,984 proteins among 5,487 proteins) and 51.70% (5,742 proteins among 11,106 proteins) of proteins had rmsd values less than 3.0 Å, respectively, and the average rmsd values were 2.85 Å and 3.57 Å.

For classification assignment, the relationship between the *E *value of the first hit and the number of correct (dark line) and false (gray line) classification assignments for the SCOP-516 dataset was calculated (Figure 5b). If the *E *value was restricted to under e^-15^, then 97.67% of 516 query structures are assigned correct classifications and the coverage was 91.47%. The coverage is defined as P/T, where P is the number of assigned structures by a method and T is total number of structures. For example, P is 472 and T is 516 for the set SCOP-516. When the *E *value was less than e^-20 ^and e^-10^, 99.31% and 95.26% of the predicted cases were correct, and the coverage values were 83.72% and 98.06%, respectively. When the sequence identity was less than 25% (154 proteins from among 516 proteins), the rate of correct assignment was 90.35%. The coverage was 72.12% when the *E *value was less than e^-15^. For the database search, the precision was 0.80 and the recall was 0.48 when the *E *value was below e^-15^; by comparison, the precision was 0.90 and the recall was 0.42 when the *E *value was below e^-20^. These analytical results demonstrate that the *E *value of 3D-BLAST enables users to examine the reliability of the structure database search of a query.

### Search examples

**Figure 6:**
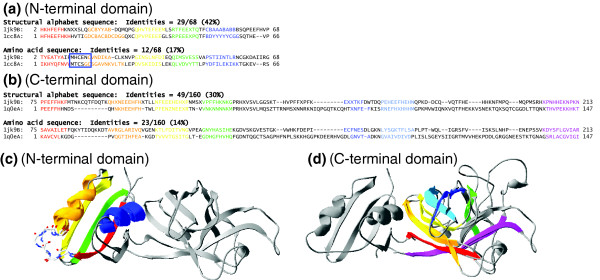
Sequence and structure alignments of 3D-BLAST search results using yCCS as the query. This protein consists of the amino-terminal and carboxyl-terminal domains. Sequence alignments (structural alphabet and amino acid sequences) of **(a) **amino-terminal domain and **(b) **carboxyl-terminal domain between the query protein and homologous proteins (Protein Data Bank [PDB] codes 1CC8-A and 1QOE-A, respectively). Structure alignments of **(c) **amino-terminal domain and **(d) **carboxyl-terminal domain between the query protein and the homologous proteins (PDB codes 1CC8-A and 1QOE-A, respectively). The aligned secondary structures are denoted as a continuous color spectrum from red through orange, yellow, green, and blue to violet. The amino-terminal domain contains an MT/HCXXC metal binding motif (blue box in panel a and wireframe model in panel c). yCCS, yeast copper chaperone for superoxide dismutase.

Using the yeast copper chaperone for superoxide dismutase (yCCS) from *Arabidopsis thaliana *(PDB code 1JK9-B) [37] as the query protein and an *E *value threshold of 10^-10^, a 3D-BLAST search of the database SCOP1.69 found 19 members (Table 1). Figure 6 shows two hits of the search results. The protein (yCCS) comprised amino-terminal and carboxyl-terminal domains. The amino-terminal domain, called HMA (heavy-metal associated) domain in the SCOP database, plays a role in copper delivery. This domain contains an MH/TCXXC metal binding motif (blue box in Figure 6a), and is very similar to the metallochaperone protein Atx1. The carboxyl-terminal domain, termed the Cu,Zn superoxide dismutase-like domain in the SCOP database, comprised an eight-stranded β-barrel that strongly resembles yeast superoxide dismutase I and human superoxide dismutase I.

**Table 1:** 3D-BLAST search results by copper chaperone for superoxide dismutase (PDB code 1JK9-B) from yeast as query

PDB code	Protein title	log(*E *value)	rmsd (Å)	Sequence identity (%)^a^	SCOP sccs	Species
1EJ8-A	Copper chaperone for yeast sod	-50.70	1.10	57.6	b.1.8.1	*Saccharomyces cerevisiae*
1QUP-A	Copper chaperone for superoxide dismutase	-27.05	0.58	28.3	d.58.17.1	*Saccharomyces cerevisiae*
1CC8-A	Superoxide dismutase 1 copper chaperone	-17.40	1.64	8.6	d.58.17.1	*Saccharomyces cerevisiae*
1TO4-A	Superoxide dismutase	-17.22	2.78	19.6	b.1.8.1	*Schistosoma mansoni*
1DO5-A	Human copper chaperone for superoxide dismutase domain II	-16.30	2.57	17.3	b.1.8.1	*Homo sapiens*
1OSD-A	Oxidized Merp from Ralstonia metallidurans CH34	-16.05	1.61	11.1	d.58.17.1	*Ralstonia metallidurans*
1Q0E-A	Copper, zinc superoxide dismutase	-14.22	1.68	17.7	b.1.8.1	*Bos taurus*
1OAL-A	Superoxide dismutase	-14.00	2.19	17.7	b.1.8.1	*Photobacterium leiognathi*
1SRD-A	Copper, zinc superoxide dismutase	-13.30	2.71	17.5	b.1.8.1	Synthetic construct
1FE0-A	Copper transport protein atox1	-13.10	1.40	9.9	d.58.17.1	*Homo sapiens*
1OZU-A	Copper, zinc superoxide dismutase	-12.70	2.42	18.5	b.1.8.1	*Homo sapiens*
1ESO	Copper, zinc superoxide dismutase	-12.30	2.49	17.6	b.1.8.1	*Escherichia coli*
1FVQ-A	Copper-transporting ATPase	-12.00	1.64	9.9	d.58.17.1	*Saccharomyces cerevisiae*
1JCV	Copper, zinc superoxide dismutase	-11.70	2.24	20.3	b.1.8.1	*Saccharomyces cerevisiae*
1S6U-A	Copper-transporting ATPase 1	-11.15	1.87	8.6	d.58.17.1	*Homo sapiens*
1XSO-A	Copper, zinc superoxide dismutase	-10.70	1.88	19.3	b.1.8.1	*Xenopus laevis*
1OQ3-A	Potential copper-transporting ATPase	-10.40	1.84	11.4	d.58.17.1	*Bacillus subtilis*
1VCA-A	Human vascular cell adhesion molecule-1	-10.30	3.76	15.9	b.1.1.3	*Homosapiens*
1KQK-A	Potential copper-transporting ATPase	-10.22	1.63	12.3	d.58.17.1	*Bacillus subtilis*
1MWY-A	The N-terminal domain of ZntA in the apo-form	-10.10	1.67	9.0	d.58.17.1	*Escherichia coli*

^a^Amino acid sequence identity is calculated using FASTA software. PDB, Protein Data Bank; rmsd, root mean square deviation.

3D-BLAST was able to identify 9 and 10 homologous structures of amino-terminal domains and carboxyl-terminal domains, respectively, using this two-domain protein (yCCS) as query. The sequence identities between yCCS and most of the homologous structures (17 out of 19 proteins) were less than 20%. Figure 6a,c illustrates sequence alignments and the structure alignment between yCCS and an amino-terminal domain homologous protein (PDB code 1CC8-A [38]). The sequence identities of structure alphabet and amino acid sequences were 42% and 17%, respectively. 3D-BLAST can align six amino acids of the metal binding motif together, and the rmsd is 1.64 Å between these two proteins. The aligned secondary structures are represented as a continuous color spectrum from red through orange, yellow, green and blue to violet. Figures 6b,d show the sequence and structure alignments between yCCS and a carboxyl-terminal domain homologous protein (PDB code 1QOE-A [39]). The sequence identities of the structure alphabet and the amino acid sequences were 30% and 14%, respectively, and the rmsd between these two proteins was is 1.68 Å. The structural alphabets were strongly conserved in areas of the secondary structures (green block), which are β-strands represented by structural alphabets, such as E, F, H, K, and N. These results reveal that the structural alphabet sequences are much better conserved than the amino acid sequences, which explains why 3D-BLAST could detect the invariant residues and find these distantly related proteins.

### Search results and comparison with PSI-BLAST

**Figure 7:**
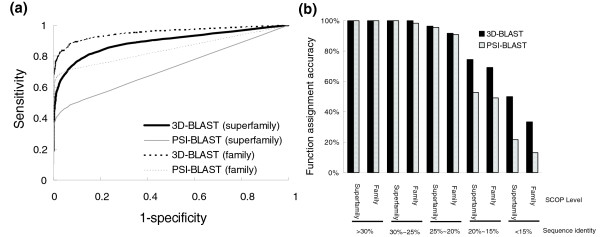
Comparison 3D-BLAST with PSI-BLAST. Evaluation of the 3D-BLAST and PSI-BLAST in database search and family/superfamily assignments by SCOP-516 based on **(a) **receiver operating characteristic (ROC) curves and **(b) **percentages of correct classification assignments. 3D-BLAST (black lines) outperforms PSI-BLAST (gray lines) in the ROC curve. The dashed and solid lines denote the ROC curves for the SCOP superfamily and SCOP family assignments, respectively. 3D-BLAST (black bars) is much better than PSI-BLAST (gray bars) when the sequence identity is under 20%.

Figure 7 illustrates the accuracies of the 3D-BLAST and PSI-BLAST in structure database searches and evolutionary classification assignments using the query set SCOP-516. For this experiment, 3D-BLAST was compared with PSI-BLAST, because PSI-BLAST often performs much better than BLAST for this purpose. Standalone PSI-BLAST [5] was installed on a personal computer with a single processor (Pentium 2.8 GHz with 512 megabytes of RAM). The main differences between 3D-BLAST and PSI-BLAST are in the search databases and substitution matrices. In 3D-BLAST, the substitution matrix is the SASM matrix and the searching database is the SADB, whereas PSI-BLAST adopts an amino acid sequence database and a BLOSUM62 substitution matrix. The number of iterations for PSI-BLAST was set to three and the open gap penalty and the extended one are 11 and 1, respectively. For database search, the threshold of the *E *values of 3D-BLAST and PSI-BLAST are set to 10^-10 ^and 0.01, respectively.

For a database search tool, the ROC curve (Figure 7a) provides an estimation of the likely number of true positive and false positive predictions. A perfect method, which can recover all true hits without any false positives, can be denoted as a point in the top left corner of this graph, whereas a random method that generates equal numbers of true positive and false positive predictions uniformly distributed across all scores would yield a diagonal line from (0,0) to (1,1). Figure 7a shows that 3D-BLAST (dark lines) yields much better predictions than does PSI-BLAST (gray lines). The sensitivity of family assignments was superior to that of superfamily assignments in both methods, whereas the false-positive rates of family assignments were higher than those of the superfamily assignments.

For most sets of sequence identities, 3D-BLAST outperformed PSI-BLAST (Figure 7b) in protein evolutionary classification assignments. Almost 70.16% (362 out of 516 proteins) of query proteins were more than 25% identical to one of the library representatives from the same SCOP superfamily, and 100% of these domains were correctly mapped by both 3D-BLAST and PSI-BLAST. When the sequence identity was less than 25% (154 out of 516 proteins), the accuracy of 3D-BLAST ranged from 96.29% to 50%, whereas the accuracy of PSI-BLAST ranged from 94.29% to 21.74% (Figure 7b). These proteins were difficult to assign because of the limited similarity of the query proteins to the representative library domains. 3D-BLAST yielded significantly better results than did PSI-BLAST at sequence identity levels of 25% or less. The analytical results reveal that, as expected, sequence comparison tools that are more sensitive to distant homology are usually more successful at making challenging assignments. In summary, 3D-BLAST achieved more reliable assignments than did PSI-BLAST in cases of low sequence identity for this test set. The structural alphabet, SADB database, and SASM matrix could predict protein structures more accurately than simple amino acid sequence analyses.

### Comparisons and discussions

**Figure 8:**
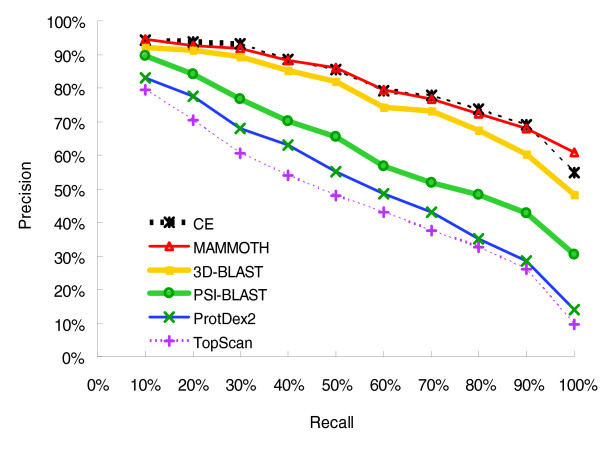
3D-BLAST versus fast structure search, sequence profile search, and detailed structural alignment. Comparison of 3D-BLAST with fast structure search methods (TopScan and ProtDex2), sequence profile search method (PSI-BLAST), and detailed structural alignment methods (CE and MAMMOTH) based on the precision and recall of the protein query set SCOP-108. The performance of TOPSCAN and ProtDex2 is summarized from previous work [9].

Comparing the results of different structure database search methods is generally neither straightforward nor completely fair, because each such method utilizes different accuracy measures, searching databases, and test complexes. Figure 8 shows the relationship between recall and precision, and Table 2 presents the average search time and average precision of 3D-BLAST, PSI-BLAST, MAMMOTH [8], CE [7], TOPSCAN [11], and ProtDex2 [9] on 108 query proteins proposed by Aung and Tan [9] (Additional data file 6). The performance of TOPSCAN and ProtDex2, which are fast search methods for scanning structure databases, was summarized from previous studies [9]. Other four programs were installed and run on the same personal computer with a single processor. Here, the PSI-BLAST and 3D-BLAST used *E *values to order the hit proteins; MAMMOTH and CE (detailed structure alignment tools) utilized Z scores to rank the hit proteins.

**Table 2:** Average search time and mean average precision of each program on 108 queries in SCOP-108

Program	Mean of average precision	Total searching time (s)	Average time per query (s)	Related to 3D-BLAST
PSI-BLAST^a^	69.8%	18.31	0.170	0.533
3D-BLAST	78.2%	34.35	0.318	1
MAMMOTH^b^	82.1%	131,855	1220.88	3838.58
CE	83.4%	~13.5 days	~3 hours	~34000

Time was measured using a personal computer equipped with an Intel Pentium 2.8 GHz processor with 1,024 megabytes of RAM memory. ^a^PSI-BLAST used *E *values to rank the hit proteins. ^b^MAMMOTH and CE utilized Z scores to rank hit proteins.

On average, 3D-BLAST required about 3.18 s seconds to scan the database for each query protein (Table 2). It is about 34,000 and 3838 times faster than CE and MAMMOTH, respectively. 3D-BLAST was about two times slower than PSI-BLAST, because 3D-BLAST identified many more words (typically of length three for proteins in BLAST) that score more than a threshold value in the SADB databases than those identified by PSI-BLAST in protein sequence databases. The reason for this stems from the fact that the BLAST algorithm scans the database for words that score at least a threshold when aligned with some words within the query sequence; the algorithm then extends each such 'hit' in both directions to check the alignment score [5].

MAMMOTH is the best and TOPSCAN is the worst for these 108 queries among these six methods (Figure 8). 3D-BLAST was much better than fast structure database search methods (TOPSCAN [11] and ProtDex2 [9]), and its performance approached those of CE and MAMMOTH. Notably, PSI-BLAST outperformed both TOPSCAN and ProtDex2, which considered secondary and 3D protein structures. As shown in Table 2, the mean of average precision of 3D-BLAST (78.2%) was better than that of PSI-BLAST (69.2%) and lightly worse than those of CE (82.1%) and MAMMOTH (83.4%). For some query proteins, such as serotonin *N*-acetyltranferase [40] (PDB code 1CJW-A) and translation initiation factor IF2/eIF5B [41] (PDB code 1G7S-A; Additional data file 6), 3D-BLAST, MAMMOTH, and CE were markedly better than PSI-BLAST because most sequence identities between the query proteins and their members are under 20%. For several query proteins, such as human dihydro-orotate dehydrogenase [42] (PDB code 1D3G-A) and yeast copper chaperones for SOD [43] (PDB code 1EJ8-A), CE and MAMMOTH were worse than 3D-BLAST. Interestingly, PSI-BLAST outperformed CE, MAMMOTH, and 3D-BLAST for *S*-adenosylhomocysteine hydrolase [44] (PDB code 1B3R-A).

The recognition performance of 3D-BLAST is expressed as top rankings (Additional data file 7), using Lindahl's benchmark [45], together with the performance of eight popular sequence comparison (for example, HMM and profile methods). The benchmark includes 976 proteins derived from the SCOP for identifying homologous pairs at different similarity levels (Additional data file 8). Sequence identities between the query proteins and their homologous members in the superfamily and fold levels are much lower than those at the family level. These methods can be divided into two categories: methods using only single sequence information (BLAST2 and SSEARCH [3]) and methods using multiple sequence alignments (PSI-BLAST, HMMER-HSSP [46], HMMER-PSI-BLAST [46], SAM-HSSP [4], SAM-PSIBLAST [4], and BLAST-LINK [45]). The methods of constructing profiles/HMMs used a larger dataset, comprising the SWISSPROT-35 and TREMBL-5 databases [47] together with the benchmark sequences of the HSSP database [48].

At the family level, 3D-BLAST identified 78.4% of homologous pairs that were ranked in the top 5. This was comparable to the best performance of any of the other methods (78.9%), which was achieved by BLAST-LINK (Additional data file 7). At the superfamily and fold levels, 3D-BLAST significantly outperformed all of the other methods. 3D-BLAST yielded 54.8% and 39.3% homologous pairs at the superfamily and fold levels, respectively. On the other hand, the best accuracies for the other methods were 40.6% (by BLAST-LINK) at the superfamily level and 18.7% (by SAM-PSIBLAST) at the fold level.

The main factors causing 3D-BLAST to perform poorly on some cases in both SCOP-516 and SCOP-108 datasets are summarized as follows. First, 3D-BLAST might have made minor shifts when aligning two local segments with similar codes, because the structural alphabets did not consider the actual euclidean distances. Hence, 3D-BLAST is more sensitive when the query proteins (for example, PDB code 1VDL-A and 1PMZ-A in SCOP-516) are the members of the 'all-α' class in SCOP. Second, the structural similarity of a query protein to the library members is rather limited. Third, an inherent problem in the BLAST algorithm is inability to detect remote homology of structural alphabet sequences. Use of PSI-BLAST as the search algorithm for 3D-BLAST slightly improved the overall performance on the SCOP-516 set. An enhanced position-specific score matrix of the structure alphabet for SADB databases should be developed to improve the performance of 3D-BLAST in the future. Finally, the *E *values of the hits are not significant.

We demonstrated the robustness and adaptability of 3D-BLAST for the initial scan of large protein structure databases; conversely, detailed structure alignment tools often align two structures slowly but accurately. Because of basic differences, comparisons between 3D-BLAST and detailed structure alignment tools are not straightforward. However, detailed structure alignment tools can be applied to refine the searching structures of 3D-BLAST to improve accuracy of prediction.

### Structural genomics targets

**Figure 9:**
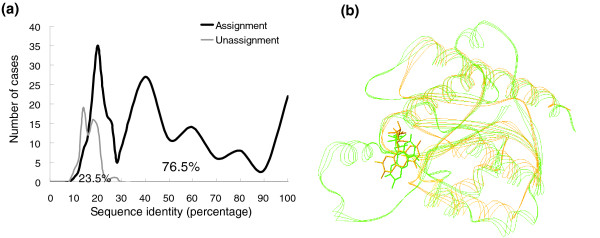
3D-BLAST function assignment results for 319 proteins targeted by structural genomics. **(a) **The percentages of assigned proteins (black) and unassigned proteins (gray) are 76.5% and 23.5%, respectively, when the threshold *E *value is set to e^-15^. **(b) **Structure alignment between the query protein (Protein Data Bank [PDB] code 1yrhA, green) and the first-rank protein (PDB code 1e5dA, orange) in the hit list. The FMN ligand of both proteins are indicted as wireframe models.

We analyzed 319 structural genomics targets, called SG-319, using 3D-BLAST (Figure 9) with regard to function assignment. The structural genomics initiative aims to determine representative structures for all protein families in cells [1,49,50]. To sample the protein structural space more efficiently, structural genomics projects employ various target selection strategies to filter out proteins that are homologous to the proteins with structures already in the PDB [51]. As a result, the molecular functions of the proteins targeted by structural genomics are often unknown. The SG-319 set contains 319 structural genomics targets contributed by more than 10 structural genomics consortia, and publication dates range from 1 January 2005 to 30 September 2005. There are 126 proteins in SG-319 having the 'unknown function' annotation.

3D-BLAST used these 319 proteins as query proteins, and the search classification database was SCOP 1.69, which contains 12,074 domains. About 38.2% (122 proteins) and 32.6% (104 proteins) of the SG-319 proteins have more than 25% and under 20% sequence identity, respectively, to one of the library representatives of the SCOP superfamily, according to search results with 3D-BLAST. In all, 3D-BLAST assigned 244 (78.5%) proteins to SCOP superfamilies if the threshold of *E *value was set at under e^-15 ^by the SG-319 query set (Figure 9a). When the sequence identity was more than 25%, 98.4% (120 out of 122) of these cases could be assigned to a SCOP superfamily by 3D-BLAST, and 62.9% (124 out of 197) of the remaining proteins could also be assigned.

The following observations help in comparing the characteristics and performance between applying 3D-BLAST to SG-319 (Figure 9a) and applying it to SCOP-516 (Figure 5b). First, the distribution of the sequence identity of these two sets was significantly different. The sequence identities of 197 (61.8%) and 154 (29.85%) proteins in SG-319 and SCOP-516, respectively, were under 25%. The average sequence identity in SG-319 is significantly lower than that of SCOP-516. Second, the assigned parentages of SG-391 and SCOP-516 were 78.5% and 91.47%, respectively, when the *E *value was restricted to under e^-15^. If the sequence identity was under 25%, then the assigned rates were 62.9% (SG-319) and 72.12% (SCOP-516). Third, 3D-BLAST achieved similar accuracies for both sets if the sequence identity was above 25%. These observations are consistent with recent analyses of proteins targeted by structural genomics [51,52].

Figure 9b shows that 3D-BLAST assigned a structural genomics target (PDB code 1YRH) to the flavodoxin-related family [53] based on the first-rank protein (PDB code 1E5D [54]) in the hits. The *E *value was 10^-25 ^and the Z score of CE and rmsd value were 5.7 and 1.56 Å, respectively, when these two proteins were aligned. These two proteins have the same Gene Ontology (GO) annotations [55] and the same domain annotations in three databases, including PROSITE [56], Pfam [57], and CATH [31]. The aligned structures of these two proteins are similar, and the FMN-binding motifs (wireframe model) are also aligned well (Figure 9b). Eight of the top 10 proteins in the hits are the members of the same SCOP superfamily. However, PSI-BLAST was unable to yield the same assignment.

## Conclusion

This study demonstrates the robustness and feasibility of the (κ, α) plot derived structural alphabet for developing a small set of sequence-structure fragments and a fast one-against-all structure database search tool. The (κ, α) plot is an effective means of assessing the quality of protein 3D structure. The 3D-BLAST tool, which exploits the benefits of BLAST, is efficient and reasonably effective. 3D-BLAST is significantly better than PSI-BLAST at 25% sequence identity or less, and is as fast as BLAST. The structural alphabet and matrix SASM achieve good agreement for protein structures and biologic inference. Future investigations can adopt the (κ, α) plot derived 3D fragment library to develop a small 3D fragment library and predict protein structures. Moreover, many sequence-based methods can be applied to mine biologic meanings quickly from protein structures based on this 23-state structural alphabet. Finally, 3D-BLAST can be adopted to develop multiple structure alignment and structure profile search methods.

## Materials and methods

### (κ, α) Plot cluster and structural alphabet

To code the structural alphabet and calculate the substitution matrix we selected 674 structural pairs (1,348 proteins; Additional data file 1), which are structurally similar and with low sequence identity, from SCOP based on two criteria: pairs must have rmsd under 3.5 Å, with more than 70% of aligned resides included in the rmsd calculation; and pairs must have under 40% sequence identity. Based on κ and α angles, an accumulated (κ, α) plot (Figure 1b) consisting of 225,523 protein fragments, each five residues long, was obtained from these 1,348 proteins. This plot is split into 648 cells (36 × 18) when the angles of κ and α are divided by 10°. In the study, the structural distance of a pair of 5-mer protein segments *i *and *j *is determined from the rmsd value of the five C_α _atom positions, and is given as follows:

${\left\{\sum_{k=1}^{5}\left[{\left(X_{k}-x_{k}\right)}^{2}+{\left(Y_{k}-y_{k}\right)}^{2}+{\left(Z_{k}-z_{k}\right)}^{2}\right]/5\right\}}^{1/2}$

Where (*X*_*k*_, *Y*_*k*_, *Z*_*k*_) and (*x*_*k*_, *y*_*k*_, *z*_*k*_) denote the coordinates of the *k*th C_α _atom of segments *i *and *j*, respectively. The structural distance is also used to define the intrasegment and intersegment distances.

3D-BLAST used BLAST as the search method and was designed to maintain the advantages of BLAST. However, 3D-BLAST is slow if the structural alphabet is un-normalized, because the BLAST algorithm searches a statistically significant alignment by two main steps [5]. It first scans the database for words that score more than a threshold value if aligned with words in the query sequence; it then extends each such 'hit' word in both directions to check the alignment score. To reduce the ill effects of using an un-normalized structural alphabet, we set a maximum number (γ) of segments in a cluster in order to have similar compositions for the 23 structural letters and 20 amino acids. The value of γ was set to 16,000 (about 7.0% of total structural segments in the pair database).

To identify a set of 3D fragment segments (a structural alphabet), we developed a novel nearest-neighbor clustering (NNC) method to cluster 225,523 fragments in the accumulated (κ, α) plot (Figure 1b) into 23 groups. For each group, a representative segment, which represents the pattern profiles of the backbone fragments, was identified and assigned to a structural letter. These representative segments of clusters comprise a set of local structural segments. The NNC algorithm used the following steps and goals. The first step is to identify a representative structural segment, namely a center segment of all segments in this cell according to structural distances, for each cell in this (κ, α) plot. The second step is to cluster 648 representative segments into 23 groups by grouping similar representative segments into individual clusters and restricting the maximum number of segments in a cluster. In the third step, a representative segment is identified in each cluster based on the cell weight, which is defined as follows:

$w_{i}=\frac{1/S_{i}}{\sum_{j=1}^{M}1/{\text{S}}_{j}}$

Where *S*_*i *_is the number of segments in cell *i *and *M *is the number of cells in this cluster. The fourth step involves assigning the representative segment of a cluster to a structural letter. In the fifth and final step, a composition of 23 structural letters is obtained that is similar to the 20 common amino acids. We developed an NNC algorithm instead of using a standard clustering algorithm, such as a hierarchical clustering method, which is unable to achieve the second, third and fifth steps/goals.

The NNC method clustered 648 representative segments into 23 groups using the following steps. First, a structural distance matrix (*D*), stored with the rmsd values by calculating the all-against-all representative segments, is first created with a size given by *N *× *N*, where *N *denotes the number of cells. An entry (*D*_*ij*_) represents the structural distance of representative segments *i *and *j*. Second, an unlabeled cell is selected with the maximum number of segments as the seed, and labeled as *C*_*i*_. Third, an unlabeled cell is added, which represents the nearest neighbor of the seed, into this cluster and labeled as *C*_*i *_if rmsd < ε (minimum structural distance), and if the sum of segments in this group is less than a bound γ (maximum number of a cluster). This step is repeated until an added cell violates the restriction thresholds, ε or γ. Fourth, steps 2 and 3 are repeated until the number of clusters equals 22 or all of the cells are labeled. Fifth, all remaining unlabeled cells are assigned to a cluster *C*_23_. Here, ε = 0.95 Å and γ = 16,000.

A set of representative segments with 23 states and its respective structural letters are identified (Figure 2a and Additional data file 2) after performing the NNC method. Here, this 23-state structural alphabet was adopted for both protein structure reconstructions and protein structure database searches. The intrasegment structural distances (blue) are much greater than the intersegment structural distances (Figure 1c), and the average rmsd values of these 3D representative segments located in the same (or similar) cluster are frequently below 0.8 Å. The composition of the 23-state structural alphabet resembles that of the 20 amino acids obtained from the pair database. The distribution of the 23-state structural segments is consistent with that of the eight-state secondary structures defined by the DSSP program (Additional data file 3).

### BLOSUM-like substitution matrix

A substitution matrix and an SADB database are the essential components for adopting BLAST to search a structural database quickly. A new BLOSUM-like substitution matrix, called SASM (Figure 4), was developed by using a method similar to that used to construct BLOSUM62 [32] based on the pair database. The SASM (23 × 23) provides insight into substitution preferences for 3D segments between homologous structures with low sequence identity. An entry (*S*_*ij*_), which is the substitution score for aligning a structural alphabet *i*, *j *pair (1 ≤ *i*, *j *≤ 23) of the SASM matrix, is defined as *S*_*ij *_= *c*log_2_(*q*_*ij*_/*e*_*ij*_), where *c *is a scale factor for the matrix, and *q*_*ij *_and *e*_*ij *_are the observed probability and the expected probability, respectively, of the occurrence of each *i*, *j *pair. *q*_*ij *_is given as $f_{ij}/\sum_{m=1}^{23}\sum_{k=1}^{m}f_{mk}$, where *f*_*ij *_is the total number of aligning alphabets *i *to *j*. The factor *e*_*ij *_equals *p*_*i*_*p*_*j *_if *i *= *j*; otherwise, it equals 2*p*_*i*_*p*_*j *_(if i ≠ j), where *p*_*i *_is the background probability of occurrence of alphabet *i *and equals $q_{ii}+\sum_{k\neq i}^{23}q_{ik}/2$. Here, the optimal *c *value is found by testing various values ranging from 0.1 to 5.0; *c *is set to 1.89 for the best performance and efficiency. The final score *S*_*ij *_is rounded to the nearest integer value.

## Additional data files

The following additional data are available with the online version of this paper. Additional data file 1 is a table listing 674 protein pairs. Additional data file 2 is a figure showing the representative 3D fragments of the 23-state structural alphabet. Additional data file 3 is a figure showing the distributions of a 23-state structural alphabet on each kind of eight DSSP secondary structure codes. Additional data file 4 is a table showing the rmsd between X-ray structures and reconstructed structures using 23 representative segments on 38 proteins. Additional data file 5 is a figure showing an overview of 3D-BLAST for structure database search. Additional data file 6 is a table listing the average precision of each program on 108 queries in SCOP-108. Additional data file 7 is a table showing the recognition performance of nine methods on the Lindahl's benchmark dataset at the family, superfamily, and fold levels. Additional data file 8 is a table listing 976 proteins of the Lindahl's benchmark.

## Supplementary Material

Additional data file 1 Table listing 674 protein pairs. Click here for file

Additional data file 2 Figure showing the representative 3D fragments of the 23-state structural alphabet. Click here for file

Additional data file 3 Figure showing the distributions of a 23-state structural alphabet on each kind of eight DSSP secondary structure codes. Click here for file

Additional data file 4 Table showing the rmsd between X-ray structures and reconstructed structures using 23 representative segments on 38 proteins. Click here for file

Additional data file 5 Figure showing an overview of 3D-BLAST for structure database search. Click here for file

Additional data file 6 Table listing the average precision of each program on 108 queries in SCOP-108. Click here for file

Additional data file 7 Table showing the recognition performance of nine methods on the Lindahl's benchmark dataset at the family, superfamily, and fold levels. Click here for file

Additional data file 8 Table listing 976 proteins of the Lindahl's benchmark. Click here for file
