# Supplementary material for: Kappa-alpha plot derived structural alphabet and BLOSUM-like substitution matrix for rapid search of protein structure database
Source: Genome Biol. 2007 Mar 3;8(3):R31. doi: 10.1186/gb-2007-8-3-r31 (PMC1868941; doi:10.1186/gb-2007-8-3-r31)
Supplement: Additional data file 1 — Table listing 674 protein pairs. [file gb-2007-8-3-r31-S1.pdf]

Additional Data File 1: The list of 674 protein pairs selected from the SCOP

Number (#)	Protein 1		Protein 2		Aligned Percentage	RMSD (Å)	Sequence Identity
	SCOP ID	Length	SCOP ID	Length			
1	d16pk__	415	d1fw8a_	415	0.78	2.34	31.3
2	d1a02n1	102	d1imhc1	101	0.98	1.62	34.3
3	d1a1x__	106	d1jsg__	111	0.91	1.56	38.7
4	d1a2za_	220	d1auga_	210	1.00	1.23	36.3
5	d1a34a_	147	d1stma_	141	0.80	2.78	22.9
6	d1a3aa_	145	d1a6ja_	150	0.89	2.26	24.3
7	d1a49a3	135	d1a3wa3	134	1.00	1.57	39
8	d1a4ia2	125	d1edza2	146	0.75	1.66	17.1
9	d1a5t_2	207	d1l8qa2	213	0.78	3.43	15.7
10	d1a8d_1	247	d1epwa1	218	1.00	1.83	30.5
11	d1a8d_2	205	d1epwa2	211	0.91	2.26	30.9
12	d1a8e__	329	d1cb6a2	357	0.84	1.72	38.5
13	d1a8l_2	107	d1hyua3	102	0.88	2.25	18.6
14	d1a8y_1	124	d1a8y_2	102	0.95	2.62	21.4
15	d1aco_1	226	d1l5ja2	212	0.81	2.76	18.7
16	d1aco_2	527	d1l5ja3	490	0.84	2.39	21.9
17	d1adr__	76	d1ner__	74	0.85	2.97	22.2
18	d1aep__	153	d1eq1a_	166	0.91	2.36	21
19	d1ah7__	245	d1ca1_1	249	0.89	2.22	27.7
20	d1aho__	64	d1bmr__	67	0.93	1.83	39.7
21	d1ahsa_	126	d1bvp12	134	0.93	1.16	29.6
22	d1aisb2	95	d1vola1	95	0.96	1.24	22.7
23	d1ajsa_	412	d1bw0a_	412	0.85	2.86	17.8
24	d1aln_1	150	d1aln_2	144	0.74	2.92	24.4
25	d1aoha_	143	d1g1ka_	143	0.97	1.54	30.9
26	d1aqt_1	50	d1e79h1	45	1.00	1.05	14
27	d1aqt_2	85	d1e79h2	86	0.98	1.01	25.3
28	d1atg__	231	d1amf__	231	0.97	1.83	25.8
29	d1aw0__	72	d1mwza_	73	0.95	1.53	18.7
30	d1aye_2	94	d1jqga2	92	0.98	1.69	25.3
31	d1ayfa_	103	d1b9ra_	105	0.96	2.86	31.2
32	d1ayl_2	227	d1khba2	250	0.78	2.65	20.9
33	d1az9_2	264	d1chma2	246	0.93	1.95	23.3
34	d1b24a1	93	d1b24a2	79	0.87	2.28	16.7
35	d1b3qa2	132	d1k0sa_	151	0.85	2.85	26.5
36	d1b4aa1	75	d1aoy__	78	0.82	1.75	24.1
37	d1b5qa2	112	d1gosa2	112	0.99	2.77	10.4
38	d1b63a2	218	d1ei1a2	219	0.83	2.42	23
39	d1b6a_1	74	d1xgsa1	77	0.90	1.36	31.2
40	d1b6g__	310	d1bn7a_	291	0.95	2.38	30
41	d1b8aa1	103	d1g51a1	104	0.93	1.68	34.3
42	d1b8aa2	335	d12asa_	327	0.84	2.63	21.2
43	d1b8da_	164	d1allb_	161	1.00	2	26.1
44	d1b8oa_	280	d1g2oa_	262	0.98	1.38	36.7
45	d1b8pa2	171	d1ldm_2	169	0.95	2.29	18.1
46	d1b9oa_	123	d1iiza_	120	0.94	2.17	31.2
47	d1bd0a2	233	d1hkva2	265	0.78	2.99	17.2
48	d1bd8__	156	d1awcb_	153	0.98	1.7	29.1
49	d1bdya_	123	d1rlw__	126	0.84	2.88	22.1
50	d1bfg__	126	d1ihka_	157	0.80	0.97	32.3
51	d1bgva2	194	d1hwxa2	208	0.84	1.41	24.5
52	d1biha1	94	d1wiu__	93	0.97	2.27	13.7
53	d1bkpa_	278	d1qqqa_	264	0.96	1.29	35.5
54	d1bkra_	108	d1h67a_	108	0.92	2.36	20.9
55	d1bl0a2	62	d1d5ya2	65	0.95	1.04	35.4
56	d1brt__	277	d1a8q__	274	0.95	1.21	38.5
57	d1brwa3	103	d2tpt_3	105	0.98	1.49	30.5
58	d1bsg__	266	d1e25a_	278	0.91	2.21	23.6
59	d1bvoa_	175	d1a02n2	178	0.82	2.44	22.4

60	d1bvyf_	152	d1e5da1	152	0.93	2.87	21.8
61	d1byfa_	123	d1hq8a_	123	0.89	2.27	19.5
62	d1byi__	224	d1g3qa_	237	0.81	3.2	22.8
63	d1c07a_	95	d1qjta_	99	0.85	2.98	24
64	d1c0ma1	53	d1ex4a1	48	0.94	1.73	30.4
65	d1c1yb_	77	d1lfda_	87	0.85	2.51	17.8
66	d1c3ga1	80	d1c3ga2	90	0.78	1.85	20
67	d1c8da_	548	d1lp3a_	519	0.83	2.47	22.5
68	d1c8na_	189	d1f2na_	189	0.97	1.75	21.8
69	d1c9fa_	87	d1f2ri_	100	0.80	3.02	34
70	d1c9oa_	66	d1bkb_2	65	0.92	2.43	20
71	d1ccza2	78	d1cdy_2	81	0.91	2.56	18.8
72	d1cewi_	108	d1eqka_	102	0.84	2.6	25
73	d1cf7a_	67	d1cf7b_	82	0.74	1.73	26.2
74	d1cfr__	283	d1knva_	290	0.95	1.79	30.4
75	d1cg2a2	113	d1fnoa3	113	0.94	2.3	20.8
76	d1cipa1	121	d1azsc1	116	0.94	1.38	33.6
77	d1ciy_1	148	d1ji6a1	150	0.98	1.61	36.4
78	d1ciy_2	206	d1ji6a2	212	0.92	2.37	28.3
79	d1ciy_3	223	d1ji6a3	227	0.96	1.24	36.3
80	d1cjsa_	212	d1mzpa_	217	0.97	2.13	36.7
81	d1ckma2	228	d1p16a2	245	0.83	1.77	27.6
82	d1cl1a_	391	d1n8pa_	393	0.94	2.13	28.7
83	d1clxa_	345	d1foba_	334	0.83	3.36	18.2
84	d1cnv__	283	d1nar__	289	0.87	3.26	19.9
85	d1coja2	122	d1idsa2	114	0.94	1.01	28.2
86	d1cola_	197	d1cii_1	174	0.96	3.21	27.1
87	d1cqqa_	180	d1hava_	216	0.81	2.27	23.8
88	d1cqxa3	142	d1gvha3	143	0.92	2.14	35.4
89	d1csei_	63	d1lw6i_	63	0.98	1.58	37.5
90	d1csh__	435	d1k3pa_	426	0.81	2.86	26.2
91	d1csn__	293	d1phk__	277	0.91	2.72	22.9
92	d1ctqa_	166	d1o3ya_	166	0.94	2.14	20.9
93	d1cuk_1	48	d1ixsa_	50	0.96	1.38	38
94	d1cuk_2	78	d1ixra1	73	0.95	1.64	31.6
95	d1cwva2	96	d1cwva3	103	0.91	1.32	25.7
96	d1cy5a_	92	d3ygsp_	97	0.92	1.36	19.6
97	d1cyo__	88	d1cxya_	81	0.95	1.23	30.4
98	d1cyx__	158	d1ocrb1	137	0.82	1.06	21
99	d1czan1	207	d1ig8a1	207	0.90	2.8	32.4
100	d1d1da1	80	d1qrjb1	84	0.86	2.48	23.3
101	d1d1ga_	164	d1vdra_	157	0.97	2.1	21.4
102	d1d2ea1	98	d1d1na_	98	0.87	2.17	10.8
103	d1d2za_	102	d1fada_	95	0.92	3.1	22.7
104	d1d5va_	94	d1e17a_	90	0.88	2.21	36.6
105	d1d66a1	41	d1pyia1	42	0.86	1.33	39.5
106	d1d6ba_	42	d1b8wa_	42	0.90	1.45	35.7
107	d1d7pm_	159	d1kexa_	155	0.98	1.15	39.5
108	d1daaa_	277	d1iyea_	304	0.90	1.53	27.9
109	d1dbha1	207	d1foea1	206	0.85	3.17	19.1
110	d1dcia_	275	d1hzda_	266	0.97	1.83	25.2
111	d1dcja_	81	d1pava_	78	0.94	2.31	29.3
112	d1ddla_	188	d1auya_	163	0.99	1.07	37
113	d1ddwa_	111	d1mkea1	114	0.89	2.53	17.4
114	d1dfca2	119	d1dfca4	111	0.94	1.87	21.3
115	d1dgna_	89	d3crd__	100	0.82	2.44	17.6
116	d1dgwa_	178	d1od5a2	173	0.89	2.42	16.8
117	d1dhn__	121	d1b9la_	119	0.97	1.96	21.6
118	d1dk5a_	316	d1axn__	323	0.96	2.09	32.6
119	d1doka_	72	d1o80a_	74	0.92	2.32	21.8
120	d1doza_	309	d1hrka_	359	0.84	2.02	25.1
121	d1dq3a3	98	d1dq3a4	109	0.72	2.84	23.5
122	d1dqaa1	117	d1qaxa1	110	0.85	1.82	19.4
123	d1dtja_	74	d1vig__	71	0.93	2.07	15.8
124	d1dvpa1	145	d1juqa_	151	0.91	1.92	29.9

125	d1dyka1	189	d1dyka2	185	0.94	1.89	23.8
126	d1dyna_	113	d1pls__	113	0.86	2.58	20.5
127	d1dz4a_	404	d1izoa_	411	0.92	3.27	18.6
128	d1e0ca1	135	d1e0ca2	136	0.86	1.5	25.9
129	d1e0ta1	98	d1pkla1	99	0.96	0.99	30.7
130	d1e12a_	239	d1h2sa_	225	0.97	1.36	28.8
131	d1e32a3	94	d1cz4a2	94	0.90	3	25.5
132	d1e3ha2	149	d1e3ha3	137	0.85	1.79	23.6
133	d1e3ha5	111	d1e3ha6	96	0.82	2.32	23
134	d1e3oc1	57	d2hdda_	55	1.00	1.05	26.3
135	d1e4bp_	206	d1k0wa_	223	0.91	1.89	26.9
136	d1e4mm_	499	d1qvba_	481	0.83	1.98	22.9
137	d1e54a_	332	d1hxxa_	340	0.82	2.38	23.2
138	d1e58a_	247	d1h2ea_	207	0.95	2.26	25.3
139	d1e6ca_	170	d1l4ua_	165	0.90	2.35	34.9
140	d1e6ia_	110	d1f68a_	103	0.97	1.1	38.2
141	d1e6ta_	129	d1dwna_	127	0.94	2.23	18.3
142	d1e79a1	131	d1e79d1	118	0.84	3.16	23.4
143	d1e79a2	76	d1e79d2	73	0.90	1.47	17.7
144	d1e79a3	285	d1e79d3	276	0.97	1.88	25.6
145	d1e8ca1	101	d1gg4a3	98	0.91	2.35	22.2
146	d1e8ga1	287	d1diqa1	279	0.99	1.31	24.9
147	d1ea5a_	532	d1mx1a_	532	0.92	1.72	35.9
148	d1eb6a_	177	d1g12a_	167	0.92	1.94	20.7
149	d1ebda2	117	d1onfa2	117	0.96	1.55	21.8
150	d1ed8a_	449	d1ew2a_	479	0.79	2.09	29.2
151	d1edg__	380	d1cz1a_	394	0.70	3	19.4
152	d1eeoa_	297	d1jlna_	297	0.91	2.37	30.7
153	d1egwa_	71	d1mnma_	85	0.74	1.71	28.2
154	d1egza_	291	d1bhga3	304	0.82	3.23	17.4
155	d1ehia1	132	d1e4ea1	130	0.78	1.63	32.1
156	d1ei7a_	158	d1cgme_	160	0.94	3.26	38
157	d1ej2a_	167	d1lw7a1	163	0.86	2.18	20.6
158	d1ejga_	46	d1bhp__	45	1.00	0.62	34.8
159	d1el4a_	194	d2scpa_	174	0.96	3.17	17.9
160	d1eo9a_	202	d1eo9b_	238	0.72	2.06	28.2
161	d1erv__	105	d1kte__	105	0.84	3.25	18.6
162	d1esfa2	113	d1an8_2	113	0.96	1.84	29.2
163	d1eu1a1	155	d1g8ka1	143	0.88	3.19	21.5
164	d1eucb2	246	d1jkjb2	238	1.00	2.26	37.4
165	d1euma_	161	d1jgca_	160	0.96	2.01	17.9
166	d1euwa_	136	d1mq7a_	134	0.92	1.27	33.6
167	d1ew4a_	106	d1ekga_	119	0.89	1.71	24
168	d1ewqa1	275	d1e3ma1	297	0.91	1.77	38
169	d1ewqa3	146	d1e3ma3	153	0.82	1.8	26.3
170	d1ewqa4	120	d1e3ma4	115	0.93	1.07	36.1
171	d1exta1	59	d1jmab1	56	0.95	1.66	32.3
172	d1eyva_	131	d1ey1a_	139	0.88	2.84	31
173	d1ez0a_	504	d1ky8a_	499	0.89	2.35	21.6
174	d1ezm__	298	d1keia_	316	0.88	2.02	32.4
175	d1ezve2	56	d1l0le2	69	0.77	1.65	32.4
176	d1ezvf_	125	d1l0lf_	109	0.93	1.27	32
177	d1ezvg_	93	d1l0lg_	79	0.89	1.86	23.2
178	d1ezvh_	74	d1l0lh_	78	0.83	1.58	26.7
179	d1ezvi_	55	d1l0lj_	60	0.85	2.36	35
180	d1f00i3	98	d1cwva5	100	0.84	2.1	20.9
181	d1f0ya2	192	d1n1ea2	189	0.86	3.07	16
182	d1f15a_	157	d1cwpa_	149	0.91	2.18	24.3
183	d1f1ua1	146	d1mpya1	145	0.93	1.92	23.4
184	d1f20a1	270	d1ja1a1	279	0.88	2.39	28.7
185	d1f20a2	165	d1ja1a3	160	0.99	1.11	34.9
186	d1f39a_	101	d1umua_	105	0.82	1.29	28.1
187	d1f5ma_	176	d1mc0a1	187	0.78	3.12	21.4
188	d1f60a2	107	d1exma2	93	0.95	1.39	24.6
189	d1f60b_	90	d1gh8a_	89	0.97	2.88	20.7

190	d1f74a_	293	d1dhpa_	292	0.96	1.99	24.9
191	d1f7ua1	124	d1iq0a1	126	0.94	1.34	29.3
192	d1f81a_	87	d1l8ca_	95	0.75	3.19	35.6
193	d1f8ea_	388	d1nsca_	390	0.97	1.96	30.5
194	d1f94a_	63	d3ebx__	62	0.97	2.6	26.9
195	d1fc6a3	92	d1k32a1	91	0.86	2.2	21.2
196	d1fdr_2	148	d1i7pa2	147	0.85	2.2	19.8
197	d1feca3	128	d1ojt_3	128	0.84	1.98	17.7
198	d1ffya2	194	d1ile_2	189	0.96	2.35	33.5
199	d1fj2a_	229	d1auoa_	218	0.99	1.6	32.6
200	d1fjsa_	234	d1bqya_	234	0.95	1.45	27
201	d1fmja_	342	d1j99a_	284	0.90	2.37	23.9
202	d1fnf_1	94	d1lwra_	96	0.95	2.52	13.9
203	d1fo4a4	117	d1jroa3	117	0.90	1.75	18.2
204	d1fp2a1	101	d1fp1d1	110	0.84	1.68	22.8
205	d1fpoa1	76	d1hdj__	77	0.79	2.45	29.1
206	d1fpza_	176	d1d5ra2	174	0.91	2.41	19.9
207	d1fqia_	138	d1agre_	128	0.99	1.22	32.4
208	d1ftra1	148	d1ftra2	148	0.72	3.07	18.3
209	d1fvka1	64	d1bed_1	64	0.98	1.2	31.8
210	d1fvqa_	72	d1k0va_	73	0.97	2.23	27.4
211	d1fx2a_	235	d1azsa_	190	0.91	3.02	19
212	d1fx8a_	254	d1j4na_	249	0.88	2.04	28.6
213	d1fxka_	107	d1fxkc_	133	0.80	2.01	17.3
214	d1g2aa_	164	d1lmea_	154	0.94	1.25	34.7
215	d1g2qa_	178	d1l1qa_	181	0.92	2.14	35.4
216	d1g31a_	107	d1p3ha_	99	0.89	3.34	22.9
217	d1g4da_	69	d1tns__	76	0.86	1.8	36.8
218	d1g51a2	120	d1c0aa2	133	0.90	1.62	35.3
219	d1g61a_	225	d1g62a_	224	0.99	1.45	32.5
220	d1g66a_	207	d1cex__	197	0.85	2.21	20.2
221	d1g6ga_	127	d1gxca_	116	0.89	2.13	20.9
222	d1g6sa_	427	d1ejda_	419	0.95	3.11	25.1
223	d1g6xa_	58	d1ktha_	58	1.00	1.3	32.8
224	d1g9za_	152	d1m5xa_	161	0.93	1.83	34.4
225	d1ga6a_	369	d1gt91_	357	0.94	1.78	31.9
226	d1gai__	472	d1ayx__	492	0.84	1.62	32.7
227	d1gci__	269	d1ic6a_	279	0.89	1.65	36.7
228	d1gcva_	140	d1itha_	141	0.95	2.15	16.2
229	d1gd0a_	118	d1dpta_	117	0.99	1.35	32.8
230	d1gdoa_	238	d1gph12	234	0.92	1.91	27.2
231	d1ghk__	79	d1lac__	80	0.99	2.92	31.2
232	d1gjja2	43	d1jeia_	53	0.79	1.59	32.1
233	d1gk8a2	143	d1geha2	125	0.97	1.54	35.9
234	d1gk8i_	125	d1bwvs_	138	0.71	1.12	26.5
235	d1gkra2	325	d1nfga2	330	0.94	1.8	31.9
236	d1gmua1	70	d1eara1	74	0.89	2.54	22.4
237	d1gmua2	68	d1eara2	68	0.93	1.19	20.6
238	d1gp0a_	133	d1c39a_	152	0.78	2.68	21.8
239	d1gpqa_	127	d1hkea_	129	0.87	2.3	23.5
240	d1gq8a_	319	d1qjva_	342	0.83	1.78	32.1
241	d1gqva_	135	d1agi__	125	0.86	2.32	21.9
242	d1gqza1	130	d1gqza2	144	0.81	3.05	23.9
243	d1gsia_	208	d4tmka_	210	0.92	2.13	26
244	d1gt0d_	79	d1l8ya_	83	0.83	2.76	16.3
245	d1gtea3	153	d1o94a2	156	0.76	2.5	20.6
246	d1guia_	155	d1cx1a_	153	0.89	2.6	22.6
247	d1guta_	67	d1fr3a_	67	0.94	0.81	35.3
248	d1gvda_	52	d1gv2a2	47	0.96	0.72	34.6
249	d1gvea_	324	d1exba_	326	0.90	2.28	27.8
250	d1gvp__	87	d1pfsa_	78	0.95	2.74	20.5
251	d1gw5m2	141	d1gw5s_	142	0.89	2.03	23
252	d1gwea_	498	d1m7sa_	484	0.96	1.47	38.5
253	d1gwua_	306	d1jdra_	294	0.77	2.09	21.9
254	d1gx3a_	276	d1e2ta_	274	0.92	1.59	35.2

255	d1gxua_	88	d2acy__	98	0.84	2.04	29.3
256	d1gy7a_	121	d1jkga_	139	0.84	1.87	29.4
257	d1gyoa_	106	d1aqe__	110	0.95	2.34	27
258	d1gyva_	120	d1p4ua_	145	0.81	1.58	31.8
259	d1gzga_	329	d1ohla_	340	0.94	1.46	36.2
260	d1gzqa2	181	d1de4a2	178	0.96	2.88	18.5
261	d1h03p2	63	d1quba1	62	0.97	1.82	20
262	d1h0hb_	214	d1kqfb1	244	0.84	1.6	30
263	d1h1oa1	82	d1fcdc1	80	0.90	1.92	22
264	d1h3fa2	81	d1jh3a_	99	0.76	2.41	24
265	d1h4ax1	85	d1npsa_	87	0.92	2	22.8
266	d1h4ra3	84	d1gg3a3	81	0.96	1.61	33.3
267	d1h4vb1	96	d1qe0a1	95	0.96	1.78	25.3
268	d1h4xa_	111	d1auz__	116	0.96	2.73	31.9
269	d1h6ga1	131	d1h6ga2	124	0.94	2.31	21.7
270	d1h6ta2	210	d1h6ua2	227	0.90	0.98	37.1
271	d1h72c1	163	d1kkha1	180	0.84	2.75	21.3
272	d1h7ca_	103	d1qsda_	102	0.95	2.26	28.4
273	d1h8ca_	82	d1i42a_	89	0.81	2.91	21.5
274	d1h9ra1	77	d1h9ra2	62	1.00	1.28	20.8
275	d1hbka_	89	d1hb6a_	86	0.95	1.39	28.1
276	d1hc7a3	74	d1nj1a2	71	0.97	1.64	27.3
277	d1hcnb_	110	d1fl7b_	107	0.89	2.05	34.5
278	d1he1a_	135	d1g4us1	130	0.92	1.47	25
279	d1heta2	176	d1qora2	179	0.96	2.79	22.3
280	d1hlra3	117	d1jrob1	122	0.84	1.5	28.4
281	d1hlva1	66	d1iufa1	78	0.71	2.67	26.2
282	d1hm9a1	208	d1hv9a1	200	0.99	0.86	39.2
283	d1hn0a3	122	d1j0ma2	118	0.96	2.52	19.2
284	d1hpi__	71	d2hipa_	71	0.94	1.91	37.8
285	d1hqka_	154	d1di0a_	148	0.95	1.95	22.6
286	d1hqsa_	423	d1lwda_	413	0.87	2.72	22.9
287	d1hqz1_	139	d1cfya_	133	0.92	2.05	20.5
288	d1hr6a1	220	d1l0lb1	219	0.97	1.95	26.2
289	d1ht6a2	347	d1bag_2	347	0.82	2.81	23.8
290	d1hx0a2	403	d1e43a2	393	0.78	2.96	20
291	d1hy0a_	447	d1jswa_	459	0.85	3.16	20.1
292	d1hyea1	145	d1llda1	143	0.97	1.59	29.3
293	d1hzia_	129	d1ik0a_	112	0.86	2.34	23.2
294	d1i0da_	331	d1bf6a_	291	0.94	1.75	27.4
295	d1i0ra_	161	d1ejea_	192	0.77	2.03	21.5
296	d1i1wa_	303	d1h1na_	304	0.85	3.49	22.5
297	d1i5za2	132	d1rgs_2	132	0.85	2.68	22.6
298	d1i6la_	326	d1jila_	323	0.77	3.49	19.3
299	d1i6pa_	214	d1ekja_	210	0.92	2.36	25.7
300	d1i71a_	83	d2hpqp_	79	0.92	2.47	28.6
301	d1iapa_	190	d1htjf_	182	0.97	1.91	33.2
302	d1icra_	216	d1vfra_	217	0.97	1.51	34.4
303	d1id0a_	146	d1bxda_	161	0.77	2.77	25.1
304	d1idra_	126	d1ngka_	126	0.92	2.21	22
305	d1ifc__	131	d1mdc__	131	0.98	1.92	22.2
306	d1ifqa_	127	d1h8ma_	140	0.86	2.51	19.3
307	d1ifya_	49	d1f4ia_	45	0.89	1.83	21.6
308	d1ig0a1	96	d1ig3a1	85	1.00	1.71	25
309	d1ig6a_	107	d1kkxa_	102	0.85	3.47	27.8
310	d1ihga1	169	d1kt1a1	168	0.91	1.88	23.1
311	d1ikta_	115	d1c44a_	123	0.88	1.97	37.1
312	d1in0a1	88	d1in0a2	74	0.92	2.82	14.6
313	d1io0a_	166	d1pgva_	167	0.98	1.33	35.5
314	d1iow_2	210	d1e4ea2	211	0.92	1.68	33.8
315	d1iqqa_	200	d1iooa_	196	0.96	2.05	25.5
316	d1irqa_	48	d2cpga_	43	1.00	2.28	24
317	d1is3a_	134	d1a3k__	137	0.95	1.69	24.8
318	d1isia_	250	d1lbea_	250	0.97	2.17	33.6
319	d1itva_	195	d1fbl_1	195	0.93	2.02	23.5

320	d1iupa_	271	d1c4xa_	281	0.94	1.62	33.8
321	d1ivwa1	417	d1oaca1	424	0.96	1.54	31.4
322	d1ivya_	452	d1ac5__	483	0.78	2.31	27
323	d1iw0a_	207	d1n45a_	214	0.96	1.44	33.9
324	d1iwga1	97	d1iwga3	107	0.79	2.66	16.2
325	d1ix9a1	90	d1my6a1	88	0.99	1.89	35.2
326	d1ixra2	62	d1bvsa3	63	0.98	1.28	31.7
327	d1iyha1	124	d1a0fa1	121	0.97	2.36	14.1
328	d1iz5a2	121	d1plq_1	126	0.90	1.84	14.4
329	d1izca_	299	d1dxea_	253	0.97	2.07	27.5
330	d1j0aa_	325	d1tdj_1	331	0.86	2.86	23.2
331	d1j54a_	174	d1j9aa_	184	0.83	2.9	21.6
332	d1j5ua_	127	d1jw3a_	140	0.82	2.65	23.1
333	d1j5ya1	65	d1bia_1	63	0.90	1.23	36.8
334	d1j6za1	143	d1jcfa1	140	0.95	2.34	14.8
335	d1j75a_	57	d1qbja_	65	0.88	0.99	30.8
336	d1j8mf1	84	d1fts_1	84	0.90	3.19	21.1
337	d1j98a_	154	d1j6wa_	161	0.90	1.33	32.1
338	d1j9la_	247	d1l5xa_	276	0.83	2.56	29.7
339	d1jaka2	143	d1qba_4	137	0.83	2.79	22.9
340	d1jbga_	106	d1exja1	118	0.90	1.81	27.7
341	d1jc9a_	220	d1fzda_	197	0.88	1.09	32.8
342	d1jd5a_	105	d1g73d_	102	0.92	1.37	39.3
343	d1jdw__	360	d1bwda_	348	0.97	1.82	37.7
344	d1jeqa1	51	d1h1js_	44	0.91	1.97	22.2
345	d1jf8a_	130	d1d1qa_	159	0.81	1.68	22.5
346	d1jfla1	115	d1jfla2	113	0.90	2.39	26.8
347	d1jfxa_	217	d1h09a2	189	0.90	2.33	24.1
348	d1jg1a_	215	d1dl5a1	213	0.95	1.74	34.4
349	d1jhda1	173	d1g8fa1	167	0.96	2.17	23.5
350	d1jhda2	223	d1g8fa2	221	0.97	2.28	37.5
351	d1ji0a_	240	d1mt0a_	241	0.93	2.4	23.3
352	d1ji1a1	122	d1ji2a1	120	0.93	2.43	22.1
353	d1ji1a2	83	d1j0ha2	83	0.95	2.29	17
354	d1jida_	114	d1kvna_	104	0.82	2.92	25.4
355	d1jiwi_	105	d1smpi_	100	1.00	1.66	36.2
356	d1jj2f_	119	d1e7ka_	125	0.95	1.41	29.6
357	d1jj2r_	81	d1n88a_	96	0.74	1.16	27.8
358	d1jkza_	46	d1gps__	47	0.94	1.36	25
359	d1jlja_	169	d1mkza_	169	0.91	1.64	26.8
360	d1jlxa1	153	d1jlxa2	146	0.94	1.69	28.1
361	d1jm7a_	103	d1jm7b_	97	0.91	2.42	21.3
362	d1jmsa1	95	d1bpya1	82	0.98	1.49	17.7
363	d1jmsa3	60	d1jn3a1	58	1.00	1.61	26.7
364	d1jmxa1	84	d1jjua1	85	0.99	1.32	38.8
365	d1jmxa3	82	d1jjua3	78	1.00	1.84	30.5
366	d1jmxa5	119	d1jjua5	108	0.94	1.33	31.4
367	d1jnra1	141	d1neka1	138	0.91	1.78	22.9
368	d1jo8a_	58	d1cska_	57	0.98	1.08	23.7
369	d1jqia1	153	d1ivha1	151	0.98	0.96	35.9
370	d1jqia2	231	d3mdda2	231	0.97	1.15	35
371	d1jr3a1	126	d1jr3d1	127	0.84	2.86	20.9
372	d1jr8a_	105	d1oqca_	112	0.89	1.1	35.7
373	d1jsda_	317	d2viua_	320	0.98	2.03	36.5
374	d1jssa_	199	d1ln1a_	203	0.94	3.02	17.8
375	d1jt6a1	71	d1pb6a1	72	0.88	1.24	28.4
376	d1jt6a2	115	d1pb6a2	126	0.84	3.25	20.4
377	d1jtaa_	361	d1o88a_	352	0.80	2.36	24.4
378	d1ju2a2	170	d1gpea2	196	0.71	2.71	19.2
379	d1ju3a1	223	d1mpxa1	233	0.84	1.29	32.4
380	d1ju3a2	347	d1mpxa2	381	0.85	3.17	26.2
381	d1jv4a_	157	d1beba_	156	0.96	2.42	18.1
382	d1jyaa_	117	d1jyoa_	130	0.85	2.45	14.2
383	d1jyka_	229	d1i52a_	225	0.85	2.9	18.7
384	d1jz8a1	114	d1jz8a2	105	0.81	2.45	20.6

385	d1jz8a3	207	d1bhga2	204	0.80	2.53	22.2
386	d1k0ra2	79	d1k0ra3	67	0.94	2.27	28.4
387	d1k2yx3	109	d3pmga3	117	0.91	2.36	19.5
388	d1k3ia2	162	d1eut_2	142	0.96	1.16	29.6
389	d1k3xa1	89	d1nnja1	88	0.97	2.16	22.8
390	d1k3xa2	124	d1k82a2	128	0.91	2.17	22.1
391	d1k3xa3	40	d1nnja3	48	0.77	0.6	33.3
392	d1k4cc_	103	d1p7ba2	116	0.78	2.93	27.2
393	d1k4ga_	372	d1iq8a1	355	0.93	2.27	26.3
394	d1k4za_	157	d1k8fa_	157	0.97	1.41	36.5
395	d1k5ca_	333	d1hg8a_	349	0.91	1.44	36.6
396	d1k5nb_	100	d1ow0a1	101	0.94	3.33	16.7
397	d1k7ja_	206	d1jcua_	208	0.87	3.12	28.7
398	d1k8kd2	163	d1k8kf_	166	0.74	2.69	18.4
399	d1k8ua_	89	d1mr8a_	90	0.90	2.9	23.9
400	d1k92a1	188	d1j20a1	165	0.99	1.83	26.8
401	d1k94a_	165	d1alva_	173	0.92	2.03	33.5
402	d1k9sa_	237	d1lx7a_	250	0.90	1.88	27.5
403	d1ka9h_	195	d1qdlb_	195	0.89	2.6	22.2
404	d1kb0a1	97	d1kv9a1	104	0.81	1.88	31.1
405	d1kb0a2	573	d1g72a_	571	0.91	1.85	33.8
406	d1kcqa_	103	d1svy__	102	0.96	1.26	35.5
407	d1kfwa2	61	d1ll7a2	62	0.95	1.29	25.4
408	d1kgna_	296	d1h0oa_	288	0.92	2.04	21.4
409	d1khia1	76	d1bkb_1	71	0.87	2.3	23.5
410	d1kit_1	192	d1kit_2	197	0.75	1.61	17
411	d1kjqa2	111	d1dv1a2	113	0.82	2.08	18.4
412	d1kk6a_	207	d1xat__	208	0.89	1.83	36.7
413	d1kk8a1	48	d2mysa1	46	1.00	1.15	31.2
414	d1kkha2	137	d1kvka2	169	0.75	1.98	22.1
415	d1klil_	61	d1fjsl_	52	0.98	1.22	36.1
416	d1km3a_	212	d1kv8a_	213	0.89	1.99	25.6
417	d1kmoa_	661	d1by5a_	697	0.85	3.01	21.1
418	d1knga_	144	d1o8xa_	143	0.90	3.36	21.2
419	d1knma_	129	d1ggpb2	128	0.93	1.66	25
420	d1ko7a1	129	d1knxa1	132	0.97	2.11	24.1
421	d1kq1a_	60	d1hk9a_	64	0.92	0.71	30.8
422	d1kr4a_	110	d1p1la_	102	1.00	0.99	38.2
423	d1krha1	100	d1ep3b1	101	0.93	2.11	18.6
424	d1krha2	133	d2pia_2	120	0.93	2.61	26.1
425	d1ks8a_	433	d1clc_1	441	0.91	2.45	24.7
426	d1ksia2	93	d1a2va2	98	0.92	1.71	18.6
427	d1ktga_	137	d1k2ea_	152	0.80	2.25	19.8
428	d1kv7a2	165	d1gska2	174	0.93	2.18	24.7
429	d1kwaa_	88	d1m5za_	91	0.91	2.36	17
430	d1kwga2	393	d1j18a2	417	0.80	3.32	18
431	d1kx5b_	102	d1kx5d_	122	0.70	2.87	17.6
432	d1kxga_	144	d1tnra_	144	0.88	1.79	23.4
433	d1kyfa1	133	d1e42a1	120	0.87	3.03	22.1
434	d1kyfa2	114	d1e42a2	113	0.94	2.27	19.7
435	d1kzka_	99	d1fmb__	104	0.94	1.61	33.6
436	d1kzla2	110	d1i8da2	113	0.94	1.19	30.7
437	d1kzqa1	129	d1kzqa2	124	0.90	1.75	26.1
438	d1l0ia_	77	d1af8__	86	0.86	3.22	27.3
439	d1l0qa1	90	d1b4ra_	80	0.95	2.17	24.2
440	d1l1sa_	111	d1jx7a_	117	0.93	1.83	18.9
441	d1l3ka2	79	d1cvja1	80	0.96	1.59	18.3
442	d1l3la1	65	d1fsea_	67	0.97	1.02	22.4
443	d1l3pa_	102	d1nlxa_	104	0.86	3.17	27.9
444	d1l3wa4	107	d1l3wa5	107	0.88	2.74	20.3
445	d1l5oa_	346	d1j33a_	333	0.98	1.54	36.8
446	d1l9na1	140	d1g0da1	135	0.96	2.15	26.1
447	d1l9na2	114	d1f13a2	112	0.99	1.26	17.4
448	d1lam_1	159	d1gyta1	178	0.80	2.95	19.9
449	d1lb2b_	72	d1doqa_	69	0.96	2.25	28.4

450	d1lb6a_	155	d1czya1	152	0.92	1.55	31.5
451	d1lbva_	252	d1g0ha_	252	0.96	1.86	35.7
452	d1lcya1	100	d1ky9a1	94	0.94	2.69	20.2
453	d1ld8b_	407	d1dceb_	329	0.93	1.83	26.3
454	d1lfpa_	243	d1mw7a_	220	0.97	2.56	30.9
455	d1li1a1	113	d1li1a2	115	0.92	1.75	36.1
456	d1lj5a_	379	d1k9oi_	376	0.90	3.06	31.3
457	d1lj9a_	144	d1lnwa_	141	0.95	2.62	17
458	d1lki__	172	d1i1rb_	167	0.91	3.48	17.9
459	d1lkka_	105	d1d4ta_	104	0.91	1.6	24.8
460	d1llna_	262	d1ift__	258	0.95	2.62	24.4
461	d1lnia_	96	d1a2pa_	108	0.73	2.58	28.7
462	d1lo7a_	140	d1njka_	133	0.97	2.24	18.9
463	d1loka_	291	d1qq9a_	276	0.91	1.97	25.6
464	d1lpla_	95	d1ixda_	104	0.81	3.31	24.3
465	d1lssa_	132	d1lsua_	134	0.96	1.81	23.5
466	d1lvaa3	64	d1lvaa4	60	0.97	1.93	14.9
467	d1lvfa_	106	d1hs7a_	97	0.87	2.5	17.3
468	d1lxja_	104	d1lxna_	99	0.98	1.41	26.7
469	d1lzla_	317	d1jjia_	311	0.96	1.9	35.2
470	d1m15a1	94	d1qh4a1	101	0.90	2.37	35.3
471	d1m1na_	477	d1miob_	457	0.85	2.78	22.9
472	d1m1qa_	90	d1qo8a1	101	0.79	2.41	32.7
473	d1m1sa_	109	d1grwa_	124	0.86	2.04	20.3
474	d1m1xa1	160	d1m1xa2	139	0.83	3.34	17.9
475	d1m2oa1	103	d1m2vb1	107	0.87	1.52	20.4
476	d1m2xa_	219	d1a7ta_	227	0.96	1.5	29.3
477	d1m4la_	307	d1jqga1	317	0.93	1.56	29.9
478	d1m5ya2	107	d1m5ya3	108	0.94	2.22	26.5
479	d1m9fc_	146	d1em9a_	147	0.88	2.91	21.6
480	d1m9sa2	75	d1m9sa3	86	0.80	1.93	24.1
481	d1mba__	146	d1ash__	147	0.96	1.84	15.1
482	d1mc2a_	122	d1le6a_	123	0.97	1.35	33.3
483	d1me4a_	215	d1iwda_	215	0.97	1.17	38.1
484	d1mf7a_	194	d1ijba_	202	0.89	1.97	19.7
485	d1mgqa_	74	d1d3ba_	72	0.96	2.07	26
486	d1mgta1	81	d1sfe_1	84	0.95	1.2	36
487	d1mgta2	88	d1sfe_2	81	0.89	2.67	18.9
488	d1mixa1	114	d1h4ra1	111	0.89	1.52	22
489	d1mixa2	92	d1gg3a2	92	0.89	1.72	15
490	d1mk4a_	157	d1qsma_	150	0.91	3.11	17.9
491	d1ml4a2	157	d1js1x2	161	0.86	2.59	16.9
492	d1mla_2	70	d1nm2a2	62	1.00	1.66	32.4
493	d1mooa_	256	d1jd0a_	260	0.97	1.44	35.7
494	d1mpga1	183	d1ko9a1	188	0.84	2.15	25.2
495	d1mtza_	290	d1qtra_	314	0.82	3.12	24.5
496	d1mu5a2	164	d1ei1a1	172	0.72	3.04	18.6
497	d1muga_	165	d1l9ga_	191	0.79	2.74	23.8
498	d1mun__	225	d1keaa_	217	0.93	1.79	25.2
499	d1muwa_	386	d1a0ca_	437	0.86	2.42	26.4
500	d1mxia_	156	d1ipaa1	158	0.91	2.37	23.8
501	d1mzga_	144	d1ni7a_	149	0.91	2.52	31.4
502	d1n0ua4	79	d1fnma4	79	0.92	1.42	34.1
503	d1n55a_	249	d1aw1a_	255	0.96	1.2	38.4
504	d1n5ua3	196	d1kxpd2	190	0.97	2.76	20.8
505	d1n62a1	82	d1fo4a1	73	0.96	1.75	37.8
506	d1n62b2	663	d1fo4a5	638	0.92	2.42	23.3
507	d1n62c2	177	d1jroa4	167	1.00	1.68	29.8
508	d1n7oa1	371	d1j0ma1	361	0.99	1.62	24.5
509	d1n7oa3	274	d1hn0a4	281	0.93	2.7	21.2
510	d1n8yc2	166	d1igra1	149	0.94	2.86	28.3
511	d1n9la_	109	d1bywa_	110	0.95	1.06	31.2
512	d1nekd_	113	d1kf6d_	119	0.87	3.21	22.3
513	d1nepa_	130	d1ktja_	129	0.98	1.92	25.4
514	d1nezg_	122	d1hxmb1	123	0.93	1.66	18.3

515	d1nf9a_	207	d1yaca_	204	0.77	1.91	19.5
516	d1ng4a2	88	d1an9a2	93	0.91	1.58	20.2
517	d1nhp_3	126	d3lada3	124	0.94	2.56	21.2
518	d1ni4b1	192	d1qs0b1	204	0.87	1.13	33.8
519	d1nkl__	78	d1m12a_	84	0.90	2.4	19
520	d1nkzb_	41	d1lghb_	43	0.91	1.09	29.5
521	d1nlwa_	79	d1am9a_	80	0.94	2.31	27.4
522	d1noa__	113	d1akp__	114	0.96	2.65	39
523	d1nox__	200	d1f5va_	240	0.72	2.01	23
524	d1nr0a1	311	d1nr0a2	299	0.95	2.58	19.4
525	d1nwaa_	168	d1fvga_	192	0.79	1.17	30.7
526	d1nxma_	194	d1ep0a_	183	0.93	2.16	31.9
527	d1ny721	182	d1a6ca1	176	0.87	2.7	14.5
528	d1nyta1	170	d1npya1	167	0.93	2.5	22.2
529	d1nyta2	101	d1npya2	102	0.93	1.44	24.3
530	d1nz0a_	109	d1a6f__	113	0.88	1.54	28.8
531	d1o0ia_	137	d1psua_	131	0.90	2.08	25.2
532	d1o0wa1	169	d1jfza_	148	0.98	2.03	31.4
533	d1o0wa2	69	d1stu__	68	0.97	2.57	23.6
534	d1o13a_	107	d1eo1a_	124	0.85	2.16	30.2
535	d1o17a1	70	d1brwa1	70	1.00	1.52	21.4
536	d1o2da_	358	d1kq3a_	364	0.89	3.11	24.9
537	d1o70a1	140	d1o70a2	156	0.85	2.52	22
538	d1o7ia_	115	d1fgua1	118	0.87	2.58	20.5
539	d1o7ka_	124	d1kmda_	117	0.79	2.54	15
540	d1o97d1	192	d1efva1	188	0.97	1.7	21.8
541	d1o9aa2	49	d1fbr_2	47	0.96	1.56	33.3
542	d1obfo2	162	d1cf2o2	165	0.83	1.81	18.9
543	d1oc7a_	364	d1dysa_	345	0.98	1.67	37.6
544	d1ocra_	514	d1ffta_	501	0.99	1.45	39.3
545	d1ocrb2	90	d1fftb2	91	0.93	2.19	21.2
546	d1odma_	329	d1gp6a_	349	0.82	3.16	20.6
547	d1ofua1	198	d1tuba1	245	0.75	3.07	18.1
548	d1ogwa_	76	d1m94a_	73	0.99	1.21	20.8
549	d1oh0a_	125	d1ocva_	125	0.98	1.1	33.9
550	d1ohta_	173	d1lba__	146	0.90	2.21	22.5
551	d1oi7a1	121	d1iuka_	136	0.83	2.16	23.8
552	d1on2a1	61	d1fx7a1	64	0.92	1.48	30.8
553	d1on2a2	74	d1fx7a2	80	0.71	1.67	20.5
554	d1on3a1	253	d1on3a2	264	0.77	2.42	20.5
555	d1opd__	85	d1pch__	88	0.95	1.21	30.3
556	d1or7a1	68	d1ku3a_	61	0.90	1.37	24.1
557	d1ot5a1	139	d1p8ja1	136	0.96	1.89	27.7
558	d1owfa_	96	d1owfb_	94	0.98	1.68	29.9
559	d1ox0a1	256	d1ek4a1	253	0.98	1.74	34.8
560	d1oxja1	62	d1dxsa_	57	0.95	1.63	20
561	d1ozna_	284	d1p9ag_	266	0.88	2.42	27
562	d1p2fa1	97	d1opc__	99	0.90	1.72	31
563	d1p2fa2	120	d1tmy__	118	0.96	2.17	21.3
564	d1p3da1	96	d2uaga1	93	0.94	2.19	19.8
565	d1p3da3	215	d1j6ua3	207	0.94	1.85	28
566	d1p5vb_	136	d1n12a_	138	0.89	3.3	21.2
567	d1pbv__	195	d1ku1a_	211	0.88	1.58	31.8
568	d1pdua_	230	d1kv6a_	222	0.97	2.54	22.9
569	d1pe0a_	187	d1oi4a_	191	0.88	1.95	24.3
570	d1pii_1	254	d1i4na_	251	0.96	1.48	31.3
571	d1plc__	99	d1kdj__	102	0.97	2.07	38.1
572	d1poib_	260	d1m3ea2	222	0.91	2.62	22.1
573	d1poxa1	183	d1jsca1	181	0.87	1.83	21.2
574	d1prtb2	86	d1prea1	83	0.89	1.88	23.9
575	d1psda1	188	d1qp8a1	181	0.99	2.15	22.5
576	d1pxfa_	111	d1gd7a_	109	0.94	1.7	31.9
577	d1pyma_	291	d1muma_	289	0.93	2.09	27.2
578	d1qcsa1	86	d1e32a1	86	0.91	1.82	13.6
579	d1qfha1	104	d1qfha2	108	0.76	2.22	30.6

580	d1qgda3	136	d1gpua3	146	0.86	1.15	36.8
581	d1qgia_	259	d1chka_	238	0.90	2.59	22.7
582	d1qhoa2	110	d1acz__	108	0.90	2.51	30.1
583	d1qhoa4	407	d1eh9a3	400	0.79	2.33	21.1
584	d1qhva_	195	d1h7za_	191	0.90	2.54	30.1
585	d1qi9a_	555	d1qhba_	595	0.83	1.73	32.6
586	d1qj8a_	148	d1p4ta_	155	0.89	3.22	21.2
587	d1qkya_	38	d1lir__	37	0.92	1.07	25.6
588	d1qlaa3	121	d1chua3	116	0.96	2.39	38.7
589	d1qlab1	133	d1nekb1	132	0.96	1.86	27.7
590	d1qlab2	106	d1kf6b2	105	0.98	1.41	35.5
591	d1qmea1	61	d1qmea2	58	0.98	2.05	15.9
592	d1qnf_1	271	d1np7a1	279	0.92	1.63	33.8
593	d1qnf_2	204	d1np7a2	204	0.94	2.19	28.8
594	d1qovh1	215	d1eysh1	201	0.95	1.19	38.3
595	d1qovl_	281	d1qovm_	302	0.76	1.4	30.9
596	d1qpoa2	115	d1qapa2	122	0.91	1.65	32.3
597	d1qq4a_	198	d1sgpe_	185	0.97	1.41	35.6
598	d1qqga1	103	d1qqga2	104	0.81	1.93	15
599	d1qqp3_	220	d2mev3_	231	0.94	1.5	31.5
600	d1qs1a1	205	d1giqa1	207	0.94	2.75	24.2
601	d1qtoa_	122	d1ecsa_	120	0.95	1.62	24.4
602	d1qupa2	72	d1afj__	72	0.94	2.35	17.1
603	d1quua2	124	d1hcia1	125	0.93	2.04	15.7
604	d1rie__	127	d1g8kb_	133	0.79	2.31	21.2
605	d1rkd__	306	d1o14a_	319	0.91	3.08	18.8
606	d1rl6a2	89	d1jj2e2	93	0.89	2.16	18.6
607	d1rsy__	135	d1dsya_	137	0.88	1.5	31.1
608	d1ryph_	205	d1rypj_	204	0.94	1.42	21.7
609	d1scjb_	71	d1itpa_	77	0.81	2.03	20.8
610	d1seia_	130	d1i6ua_	129	0.90	1.86	31.6
611	d1sfp__	111	d1sppb_	112	0.91	1.65	36.3
612	d1sgpi_	51	d1tbrr1	51	0.86	1.4	30.9
613	d1svb_1	93	d1okea1	97	0.94	1.48	32.3
614	d1svpa_	160	d1befa_	177	0.73	2.81	23
615	d1tafa_	67	d1tafb_	69	0.91	1.64	18.1
616	d1tfe__	142	d1efub2	143	0.85	1.37	33.8
617	d1thfd_	253	d1qo2a_	241	0.91	2.27	22.4
618	d1toaa_	277	d1psza_	286	0.94	1.71	31.3
619	d1tuba2	195	d1tubb2	184	1.00	1.42	34.9
620	d1tvxa_	64	d1f2la_	64	0.91	1.39	23.9
621	d1tx4a_	196	d1pbwa_	184	0.97	2.73	21
622	d1ubkl_	534	d1cc1l_	487	0.93	1.41	38.1
623	d1uox_1	136	d1uox_2	159	0.79	2.27	14.4
624	d1uroa_	357	d1j93a_	343	0.99	1.79	35.3
625	d1utea_	302	d4kbpa2	312	0.79	2.17	21.1
626	d1uxy_1	198	d1hska1	194	0.89	1.72	25
627	d1vin_1	128	d1bu2a1	127	0.99	1.77	26
628	d1vjw__	58	d1fxra_	64	0.91	1.58	29.7
629	d1wba__	171	d1avac_	181	0.90	2.44	21.9
630	d1whi__	122	d1jj2j_	132	0.89	1.37	36.8
631	d1xxaa_	71	d1b4ba_	71	0.96	2.05	34.7
632	d1ycsb1	130	d1myo__	118	0.97	2.65	24.4
633	d1ytba1	95	d1aisa1	92	0.92	1.4	38.4
634	d1zpda2	186	d1jsca2	188	0.93	2.42	21.8
635	d1zpda3	204	d1ovma3	196	0.97	1.31	29.5
636	d2aak__	150	d1c4zd_	144	0.99	1.55	25
637	d2ae2a_	259	d1qg6a_	257	0.96	1.98	23.4
638	d2apr__	325	d1lf2a_	329	0.95	2.05	25.7
639	d2bbkh_	355	d1mdah_	368	0.95	0.8	30.9
640	d2bpa2_	175	d1m06g_	187	0.93	1.46	32.1
641	d2cbla1	86	d1qasa1	94	0.82	3.46	18.8
642	d2ccya_	127	d1mqva_	123	0.98	1.58	26.8
643	d2cpl__	164	d1lopa_	164	0.88	1.66	30.2
644	d2dkb__	431	d2gsaa_	427	0.91	2.19	24.9

645	d2dora_	311	d1ep3a_	311	0.93	2.44	29.4
646	d2dri__	271	d1jyea_	271	0.95	2.13	24
647	d2gsta2	84	d1gnwa2	84	0.93	2.39	17.6
648	d2igd__	61	d1hz6a_	67	0.85	2.13	25.7
649	d2lisa_	131	d1gaka_	137	0.90	2.93	21.4
650	d2mnr_1	227	d1jpma1	234	0.94	2.01	23.3
651	d2mnr_2	130	d1jpma2	125	0.91	1.94	27.9
652	d2napa2	597	d1aa6_2	564	0.96	1.74	31.8
653	d2nlra_	222	d1oa2a_	218	0.95	1.71	30.3
654	d2pgd_1	297	d1pgja1	300	0.97	1.42	30.2
655	d2plc__	274	d2ptd__	296	0.86	2.11	29.4
656	d2pola1	122	d2pola2	122	0.97	2.07	18.3
657	d2pspa1	53	d2pspa2	53	0.89	1.33	35.1
658	d2sak__	121	d1qqra_	138	0.75	2.89	19.2
659	d2tgi__	112	d1lxia_	104	0.95	1.83	33.3
660	d2trxa_	108	d1f9ma_	112	0.93	1.4	31.6
661	d2uaga2	140	d1gg4a1	135	0.93	2.45	20
662	d3chbd_	103	d1qb5d_	99	0.95	2.48	16.2
663	d3cla__	213	d1eaf__	243	0.73	2.82	23.1
664	d3grx__	82	d1h75a_	76	0.99	1.86	19.5
665	d3kvt__	103	d1nn7a_	105	0.93	1.47	38
666	d3nul__	130	d1acf__	125	1.00	1.69	37.7
667	d3tgl__	265	d1tib__	269	0.94	1.82	33.1
668	d3tss_1	89	d1an8_1	93	0.89	1.94	22.5
669	d451c__	82	d1gks__	78	0.95	3.07	20.9
670	d6mhta_	327	d1dcta_	324	0.84	2.04	31
671	d7a3ha_	300	d1bqca_	302	0.87	2.48	22
672	d7reqa1	557	d7reqb1	460	0.95	2.12	24.8
673	d7reqa2	168	d7reqb2	163	0.86	3.29	18.4
674	d7taa_2	381	d1ji2a3	382	0.86	2.18	23.3
