# Supplementary figures and images for: Kappa-alpha plot derived structural alphabet and BLOSUM-like substitution matrix for rapid search of protein structure database
Source: Genome Biol. 2007 Mar 3;8(3):R31. doi: 10.1186/gb-2007-8-3-r31 (PMC1868941; doi:10.1186/gb-2007-8-3-r31)

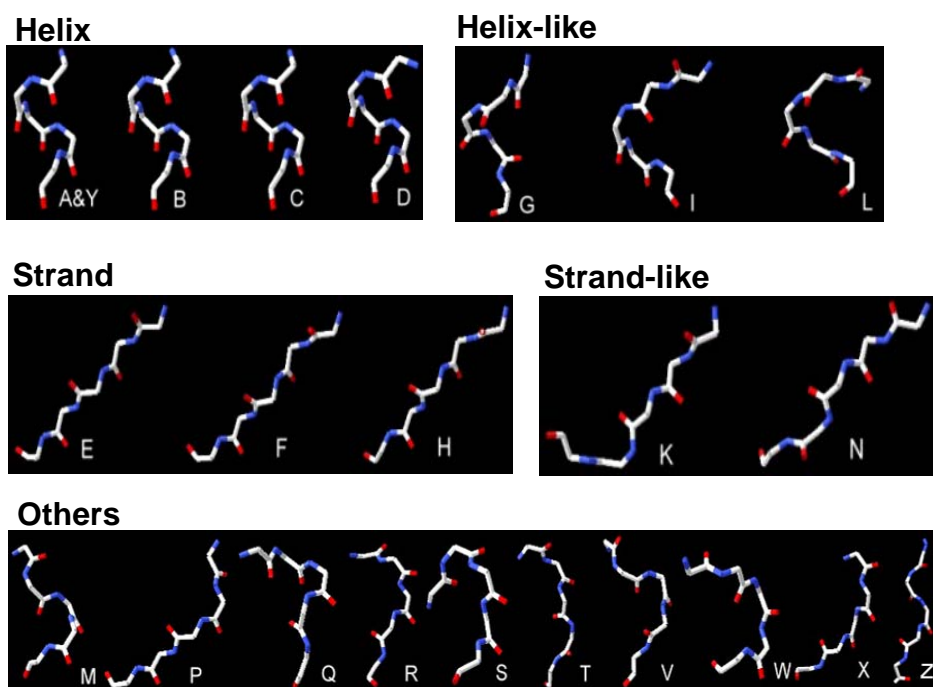

**Additional Data File 2:** The representative 3D fragments of the 23-state structural alphabet.

Supplement: Additional data file 2 — Figure showing the representative 3D fragments of the 23-state structural alphabet. [file gb-2007-8-3-r31-S2.pdf]
