# Supplementary material for: Kappa-alpha plot derived structural alphabet and BLOSUM-like substitution matrix for rapid search of protein structure database
Source: Genome Biol. 2007 Mar 3;8(3):R31. doi: 10.1186/gb-2007-8-3-r31 (PMC1868941; doi:10.1186/gb-2007-8-3-r31)
Supplement: Additional data file 3 — Figure showing the distributions of a 23-state structural alphabet on each kind of eight DSSP secondary structure codes. [file gb-2007-8-3-r31-S3.pdf]

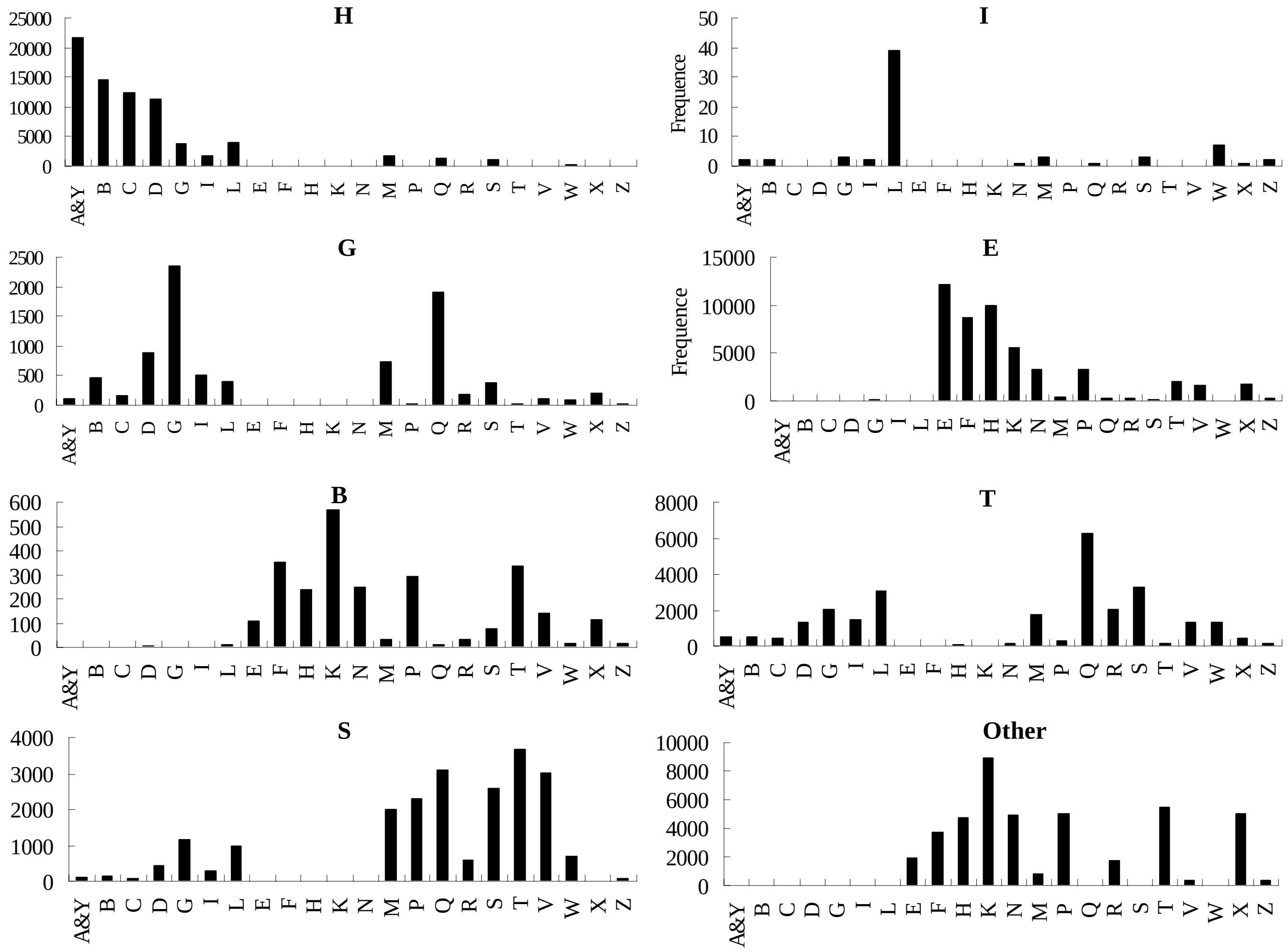

Additional Data File 3: The distributions of a 23-state structural alphabet on each kind of eight DSSP secondary structure codes, including the H, I, G, E, B, T, S, and other. In general, the H, I, and G are considered as α-helixs; the E and B considered as β-strands, and other codes are considered as coils.
