# Supplementary material for: Kappa-alpha plot derived structural alphabet and BLOSUM-like substitution matrix for rapid search of protein structure database
Source: Genome Biol. 2007 Mar 3;8(3):R31. doi: 10.1186/gb-2007-8-3-r31 (PMC1868941; doi:10.1186/gb-2007-8-3-r31)
Supplement: Additional data file 4 — Table showing the rmsd between X-ray structures and reconstructed structures using 23 representative segments on 38 proteins. [file gb-2007-8-3-r31-S4.pdf]

Additional Data File 4: The root mean square derivation between x-ray structures and reconstructing structures using 23 representative segments on 38 proteins selected from the set SCOP516

PDB code	Number of residues	SCOP family name	RMSD (Å)
1VD2-A	89	d.15.2.2	0.63
1T1V-A	93	c.47.1.14	1.10
1VB7-A	94	b.36.1.1	0.83
1SQ2-N	113	b.1.1.1	0.93
1RYU-A	120	a.4.3.1	0.73
1OZY-A	121	a.133.1.2	0.97
1T2S-A	123	b.34.14.1	0.81
1S28-A	132	d.198.1.1	0.58
1TTW-A	138	d.198.1.1	0.77
1WMU-A	141	a.1.1.2	0.66
1UFG-A	151	b.1.16.1	1.05
1TO4-A	156	b.1.8.1	1.02
1VL7-A	157	b.45.1.1	1.39
1S3Z-A	165	d.108.1.1	0.70
1PM1-X	180	b.60.1.1	1.01
1SY1-A	184	b.60.1.1	1.34
1ULI-B	187	d.17.4.4	0.96
1S6I-A	188	a.39.1.5	0.99
1T4W-A	196	b.2.5.2	0.85
1TEV-A	196	c.37.1.1	0.69
1X9G-A	200	c.33.1.3	0.94
1RO5-A	201	d.108.1.3	0.62
1PZS-A	208	b.1.8.1	0.83
1V9K-A	228	d.265.1.3	0.89
1VL1-A	232	c.124.1.1	0.98
1UD9-A	245	d.131.1.2	0.73
1QYR-A	252	c.66.1.24	0.74
1T8P-A	267	c.60.1.1	0.83
1T6L-A	290	d.131.1.2	0.97
1TUG-A	310	c.78.1.1	0.79
1J2G-A	319	d.96.1.4	0.74
1Q5M-A	322	c.1.7.1	0.81
1SYY-A	346	a.25.1.2	0.63
1U0M-A	382	c.95.1.2	0.94
1TQY-B	415	c.95.1.1	0.65
1QWO-A	442	c.60.1.2	0.81
1UKC-A	522	c.69.1.17	1.03
1V02-A	565	c.1.8.4	0.87
