# Supplementary material for: Kappa-alpha plot derived structural alphabet and BLOSUM-like substitution matrix for rapid search of protein structure database
Source: Genome Biol. 2007 Mar 3;8(3):R31. doi: 10.1186/gb-2007-8-3-r31 (PMC1868941; doi:10.1186/gb-2007-8-3-r31)
Supplement: Additional data file 5 — Figure showing an overview of 3D-BLAST for structure database search. [file gb-2007-8-3-r31-S5.pdf]

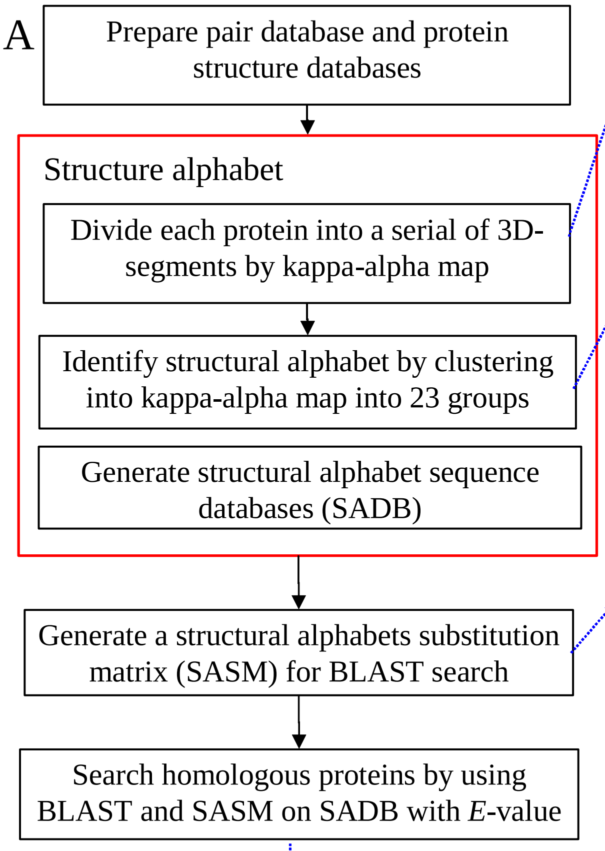

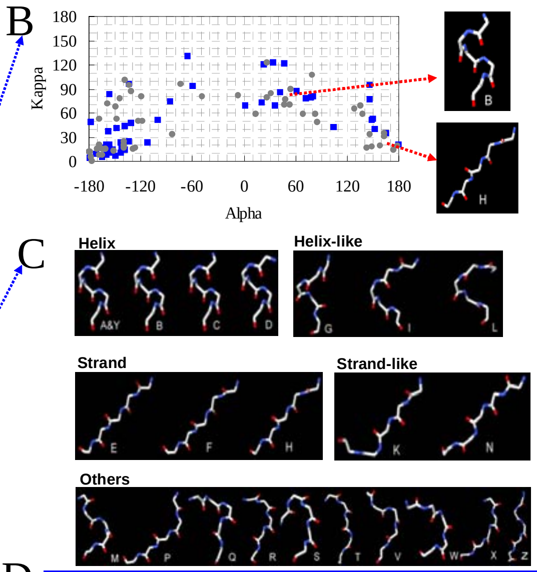

D

	A	Y	B	C	D	E	F	H	G	I	L	K	N	T	P	S	W	X	V	M	R	Q	Z
A	5	3	2	2	2	-12	-12	-9	-1	-2	0	-8	-7	-7	-7	-5	-4	-6	-6	-3	-5	-3	-4
Y	3	5	2	3	2	-15	-10	-10	-1	-2	-1	-8	-8	-7	-7	-5	-6	-7	-7	-3	-5	-3	-4
B	2	2	5	2	2	-12	-10	-10	1	-2	-2	-7	-7	-6	-6	-5	-4	-6	-5	-2	-5	-3	-4
C	2	3	2	5	1	-11	-9	-9	-1	1	-1	-8	-7	-7	-6	-5	-5	-6	-6	-3	-5	-3	-4
D	2	2	2	1	5	-10	-9	-9	1	0	1	-6	-5	-5	-5	-4	-1	-4	-4	-1	-4	-2	-3
E	-12	-15	-12	-11	-10	6	1	2	-8	-9	-8	-2	-1	-4	-4	-8	-6	-3	-4	-6	-6	-7	-3
F	-12	-10	-10	-9	-9	1	6	0	-6	-7	-7	1	-1	-3	-3	-6	-5	-2	-4	-4	-4	-5	-2
H	-9	-10	-10	-9	-9	2	0	6	-5	-6	-6	-1	2	-3	-2	-6	-4	0	-3	-4	-2	-4	-2
G	-1	-1	1	-1	1	-8	-6	-5	7	0	-1	-4	-4	-3	-3	-3	-1	-2	-1	2	-2	1	-2
I	-2	-2	-2	1	0	-9	-7	-6	0	9	3	-5	-3	-4	-4	-2	2	-3	-3	-1	-2	-1	-2
L	0	-1	-2	-1	1	-8	-7	-6	-1	3	7	-6	-5	-3	-4	-1	3	-4	-2	-2	-1	-1	-1
K	-8	-8	-7	-8	-6	-2	1	-1	-4	-5	-6	6	1	-1	-3	-4	-4	-1	-2	-3	-4	-4	0
N	-7	-8	-7	-7	-5	-1	-1	2	-4	-3	-5	1	6	1	1	-3	-3	0	-1	-3	0	-2	0
T	-7	-7	-6	-7	-5	-4	-3	-3	-3	-4	-3	-1	1	6	1	0	-1	-1	0	-2	-1	-2	-2
P	-7	-7	-6	-6	-5	-4	-3	-2	-3	-4	-4	-3	1	1	7	0	-2	-2	-2	-3	1	-2	-1
S	-5	-5	-5	-5	-4	-8	-6	-6	-3	-2	-1	-4	-3	0	0	8	2	-3	-1	-4	-2	-2	-2
W	-4	-6	-4	-5	-1	-6	-5	-4	-1	2	3	-4	-3	-1	-2	2	11	-2	2	-1	-2	-1	-2
X	-6	-7	-6	-6	-4	-3	-2	0	-2	-3	-4	-1	0	-1	-2	-3	-2	7	1	2	1	-1	0
V	-6	-7	-5	-6	-4	-4	-4	-3	-1	-3	-2	-2	-1	0	-2	-1	2	1	8	2	-2	-3	-1
M	-3	-3	-2	-3	-1	-6	-4	-4	2	-1	-2	-3	-3	-2	-3	-4	-1	2	2	7	-2	-1	-2
R	-5	-5	-5	-5	-4	-6	-4	-2	-2	-2	-1	-4	0	-1	1	-2	-2	1	-2	-2	8	3	-2
Q	-3	-3	-3	-3	-2	-7	-5	-4	1	-1	-1	-4	-2	-2	-2	-2	-1	-1	-3	-1	3	6	-2
Z	-4	-4	-4	-4	-3	-3	-2	-2	-2	-2	-1	0	0	-2	-1	-2	-2	0	-1	-2	-2	-2	9

E

Protein Code	Length	Score	*E*-value	Identity	Title
1o91A	127	241	1e-65	70.1	crystal structure of a collagen viii nc1 domain trimer
1pk6C	129	159	8e-41	43.4	globular head of the complement system protein c1q
1c3hA	130	153	5e-39	44.6	acrp30 calcium complex
1dg6A	144	92.5	9e-21	37.5	crystal structure of apo2l/trail
1rj8A	100	86.4	6e-19	35.0	the crystal structure of tnf family member eda-a2
1kxgA	137	86.0	8e-19	33.6	the 2.0 ang resolution structure of blys, b lymphocyte stimulator.
1aly	93	80.3	4e-17	40.9	crystal structure of human cd40 ligand
4tsvA	106	76.8	4e-16	36.8	high resolution crystal structure of a human tnf-alpha mutant

F

Structural alphabet sequence:

```
  Identities = 35/100 (35%), Positives = 70/100 (70%)

1gr3A:  33 EMLSRPEFHMSXVPFHEEEEFFHX--WVTHHEFNEVWQPHMPEEHFFEKVLP--HFEEHEEKHFHETLQTEXKHKHMPGDQXHHNNNLCPEHNHHHKHVT 128
1rj8A:  38 EGLSXTHFFXTRTPHHHHEEFEHMRHVWPHHFFNHVSRTNMPEHHHHKXTGSTEHKHEHHHKHKHFKLQTEHKNFKKRTQTEFEHTYLARNH-HFFENVT 136
              b          c              d        e              f            g                       h
```

Amino acid sequence:

```
  Identities = 12/100 (12%)

1gr3A:  33 DPRTGIFTCQIPGIYYFSYHVHVK--GTHVWVGLYKNGTPVMYTYDEYTKGY--LDQASGSAIIDLTENDQVWLQLPNAESNGLYSSEYVHSSFSGFLVA 128
1rj8A:  38 HPRSGELEVLVDGTYFIYSQVYYINFTDFASYEVVVDEKPFLQCTRSIETGKTNYNTCYTAGVCLLKARQKIAVKMVHADISINMSKHTTFF-GAIRLGE 136
              b          c              f        e              f            g                       h
```

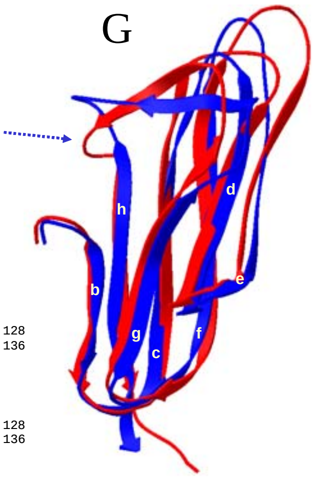

Additional Data File 5: Overview of 3D-BLAST for structure database search. (A) Seven steps of developing 3D-BLAST. (B) The (κ, α) map of two proteins (PDB code 1gr3A (blue) and 1rj8A (gray)). (C) The representative 3D fragments of 23 structural alphabets. (D) Structural alphabets substitution matrix (SASM). The scores are high when similar alphabets are aligned, e.g., helix alphabets (A, Y, B, C, and D) aligned with helix alphabets (yellow blocks). Conversely, scores are low when helix alphabets are aligned with strand alphabets (orange blocks). (E) Search results using human collagen X NC1 trimer (PDB code 1gr3A) as the query based on structural alphabet sequences. (F) Alignment results for both structural alphabets and amino acid sequences between the query and the hit protein, TNF family member EDA-A2 (PDB code 1rj8A). The locations of secondary structures (green boxes) are indicated as b, c, d, e, f, g, and h. The structural alphabet sequence is more conserved than the amino acid sequence. (G) The resulting structure alignment for the query protein (blue) and the EDA-A2 (red). The positions of secondary structures (b, c, d, e, f, g, and h) are respective these shown in (F) from N-terminal to C-terminal.
