# Supplementary material for: Kappa-alpha plot derived structural alphabet and BLOSUM-like substitution matrix for rapid search of protein structure database
Source: Genome Biol. 2007 Mar 3;8(3):R31. doi: 10.1186/gb-2007-8-3-r31 (PMC1868941; doi:10.1186/gb-2007-8-3-r31)
Supplement: Additional data file 6 — Table listing the average precision of each program on 108 queries in SCOP-108. [file gb-2007-8-3-r31-S6.pdf]

Additional Data File 6: The average precision of each program on 108 queries in SCOP-108

SCOP id	SCOP sccs	length	number of members	3D-BLAST	PSI-BLAST	CE	MAMMOTH
1A8H_2	c.26.1.1	89	5	71%	80%	87%	91%
1AFWA2	c.95.1.1	93	1	67%	33%	70%	73%
1AJSA_	c.67.1.1	94	42	88%	90%	86%	89%
1ATG__	c.94.1.1	113	538	72%	21%	90%	89%
1AW1A_	c.1.1.1	120	3	100%	100%	100%	100%
1B3RA1	c.2.1.4	121	43	75%	83%	38%	56%
1B5EA_	d.117.1.1	123	3	100%	100%	100%	100%
1BD3A_	c.61.1.1	132	7	63%	36%	97%	73%
1BG2__	c.37.1.9	138	7	58%	53%	53%	51%
1BGVA2	c.58.1.1	141	68	100%	100%	100%	100%
1BI5A1	c.95.1.2	151	2	50%	50%	58%	53%
1BU6O1	c.55.1.4	156	12	50%	50%	50%	50%
1BWVS_	d.73.1.1	157	4	100%	100%	100%	100%
1CCWA_	c.23.6.1	163	3	82%	80%	94%	100%
1CE7A_	d.165.1.1	165	19	100%	100%	100%	100%
1CJWA_	d.108.1.1	180	28	97%	35%	77%	97%
1CP2A_	c.37.1.10	184	28	54%	58%	68%	56%
1CPT__	a.104.1.1	187	1	100%	100%	100%	100%
1D3GA_	c.1.4.1	188	48	65%	51%	48%	55%
1DBFA_	d.79.1.2	196	2	100%	100%	100%	100%
1DBQA_	c.93.1.1	196	38	95%	65%	94%	84%
1DI0A_	c.16.1.1	200	5	100%	100%	100%	86%
1DK5A_	a.65.1.1	201	1	93%	100%	100%	100%
1DPGA2	d.81.1.5	206	61	67%	67%	100%	100%
1DSSG2	d.81.1.1	208	12	93%	73%	100%	100%
1DZKA_	b.60.1.1	215	1	90%	79%	100%	99%
1E0TA2	c.1.12.1	228	3	100%	100%	100%	100%
1E0TA3	c.49.1.1	232	3	100%	100%	100%	100%
1E4FT1	c.55.1.1	245	14	9%	6%	24%	6%
1E6TA_	d.85.1.1	252	3	100%	70%	100%	100%
1EAL__	b.60.1.2	261	3	98%	100%	100%	100%
1EJ8A_	b.1.8.1	267	5	100%	42%	94%	50%
1EKXA1	c.78.1.1	290	14	47%	47%	48%	47%
1EP3B1	b.43.4.2	297	4	77%	8%	95%	96%
1EU3A1	b.40.2.2	310	15	68%	52%	100%	74%
1EUAA_	c.1.10.1	313	55	16%	6%	17%	18%
1EUHA_	c.82.1.1	319	2	100%	100%	100%	100%
1EXQA_	c.55.3.2	322	18	100%	100%	95%	100%
1EYZA3	d.142.1.2	346	13	86%	78%	81%	96%
1F3MC_	d.144.1.1	382	4	100%	100%	100%	100%
1F4PA_	c.23.5.1	415	22	77%	85%	74%	84%
1F86A_	b.3.4.1	415	1	100%	100%	100%	100%
1FC4A_	c.67.1.4	422	2	57%	61%	64%	58%
1FECA3	d.87.1.1	442	6	85%	83%	95%	90%
1FJEB2	d.58.7.1	477	19	83%	100%	95%	93%
1FSOA_	b.1.1.5	522	10	99%	88%	100%	99%
1FXOA_	c.68.1.6	542	48	100%	100%	100%	100%
1G3NC1	a.74.1.1	544	3	43%	34%	50%	74%
1G5TA_	c.37.1.11	565	11	34%	13%	67%	46%
1G7SA2	b.43.3.1	89	5	96%	9%	100%	94%
1GEHA1	c.1.14.1	93	1	100%	100%	100%	100%
1GGXA_	d.22.1.1	94	42	100%	100%	100%	100%
1GNIA3	a.126.1.1	113	538	86%	100%	100%	96%
1GR3A_	b.22.1.1	120	3	100%	22%	99%	90%
1GSOA2	c.30.1.1	121	43	44%	17%	70%	61%
1GTMA1	c.2.1.7	123	3	74%	47%	76%	86%
1H4VB2	d.104.1.1	132	7	65%	41%	92%	57%
1H8VA_	b.29.1.11	138	7	99%	45%	100%	100%
1HQSA_	c.77.1.1	141	68	100%	100%	100%	100%
1HR6A2	d.185.1.1	151	2	87%	44%	100%	98%
1HYHA2	d.162.1.1	156	12	100%	100%	100%	100%

1I1RA1	b.1.2.1	157	4	3%	2%	7%	5%
1IDSA2	d.44.1.1	163	3	100%	100%	100%	100%
1IE9A_	a.123.1.1	165	19	100%	100%	100%	100%
1IG8A1	c.55.1.3	180	28	50%	50%	52%	64%
1IH7A1	c.55.3.5	184	28	53%	46%	85%	88%
1IS8A_	d.96.1.1	187	1	100%	100%	100%	100%
1J7NA3	d.166.1.1	188	48	67%	12%	67%	67%
1JB7A2	b.40.4.3	196	2	30%	10%	59%	50%
1JJWA_	d.153.1.4	196	38	100%	68%	100%	100%
1HRHA1	c.55.3.1	200	5	80%	66%	73%	73%
1JSWA_	a.127.1.1	201	1	100%	100%	100%	100%
1JZ8A4	b.30.1.1	206	61	100%	100%	100%	100%
1K0IA1	c.3.1.2	208	12	23%	32%	24%	34%
1K94A_	a.39.1.7	215	1	100%	100%	100%	100%
1K9SA_	c.56.2.1	228	3	100%	72%	100%	100%
1KBVA2	b.6.1.3	232	3	82%	18%	98%	89%
1KID__	c.8.5.1	245	14	100%	100%	100%	100%
1KYGA_	c.47.1.10	252	3	74%	70%	98%	100%
1MTYD_	a.25.1.2	261	3	64%	17%	75%	92%
1KFWA1	c.1.8.5	267	5	87%	79%	99%	91%
1OELA1	a.129.1.1	290	14	100%	100%	100%	100%
1ONC__	d.5.1.1	297	4	100%	100%	100%	100%
1PBGA_	c.1.8.4	310	15	100%	100%	100%	100%
1PINA2	d.26.1.1	313	55	22%	30%	29%	34%
1QAXA2	d.179.1.1	319	2	100%	100%	100%	100%
1QDEA_	c.37.1.13	322	18	30%	23%	31%	49%
1QDLB_	c.23.16.1	346	13	100%	100%	100%	100%
1QE0A1	c.51.1.1	382	4	100%	93%	100%	100%
1QFJA2	c.25.1.1	415	22	75%	83%	60%	61%
1QGNA_	c.67.1.3	415	1	73%	70%	82%	81%
1QGWC_	a.1.1.3	422	2	100%	100%	100%	100%
1QKKA_	c.23.1.1	442	6	81%	99%	98%	91%
1QMGA2	c.2.1.6	477	19	12%	24%	79%	71%
1QOPB_	c.79.1.1	522	10	93%	93%	100%	100%
1QORA2	c.2.1.1	542	48	100%	100%	100%	100%
1QQ4A_	b.47.1.1	544	3	69%	63%	65%	67%
1SMVA_	b.10.1.2	565	11	100%	100%	98%	100%
1TRB_2	c.3.1.5	415	1	54%	61%	59%	62%
1VCAA2	b.1.1.4	422	2	9%	23%	45%	23%
1VDRA_	c.71.1.1	442	6	100%	100%	100%	100%
1GGWA_	a.39.1.5	477	19	75%	83%	67%	47%
1ZIN_1	c.37.1.1	522	10	62%	69%	70%	60%
2CMD_1	c.2.1.5	542	48	99%	99%	100%	83%
2SHPA1	c.45.1.2	544	3	100%	100%	100%	100%
1CQDA_	d.3.1.1	565	11	96%	98%	96%	97%
3GRX__	c.47.1.1	589	5	27%	49%	34%	35%
3PMGA1	c.84.1.1	589	5	34%	33%	63%	41%
