# Supplementary material for: Kappa-alpha plot derived structural alphabet and BLOSUM-like substitution matrix for rapid search of protein structure database
Source: Genome Biol. 2007 Mar 3;8(3):R31. doi: 10.1186/gb-2007-8-3-r31 (PMC1868941; doi:10.1186/gb-2007-8-3-r31)
Supplement: Additional data file 7 — Table showing the recognition performance of nine methods on the Lindahl's benchmark dataset at the family, superfamily, and fold levels. [file gb-2007-8-3-r31-S7.pdf]

Additional Data File 7: The recognition performance of nine methods on the Lindahl's benchmark dataset at the family, superfamily, and fold levels

% pairs ranked at top 1/top 5 Methods	Family (%)		Superfamily (%)		Fold (%)	
	Top 1	Top 5	Top 1	Top 5	Top 1	Top 5
BLAST2 [a]	65.9	70.1	18.7	29.7	5.6	13.1
SSEARCH	68.6	75.7	20.7	32.5	5.6	15.6
PSI-BLAST	71.2	72.3	27.4	27.9	4.0	4.7
HMMER-HSSP	68.8	74.4	16.1	26.3	5.3	11.8
SAM-HSSP	68.5	73.2	25.1	37.8	4.7	14.6
HMMER-PSIBLAST	67.7	73.5	20.7	31.3	4.4	14.6
SAM-PSIBLAST	70.1	74.4	28.3	38.9	3.4	18.7
BLAST-LINK	**74.6**	**78.9**	29.3	40.6	6.9	16.5
3D-BLAST	**68.1**	**78.4**	**39.2**	**54.8**	**19.0**	**39.3**

[a] The results of eight comparative methods are directly summarized from Lindahl and Elofsson [1]
