# Supplementary material for: Kappa-alpha plot derived structural alphabet and BLOSUM-like substitution matrix for rapid search of protein structure database
Source: Genome Biol. 2007 Mar 3;8(3):R31. doi: 10.1186/gb-2007-8-3-r31 (PMC1868941; doi:10.1186/gb-2007-8-3-r31)
Supplement: Additional data file 8 — Table listing 976 proteins of the Lindahl's benchmark. [file gb-2007-8-3-r31-S8.pdf]

Additional Data File 8: The list of 976 proteins of the Lindahl's benchmark

					Family		Superfamily		Fold		3D-BLAST Top 9								
	Query name	SCOP sccs	Length	members	Rank 1	Rank 5	Rank 1	Rank 5	Rank 1	Rank 5	1	2	3	4	5	6	7	8	9
1	119l-d119l	4_2_1_3	162	1	0	0	0	1	0	0	1jkw-d1jkw_1 (1_59_1_1)	1oxa-d1oxa (1_75_1_1)	1occc-d1occc1 (6_5_1_1)	1sly-d1sly_2 (4_2_1_5)	1bfra-d1bcfa (1_24_1_1)	1afra-d1afra (1_24_1_2)	1cpca-d1cpca (1_1_1_2)	2hbg-d1hbg (1_1_1_1)	1phe-d1phb (1_75_1_1)
2	1aab-d1aab	1_20_1_1	83	4	0	1	0	0	0	0	1orda-d1orda2 (3_48_1_4)	1occd-d1occd1 (6_5_1_1)	1aep-d1aep (1_49_1_1)	1lef-d1lefa (1_20_1_1)	1lis-d1lis (1_17_1_1)	1lbd-d1lbd (1_87_1_1)	1hme-d1hme (1_20_1_1)	1sly-d1sly_1 (1_84_4_1)	1lpe-d1lpe (1_23_1_1)
3	1aara-d1ubi	4_12_2_1	76	1	0	0	0	0	1	1	1guab-d1guab (4_12_3_1)	1otfa-d1otfa (4_40_1_1)	1aco-d1aco_1 (3_5_2_1)	1put-d1put (4_12_4_1)	1ltge-d1ltsd (2_26_2_1)	1poh-d1poh (4_52_1_1)	1ptf-d1ptf (4_52_1_1)	1ctt-d1ctt_2 (3_75_1_1)	1seta-d1seta2 (4_59_1_1)
4	1aay-d1zaac2	7_28_1_1	28	10	1	1	0	0	0	0	1ard-d1ard (7_28_1_1)	1aay-d1zaac3 (7_28_1_1)	1znf-d1znf (7_28_1_1)	1zaac-d1zaac1 (7_28_1_1)	1paa-d1paa (7_28_1_1)	1tgsi-d1tgsi (7_12_1_1)	1tdta-d1tdta (2_57_1_2)	1nal1-d1nal1 (3_1_3_1)	1tfi-d1tfi (7_32_3_1)
5	1aay-d1zaac3	7_28_1_1	28	10	1	1	0	0	0	0	1ard-d1ard (7_28_1_1)	1aay-d1zaac2 (7_28_1_1)	1paa-d1paa (7_28_1_1)	1faga-d2bmha (1_75_1_1)	1mmob-d1mmob (1_24_1_2)	1znf-d1znf (7_28_1_1)	1puee-d1puee (1_4_3_9)	2anhb-d2anha (3_56_1_1)	1tns-d1tns (1_6_1_1)
6	1ab3-d1ab3	1_48_1_1	88	1	0	0	0	0	0	0	1mtyd-d1mmod (1_24_1_2)	1fps-d1fps (1_91_1_1)	2ccya-d2ccya (1_23_3_2)	1cbg-d1cbg (3_1_1_4)	1csr-d1csh (1_74_1_1)	1broa-d1broa (3_50_1_5)	1ad2-d1ad2 (5_20_1_1)	1bgl-d1bgla5 (3_1_1_3)	1itha-d1itha (1_1_1_1)
7	1aba-d1aba	3_33_1_1	87	4	0	0	0	0	0	0	3pmga-d3pmga1 (3_64_1_1)	1geua-d1gesa2 (3_4_1_4)	1hdca-d1hdca (3_19_1_2)	1tbrr-d1tbrr2 (7_12_1_1)	1mioa-d1mioa (3_67_1_1)	1igs-d1igs (3_1_8_1)	1bmta-d1bmta2 (3_13_5_1)	1egr-d1ego (3_33_1_1)	4xis-d2xis (3_1_12_1)
8	1abra-d1abra	4_94_1_1	251	3	1	1	0	0	0	0	1mrj-d1mrj (4_94_1_1)	1apa-d1apa (4_94_1_1)	1faga-d2bmha (1_75_1_1)	1dik-d1dik_1 (3_1_9_2)	1vin-d1vin_1 (1_59_1_1)	1gcb-d1gcb (4_3_1_1)	1cyda-d1cyda (3_19_1_2)	1gtma-d1gtma1 (3_19_1_7)	1csr-d1csh (1_74_1_1)
9	1abrb-d1abrb1	2_28_2_1	140	2	1	1	0	0	1	1	1abrb-d1abrb2 (2_28_2_1)	1iob-d1i1b (2_28_1_2)	1mrj-d1mrj (4_94_1_1)	2bbkh-d2bbkh (2_46_2_1)	1gof-d1gof_3 (2_46_1_1)	1php-d1php (3_66_1_1)	2bfh-d2fgf (2_28_1_1)	1pea-d1pea (3_72_1_1)	2sil-d2sil (2_45_1_1)
10	1abrb-d1abrb2	2_28_2_1	127	2	1	1	0	0	1	1	1abrb-d1abrb1 (2_28_2_1)	2bfh-d2fgf (2_28_1_1)	2trcb-d2trcb (2_46_3_1)	1eur-d1eur (2_45_1_1)	4sgbi-d4sgbi (7_12_1_2)	1pea-d1pea (3_72_1_1)	1scb-d1csee (3_28_1_1)	1msad-d1msaa (2_54_1_1)	1iob-d1i1b (2_28_1_2)
11	1aca-d1aca	1_11_1_1	86	1	0	0	0	0	0	0	1occa-d1occa1 (6_5_1_1)	1dik-d1dik_3 (4_83_1_4)	1occe-d1occe (1_84_7_1)	1gtma-d1gtma1 (3_19_1_7)	1occc-d1occc1 (6_5_1_1)	1pgo-d2pgd_1 (1_71_1_1)	1jkw-d1jkw_1 (1_59_1_1)	2dhd-d1ede (3_50_1_3)	1csr-d1csh (1_74_1_1)
12	1acf-d1acf	4_61_1_1	125	2	1	1	0	0	0	0	1pne-d1pne (4_61_1_1)	2dkb-d2dkb (3_48_1_3)	1ospo-d1ospo (2_52_1_1)	1gym-d1gym (3_1_15_2)	1cof-d1ahq (4_60_1_2)	1eri-d1eria (3_38_1_1)	2tgi-d1tfg (7_14_1_2)	1tcma-d1cdg_3 (2_48_1_1)	1hpm-d1hpm_1 (3_41_1_1)
13	1aco-d1aco_1	3_5_2_1	226	1	0	0	0	0	0	1	1ecpa-d1ecpa (3_52_1_1)	2lbp-d2lbp (3_72_1_1)	1zymb-d1zyma (3_5_1_2)	1orb-d1orb_2 (3_60_1_1)	1eft-d1eft_3 (3_25_1_3)	1rnl-d1rnl_2 (3_13_2_1)	2hnp-d2hnp (3_32_1_2)	1sfta-d1sfta2 (3_1_18_1)	1edt-d1edt (3_1_1_5)
14	1acp-d1acp	1_26_1_1	77	1	0	0	0	0	0	0	1nis-d1aco_2 (3_63_1_1)	6ldh-d1ldm_2 (4_92_1_1)	2abk-d2abk (1_66_1_1)	1llda-d1llda2 (4_92_1_1)	1orta-d1orta1 (3_58_1_1)	1geua-d1gesa2 (3_4_1_4)	2csn-d1csn (5_1_1_1)	1wdcb-d1scmb (1_34_1_5)	1miob-d1miob (3_67_1_1)
15	1acw-d1acw	7_3_6_2	29	4	0	0	0	0	0	0	1seia-d1seia (4_80_1_1)	1chka-d1chka (4_2_1_6)	1kit-d1kit_2 (2_19_1_6)	1ospo-d1ospo (2_52_1_1)	1ptf-d1ptf (4_52_1_1)	1aara-d1ubi (4_12_2_1)	2blta-d2blta (5_4_1_1)	1ctn-d1ctn_3 (4_19_3_1)	1bll-d1lam_2 (3_52_3_3)
16	1ad2-d1ad2	5_20_1_1	224	1	0	0	0	0	0	0	1orda-d1orda3 (4_70_1_1)	1mtyd-d1mmod (1_24_1_2)	1hpm-d1hpm_1 (3_41_1_1)	1mpd-d1mpb (3_73_1_1)	2ebn-d2ebn (3_1_1_5)	1dhpa-d1dhpa (3_1_3_1)	1iov-d2dln_2 (4_83_1_1)	1broa-d1broa (3_50_1_5)	1xyza-d1xyza (3_1_1_3)
17	1adn-d1adn	7_37_1_1	92	1	0	0	0	0	0	0	1pgs-d1pgs_1 (2_11_1_1)	1gof-d1gof_3 (2_46_1_1)	1wdcb-d1scmb (1_34_1_5)	1fcdb-d1fcda2 (3_4_1_4)	1tpn-d1tpm (7_40_1_1)	3rubs-d3rubs (4_37_1_1)	2aaa-d2aaa_1 (2_48_1_1)	1gal-d1gal_1 (3_4_1_2)	1kit-d1kit_2 (2_19_1_6)
18	1adr-d1adr	1_30_1_2	76	5	1	1	0	0	0	0	1llib-d1llia (1_30_1_2)	1perl-d1r69 (1_30_1_2)	1lis-d1lis (1_17_1_1)	1rnl-d1rnl_1 (1_31_1_2)	2abk-d2abk (1_66_1_1)	1hcr-d1hcra (1_4_1_2)	1ezm-d1ezm_1 (1_53_1_1)	1bdi-d1pnra1 (1_30_1_3)	1onea-d1ebha2 (4_31_1_1)
19	1adt-d1adt_1	1_45_1_1	90	1	0	0	0	0	0	0	1pgo-d2pgd_1 (1_71_1_1)	1sly-d1sly_1 (1_84_4_1)	1nfp-d1nfp (3_1_13_2)	1occc-d1occc1 (6_5_1_1)	1glm-d1glm (1_73_1_1)	1gtma-d1gtma1 (3_19_1_7)	1vin-d1vin_1 (1_59_1_1)	2myd-d1mbd (1_1_1_1)	2dri-d2dri (3_72_1_1)
20	1aep-d1aep	1_49_1_1	153	1	0	0	0	0	0	0	1mrrb-d1riba (1_24_1_2)	1lpe-d1lpe (1_23_1_1)	1ciy-d1ciy_3 (6_1_3_1)	1xsm-d1xsm (1_24_1_2)	1mtyd-d1mmod (1_24_1_2)	1ryt-d1ryt_1 (1_24_1_1)	1bfra-d1bcfa (1_24_1_1)	1occa-d1occa1 (6_5_1_1)	1rcd-d1rcd (1_24_1_1)
21	1aey-d1shg	2_21_2_1	57	4	1	1	0	0	1	1	1sema-d1sema (2_21_2_1)	1pht-d1pht (2_21_2_1)	1hsq-d1hsq (2_21_2_1)	1ihva-d1ihwa (2_21_6_1)	1sty-d1sty (2_26_1_1)	1hcd-d1hcd (2_28_4_1)	1wba-d1wba (2_28_3_1)	1efrd-d1bmfd2 (2_33_1_1)	1dar-d1dar_1 (2_29_3_1)
22	1afp-d1afp	7_23_1_1	51	1	0	0	0	0	0	0	4aahc-d4aaha (2_47_1_1)	2trcb-d2trcb (2_46_3_1)	1gof-d1gof_3 (2_46_1_1)	2bfh-d2fgf (2_28_1_1)	1pex-d1pex (2_44_1_1)	1gen-d1gen (2_44_1_1)	1abra-d1abra (4_94_1_1)	2prd-d2prd (2_26_5_1)	2bbkh-d2bbkh (2_46_2_1)
23	1afra-d1afra	1_24_1_2	345	5	1	1	0	1	0	0	1mrrb-d1riba (1_24_1_2)	1occc-d1occc1 (6_5_1_1)	1bfra-d1bcfa (1_24_1_1)	1sly-d1sly_1 (1_84_4_1)	1xsm-d1xsm (1_24_1_2)	1csr-d1csh (1_74_1_1)	1rcd-d1rcd (1_24_1_1)	1occa-d1occa1 (6_5_1_1)	1fps-d1fps (1_91_1_1)
24	1agre-d1agre	1_64_1_1	128	1	0	0	0	0	0	0	1prcl-d1prcl1 (6_5_1_1)	1occc-d1occc1 (6_5_1_1)	1mrrb-d1riba (1_24_1_2)	1mmob-d1mmob (1_24_1_2)	1zymb-d1zyma (3_5_1_2)	1aep-d1aep (1_49_1_1)	2sas-d2sas (1_34_1_5)	2hbg-d1hbg (1_1_1_1)	1glqa-d1glqa1 (1_38_1_1)
25	1ahia-d1fmca	3_19_1_2	255	6	1	1	0	0	0	0	1hdca-d1hdca (3_19_1_2)	1cyda-d1cyda (3_19_1_2)	1eny-d1eny (3_19_1_2)	1dhr-d1dhr (3_19_1_2)	1nai-d1xel (3_19_1_2)	2lbp-d2lbp (3_72_1_1)	1rvva-d1rvva (3_9_1_1)	2dri-d2dri (3_72_1_1)	1gca-d1gca (3_72_1_1)
26	1ahl-d1ahl	7_7_1_1	49	5	0	0	0	0	0	0	2trcb-d2trcb (2_46_3_1)	1poxa-d1poxa2 (3_24_1_1)	1tuia-d1eft_1 (2_29_3_1)	1fjm-d1fjma (4_91_1_2)	1vhh-d1vhh (4_34_1_2)	1obr-d1obr (3_52_3_2)	1kuh-d1kuh (4_50_1_1)	1gph1-d1gph12 (4_88_1_1)	2sh1-d1sh1 (7_7_1_1)
27	1ahsa-d1ahsa	2_14_1_1	126	2	1	1	0	0	0	0	1bvp1-d1bvp12 (2_14_1_1)	1kit-d1kit_1 (2_19_1_6)	1kcw-d1kcw_1 (2_5_1_3)	2hmb-d1hms (2_41_1_2)	1eal-d1eal (2_41_1_2)	1con-d1scs (2_19_1_1)	1cpn-d1cpn (2_19_1_2)	1p03a-d2alp (2_31_1_1)	1knb-d1knb (2_16_1_1)
28	1akl-d1kapp1	2_55_1_1	224	1	0	0	0	0	0	0	1ospo-d1ospo (2_52_1_1)	2trcb-d2trcb (2_46_3_1)	1eal-d1eal (2_41_1_2)	1sacb-d1saca (2_19_1_4)	1sva5-d1sva1 (2_8_1_4)	1mpmb-d1mal (6_7_1_2)	4aahc-d4aaha (2_47_1_1)	3nn9-d3nn9 (2_45_1_1)	1ulo-d1ulo (2_13_1_4)
29	1akl-d1kapp2	4_50_1_6	239	1	0	0	1	1	0	0	2tcl-d1hfc (4_50_1_7)	2csn-d1csn (5_1_1_1)	1mbt-d1mbb_2 (4_85_1_1)	1iad-d1ast (4_50_1_4)	1htda-d1atla (4_50_1_5)	1jeva-d2olba (3_73_1_1)	1phk-d1phk (5_1_1_1)	1tlfa-d1tlfa (3_72_1_1)	1prcc-d1prcc (1_77_1_1)
30	1alla-d1alla	1_1_1_2	160	3	1	1	1	1	0	0	1cpcb-d1cpcb (1_1_1_2)	1cpca-d1cpca (1_1_1_2)	1mba-d1mba (1_1_1_1)	1occc-d1occc1 (6_5_1_1)	1lh7-d1lh1 (1_1_1_1)	2myd-d1mbd (1_1_1_1)	1itha-d1itha (1_1_1_1)	1hlb-d1hlb (1_1_1_1)	1lpe-d1lpe (1_23_1_1)
31	1alo-d1alo_1	1_47_1_1	113	1	0	0	0	0	0	0	1lbd-d1lbd (1_87_1_1)	1jkw-d1jkw_1 (1_59_1_1)	1octc-d1octc1 (1_4_1_1)	1dik-d1dik_1 (3_1_9_2)	1vhra-d1vhra (3_32_1_1)	1mtyd-d1mmod (1_24_1_2)	1gtra-d1gtra2 (3_15_1_1)	1tcob-d1tcob (1_34_1_5)	1vin-d1vin_1 (1_59_1_1)
32	1alo-d1alo_3	4_23_1_1	117	1	0	0	0	0	0	0	1asya-d1asya1 (2_26_4_1)	1pii-d1pii_2 (3_1_8_1)	1csr-d1csh (1_74_1_1)	1gpr-d1gpr (2_59_3_1)	1lis-d1lis (1_17_1_1)	1qba-d1qba_3 (3_1_1_6)	1dxy-d1dxy_2 (3_19_1_4)	1cdaa-d1aly (2_17_1_1)	1alo-d1alo_5 (4_77_1_1)
33	1alo-d1alo_4	4_77_1_1	132	4	0	1	0	0	0	0	1fead-d2tpra3 (4_46_1_1)	1lvl-d1lvl_3 (4_46_1_1)	2npx-d1nhp_3 (4_46_1_1)	1hrda-d1hrda2 (3_54_1_1)	1alo-d1alo_6 (4_77_1_1)	3pte-d3pte (5_4_1_1)	1juy-d1adea (3_25_1_5)	1iov-d2dln_2 (4_83_1_1)	1pce-d1pce (7_12_1_1)
34	1alo-d1alo_5	4_77_1_1	115	4	0	0	0	0	0	0	1bll-d1lam_2 (3_52_3_3)	1fem-d1hbp (2_41_1_1)	1coy-d1coy_1 (3_4_1_2)	1gtma-d1gtma2 (3_54_1_1)	1oiba-d1pbp (3_73_1_1)	1gky-d1gky (3_25_1_1)	1seta-d1seta2 (4_59_1_1)	1gnd-d1gnd_2 (4_13_1_5)	1udii-d1udii (4_14_4_1)
35	1alo-d1alo_6	4_77_1_1	178	4	0	0	0	0	0	0	1tkaa-d1trka1 (3_24_1_2)	1pda-d1pda_2 (4_28_2_1)	1pgo-d2pgd_1 (1_71_1_1)	2tmda-d2tmda1 (3_1_7_1)	2liga-d2liga (1_23_2_1)	1octc-d1octc2 (1_30_1_1)	1gesa-d1gesa3 (4_46_1_1)	1cem-d1cem (1_73_1_2)	1mtyd-d1mmod (1_24_1_2)
36	1alo-d1alo_7	4_77_1_1	172	4	0	0	0	0	0	0	2hnp-d2hnp (3_32_1_2)	2npx-d1nhp_3 (4_46_1_1)	1lxta-d3pmga4 (4_74_2_1)	1dkza-d1dkza (5_17_1_1)	2omf-d2omf (6_7_1_1)	1cof-d1ahq (4_60_1_2)	1leha-d1leha2 (3_54_1_1)	1asu-d1asu (3_41_3_2)	1asza-d1asya2 (4_59_1_1)
37	1amb-d1amb	8_38_1_1	28	1	0	0	0	0	0	0	1occc-d1occc1 (6_5_1_1)	2sas-d2sas (1_34_1_5)	1prhb-d1prha1 (1_65_1_2)	1xsm-d1xsm (1_24_1_2)	1gsea-d1gsea1 (1_38_1_1)	1knya-d1knya (5_10_1_2)	7cat-d7cata (5_6_1_1)	1vola-d1vola1 (1_59_1_2)	1fps-d1fps (1_91_1_1)
38	1amp-d1amp	3_52_3_4	291	1	0	0	1	1	0	0	1bll-d1lam_2 (3_52_3_3)	1nai-d1xel (3_19_1_2)	1nis-d1aco_2 (3_63_1_1)	2dri-d2dri (3_72_1_1)	1opr-d1opr (3_44_1_1)	1hora-d1deaa (3_23_1_1)	2lbp-d2lbp (3_72_1_1)	1gtma-d1gtma2 (3_54_1_1)	2aaa-d2aaa_2 (3_1_1_1)
39	1amy-d1amy_1	2_48_1_1	57	6	0	1	0	0	0	0	1acf-d1acf (4_61_1_1)	1dar-d1dar_1 (2_29_3_1)	1tcma-d1cdg_3 (2_48_1_1)	1qila-d1tssa1 (2_26_2_2)	1mpmb-d1mal (6_7_1_2)	1cur-d1cur (2_5_1_1)	1gen-d1gen (2_44_1_1)	1bvp1-d1bvp12 (2_14_1_1)	1orda-d1orda2 (3_48_1_4)
40	1amy-d1amy_2	3_1_1_1	346	4	1	1	0	1	0	1	2aaa-d2aaa_2 (3_1_1_1)	1cxh-d1cdg_4 (3_1_1_1)	1ppi-d1ppi_2 (3_1_1_1)	1gtra-d1gtra2 (3_15_1_1)	1bmta-d1bmta2 (3_13_5_1)	1eceb-d1ecea (3_1_1_3)	1nal1-d1nal1 (3_1_3_1)	2lbp-d2lbp (3_72_1_1)	2ebn-d2ebn (3_1_1_5)
41	1aocb-d1aoca	7_14_1_5	175	1	0	0	1	1	0	0	1btga-d1bet (7_14_1_3)	1pfsa-d1pfsa (2_26_4_7)	1cdaa-d1aly (2_17_1_1)	1gpc-d1gpc (2_26_4_7)	1ahsa-d1ahsa (2_14_1_1)	1eur-d1eur (2_45_1_1)	4xis-d2xis (3_1_12_1)	2bbkh-d2bbkh (2_46_2_1)	1nsdb-d1nsca (2_45_1_1)
42	1apa-d1apa	4_94_1_1	261	3	1	1	0	0	0	0	1abra-d1abra (4_94_1_1)	1mrj-d1mrj (4_94_1_1)	1dik-d1dik_1 (3_1_9_2)	2hnp-d2hnp (3_32_1_2)	1chd-d1chd (3_27_1_1)	1oxa-d1oxa (1_75_1_1)	1occa-d1occa1 (6_5_1_1)	1eft-d1eft_3 (3_25_1_3)	1prch-d1prch1 (2_27_1_1)
43	1apme-d1apme	5_1_1_1	341	4	1	1	0	0	0	0	1phk-d1phk (5_1_1_1)	1kob-d1koba (5_1_1_1)	2csn-d1csn (5_1_1_1)	7icd-d7icd (3_57_1_1)	3tgl-d3tgl (3_50_1_7)	1lyla-d1lyla2 (4_59_1_1)	1edt-d1edt (3_1_1_5)	1iov-d2dln_2 (4_83_1_1)	2lbp-d2lbp (3_72_1_1)
44	1aps-d1aps	4_33_10_1	98	1	0	0	0	0	0	0	1juy-d1adea (3_25_1_5)	1aba-d1aba (3_33_1_1)	1kob-d1koba (5_1_1_1)	1hmp-d1hmpa (3_44_1_1)	1scub-d1scub2 (4_83_1_3)	1geua-d1gesa2 (3_4_1_4)	1bya-d1byb (3_1_1_2)	1php-d1php (3_66_1_1)	1copd-d1copd (1_30_1_2)

45	1arc-d1arb	2_31_1_1	263	3	1	1	1	1	0	0	1p03a-d2alp (2_31_1_1)	1tnj-d1bty (2_31_1_2)	5sgae-d1sgc (2_31_1_1)	1ospo-d1ospo (2_52_1_1)	1gof-d1gof_3 (2_46_1_1)	2cas-d2cas (2_8_1_4)	4aahc-d4aaha (2_47_1_1)	1kxa-d2snv (2_31_1_3)	1wba-d1wba (2_28_3_1)
46	1ard-d1ard	7_28_1_1	29	10	1	1	0	0	0	0	1aay-d1zaac2 (7_28_1_1)	1aay-d1zaac3 (7_28_1_1)	1paa-d1paa (7_28_1_1)	1znf-d1znf (7_28_1_1)	1zaac-d1zaac1 (7_28_1_1)	2lbp-d2lbp (3_72_1_1)	1mmob-d1mmob (1_24_1_2)	1oya-d1oya (3_1_7_1)	1orda-d1orda2 (3_48_1_4)
47	1art-d1art	3_48_1_1	396	1	0	0	1	1	0	0	2dkb-d2dkb (3_48_1_3)	1nal1-d1nal1 (3_1_3_1)	2dri-d2dri (3_72_1_1)	1orda-d1orda2 (3_48_1_4)	2gpb-d1gpb (3_68_1_2)	1wsyb-d1wsyb (3_59_1_1)	1igs-d1igs (3_1_8_1)	1miob-d1miob (3_67_1_1)	1tlfa-d1tlfa (3_72_1_1)
48	1asob-d1aoza3	2_5_1_3	214	8	1	1	0	1	0	0	1kcw-d1kcw_4 (2_5_1_3)	1kcw-d1kcw_2 (2_5_1_3)	1kcw-d1kcw_6 (2_5_1_3)	1asqa-d1aoza2 (2_5_1_3)	1aspb-d1aoza1 (2_5_1_3)	4aahc-d4aaha (2_47_1_1)	1kcw-d1kcw_5 (2_5_1_3)	1ciy-d1ciy_2 (2_53_2_1)	1kcw-d1kcw_1 (2_5_1_3)
49	1aspb-d1aoza1	2_5_1_3	129	8	1	1	1	1	0	0	1kcw-d1kcw_1 (2_5_1_3)	1kcw-d1kcw_6 (2_5_1_3)	1kcw-d1kcw_4 (2_5_1_3)	1kcw-d1kcw_2 (2_5_1_3)	1asob-d1aoza3 (2_5_1_3)	1cur-d1cur (2_5_1_1)	1kcw-d1kcw_5 (2_5_1_3)	1asqa-d1aoza2 (2_5_1_3)	2cbp-d2cbp (2_5_1_1)
50	1asqa-d1aoza2	2_5_1_3	209	8	1	1	0	1	0	0	1kcw-d1kcw_6 (2_5_1_3)	1asob-d1aoza3 (2_5_1_3)	1kcw-d1kcw_4 (2_5_1_3)	1kcw-d1kcw_1 (2_5_1_3)	1aspb-d1aoza1 (2_5_1_3)	1kcw-d1kcw_5 (2_5_1_3)	1vcaa-d1vcaa1 (2_1_1_3)	1bbpa-d1bbpa (2_41_1_1)	1cur-d1cur (2_5_1_1)
51	1asu-d1asu	3_41_3_2	162	1	0	0	0	0	0	0	1rvva-d1rvva (3_9_1_1)	2dri-d2dri (3_72_1_1)	7icd-d7icd (3_57_1_1)	1art-d1art (3_48_1_1)	1dora-d1dora (3_1_7_1)	1dkza-d1dkza (5_17_1_1)	1crr-d5p21 (3_25_1_3)	2sas-d2sas (1_34_1_5)	1dik-d1dik_1 (3_1_9_2)
52	1asya-d1asya1	2_26_4_1	137	2	1	1	0	0	0	1	1lylb-d1lyla1 (2_26_4_1)	1shca-d1shca (2_37_1_2)	1obpa-d1obpa (2_41_1_1)	1eft-d1eft_2 (2_30_1_1)	1aocb-d1aoca (7_14_1_5)	1tiig-d1tiid (2_26_2_1)	1phk-d1phk (5_1_1_1)	1mai-d1mai (2_37_1_1)	1sty-d1sty (2_26_1_1)
53	1asza-d1asya2	4_59_1_1	353	3	1	1	0	0	0	0	1lyla-d1lyla2 (4_59_1_1)	1rpl-d1rpl (5_10_1_1)	1daaa-d1daaa (5_14_1_1)	2lbp-d2lbp (3_72_1_1)	1msk-d1msk (4_99_1_1)	1fbaa-d1fbaa (3_1_3_1)	1seta-d1seta2 (4_59_1_1)	1mbt-d1mbb_2 (4_85_1_1)	1oiba-d1pbp (3_73_1_1)
54	1ata-d1ata	7_21_1_1	62	1	0	0	0	0	0	0	2ebn-d2ebn (3_1_1_5)	2aaa-d2aaa_1 (2_48_1_1)	1cpn-d1cpn (2_19_1_2)	1bgl-d1bgla5 (3_1_1_3)	1rie-d1rie (7_33_1_2)	1tcmb-d1cdg_1 (2_1_1_5)	1p03a-d2alp (2_31_1_1)	1mpmb-d1mal (6_7_1_2)	1scb-d1csee (3_28_1_1)
55	1azca-d1aiza	2_5_1_1	129	4	0	0	0	1	0	0	2ncm-d2ncm (2_1_1_4)	1svb-d1svb_2 (6_10_1_1)	1kcw-d1kcw_1 (2_5_1_3)	1kcw-d1kcw_5 (2_5_1_3)	1rldb-d3rubl2 (4_33_9_1)	1chd-d1chd (3_27_1_1)	1aspb-d1aoza1 (2_5_1_3)	1whi-d1whi (2_25_1_1)	1thw-d1thw (2_18_1_1)
56	1baba-d1baba	1_1_1_1	141	10	1	1	1	1	0	0	2myd-d1mbd (1_1_1_1)	2hbg-d1hbg (1_1_1_1)	2lhb-d2lhb (1_1_1_1)	1lh7-d1lh1 (1_1_1_1)	1mba-d1mba (1_1_1_1)	1flp-d1flp (1_1_1_1)	1hlb-d1hlb (1_1_1_1)	1itha-d1itha (1_1_1_1)	1cpca-d1cpca (1_1_1_2)
57	1bba-d1bba	8_5_1_1	36	2	0	0	0	0	0	0	1fnc-d1fnb_2 (3_14_1_1)	1daaa-d1daaa (5_14_1_1)	1reqb-d1reqb2 (3_13_5_2)	1leha-d1leha2 (3_54_1_1)	2dri-d2dri (3_72_1_1)	1chma-d1chma2 (4_72_1_1)	1cem-d1cem (1_73_1_2)	3fx2-d2fx2 (3_13_4_1)	1faga-d2bmha (1_75_1_1)
58	1bbha-d1bbha	1_23_3_2	131	3	1	1	0	0	0	0	1rcpa-d1cpq (1_23_3_2)	2ccya-d2ccya (1_23_3_2)	1ciy-d1ciy_3 (6_1_3_1)	1occc-d1occc1 (6_5_1_1)	1dlc-d1dlc_3 (6_1_3_1)	1bfra-d1bcfa (1_24_1_1)	1aep-d1aep (1_49_1_1)	1cpcb-d1cpcb (1_1_1_2)	1ryt-d1ryt_1 (1_24_1_1)
59	1bbl-d1bbl	1_9_1_1	37	1	0	0	0	0	0	0	1cpca-d1cpca (1_1_1_2)	1orb-d1orb_2 (3_60_1_1)	3pmga-d3pmga1 (3_64_1_1)	1fbaa-d1fbaa (3_1_3_1)	1poxa-d1poxa3 (3_24_1_1)	1vhh-d1vhh (4_34_1_2)	1gdha-d1gdha2 (3_19_1_4)	1xyza-d1xyza (3_1_1_3)	1brnl-d1brnl (4_1_1_1)
60	1bbo-d1bbo_2	7_28_1_1	28	10	0	0	0	0	0	0	1plq-d1plq_1 (4_76_1_2)	1poxa-d1poxa3 (3_24_1_1)	1jdya-d3pmga3 (3_64_1_1)	1gsea-d1gsea2 (3_33_1_5)	1urna-d1urna (4_33_7_1)	1inp-d1inp (5_8_1_3)	1etpa-d1etpa2 (1_3_1_3)	1apa-d1apa (4_94_1_1)	5znf-d5znf (7_28_1_1)
61	1bbpa-d1bbpa	2_41_1_1	173	4	1	1	1	1	0	0	1fem-d1hbp (2_41_1_1)	1obpa-d1obpa (2_41_1_1)	2hmb-d1hms (2_41_1_2)	1bebb-d1beba (2_41_1_1)	1eal-d1eal (2_41_1_2)	1sacb-d1saca (2_19_1_4)	1ure-d1ifc (2_41_1_2)	1dlc-d1dlc_2 (2_53_2_1)	2por-d2por (6_7_1_1)
62	1bco-d1bco_1	2_32_1_1	80	1	0	0	0	0	0	0	1bebb-d1beba (2_41_1_1)	1ospo-d1ospo (2_52_1_1)	2bbkh-d2bbkh (2_46_2_1)	1p03a-d2alp (2_31_1_1)	1juy-d1adea (3_25_1_5)	1noy-d1noya (3_41_3_5)	1asqa-d1aoza2 (2_5_1_3)	1smpi-d1smpi (2_42_2_1)	3rnt-d9rnt (4_1_1_1)
63	1bcpb-d1prtb1	2_26_2_1	112	6	0	1	0	0	0	0	1hjra-d1hjra (3_41_3_6)	1coy-d1coy_1 (3_4_1_2)	2hnp-d2hnp (3_32_1_2)	1prtf-d1prtf (2_26_2_1)	1prtd-d1prtd (2_26_2_1)	1bove-d1bova (2_26_2_1)	1nhq-d1nhp_2 (3_4_1_4)	1eal-d1eal (2_41_1_2)	1ltge-d1ltsd (2_26_2_1)
64	1bct-d1bct	8_30_1_1	69	2	0	0	0	0	0	0	1lpe-d1lpe (1_23_1_1)	1sesa-d1seta1 (1_2_3_1)	1rcd-d1rcd (1_24_1_1)	1lki-d1lki (1_25_1_1)	1vsga-d1vsga (6_3_1_1)	1bfra-d1bcfa (1_24_1_1)	1ryt-d1ryt_1 (1_24_1_1)	1mmob-d1mmob (1_24_1_2)	1aep-d1aep (1_49_1_1)
65	1bdi-d1pnra1	1_30_1_3	56	2	0	1	1	1	0	0	1llib-d1llia (1_30_1_2)	1lcc-d1lcca (1_30_1_3)	2ada-d1add (3_1_2_1)	1vhra-d1vhra (3_32_1_1)	1adr-d1adr (1_30_1_2)	2utga-d1utg (1_72_1_1)	1csr-d1csh (1_74_1_1)	1coy-d1coy_1 (3_4_1_2)	1vin-d1vin_1 (1_59_1_1)
66	1bdmb-d1bdma2	4_92_1_1	172	5	1	1	0	0	0	0	1llda-d1llda2 (4_92_1_1)	6ldh-d1ldm_2 (4_92_1_1)	1emd-d2cmd_2 (4_92_1_1)	1hlpa-d1hlpa2 (4_92_1_1)	1esd-d1esc (3_13_8_1)	1gbs-d1gbs (4_2_1_4)	1nfp-d1nfp (3_1_13_2)	1ytn-d1ypta (3_32_1_2)	2csn-d1csn (5_1_1_1)
67	1bdo-d1bdo	2_59_1_1	80	5	1	1	0	0	1	1	1iyu-d1iyu (2_59_1_1)	1ghj-d1ghk (2_59_1_1)	1lac-d1lac (2_59_1_1)	1hpca-d1htp (2_59_1_1)	1gpr-d1gpr (2_59_3_1)	1dar-d1dar_1 (2_29_3_1)	1tdta-d1tdta (2_57_1_2)	1p03a-d2alp (2_31_1_1)	1akl-d1kapp1 (2_55_1_1)
68	1bds-d1bds	7_7_1_1	43	5	0	0	0	0	0	0	1gpma-d1gpma3 (4_30_1_1)	1ghr-d1ghr (3_1_1_3)	5sgae-d1sgc (2_31_1_1)	1mrj-d1mrj (4_94_1_1)	1ata-d1ata (7_21_1_1)	1gen-d1gen (2_44_1_1)	1rie-d1rie (7_33_1_2)	1chd-d1chd (3_27_1_1)	1obr-d1obr (3_52_3_2)
69	1bebb-d1beba	2_41_1_1	156	4	1	1	0	1	0	0	1obpa-d1obpa (2_41_1_1)	1fem-d1hbp (2_41_1_1)	1bbpa-d1bbpa (2_41_1_1)	1ospo-d1ospo (2_52_1_1)	1cyx-d1cyx (2_5_1_2)	1iob-d1i1b (2_28_1_2)	2sil-d2sil (2_45_1_1)	1eal-d1eal (2_41_1_2)	1wit-d1wiu (2_1_1_4)
70	1beo-d1beo	1_96_1_1	98	1	0	0	0	0	0	0	1paw-d1pax_1 (1_35_1_1)	1hrda-d1hrda1 (3_19_1_7)	1lis-d1lis (1_17_1_1)	1fbaa-d1fbaa (3_1_3_1)	2sas-d2sas (1_34_1_5)	1afra-d1afra (1_24_1_2)	1mba-d1mba (1_1_1_1)	1buca-d1buca1 (1_23_6_1)	1mrj-d1mrj (4_94_1_1)
71	1bfma-d1bfma	1_21_1_2	69	1	0	0	0	0	0	0	1occc-d1occc1 (6_5_1_1)	1hrda-d1hrda1 (3_19_1_7)	1sesa-d1seta1 (1_2_3_1)	2liga-d2liga (1_23_2_1)	1xsm-d1xsm (1_24_1_2)	1lis-d1lis (1_17_1_1)	1lbd-d1lbd (1_87_1_1)	1juy-d1adea (3_25_1_5)	1cpca-d1cpca (1_1_1_2)
72	1bfra-d1bcfa	1_24_1_1	158	3	1	1	0	1	0	0	1rcd-d1rcd (1_24_1_1)	1ryt-d1ryt_1 (1_24_1_1)	1occc-d1occc1 (6_5_1_1)	1afra-d1afra (1_24_1_2)	1mrrb-d1riba (1_24_1_2)	2liga-d2liga (1_23_2_1)	1ciy-d1ciy_3 (6_1_3_1)	1lpe-d1lpe (1_23_1_1)	1aep-d1aep (1_49_1_1)
73	1bgk-d1bgk	7_16_1_1	37	2	0	0	0	0	0	0	2sas-d2sas (1_34_1_5)	1ad2-d1ad2 (5_20_1_1)	1gsea-d1gsea2 (3_33_1_5)	2btf-d2btfa2 (3_41_1_1)	1igs-d1igs (3_1_8_1)	6lyt-d193l (4_2_1_2)	2bbvc-d2bbva (2_8_1_3)	1put-d1put (4_12_4_1)	1vtm-d1vtmp (1_23_5_1)
74	1bgl-d1bgla1	2_1_3_1	114	2	0	1	0	0	0	1	1ospo-d1ospo (2_52_1_1)	2por-d2por (6_7_1_1)	1dlc-d1dlc_1 (2_13_1_2)	1bgl-d1bgla2 (2_1_3_1)	1cdcb-d1cdcb (2_1_1_1)	1vcaa-d1vcaa1 (2_1_1_3)	1prn-d1prn (6_7_1_1)	1fem-d1hbp (2_41_1_1)	1nsdb-d1nsca (2_45_1_1)
75	1bgl-d1bgla2	2_1_3_1	105	2	1	1	0	0	0	1	1bgl-d1bgla1 (2_1_3_1)	2hmb-d1hms (2_41_1_2)	1dlc-d1dlc_1 (2_13_1_2)	3nn9-d3nn9 (2_45_1_1)	1bbpa-d1bbpa (2_41_1_1)	1vcaa-d1vcaa1 (2_1_1_3)	1nsdb-d1nsca (2_45_1_1)	2por-d2por (6_7_1_1)	2bpa1-d2bpa1 (2_8_1_1)
76	1bgl-d1bgla3	2_13_1_3	217	1	0	0	0	1	0	0	1lcl-d1lcl (2_19_1_3)	3nn9-d3nn9 (2_45_1_1)	1kcw-d1kcw_5 (2_5_1_3)	1ciy-d1ciy_1 (2_13_1_2)	1sacb-d1saca (2_19_1_4)	2prd-d2prd (2_26_5_1)	1cpn-d1cpn (2_19_1_2)	1prn-d1prn (6_7_1_1)	1dlc-d1dlc_1 (2_13_1_2)
77	1bgl-d1bgla5	3_1_1_3	292	5	0	1	0	1	0	1	2bnh-d1dfji (3_7_1_1)	1eceb-d1ecea (3_1_1_3)	2aaa-d2aaa_2 (3_1_1_1)	1edg-d1edg (3_1_1_3)	2tmda-d2tmda1 (3_1_7_1)	1pnr-d1pnra2 (3_72_1_1)	1ghr-d1ghr (3_1_1_3)	1dora-d1dora (3_1_7_1)	1tlfa-d1tlfa (3_72_1_1)
78	1bhp-d1bhp	7_10_1_1	45	2	1	1	0	0	0	0	1ccn-d1cnr (7_10_1_1)	1nis-d1aco_2 (3_63_1_1)	1perl-d1r69 (1_30_1_2)	2tct-d2tct_1 (1_31_1_1)	1mml-d1mml (5_9_1_2)	1faga-d2bmha (1_75_1_1)	1dik-d1dik_3 (4_83_1_4)	1csr-d1csh (1_74_1_1)	1sbp-d1sbp (3_73_1_1)
79	1bia-d1bia_1	1_4_3_1	63	1	0	0	1	1	0	1	1dpra-d1dpra1 (1_4_3_11)	1vola-d1vola1 (1_59_1_2)	1rro-d1rro (1_34_1_4)	1pdn-d1pdnc (1_4_1_4)	2wrpr-d2wrpr (1_78_1_1)	1hvd-d1hvd (1_51_1_1)	1copd-d1copd (1_30_1_2)	1hddc-d1enh (1_4_1_1)	2cpo-d1cpo_1 (1_34_2_1)
80	1bia-d1bia_2	2_21_1_1	47	1	0	0	0	0	0	0	1ospo-d1ospo (2_52_1_1)	1msad-d1msaa (2_54_1_1)	1ure-d1ifc (2_41_1_2)	1bbpa-d1bbpa (2_41_1_1)	2hmb-d1hms (2_41_1_2)	1kit-d1kit_2 (2_19_1_6)	1sacb-d1saca (2_19_1_4)	1nfp-d1nfp (3_1_13_2)	3nn9-d3nn9 (2_45_1_1)
81	1bip-d1bip	1_42_1_2	122	1	0	0	0	0	0	0	1cem-d1cem (1_73_1_2)	2lbp-d2lbp (3_72_1_1)	1pgo-d2pgd_1 (1_71_1_1)	2mtac-d2mtac (1_3_1_1)	1lucb-d1lucb (3_1_13_1)	1rfba-d1rfba (1_25_1_3)	1rlr-d1rlr_1 (1_68_1_1)	1imba-d2hhma (5_8_1_2)	1broa-d1broa (3_50_1_5)
82	1blba-d2bb2_2	2_9_1_1	91	3	1	1	1	1	0	0	4gcr-d4gcr_2 (2_9_1_1)	1gcs-d4gcr_1 (2_9_1_1)	1prr-d1prr_1 (2_9_1_2)	1prs-d1prr_2 (2_9_1_2)	2omf-d2omf (6_7_1_1)	1rgs-d1rgs_2 (2_58_3_2)	2trcb-d2trcb (2_46_3_1)	1juy-d1adea (3_25_1_5)	2pec-d2pec (2_56_1_1)
83	1bll-d1lam_2	3_52_3_3	325	1	0	0	1	1	0	0	1amp-d1amp (3_52_3_4)	1scua-d1scua1 (3_13_3_1)	1eri-d1eria (3_38_1_1)	1bya-d1byb (3_1_1_2)	3chy-d3chy (3_13_2_1)	1wsyb-d1wsyb (3_59_1_1)	1php-d1php (3_66_1_1)	1tlfa-d1tlfa (3_72_1_1)	1lyla-d1lyla2 (4_59_1_1)
84	1bmfc-d1bmfa3	3_25_1_6	285	2	1	1	0	0	0	0	1cowe-d1bmfd3 (3_25_1_6)	2chr-d2chr_1 (3_1_6_2)	1gca-d1gca (3_72_1_1)	1gtra-d1gtra2 (3_15_1_1)	1rvva-d1rvva (3_9_1_1)	1miob-d1miob (3_67_1_1)	1fbaa-d1fbaa (3_1_3_1)	1tlfa-d1tlfa (3_72_1_1)	1rnl-d1rnl_2 (3_13_2_1)
85	1bmfd-d1bmfd1	1_55_1_1	118	2	0	1	0	0	0	0	1mtyg-d1mmog (1_22_1_1)	1jud-d1jud (5_18_1_1)	1cowa-d1bmfa1 (1_55_1_1)	1tkaa-d1trka1 (3_24_1_2)	1jkw-d1jkw_1 (1_59_1_1)	1lis-d1lis (1_17_1_1)	1vin-d1vin_2 (1_59_1_1)	1hvd-d1hvd (1_51_1_1)	1zymb-d1zyma (3_5_1_2)
86	1bmta-d1bmta1	1_39_1_1	90	1	0	0	0	0	0	0	1mrrb-d1riba (1_24_1_2)	1cola-d1cola (6_1_1_1)	1occc-d1occc1 (6_5_1_1)	1hlb-d1hlb (1_1_1_1)	1xsm-d1xsm (1_24_1_2)	1sesa-d1seta1 (1_2_3_1)	1fps-d1fps (1_91_1_1)	2myd-d1mbd (1_1_1_1)	1csr-d1csh (1_74_1_1)
87	1bmta-d1bmta2	3_13_5_1	156	1	0	0	0	0	1	1	1scub-d1scub1 (3_13_3_1)	1pea-d1pea (3_72_1_1)	2lbp-d2lbp (3_72_1_1)	1gca-d1gca (3_72_1_1)	1miob-d1miob (3_67_1_1)	1igs-d1igs (3_1_8_1)	1tlfa-d1tlfa (3_72_1_1)	1pdo-d1pdo (3_40_1_1)	1fbaa-d1fbaa (3_1_3_1)
88	1bmv1-d1bmv1	2_8_1_2	185	4	0	0	0	0	0	0	1prn-d1prn (6_7_1_1)	2omf-d2omf (6_7_1_1)	1svb-d1svb_2 (6_10_1_1)	1tnra-d1tnra (2_17_1_1)	2por-d2por (6_7_1_1)	1mpmb-d1mal (6_7_1_2)	2plv-d1pvc1 (2_8_1_4)	1dlc-d1dlc_2 (2_53_2_1)	1bbpa-d1bbpa (2_41_1_1)
89	1bnb-d1bnb	7_7_1_1	38	5	1	1	0	0	0	0	1dfna-d1dfna (7_7_1_1)	2sh1-d1sh1 (7_7_1_1)	4aahc-d4aaha (2_47_1_1)	1mtyd-d1mmod (1_24_1_2)	2prd-d2prd (2_26_5_1)	1fica-d3fib (4_98_1_1)	1han-d1han_2 (4_20_1_3)	1tdta-d1tdta (2_57_1_2)	2por-d2por (6_7_1_1)
90	1bncb-d1bnca2	3_20_1_1	114	1	0	0	0	0	0	0	1gca-d1gca (3_72_1_1)	2dri-d2dri (3_72_1_1)	1kob-d1koba (5_1_1_1)	1mba-d1mba (1_1_1_1)	1asza-d1asya2 (4_59_1_1)	1miob-d1miob (3_67_1_1)	1cyx-d1cyx (2_5_1_2)	1art-d1art (3_48_1_1)	1qbb-d1qba_4 (4_50_2_1)
91	1bnc-d1bnca1	2_59_2_1	116	1	0	0	0	0	0	0	1psda-d1psda2 (3_19_1_4)	1dxy-d1dxy_1 (3_13_9_1)	3btoa-d2ohxa2 (3_19_1_1)	1fld-d5nul (3_13_4_1)	1leha-d1leha1 (3_19_1_7)	1dxy-d1dxy_2 (3_19_1_4)	1daaa-d1daaa (5_14_1_1)	1tlfa-d1tlfa (3_72_1_1)	1lvl-d1lvl_2 (3_4_1_4)
92	1bove-d1bova	2_26_2_1	69	6	0	0	0	0	0	0	1asza-d1asya2 (4_59_1_1)	1nfp-d1nfp (3_1_13_2)	1gym-d1gym (3_1_15_2)	1gdob-d1gdoa (4_88_1_1)	1leha-d1leha1 (3_19_1_7)	1hgea-d1hgea (2_14_1_2)	1bcpb-d1prtb1 (2_26_2_1)	1mtyd-d1mmod (1_24_1_2)	3chy-d3chy (3_13_2_1)

93	1brnl-d1brnl	4_1_1_1	108	3	1	1	0	0	0	0	1gmqb-d1gmpa (4_1_1_1)	3rnt-d9rnt (4_1_1_1)	119l-d119l (4_2_1_3)	4aahc-d4aaha (2_47_1_1)	1eur-d1eur (2_45_1_1)	1gpr-d1gpr (2_59_3_1)	1vhh-d1vhh (4_34_1_2)	1ospo-d1ospo (2_52_1_1)	2sil-d2sil (2_45_1_1)
94	1broa-d1broa	3_50_1_5	277	1	0	0	1	1	0	0	2dhd-d1ede (3_50_1_3)	1yasa-d1yasa (3_50_1_10)	1taha-d1tahb (3_50_1_8)	1lbt-d1tca (3_50_1_7)	1xzl-d1cus (3_13_7_1)	1leha-d1leha1 (3_19_1_7)	1fjm-d1fjma (4_91_1_2)	1bmta-d1bmta2 (3_13_5_1)	2bnh-d1dfji (3_7_1_1)
95	1btga-d1bet	7_14_1_3	107	1	0	0	1	1	0	0	1aocb-d1aoca (7_14_1_5)	1bvp1-d1bvp12 (2_14_1_1)	1ahsa-d1ahsa (2_14_1_1)	1lylb-d1lyla1 (2_26_4_1)	1ciy-d1ciy_2 (2_53_2_1)	2cas-d2cas (2_8_1_4)	1toxa-d1ddt_1 (2_2_1_1)	1mkaa-d1mkaa (4_21_1_1)	1p03a-d2alp (2_31_1_1)
96	1btl-d1btl	5_4_1_1	263	3	1	1	0	0	0	0	3pte-d3pte (5_4_1_1)	2blta-d2blta (5_4_1_1)	2ada-d1add (3_1_2_1)	1lyla-d1lyla2 (4_59_1_1)	1hrda-d1hrda2 (3_54_1_1)	1gln-d1gln_2 (3_15_1_1)	1mrj-d1mrj (4_94_1_1)	2hnp-d2hnp (3_32_1_2)	1pgo-d2pgd_1 (1_71_1_1)
97	1btn-d1btn	2_37_1_1	106	5	1	1	0	0	0	0	1pls-d1pls (2_37_1_1)	1obpa-d1obpa (2_41_1_1)	1gph1-d1gph12 (4_88_1_1)	1hpca-d1htp (2_59_1_1)	1dona-d1dona (4_7_1_1)	1nfp-d1nfp (3_1_13_2)	1ytfa-d1ytba1 (4_74_1_1)	1bdmb-d1bdma2 (4_92_1_1)	3cd4-d3cd4_1 (2_1_1_1)
98	1buca-d1buca1	1_23_6_1	151	1	0	0	0	0	0	1	1occa-d1occa1 (6_5_1_1)	1occc-d1occc1 (6_5_1_1)	1rcd-d1rcd (1_24_1_1)	1lpe-d1lpe (1_23_1_1)	1xsm-d1xsm (1_24_1_2)	2liga-d2liga (1_23_2_1)	1aep-d1aep (1_49_1_1)	1dlc-d1dlc_3 (6_1_3_1)	1afra-d1afra (1_24_1_2)
99	1buca-d1buca2	5_7_1_1	232	1	0	0	0	0	0	0	1gln-d1gln_1 (1_67_1_1)	1itha-d1itha (1_1_1_1)	1csr-d1csh (1_74_1_1)	1cpca-d1cpca (1_1_1_2)	1mba-d1mba (1_1_1_1)	1flp-d1flp (1_1_1_1)	1mmob-d1mmob (1_24_1_2)	1cpcb-d1cpcb (1_1_1_2)	1occa-d1occa1 (6_5_1_1)
100	1bunb-d1bunb	7_6_1_1	61	3	1	1	0	0	0	0	1knt-d1knt (7_6_1_1)	1mtnh-d1bpi (7_6_1_1)	1gof-d1gof_3 (2_46_1_1)	1seta-d1seta2 (4_59_1_1)	1yua-d1yua_2 (4_67_1_2)	1sty-d1sty (2_26_1_1)	1bgl-d1bgla5 (3_1_1_3)	1cdaa-d1aly (2_17_1_1)	1smpi-d1smpi (2_42_2_1)
101	1bvp1-d1bvp12	2_14_1_1	134	2	1	1	0	0	0	0	1ahsa-d1ahsa (2_14_1_1)	1sacb-d1saca (2_19_1_4)	1pax-d1pax_2 (4_95_1_2)	5sgae-d1sgc (2_31_1_1)	2omf-d2omf (6_7_1_1)	1svb-d1svb_2 (6_10_1_1)	1aspb-d1aoza1 (2_5_1_3)	1kit-d1kit_2 (2_19_1_6)	2hmb-d1hms (2_41_1_2)
102	1bw4-d1bw3	2_35_1_2	125	1	0	0	0	0	0	0	1asu-d1asu (3_41_3_2)	1nsdb-d1nsca (2_45_1_1)	1hcz-d1ctm_2 (2_59_2_2)	3nn9-d3nn9 (2_45_1_1)	1inp-d1inp (5_8_1_3)	2mipa-d1idaa (2_34_1_1)	1clc-d1clc_2 (2_1_1_5)	1leha-d1leha2 (3_54_1_1)	1htt-d1htta1 (3_37_1_1)
103	1bya-d1byb	3_1_1_2	490	1	0	0	0	0	1	1	1oya-d1oya (3_1_7_1)	1pea-d1pea (3_72_1_1)	1dik-d1dik_1 (3_1_9_2)	2tmda-d2tmda1 (3_1_7_1)	1tlfa-d1tlfa (3_72_1_1)	1edg-d1edg (3_1_1_3)	4xis-d2xis (3_1_12_1)	1nai-d1xel (3_19_1_2)	1gky-d1gky (3_25_1_1)
104	1c5a-d1c5a	1_40_1_1	65	1	0	0	0	0	0	0	1mtyd-d1mmod (1_24_1_2)	1yrnb-d1yrnb (1_4_1_1)	1rcd-d1rcd (1_24_1_1)	1dik-d1dik_1 (3_1_9_2)	2hbg-d1hbg (1_1_1_1)	1afra-d1afra (1_24_1_2)	1hlb-d1hlb (1_1_1_1)	2gpb-d1gpb (3_68_1_2)	1pmi-d1pmi (2_58_2_1)
105	1cb2b-d1cb2a	3_2_1_1	363	2	1	1	0	0	0	0	1tml-d1tml (3_2_1_1)	1dhr-d1dhr (3_19_1_2)	1edg-d1edg (3_1_1_3)	2tmda-d2tmda1 (3_1_7_1)	1pnr-d1pnra2 (3_72_1_1)	1eny-d1eny (3_19_1_2)	1cyda-d1cyda (3_19_1_2)	1gca-d1gca (3_72_1_1)	1oya-d1oya (3_1_7_1)
106	1cbg-d1cbg	3_1_1_4	490	1	0	0	1	1	0	1	1xyza-d1xyza (3_1_1_3)	1edg-d1edg (3_1_1_3)	1mmob-d1mmob (1_24_1_2)	1eceb-d1ecea (3_1_1_3)	4xis-d2xis (3_1_12_1)	1lucb-d1lucb (3_1_13_1)	2dri-d2dri (3_72_1_1)	1dik-d1dik_3 (4_83_1_4)	1tig-d1tig (4_35_1_1)
107	1cbh-d1cbh	7_3_7_1	36	1	0	0	0	0	1	1	1gur-d1gur (7_3_4_1)	1amy-d1amy_1 (2_48_1_1)	1ahsa-d1ahsa (2_14_1_1)	2csn-d1csn (5_1_1_1)	2cbp-d2cbp (2_5_1_1)	3nn9-d3nn9 (2_45_1_1)	1tnrr-d1ncfa2 (7_22_1_1)	1han-d1han_2 (4_20_1_3)	1bvp1-d1bvp12 (2_14_1_1)
108	1cby-d1cby	4_58_1_1	227	1	0	0	0	0	0	0	1eft-d1eft_3 (3_25_1_3)	2bnh-d1dfji (3_7_1_1)	1fbaa-d1fbaa (3_1_3_1)	1gtma-d1gtma2 (3_54_1_1)	1orda-d1orda2 (3_48_1_4)	1obpa-d1obpa (2_41_1_1)	1gdob-d1gdoa (4_88_1_1)	1mioa-d1mioa (3_67_1_1)	1clc-d1clc_1 (1_73_1_2)
109	1cc5-d1cc5	1_3_1_1	83	7	1	1	0	1	0	0	1gks-d1gks (1_3_1_1)	1cyj-d1cyi (1_3_1_1)	1broa-d1broa (3_50_1_5)	1fcdc-d1fcdc1 (1_3_1_3)	3dfr-d3dfr (3_53_1_1)	2mtac-d2mtac (1_3_1_1)	1msec-d1msec1 (1_4_1_3)	1occd-d1occd1 (6_5_1_1)	1lki-d1lki (1_25_1_1)
110	1ccn-d1cnr	7_10_1_1	46	2	1	1	0	0	0	0	1bhp-d1bhp (7_10_1_1)	1fipa-d1fipa (1_76_1_1)	2sas-d2sas (1_34_1_5)	2gpb-d1gpb (3_68_1_2)	1bia-d1bia_1 (1_4_3_1)	2wrpr-d2wrpr (1_78_1_1)	1neq-d1ner (1_30_1_2)	1lba-d1lba (4_64_1_1)	1pdn-d1pdnc (1_4_1_4)
111	1cdaa-d1aly	2_17_1_1	146	3	1	1	0	0	0	0	1tnfa-d1tnfa (2_17_1_1)	1tnra-d1tnra (2_17_1_1)	1gof-d1gof_3 (2_46_1_1)	1ggt-d1ggta2 (2_1_4_1)	1mpmb-d1mal (6_7_1_2)	3nn9-d3nn9 (2_45_1_1)	1ouna-d1ouna (4_14_3_2)	1fem-d1hbp (2_41_1_1)	1prn-d1prn (6_7_1_1)
112	1cdcb-d1cdcb	2_1_1_1	96	4	0	0	1	1	1	1	1tlk-d1tlk (2_1_1_4)	1vcaa-d1vcaa1 (2_1_1_3)	1bgl-d1bgla1 (2_1_3_1)	1ospo-d1ospo (2_52_1_1)	2ncm-d2ncm (2_1_1_4)	1kiqa-d1vfba (2_1_1_1)	1gen-d1gen (2_44_1_1)	2trcb-d2trcb (2_46_3_1)	3cd4-d3cd4_1 (2_1_1_1)
113	1cdi-d3cd4_2	2_1_1_3	81	3	0	0	1	1	0	0	1vcaa-d1vcaa2 (2_1_1_4)	2ncm-d2ncm (2_1_1_4)	1kiqa-d1vfba (2_1_1_1)	1tlk-d1tlk (2_1_1_4)	1wit-d1wiu (2_1_1_4)	1akl-d1kapp1 (2_55_1_1)	1svb-d1svb_2 (6_10_1_1)	1tnn-d1tnm (2_1_1_4)	1ospo-d1ospo (2_52_1_1)
114	1cdr-d1cdq	7_5_1_3	77	1	0	0	0	0	0	0	1pkp-d1pkp_1 (4_11_1_1)	1froa-d1froa (4_20_1_1)	2ctb-d2ctb (3_52_3_1)	1lcl-d1lcl (2_19_1_3)	1azca-d1aiza (2_5_1_1)	1nsdb-d1nsca (2_45_1_1)	1lyla-d1lyla2 (4_59_1_1)	1kcw-d1kcw_1 (2_5_1_3)	1gdob-d1gdoa (4_88_1_1)
115	1cem-d1cem	1_73_1_2	363	2	0	1	1	1	0	0	1glm-d1glm (1_73_1_1)	1prcl-d1prcl1 (6_5_1_1)	1clc-d1clc_1 (1_73_1_2)	2lbp-d2lbp (3_72_1_1)	1csr-d1csh (1_74_1_1)	1occa-d1occa1 (6_5_1_1)	1sly-d1sly_1 (1_84_4_1)	1scha-d1scha (1_65_1_1)	1mrrb-d1riba (1_24_1_2)
116	1cew-d1cewi	4_14_1_2	108	2	0	0	1	1	0	1	1mola-d1mola (4_14_1_1)	1mxb-d1mxa_3 (4_75_1_1)	1ouna-d1ouna (4_14_3_2)	1mkaa-d1mkaa (4_21_1_1)	1gdob-d1gdoa (4_88_1_1)	1mxa-d1mxa_1 (4_75_1_1)	1ris-d1ris (4_33_11_1)	1ggt-d1ggta2 (2_1_4_1)	1mbb-d1mbb_1 (4_84_1_2)
117	1cfb-d1cfb_1	2_1_2_1	100	4	1	1	0	0	0	0	1fna-d1fna (2_1_2_1)	1ten-d1ten (2_1_2_1)	1dlc-d1dlc_2 (2_53_2_1)	4aahc-d4aaha (2_47_1_1)	3nn9-d3nn9 (2_45_1_1)	2hnp-d2hnp (3_32_1_2)	2pcd-d2pcda (2_3_3_1)	1vmoa-d1vmoa (2_53_1_1)	2sil-d2sil (2_45_1_1)
118	1cgpb-d1cgpa1	1_4_3_4	68	1	0	0	1	1	1	1	1dpra-d1dpra1 (1_4_3_11)	1pdn-d1pdnc (1_4_1_4)	1bia-d1bia_1 (1_4_3_1)	1apme-d1apme (5_1_1_1)	1tpfa-d1tpfa (3_1_11_1)	1rvva-d1rvva (3_9_1_1)	1regx-d1regx (4_33_19_1)	1bmfd-d1bmfd1 (1_55_1_1)	1dhr-d1dhr (3_19_1_2)
119	1chc-d1chc	7_35_1_1	68	1	0	0	0	0	0	0	1wba-d1wba (2_28_3_1)	1faq-d1faq (7_38_1_1)	1vmoa-d1vmoa (2_53_1_1)	1bnc-d1bnca1 (2_59_2_1)	1aspb-d1aoza1 (2_5_1_3)	2trcb-d2trcb (2_46_3_1)	1han-d1han_2 (4_20_1_3)	1bgl-d1bgla5 (3_1_1_3)	1tfi-d1tfi (7_32_3_1)
120	1chd-d1chd	3_27_1_1	198	1	0	0	0	0	0	0	1dora-d1dora (3_1_7_1)	1apa-d1apa (4_94_1_1)	2lbp-d2lbp (3_72_1_1)	1xva-d1xvaa (3_47_1_2)	1onea-d1ebha1 (3_1_6_1)	1rnl-d1rnl_2 (3_13_2_1)	2dri-d2dri (3_72_1_1)	1mrj-d1mrj (4_94_1_1)	1kfd-d1kfd_1 (3_41_3_4)
121	1chka-d1chka	4_2_1_6	238	1	0	0	0	0	0	0	1xsm-d1xsm (1_24_1_2)	1sly-d1sly_1 (1_84_4_1)	1ilk-d1ilk (1_25_1_3)	1flp-d1flp (1_1_1_1)	1prcl-d1prcl1 (6_5_1_1)	1lh7-d1lh1 (1_1_1_1)	1csr-d1csh (1_74_1_1)	1rpa-d1rpa (3_43_1_2)	1afra-d1afra (1_24_1_2)
122	1chl-d1chl	7_3_6_2	36	4	0	0	0	0	0	0	1hcd-d1hcd (2_28_4_1)	2naca-d2naca2 (3_19_1_4)	1oxa-d1oxa (1_75_1_1)	1pmi-d1pmi (2_58_2_1)	1gbs-d1gbs (4_2_1_4)	4kbpa-d4kbpa2 (4_91_1_1)	1mpd-d1mpb (3_73_1_1)	1fd2-d1fd2 (4_33_1_2)	1dxy-d1dxy_2 (3_19_1_4)
123	1chma-d1chma1	3_41_2_1	155	1	0	0	0	0	0	0	1tlfa-d1tlfa (3_72_1_1)	3tgl-d3tgl (3_50_1_7)	1hrda-d1hrda1 (3_19_1_7)	1gtma-d1gtma1 (3_19_1_7)	1plr-d1plq_2 (4_76_1_2)	1eft-d1eft_3 (3_25_1_3)	2hmx-d2hmx (1_58_1_1)	1php-d1php (3_66_1_1)	1jeva-d2olba (3_73_1_1)
124	1chma-d1chma2	4_72_1_1	246	1	0	0	0	0	0	0	1rpa-d1rpa (3_43_1_2)	1dkza-d1dkza (5_17_1_1)	1xsm-d1xsm (1_24_1_2)	1zymb-d1zyma (3_5_1_2)	1ngi-d1hpm_2 (3_41_1_1)	1gcb-d1gcb (4_3_1_1)	1pne-d1pne (4_61_1_1)	1lpe-d1lpe (1_23_1_1)	1rcd-d1rcd (1_24_1_1)
125	1chra-d2chr_2	4_31_1_1	126	3	1	1	0	0	0	0	2mnr-d2mnr_2 (4_31_1_1)	1onea-d1ebha2 (4_31_1_1)	1dkza-d1dkza (5_17_1_1)	1mba-d1mba (1_1_1_1)	1tfe-d1tfe (4_24_1_1)	3sdha-d3sdha (1_1_1_1)	1dik-d1dik_3 (4_83_1_4)	1ggt-d1ggta2 (2_1_4_1)	1gtqa-d1gtqa (4_54_1_2)
126	1cid-d1cid_1	2_1_1_1	105	4	1	1	1	1	0	0	3cd4-d3cd4_1 (2_1_1_1)	1kiqa-d1vfba (2_1_1_1)	1wit-d1wiu (2_1_1_4)	1vcaa-d1vcaa2 (2_1_1_4)	1ure-d1ifc (2_41_1_2)	1bbpa-d1bbpa (2_41_1_1)	1ospo-d1ospo (2_52_1_1)	1nsdb-d1nsca (2_45_1_1)	2ncm-d2ncm (2_1_1_4)
127	1cid-d1cid_2	2_1_1_3	72	3	0	0	0	1	0	0	1lcl-d1lcl (2_19_1_3)	1nsdb-d1nsca (2_45_1_1)	3nn9-d3nn9 (2_45_1_1)	2hmb-d1hms (2_41_1_2)	1cdcb-d1cdcb (2_1_1_1)	1kit-d1kit_1 (2_19_1_6)	1vcaa-d1vcaa1 (2_1_1_3)	1cpn-d1cpn (2_19_1_2)	1cdi-d3cd4_2 (2_1_1_3)
128	1ciy-d1ciy_1	2_13_1_2	148	2	1	1	0	0	0	0	1dlc-d1dlc_1 (2_13_1_2)	1pmi-d1pmi (2_58_2_1)	3nn9-d3nn9 (2_45_1_1)	1lcl-d1lcl (2_19_1_3)	2sil-d2sil (2_45_1_1)	1bgl-d1bgla1 (2_1_3_1)	1bgl-d1bgla2 (2_1_3_1)	1kit-d1kit_1 (2_19_1_6)	1nsdb-d1nsca (2_45_1_1)
129	1ciy-d1ciy_2	2_53_2_1	206	2	1	1	0	0	0	0	1dlc-d1dlc_2 (2_53_2_1)	1qbb-d1qba_2 (2_2_2_3)	4kbpb-d4kbpa1 (2_1_9_1)	1obpa-d1obpa (2_41_1_1)	1asob-d1aoza3 (2_5_1_3)	3nn9-d3nn9 (2_45_1_1)	1svb-d1svb_2 (6_10_1_1)	1eal-d1eal (2_41_1_2)	1cid-d1cid_1 (2_1_1_1)
130	1ciy-d1ciy_3	6_1_3_1	223	2	1	1	0	0	0	0	1dlc-d1dlc_3 (6_1_3_1)	1mrrb-d1riba (1_24_1_2)	1occc-d1occc1 (6_5_1_1)	1xsm-d1xsm (1_24_1_2)	1mmob-d1mmob (1_24_1_2)	1sly-d1sly_1 (1_84_4_1)	1occa-d1occa1 (6_5_1_1)	1fps-d1fps (1_91_1_1)	1aep-d1aep (1_49_1_1)
131	1clc-d1clc_1	1_73_1_2	441	2	0	0	0	0	0	0	1pgo-d2pgd_1 (1_71_1_1)	1sly-d1sly_1 (1_84_4_1)	1afra-d1afra (1_24_1_2)	1bfra-d1bcfa (1_24_1_1)	2hbg-d1hbg (1_1_1_1)	1lucb-d1lucb (3_1_13_1)	1occa-d1occa1 (6_5_1_1)	1rcd-d1rcd (1_24_1_1)	1aep-d1aep (1_49_1_1)
132	1clc-d1clc_2	2_1_1_5	100	6	1	1	0	0	0	0	1gog-d1gof_1 (2_1_1_5)	1gof-d1gof_3 (2_46_1_1)	1hpl-d1hpla1 (2_10_2_1)	1ospo-d1ospo (2_52_1_1)	2bbkh-d2bbkh (2_46_2_1)	2prd-d2prd (2_26_5_1)	1ggt-d1ggta4 (4_3_1_2)	1bgl-d1bgla2 (2_1_3_1)	2trcb-d2trcb (2_46_3_1)
133	1cmc-d1cmba	1_36_1_2	104	1	0	0	0	0	0	0	1vin-d1vin_1 (1_59_1_1)	1dik-d1dik_3 (4_83_1_4)	1flp-d1flp (1_1_1_1)	1pgo-d2pgd_1 (1_71_1_1)	2mnr-d2mnr_1 (3_1_6_2)	1vola-d1vola1 (1_59_1_2)	1gca-d1gca (3_72_1_1)	2lbp-d2lbp (3_72_1_1)	1gtra-d1gtra2 (3_15_1_1)
134	1cnf-d2cnd_1	2_29_1_1	114	2	0	1	1	1	0	0	2pia-d2pia_1 (2_29_1_2)	1fnc-d1fnb_1 (2_29_1_1)	1bbpa-d1bbpa (2_41_1_1)	1hgea-d1hgea (2_14_1_2)	2trcb-d2trcb (2_46_3_1)	4aahc-d4aaha (2_47_1_1)	1gen-d1gen (2_44_1_1)	1kcw-d1kcw_5 (2_5_1_3)	1cyx-d1cyx (2_5_1_2)
135	1cnf-d2cnd_2	3_14_1_1	146	2	1	1	1	1	0	0	1fnc-d1fnb_2 (3_14_1_1)	2pia-d2pia_2 (3_14_1_2)	1tib-d1tib (3_50_1_7)	2mnr-d2mnr_1 (3_1_6_2)	1xoa-d2trxa (3_33_1_1)	1tpfa-d1tpfa (3_1_11_1)	1tkca-d1trka3 (3_34_1_1)	1pnr-d1pnra2 (3_72_1_1)	1cowe-d1bmfd3 (3_25_1_6)
136	1coa-d1coai	4_22_1_1	64	3	1	1	0	0	0	0	1csei-d1csei (4_22_1_1)	1mit-d1tin (4_22_1_1)	1scb-d1csee (3_28_1_1)	1ggt-d1ggta4 (4_3_1_2)	2anhb-d2anha (3_56_1_1)	1emd-d2cmd_1 (3_19_1_5)	1rpl-d1rpl (5_10_1_1)	1hpm-d1hpm_1 (3_41_1_1)	6ldh-d1ldm_1 (3_19_1_5)
137	1cof-d1ahq	4_60_1_2	133	1	0	0	0	1	0	0	1gtma-d1gtma2 (3_54_1_1)	2vik-d2vik (4_60_1_1)	1obpa-d1obpa (2_41_1_1)	1acf-d1acf (4_61_1_1)	1alo-d1alo_7 (4_77_1_1)	1llda-d1llda1 (3_19_1_5)	1asu-d1asu (3_41_3_2)	1psda-d1psda3 (4_33_15_1)	1ytn-d1ypta (3_32_1_2)
138	1coi-d1coi	9_4_1_1	29	1	0	0	0	0	0	0	1rcd-d1rcd (1_24_1_1)	1lh7-d1lh1 (1_1_1_1)	1sesa-d1seta1 (1_2_3_1)	4fua-d1fua (3_55_1_1)	1bbha-d1bbha (1_23_3_2)	2liga-d2liga (1_23_2_1)	1lpe-d1lpe (1_23_1_1)	1lki-d1lki (1_25_1_1)	1runb-d1cgpa2 (2_58_3_1)
139	1cola-d1cola	6_1_1_1	197	1	0	0	0	0	1	1	1dlc-d1dlc_3 (6_1_3_1)	1sly-d1sly_1 (1_84_4_1)	1occc-d1occc1 (6_5_1_1)	1afra-d1afra (1_24_1_2)	1ciy-d1ciy_3 (6_1_3_1)	1xsm-d1xsm (1_24_1_2)	1rcd-d1rcd (1_24_1_1)	1cpcb-d1cpcb (1_1_1_2)	1cpca-d1cpca (1_1_1_2)
140	1con-d1scs	2_19_1_1	237	1	0	0	0	0	0	0	1mpmb-d1mal (6_7_1_2)	1ospo-d1ospo (2_52_1_1)	2por-d2por (6_7_1_1)	1ure-d1ifc (2_41_1_2)	2omf-d2omf (6_7_1_1)	2hmb-d1hms (2_41_1_2)	1obpa-d1obpa (2_41_1_1)	1kit-d1kit_2 (2_19_1_6)	1prn-d1prn (6_7_1_1)

141	1coo-d1coo	1_32_1_1	81	1	0	0	0	0	0	0	1fps-d1fps (1_91_1_1)	119l-d119l (4_2_1_3)	1gtra-d1gtra2 (3_15_1_1)	1lef-d1lefa (1_20_1_1)	1sly-d1sly_1 (1_84_4_1)	1fjm-d1fjma (4_91_1_2)	1broa-d1broa (3_50_1_5)	1neq-d1ner (1_30_1_2)	1iov-d2dln_2 (4_83_1_1)
142	1copd-d1copd	1_30_1_2	66	5	0	0	0	0	0	0	2wrpr-d2wrpr (1_78_1_1)	1bia-d1bia_1 (1_4_3_1)	1ctt-d1ctt_1 (3_75_1_1)	1srsa-d1srsa (4_47_1_1)	1han-d1han_1 (4_20_1_3)	1gtma-d1gtma2 (3_54_1_1)	1ctt-d1ctt_2 (3_75_1_1)	1apa-d1apa (4_94_1_1)	1buca-d1buca2 (5_7_1_1)
143	1cowa-d1bmfa1	1_55_1_1	131	2	0	0	0	0	0	0	2hbg-d1hbg (1_1_1_1)	1vin-d1vin_1 (1_59_1_1)	1cola-d1cola (6_1_1_1)	1gsea-d1gsea1 (1_38_1_1)	1faga-d2bmha (1_75_1_1)	1osa-d1osa (1_34_1_5)	1mioa-d1mioa (3_67_1_1)	1oxa-d1oxa (1_75_1_1)	1jkw-d1jkw_1 (1_59_1_1)
144	1cowe-d1bmfd3	3_25_1_6	276	2	1	1	0	0	0	0	1bmfc-d1bmfa3 (3_25_1_6)	1taha-d1tahb (3_50_1_8)	1pnr-d1pnra2 (3_72_1_1)	1ahia-d1fmca (3_19_1_2)	2chr-d2chr_1 (3_1_6_2)	1scub-d1scub1 (3_13_3_1)	1rvva-d1rvva (3_9_1_1)	1fbaa-d1fbaa (3_1_3_1)	2dri-d2dri (3_72_1_1)
145	1coy-d1coy_1	3_4_1_2	315	2	1	1	0	1	0	0	1gal-d1gal_1 (3_4_1_2)	1hpm-d1hpm_1 (3_41_1_1)	1prtf-d1prtf (2_26_2_1)	1eac-d1eaf (3_30_1_1)	1geua-d1gesa2 (3_4_1_4)	2tmda-d2tmda2 (3_4_1_1)	1tlfa-d1tlfa (3_72_1_1)	1febb-d2tpra2 (3_4_1_4)	1asza-d1asya2 (4_59_1_1)
146	1cpca-d1cpca	1_1_1_2	162	3	1	1	1	1	0	0	1cpcb-d1cpcb (1_1_1_2)	1alla-d1alla (1_1_1_2)	2myd-d1mbd (1_1_1_1)	3sdha-d3sdha (1_1_1_1)	1mba-d1mba (1_1_1_1)	1hvd-d1hvd (1_51_1_1)	1itha-d1itha (1_1_1_1)	1occc-d1occc1 (6_5_1_1)	1baba-d1baba (1_1_1_1)
147	1cpcb-d1cpcb	1_1_1_2	172	3	1	1	1	1	0	0	1alla-d1alla (1_1_1_2)	1cpca-d1cpca (1_1_1_2)	1lh7-d1lh1 (1_1_1_1)	1hlb-d1hlb (1_1_1_1)	1mba-d1mba (1_1_1_1)	1cola-d1cola (6_1_1_1)	1dlc-d1dlc_3 (6_1_3_1)	1occa-d1occa1 (6_5_1_1)	1itha-d1itha (1_1_1_1)
148	1cpn-d1cpn	2_19_1_2	208	1	0	0	1	1	0	0	1kit-d1kit_2 (2_19_1_6)	1lcl-d1lcl (2_19_1_3)	3nn9-d3nn9 (2_45_1_1)	1eur-d1eur (2_45_1_1)	1tul-d1tul (2_60_4_1)	1mkaa-d1mkaa (4_21_1_1)	1kit-d1kit_1 (2_19_1_6)	1p03a-d2alp (2_31_1_1)	1bbpa-d1bbpa (2_41_1_1)
149	1crr-d5p21	3_25_1_3	166	2	1	1	0	0	0	0	1eft-d1eft_3 (3_25_1_3)	3tgl-d3tgl (3_50_1_7)	1asu-d1asu (3_41_3_2)	1fld-d5nul (3_13_4_1)	3fx2-d2fx2 (3_13_4_1)	2chr-d2chr_1 (3_1_6_2)	1hmp-d1hmpa (3_44_1_1)	2dri-d2dri (3_72_1_1)	1rvva-d1rvva (3_9_1_1)
150	1csei-d1csei	4_22_1_1	63	3	1	1	0	0	0	0	1coa-d1coai (4_22_1_1)	1mit-d1tin (4_22_1_1)	1arc-d1arb (2_31_1_1)	2phl-d2phla1 (2_58_1_1)	2anhb-d2anha (3_56_1_1)	1rgs-d1rgs_2 (2_58_3_2)	1apa-d1apa (4_94_1_1)	1fiva-d1fiva (2_34_1_1)	1prch-d1prch1 (2_27_1_1)
151	1csr-d1csh	1_74_1_1	435	1	0	0	0	0	0	0	1occa-d1occa1 (6_5_1_1)	1sly-d1sly_1 (1_84_4_1)	1afra-d1afra (1_24_1_2)	1pgo-d2pgd_1 (1_71_1_1)	1hvd-d1hvd (1_51_1_1)	1xsm-d1xsm (1_24_1_2)	1occc-d1occc1 (6_5_1_1)	1prcl-d1prcl1 (6_5_1_1)	1mrrb-d1riba (1_24_1_2)
152	1ctf-d1ctf	4_26_1_1	68	1	0	0	0	0	0	0	1jud-d1jud (5_18_1_1)	1sly-d1sly_1 (1_84_4_1)	1vola-d1vola1 (1_59_1_2)	1miob-d1miob (3_67_1_1)	1pdn-d1pdnc (1_4_1_4)	1dik-d1dik_1 (3_1_9_2)	2dri-d2dri (3_72_1_1)	1orta-d1orta1 (3_58_1_1)	1occe-d1occe (1_84_7_1)
153	1ctn-d1ctn_1	2_1_1_5	109	6	0	0	0	1	0	0	1nsdb-d1nsca (2_45_1_1)	3nn9-d3nn9 (2_45_1_1)	1kit-d1kit_1 (2_19_1_6)	1cyx-d1cyx (2_5_1_2)	1vcaa-d1vcaa1 (2_1_1_3)	1kit-d1kit_2 (2_19_1_6)	1bgl-d1bgla1 (2_1_3_1)	1dlc-d1dlc_1 (2_13_1_2)	1vcaa-d1vcaa2 (2_1_1_4)
154	1ctn-d1ctn_3	4_19_3_1	74	1	0	0	0	0	0	0	3nn9-d3nn9 (2_45_1_1)	1htt-d1htta1 (3_37_1_1)	1dkza-d1dkza (5_17_1_1)	2bbkh-d2bbkh (2_46_2_1)	2qila-d1tssa2 (4_12_5_1)	1eph-d1egf (7_3_9_1)	1dlc-d1dlc_2 (2_53_2_1)	4aahc-d4aaha (2_47_1_1)	7icd-d7icd (3_57_1_1)
155	1ctt-d1ctt_1	3_75_1_1	150	2	1	1	0	0	0	0	1ctt-d1ctt_2 (3_75_1_1)	3pte-d3pte (5_4_1_1)	1gln-d1gln_2 (3_15_1_1)	7cat-d7cata (5_6_1_1)	1hddc-d1enh (1_4_1_1)	1xra-d1mxa_2 (4_75_1_1)	1xva-d1xvaa (3_47_1_2)	1wdcb-d1scmb (1_34_1_5)	1ltge-d1ltsd (2_26_2_1)
156	1ctt-d1ctt_2	3_75_1_1	144	2	1	1	0	0	0	0	1ctt-d1ctt_1 (3_75_1_1)	1ngi-d1hpm_2 (3_41_1_1)	1eft-d1eft_3 (3_25_1_3)	1obpa-d1obpa (2_41_1_1)	1ecpa-d1ecpa (3_52_1_1)	1gdob-d1gdoa (4_88_1_1)	1srsa-d1srsa (4_47_1_1)	1froa-d1froa (4_20_1_1)	1asu-d1asu (3_41_3_2)
157	1cur-d1cur	2_5_1_1	155	4	0	1	1	1	0	0	1aspb-d1aoza1 (2_5_1_3)	1kcw-d1kcw_2 (2_5_1_3)	1occb-d1occb1 (2_5_1_2)	1kcw-d1kcw_4 (2_5_1_3)	2cbp-d2cbp (2_5_1_1)	2hmb-d1hms (2_41_1_2)	1asqa-d1aoza2 (2_5_1_3)	1gen-d1gen (2_44_1_1)	1kcw-d1kcw_6 (2_5_1_3)
158	1cwpb-d1cwpa	2_8_1_2	149	4	0	0	0	0	0	0	1cfb-d1cfb_1 (2_1_2_1)	1svb-d1svb_2 (6_10_1_1)	1ggt-d1ggta2 (2_1_4_1)	1ctt-d1ctt_2 (3_75_1_1)	2pcd-d2pcda (2_3_3_1)	1dar-d1dar_3 (4_11_1_1)	1cdaa-d1aly (2_17_1_1)	1kcw-d1kcw_5 (2_5_1_3)	1tsra-d1tupa (2_2_3_1)
159	1cxh-d1cdg_2	2_3_1_1	105	1	0	0	0	0	0	0	3nn9-d3nn9 (2_45_1_1)	2hmb-d1hms (2_41_1_2)	1nsdb-d1nsca (2_45_1_1)	1seia-d1seia (4_80_1_1)	2omf-d2omf (6_7_1_1)	1bbpa-d1bbpa (2_41_1_1)	1ure-d1ifc (2_41_1_2)	1asqa-d1aoza2 (2_5_1_3)	1cid-d1cid_1 (2_1_1_1)
160	1cxh-d1cdg_4	3_1_1_1	382	4	1	1	0	1	0	1	2aaa-d2aaa_2 (3_1_1_1)	1ppi-d1ppi_2 (3_1_1_1)	1amy-d1amy_2 (3_1_1_1)	1dik-d1dik_3 (4_83_1_4)	1qba-d1qba_3 (3_1_1_6)	1vin-d1vin_1 (1_59_1_1)	1miob-d1miob (3_67_1_1)	1pnr-d1pnra2 (3_72_1_1)	1lucb-d1lucb (3_1_13_1)
161	1cxn-d1tgxa	7_5_1_1	60	1	0	0	0	0	0	0	2trcb-d2trcb (2_46_3_1)	1svb-d1svb_2 (6_10_1_1)	1kxa-d2snv (2_31_1_3)	1eur-d1eur (2_45_1_1)	1duta-d1duta (2_60_3_1)	1pii-d1pii_2 (3_1_8_1)	1bebb-d1beba (2_41_1_1)	1ure-d1ifc (2_41_1_2)	2bbkh-d2bbkh (2_46_2_1)
162	1cxsa-d1cxsa1	2_35_2_1	155	1	0	0	0	0	0	0	7icd-d7icd (3_57_1_1)	4kbpa-d4kbpa2 (4_91_1_1)	3ait-d1hoe (2_4_1_1)	1gof-d1gof_3 (2_46_1_1)	5sgae-d1sgc (2_31_1_1)	1hava-d1hava (2_31_1_4)	1bgl-d1bgla3 (2_13_1_3)	1psda-d1psda2 (3_19_1_4)	1rpa-d1rpa (3_43_1_2)
163	1cyda-d1cyda	3_19_1_2	242	6	1	1	0	1	0	0	1ahia-d1fmca (3_19_1_2)	1hdca-d1hdca (3_19_1_2)	1eny-d1eny (3_19_1_2)	1dhr-d1dhr (3_19_1_2)	1nai-d1xel (3_19_1_2)	1fbaa-d1fbaa (3_1_3_1)	3btoa-d2ohxa2 (3_19_1_1)	2lbp-d2lbp (3_72_1_1)	1tpfa-d1tpfa (3_1_11_1)
164	1cyj-d1cyi	1_3_1_1	89	7	1	1	0	0	0	0	2mtac-d2mtac (1_3_1_1)	1gks-d1gks (1_3_1_1)	2sas-d2sas (1_34_1_5)	1cc5-d1cc5 (1_3_1_1)	1xyza-d1xyza (3_1_1_3)	1dnpa-d1dnpa1 (1_69_1_1)	1mba-d1mba (1_1_1_1)	1ctf-d1ctf (4_26_1_1)	1mtyd-d1mmod (1_24_1_2)
165	1cyx-d1cyx	2_5_1_2	158	2	1	1	0	0	0	0	1occb-d1occb1 (2_5_1_2)	1eal-d1eal (2_41_1_2)	2hmb-d1hms (2_41_1_2)	1ospo-d1ospo (2_52_1_1)	1vcaa-d1vcaa1 (2_1_1_3)	1mpmb-d1mal (6_7_1_2)	1bebb-d1beba (2_41_1_1)	1xnb-d1xnb (2_19_1_8)	1noy-d1noya (3_41_3_5)
166	1d66a-d1d66a2	7_29_1_1	41	3	1	1	0	0	0	0	1pyc-d1pyc (7_29_1_1)	1pyib-d1pyia2 (7_29_1_1)	1art-d1art (3_48_1_1)	1jud-d1jud (5_18_1_1)	2gpb-d1gpb (3_68_1_2)	1bmta-d1bmta2 (3_13_5_1)	1oela-d1grl_2 (3_5_3_1)	1lvl-d1lvl_3 (4_46_1_1)	1tpfa-d1tpfa (3_1_11_1)
167	1daaa-d1daaa	5_14_1_1	277	1	0	0	0	0	0	0	1asza-d1asya2 (4_59_1_1)	1han-d1han_1 (4_20_1_3)	2chr-d2chr_1 (3_1_6_2)	1tlfa-d1tlfa (3_72_1_1)	1taha-d1tahb (3_50_1_8)	1bncb-d1bnca2 (3_20_1_1)	1bll-d1lam_2 (3_52_3_3)	1udh-d1udg (3_11_1_1)	1edt-d1edt (3_1_1_5)
168	1dar-d1dar_1	2_29_3_1	118	2	1	1	0	0	0	0	1tuia-d1eft_1 (2_29_3_1)	2trcb-d2trcb (2_46_3_1)	1dlc-d1dlc_1 (2_13_1_2)	1dlh-d1dlha2 (4_15_1_1)	1efr-d1bmfa2 (2_33_1_1)	5sgae-d1sgc (2_31_1_1)	1pex-d1pex (2_44_1_1)	4aahc-d4aaha (2_47_1_1)	1ospo-d1ospo (2_52_1_1)
169	1dar-d1dar_3	4_11_1_1	123	2	0	0	0	0	0	0	1hpm-d1hpm_1 (3_41_1_1)	1han-d1han_2 (4_20_1_3)	1leha-d1leha2 (3_54_1_1)	1hrda-d1hrda2 (3_54_1_1)	2sil-d2sil (2_45_1_1)	2prd-d2prd (2_26_5_1)	1prtf-d1prtf (2_26_2_1)	7cat-d7cata (5_6_1_1)	2hmb-d1hms (2_41_1_2)
170	1dar-d1dar_4	4_33_12_1	90	1	0	0	0	0	0	1	1fnc-d1fnb_2 (3_14_1_1)	1kob-d1koba (5_1_1_1)	1poxa-d1poxa2 (3_24_1_1)	1dcha-d1dcoa (4_38_1_1)	1fwp-d1fwp (4_33_18_1)	1psda-d1psda3 (4_33_15_1)	1reqb-d1reqb2 (3_13_5_2)	2dkb-d2dkb (3_48_1_3)	1vhh-d1vhh (4_34_1_2)
171	1dcha-d1dcoa	4_38_1_1	99	1	0	0	0	0	0	0	1jeva-d2olba (3_73_1_1)	1kob-d1koba (5_1_1_1)	1phk-d1phk (5_1_1_1)	1edt-d1edt (3_1_1_5)	1dora-d1dora (3_1_7_1)	1hrda-d1hrda1 (3_19_1_7)	1plr-d1plq_2 (4_76_1_2)	1lyla-d1lyla2 (4_59_1_1)	1fbaa-d1fbaa (3_1_3_1)
172	1dcta-d1dcta	3_47_1_4	324	2	1	1	1	1	0	0	4mhta-d1hmy (3_47_1_4)	1xva-d1xvaa (3_47_1_2)	1cyda-d1cyda (3_19_1_2)	1eceb-d1ecea (3_1_1_3)	1tlfa-d1tlfa (3_72_1_1)	1rcf-d1rcf (3_13_4_1)	1mioa-d1mioa (3_67_1_1)	2dri-d2dri (3_72_1_1)	7icd-d7icd (3_57_1_1)
173	1dec-d1dec	7_18_1_1	39	1	0	0	0	0	0	0	1hfi-d1hfi (7_15_1_1)	2pec-d2pec (2_56_1_1)	1sty-d1sty (2_26_1_1)	1oxa-d1oxa (1_75_1_1)	2prd-d2prd (2_26_5_1)	1phe-d1phb (1_75_1_1)	5sgae-d1sgc (2_31_1_1)	2bbvc-d2bbva (2_8_1_3)	1cxh-d1cdg_4 (3_1_1_1)
174	1def-d1def	4_96_1_1	147	1	0	0	0	0	0	0	1vjs-d1bplb1 (2_48_1_1)	1leha-d1leha2 (3_54_1_1)	1mola-d1mola (4_14_1_1)	1hpl-d1hpla1 (2_10_2_1)	1shca-d1shca (2_37_1_2)	1noa-d1noa (2_1_6_1)	1vmoa-d1vmoa (2_53_1_1)	1gdob-d1gdoa (4_88_1_1)	1ecpa-d1ecpa (3_52_1_1)
175	1dfna-d1dfna	7_7_1_1	30	5	1	1	0	0	0	0	1bnb-d1bnb (7_7_1_1)	5sgae-d1sgc (2_31_1_1)	1p03a-d2alp (2_31_1_1)	2act-d2act (4_3_1_1)	1xnb-d1xnb (2_19_1_8)	1iyu-d1iyu (2_59_1_1)	1lac-d1lac (2_59_1_1)	1kfd-d1kfd_1 (3_41_3_4)	1bdmb-d1bdma2 (4_92_1_1)
176	1dhma-d1dhma	4_33_8_1	83	3	1	1	0	0	0	1	2bop-d2bopa (4_33_8_1)	2pec-d2pec (2_56_1_1)	1iad-d1ast (4_50_1_4)	1pba-d1pba (4_33_3_1)	1bya-d1byb (3_1_1_2)	1dcha-d1dcoa (4_38_1_1)	1cem-d1cem (1_73_1_2)	1oiba-d1pbp (3_73_1_1)	1ghr-d1ghr (3_1_1_3)
177	1dhpa-d1dhpa	3_1_3_1	292	3	1	1	0	0	1	1	1nal1-d1nal1 (3_1_3_1)	2ada-d1add (3_1_2_1)	1mioa-d1mioa (3_67_1_1)	1dora-d1dora (3_1_7_1)	1gca-d1gca (3_72_1_1)	1tlfa-d1tlfa (3_72_1_1)	1gtma-d1gtma1 (3_19_1_7)	1pii-d1pii_2 (3_1_8_1)	7icd-d7icd (3_57_1_1)
178	1dhr-d1dhr	3_19_1_2	236	6	1	1	0	0	0	0	1hdca-d1hdca (3_19_1_2)	1ahia-d1fmca (3_19_1_2)	1cyda-d1cyda (3_19_1_2)	1eny-d1eny (3_19_1_2)	1nai-d1xel (3_19_1_2)	2dri-d2dri (3_72_1_1)	1cb2b-d1cb2a (3_2_1_1)	1tlfa-d1tlfa (3_72_1_1)	1gca-d1gca (3_72_1_1)
179	1dih-d1dih_2	4_41_1_2	110	1	0	0	0	0	0	0	1div-d1div (4_82_1_1)	2chr-d2chr_1 (3_1_6_2)	1hpm-d1hpm_1 (3_41_1_1)	1gca-d1gca (3_72_1_1)	1ouna-d1ouna (4_14_3_2)	3tgl-d3tgl (3_50_1_7)	1fbaa-d1fbaa (3_1_3_1)	1vcaa-d1vcaa1 (2_1_1_3)	1knya-d1knya (5_10_1_2)
180	1dik-d1dik_1	3_1_9_2	365	1	0	0	0	0	0	1	1occa-d1occa1 (6_5_1_1)	1pea-d1pea (3_72_1_1)	1hvd-d1hvd (1_51_1_1)	1dora-d1dora (3_1_7_1)	1miob-d1miob (3_67_1_1)	1pgo-d2pgd_1 (1_71_1_1)	4xis-d2xis (3_1_12_1)	1cola-d1cola (6_1_1_1)	1gca-d1gca (3_72_1_1)
181	1dik-d1dik_2	3_5_1_1	129	1	0	0	1	1	0	0	1zymb-d1zyma (3_5_1_2)	1gof-d1gof_3 (2_46_1_1)	2hnp-d2hnp (3_32_1_2)	3btoa-d2ohxa2 (3_19_1_1)	2bnh-d1dfji (3_7_1_1)	1asu-d1asu (3_41_3_2)	1tpfa-d1tpfa (3_1_11_1)	1gen-d1gen (2_44_1_1)	3chy-d3chy (3_13_2_1)
182	1dik-d1dik_3	4_83_1_4	375	1	0	0	0	0	0	0	1rpa-d1rpa (3_43_1_2)	1lucb-d1lucb (3_1_13_1)	1pgo-d2pgd_1 (1_71_1_1)	1nfp-d1nfp (3_1_13_2)	1prcl-d1prcl1 (6_5_1_1)	1occc-d1occc1 (6_5_1_1)	1csr-d1csh (1_74_1_1)	1cola-d1cola (6_1_1_1)	1occa-d1occa1 (6_5_1_1)
183	1div-d1div	4_82_1_1	149	1	0	0	0	0	0	0	1clc-d1clc_1 (1_73_1_2)	1fbaa-d1fbaa (3_1_3_1)	1dih-d1dih_2 (4_41_1_2)	1art-d1art (3_48_1_1)	2hmgd-d1htmb (6_2_1_1)	1prcl-d1prcl1 (6_5_1_1)	1ciy-d1ciy_3 (6_1_3_1)	1pnr-d1pnra2 (3_72_1_1)	1glm-d1glm (1_73_1_1)
184	1dkza-d1dkza	5_17_1_1	215	1	0	0	0	0	0	0	1vsga-d1vsga (6_3_1_1)	2liga-d2liga (1_23_2_1)	1aep-d1aep (1_49_1_1)	1lpe-d1lpe (1_23_1_1)	1occc-d1occc1 (6_5_1_1)	1bbha-d1bbha (1_23_3_2)	1zymb-d1zyma (3_5_1_2)	1fps-d1fps (1_91_1_1)	1gsea-d1gsea1 (1_38_1_1)
185	1dlc-d1dlc_1	2_13_1_2	145	2	1	1	0	0	0	0	1ciy-d1ciy_1 (2_13_1_2)	1bgl-d1bgla1 (2_1_3_1)	3nn9-d3nn9 (2_45_1_1)	1bgl-d1bgla2 (2_1_3_1)	1dar-d1dar_1 (2_29_3_1)	1kiqa-d1vfba (2_1_1_1)	1cdaa-d1aly (2_17_1_1)	1tnfa-d1tnfa (2_17_1_1)	1nsdb-d1nsca (2_45_1_1)
186	1dlc-d1dlc_2	2_53_2_1	210	2	1	1	0	0	0	0	1ciy-d1ciy_2 (2_53_2_1)	1nsdb-d1nsca (2_45_1_1)	1ggt-d1ggta2 (2_1_4_1)	1cpn-d1cpn (2_19_1_2)	1eur-d1eur (2_45_1_1)	1bbpa-d1bbpa (2_41_1_1)	1vmoa-d1vmoa (2_53_1_1)	1pex-d1pex (2_44_1_1)	1gof-d1gof_3 (2_46_1_1)
187	1dlc-d1dlc_3	6_1_3_1	229	2	1	1	0	0	0	1	1ciy-d1ciy_3 (6_1_3_1)	1occa-d1occa1 (6_5_1_1)	1mmob-d1mmob (1_24_1_2)	1occc-d1occc1 (6_5_1_1)	1cola-d1cola (6_1_1_1)	1sly-d1sly_1 (1_84_4_1)	1afra-d1afra (1_24_1_2)	2liga-d2liga (1_23_2_1)	1mtyd-d1mmod (1_24_1_2)
188	1dlha-d1dlha1	2_1_1_2	101	1	0	0	1	1	0	0	1dar-d1dar_1 (2_29_3_1)	1ospo-d1ospo (2_52_1_1)	3mdsa-d1mnga2 (4_25_1_1)	1pgs-d1pgs_1 (2_11_1_1)	1taha-d1tahb (3_50_1_8)	1nsdb-d1nsca (2_45_1_1)	1clc-d1clc_2 (2_1_1_5)	1sacb-d1saca (2_19_1_4)	1ngi-d1hpm_2 (3_41_1_1)

189	1dlh-d1dlha2	4_15_1_1	79	1	0	0	0	0	0	0	1vcaa-d1vcaa1 (2_1_1_3)	1dkza-d1dkza (5_17_1_1)	1tnra-d1tnra (2_17_1_1)	1obpa-d1obpa (2_41_1_1)	3nn9-d3nn9 (2_45_1_1)	2pcd-d2pcda (2_3_3_1)	1mpmb-d1mal (6_7_1_2)	1hpl-d1hpla1 (2_10_2_1)	1ggt-d1ggta4 (4_3_1_2)
190	1dmc-d1dmc	7_36_1_1	31	1	0	0	0	0	0	0	1gln-d1gln_2 (3_15_1_1)	3mdsa-d1mnga2 (4_25_1_1)	1etd-d1etd (1_4_3_9)	1eft-d1eft_3 (3_25_1_3)	1wgcb-d1wgta4 (7_3_1_1)	1fd2-d1fd2 (4_33_1_2)	2abk-d2abk (1_66_1_1)	1wgta-d1wgta2 (7_3_1_1)	1broa-d1broa (3_50_1_5)
191	1dnpa-d1dnpa1	1_69_1_1	269	1	0	0	0	0	0	0	1ciy-d1ciy_3 (6_1_3_1)	2myd-d1mbd (1_1_1_1)	1hvd-d1hvd (1_51_1_1)	1mmob-d1mmob (1_24_1_2)	1sly-d1sly_1 (1_84_4_1)	1cpca-d1cpca (1_1_1_2)	1baba-d1baba (1_1_1_1)	1zymb-d1zyma (3_5_1_2)	1mtyg-d1mmog (1_22_1_1)
192	1dnpa-d1dnpa2	3_17_1_1	200	1	0	0	0	0	0	0	2lbp-d2lbp (3_72_1_1)	1tlfa-d1tlfa (3_72_1_1)	2dri-d2dri (3_72_1_1)	1pnr-d1pnra2 (3_72_1_1)	1nai-d1xel (3_19_1_2)	1tml-d1tml (3_2_1_1)	1pea-d1pea (3_72_1_1)	1gca-d1gca (3_72_1_1)	1rvva-d1rvva (3_9_1_1)
193	1dona-d1dona	4_7_1_1	76	3	1	1	0	0	0	0	1napd-d1napa (4_7_1_1)	1huma-d1huma (4_7_1_1)	1btn-d1btn (2_37_1_1)	1fica-d3fib (4_98_1_1)	1mrj-d1mrj (4_94_1_1)	1gdob-d1gdoa (4_88_1_1)	1kte-d1kte (3_33_1_1)	1orda-d1orda2 (3_48_1_4)	1tsra-d1tupa (2_2_3_1)
194	1dora-d1dora	3_1_7_1	311	3	0	0	0	0	1	1	1igs-d1igs (3_1_8_1)	2chr-d2chr_1 (3_1_6_2)	2mnr-d2mnr_1 (3_1_6_2)	2dri-d2dri (3_72_1_1)	1nal1-d1nal1 (3_1_3_1)	1dhpa-d1dhpa (3_1_3_1)	1dik-d1dik_1 (3_1_9_2)	1tlfa-d1tlfa (3_72_1_1)	2gpb-d1gpb (3_68_1_2)
195	1dppc-d1dppa	3_73_1_1	507	8	1	1	0	0	0	0	1jeva-d2olba (3_73_1_1)	1rvva-d1rvva (3_9_1_1)	1reqb-d1reqb2 (3_13_5_2)	1ahia-d1fmca (3_19_1_2)	1wsyb-d1wsyb (3_59_1_1)	3chy-d3chy (3_13_2_1)	2dri-d2dri (3_72_1_1)	1nfp-d1nfp (3_1_13_2)	1tlfa-d1tlfa (3_72_1_1)
196	1dpra-d1dpra1	1_4_3_11	62	1	0	0	0	1	1	1	1pdn-d1pdnc (1_4_1_4)	2wrpr-d2wrpr (1_78_1_1)	1vin-d1vin_2 (1_59_1_1)	1bia-d1bia_1 (1_4_3_1)	1cgpb-d1cgpa1 (1_4_3_4)	1rnl-d1rnl_1 (1_31_1_2)	1octc-d1octc2 (1_30_1_1)	1hddc-d1enh (1_4_1_1)	1gtma-d1gtma1 (3_19_1_7)
197	1dpra-d1dpra2	1_61_1_1	72	1	0	0	0	0	0	0	1pgo-d2pgd_1 (1_71_1_1)	1occc-d1occc1 (6_5_1_1)	2gpb-d1gpb (3_68_1_2)	1mmob-d1mmob (1_24_1_2)	1glqa-d1glqa1 (1_38_1_1)	2lhb-d2lhb (1_1_1_1)	1lef-d1lefa (1_20_1_1)	1xsm-d1xsm (1_24_1_2)	1vin-d1vin_1 (1_59_1_1)
198	1dsba-d1dsba1	1_37_1_1	64	1	0	0	0	0	0	0	1cola-d1cola (6_1_1_1)	1occc-d1occc1 (6_5_1_1)	1hvd-d1hvd (1_51_1_1)	2myd-d1mbd (1_1_1_1)	1octc-d1octc2 (1_30_1_1)	2scpa-d2scpa (1_34_1_5)	2wrpr-d2wrpr (1_78_1_1)	1flp-d1flp (1_1_1_1)	1chka-d1chka (4_2_1_6)
199	1dud-d1dupa	2_60_3_1	136	2	1	1	0	0	0	0	1duta-d1duta (2_60_3_1)	1kcw-d1kcw_5 (2_5_1_3)	2ncm-d2ncm (2_1_1_4)	1bvp1-d1bvp12 (2_14_1_1)	1kcw-d1kcw_4 (2_5_1_3)	1lcl-d1lcl (2_19_1_3)	1asqa-d1aoza2 (2_5_1_3)	1kcw-d1kcw_1 (2_5_1_3)	2sil-d2sil (2_45_1_1)
200	1duta-d1duta	2_60_3_1	117	2	1	1	0	0	1	1	1dud-d1dupa (2_60_3_1)	1tul-d1tul (2_60_4_1)	1p03a-d2alp (2_31_1_1)	1wba-d1wba (2_28_3_1)	1krcb-d2kaub (2_60_2_1)	2trcb-d2trcb (2_46_3_1)	2bpa1-d2bpa1 (2_8_1_1)	1tiig-d1tiid (2_26_2_1)	1mpmb-d1mal (6_7_1_2)
201	1dvh-d1dvh	1_3_1_1	79	7	0	0	1	1	0	0	1fcdc-d1fcdc1 (1_3_1_3)	1etpa-d1etpa2 (1_3_1_3)	1etpa-d1etpa1 (1_3_1_3)	1fcdc-d1fcdc2 (1_3_1_3)	1cem-d1cem (1_73_1_2)	2mtac-d2mtac (1_3_1_1)	1hvd-d1hvd (1_51_1_1)	1dnpa-d1dnpa1 (1_69_1_1)	1taha-d1tahb (3_50_1_8)
202	1dxy-d1dxy_1	3_13_9_1	100	1	0	0	0	0	0	0	1bncb-d1bnca2 (3_20_1_1)	1igs-d1igs (3_1_8_1)	1nai-d1xel (3_19_1_2)	1oiba-d1pbp (3_73_1_1)	2bnh-d1dfji (3_7_1_1)	3fx2-d2fx2 (3_13_4_1)	1pbn-d1pbn (3_52_1_1)	1ghr-d1ghr (3_1_1_3)	1aco-d1aco_1 (3_5_2_1)
203	1dxy-d1dxy_2	3_19_1_4	230	4	1	1	1	1	0	0	1psda-d1psda2 (3_19_1_4)	1gdha-d1gdha2 (3_19_1_4)	2naca-d2naca2 (3_19_1_4)	1gtma-d1gtma1 (3_19_1_7)	1leha-d1leha1 (3_19_1_7)	1hrda-d1hrda1 (3_19_1_7)	1lst-d1lst (3_73_1_1)	1ggga-d1ggga (3_73_1_1)	1onea-d1ebha1 (3_1_6_1)
204	1dyna-d1dyna	2_37_1_1	113	5	0	1	0	1	0	0	1msk-d1msk (4_99_1_1)	1pls-d1pls (2_37_1_1)	1pms-d1pms (2_37_1_1)	1han-d1han_2 (4_20_1_3)	1pnr-d1pnra2 (3_72_1_1)	1cpn-d1cpn (2_19_1_2)	1shca-d1shca (2_37_1_2)	1btn-d1btn (2_37_1_1)	1han-d1han_1 (4_20_1_3)
205	1eac-d1eaf	3_30_1_1	243	1	0	0	0	0	0	0	1gtra-d1gtra2 (3_15_1_1)	1lxta-d3pmga4 (4_74_2_1)	1tlfa-d1tlfa (3_72_1_1)	1coy-d1coy_1 (3_4_1_2)	1sbp-d1sbp (3_73_1_1)	1vin-d1vin_1 (1_59_1_1)	2ms2a-d2ms2a (4_45_1_1)	2anhb-d2anha (3_56_1_1)	1nfp-d1nfp (3_1_13_2)
206	1eal-d1eal	2_41_1_2	127	3	1	1	0	1	0	0	2hmb-d1hms (2_41_1_2)	1ure-d1ifc (2_41_1_2)	1ospo-d1ospo (2_52_1_1)	1bbpa-d1bbpa (2_41_1_1)	1cyx-d1cyx (2_5_1_2)	1prn-d1prn (6_7_1_1)	1tlk-d1tlk (2_1_1_4)	1obpa-d1obpa (2_41_1_1)	1mpmb-d1mal (6_7_1_2)
207	1eceb-d1ecea	3_1_1_3	358	5	1	1	1	1	1	1	1edg-d1edg (3_1_1_3)	1edt-d1edt (3_1_1_5)	2ebn-d2ebn (3_1_1_5)	2ada-d1add (3_1_2_1)	1cbg-d1cbg (3_1_1_4)	1qba-d1qba_3 (3_1_1_6)	4xis-d2xis (3_1_12_1)	1xyza-d1xyza (3_1_1_3)	1bgl-d1bgla5 (3_1_1_3)
208	1ecia-d1ecia	1_29_1_1	37	1	0	0	0	0	0	0	1hlb-d1hlb (1_1_1_1)	1mtyd-d1mmod (1_24_1_2)	1aep-d1aep (1_49_1_1)	1ilk-d1ilk (1_25_1_3)	1cpca-d1cpca (1_1_1_2)	1xsm-d1xsm (1_24_1_2)	1fox-d1fow (1_4_4_1)	1emd-d2cmd_2 (4_92_1_1)	1hvd-d1hvd (1_51_1_1)
209	1ecma-d1ecma	1_93_1_1	91	1	0	0	0	0	0	0	1ciy-d1ciy_3 (6_1_3_1)	2liga-d2liga (1_23_2_1)	1aep-d1aep (1_49_1_1)	2myd-d1mbd (1_1_1_1)	1flp-d1flp (1_1_1_1)	1lh7-d1lh1 (1_1_1_1)	2hbg-d1hbg (1_1_1_1)	1dkza-d1dkza (5_17_1_1)	1occc-d1occc1 (6_5_1_1)
210	1ecpa-d1ecpa	3_52_1_1	237	2	1	1	0	0	0	0	1pbn-d1pbn (3_52_1_1)	1tlfa-d1tlfa (3_72_1_1)	2dri-d2dri (3_72_1_1)	1lyla-d1lyla2 (4_59_1_1)	2ada-d1add (3_1_2_1)	1ahia-d1fmca (3_19_1_2)	1dora-d1dora (3_1_7_1)	1vid-d1vid (3_47_1_1)	1nfp-d1nfp (3_1_13_2)
211	1edg-d1edg	3_1_1_3	380	5	1	1	1	1	0	1	1eceb-d1ecea (3_1_1_3)	1qba-d1qba_3 (3_1_1_6)	1cbg-d1cbg (3_1_1_4)	1cb2b-d1cb2a (3_2_1_1)	4xis-d2xis (3_1_12_1)	1fbaa-d1fbaa (3_1_3_1)	1tml-d1tml (3_2_1_1)	1dhr-d1dhr (3_19_1_2)	1xyza-d1xyza (3_1_1_3)
212	1edt-d1edt	3_1_1_5	265	3	1	1	1	1	1	1	2ebn-d2ebn (3_1_1_5)	1eceb-d1ecea (3_1_1_3)	1dora-d1dora (3_1_7_1)	1gym-d1gym (3_1_15_2)	1oya-d1oya (3_1_7_1)	1xyza-d1xyza (3_1_1_3)	1dnpa-d1dnpa2 (3_17_1_1)	1tml-d1tml (3_2_1_1)	1llo-d1hvq (3_1_1_5)
213	1efr-d1bmfa2	2_33_1_1	71	2	1	1	0	0	0	0	1efrd-d1bmfd2 (2_33_1_1)	1dar-d1dar_1 (2_29_3_1)	1tuia-d1eft_1 (2_29_3_1)	1iyu-d1iyu (2_59_1_1)	1bll-d1lam_2 (3_52_3_3)	1kxa-d2snv (2_31_1_3)	1ospo-d1ospo (2_52_1_1)	2aaa-d2aaa_1 (2_48_1_1)	1brnl-d1brnl (4_1_1_1)
214	1efrd-d1bmfd2	2_33_1_1	73	2	1	1	0	0	0	0	1efr-d1bmfa2 (2_33_1_1)	1dar-d1dar_1 (2_29_3_1)	1p03a-d2alp (2_31_1_1)	1tuia-d1eft_1 (2_29_3_1)	1dlc-d1dlc_1 (2_13_1_2)	1qila-d1tssa1 (2_26_2_2)	2aaa-d2aaa_1 (2_48_1_1)	1eac-d1eaf (3_30_1_1)	1ospo-d1ospo (2_52_1_1)
215	1eft-d1eft_2	2_30_1_1	93	1	0	0	0	0	0	0	1p03a-d2alp (2_31_1_1)	1ospo-d1ospo (2_52_1_1)	1asya-d1asya1 (2_26_4_1)	1clc-d1clc_2 (2_1_1_5)	1iob-d1i1b (2_28_1_2)	1yhb-d1yhb (2_26_4_7)	1vcaa-d1vcaa1 (2_1_1_3)	1tnra-d1tnra (2_17_1_1)	1pgs-d1pgs_2 (2_11_1_1)
216	1eft-d1eft_3	3_25_1_3	212	2	0	1	0	0	0	0	1fbaa-d1fbaa (3_1_3_1)	1crr-d5p21 (3_25_1_3)	2mnr-d2mnr_1 (3_1_6_2)	1nal1-d1nal1 (3_1_3_1)	1fnc-d1fnb_2 (3_14_1_1)	1dora-d1dora (3_1_7_1)	1gky-d1gky (3_25_1_1)	1tlfa-d1tlfa (3_72_1_1)	1mioa-d1mioa (3_67_1_1)
217	1egr-d1ego	3_33_1_1	85	4	0	0	1	1	0	0	2glra-d1glqa2 (3_33_1_5)	3gsta-d2gsta2 (3_33_1_5)	1gsea-d1gsea2 (3_33_1_5)	1cowe-d1bmfd3 (3_25_1_6)	1dhpa-d1dhpa (3_1_3_1)	1art-d1art (3_48_1_1)	1mek-d1mek (3_33_1_2)	1aba-d1aba (3_33_1_1)	1idm-d1idm (3_57_1_1)
218	1ehs-d1ehs	7_2_1_1	48	1	0	0	0	0	0	0	1pbwa-d1pbwa (1_83_1_1)	1fps-d1fps (1_91_1_1)	1sra-d1sra (1_34_1_3)	2dkb-d2dkb (3_48_1_3)	3mdsa-d1mnga2 (4_25_1_1)	1aab-d1aab (1_20_1_1)	1pbn-d1pbn (3_52_1_1)	1occa-d1occa1 (6_5_1_1)	1pea-d1pea (3_72_1_1)
219	1emd-d2cmd_1	3_19_1_5	145	4	1	1	1	1	0	0	6ldh-d1ldm_1 (3_19_1_5)	1llda-d1llda1 (3_19_1_5)	1hlpa-d1hlpa1 (3_19_1_5)	1pgn-d2pgd_2 (3_19_1_6)	1hdca-d1hdca (3_19_1_2)	1gca-d1gca (3_72_1_1)	1rvva-d1rvva (3_9_1_1)	1mml-d1mml (5_9_1_2)	1ahia-d1fmca (3_19_1_2)
220	1emd-d2cmd_2	4_92_1_1	167	5	1	1	0	0	0	0	1bdmb-d1bdma2 (4_92_1_1)	6ldh-d1ldm_2 (4_92_1_1)	1hlpa-d1hlpa2 (4_92_1_1)	1llda-d1llda2 (4_92_1_1)	4xis-d2xis (3_1_12_1)	1lbd-d1lbd (1_87_1_1)	1hrda-d1hrda1 (3_19_1_7)	1hdca-d1hdca (3_19_1_2)	1fbaa-d1fbaa (3_1_3_1)
221	1emo-d1emo_1	7_3_9_1	43	7	1	1	0	0	0	0	1hcgb-d1hcgb (7_3_9_1)	1bcpb-d1prtb1 (2_26_2_1)	1gph1-d1gph12 (4_88_1_1)	2plda-d2plda (4_51_1_1)	1sso-d1sso (4_9_1_1)	1asob-d1aoza3 (2_5_1_3)	1tul-d1tul (2_60_4_1)	1occb-d1occb1 (2_5_1_2)	1fwp-d1fwp (4_33_18_1)
222	1emo-d1emo_2	7_3_9_1	39	7	0	0	0	0	0	0	2cpl-d2cpl (2_43_1_1)	1pht-d1pht (2_21_2_1)	4fua-d1fua (3_55_1_1)	1bgl-d1bgla5 (3_1_1_3)	2mtal-d2bbkl (7_19_1_1)	1wba-d1wba (2_28_3_1)	1gur-d1gur (7_3_4_1)	1chd-d1chd (3_27_1_1)	2pia-d2pia_3 (4_12_4_1)
223	1eny-d1eny	3_19_1_2	268	6	1	1	0	0	0	0	1ahia-d1fmca (3_19_1_2)	1cyda-d1cyda (3_19_1_2)	1hdca-d1hdca (3_19_1_2)	1dhr-d1dhr (3_19_1_2)	1nai-d1xel (3_19_1_2)	1gca-d1gca (3_72_1_1)	1fbaa-d1fbaa (3_1_3_1)	2dri-d2dri (3_72_1_1)	1cb2b-d1cb2a (3_2_1_1)
224	1eph-d1egf	7_3_9_1	53	7	1	1	0	0	0	0	4tgf-d2tgf (7_3_9_1)	1mtyd-d1mmod (1_24_1_2)	1ctn-d1ctn_3 (4_19_3_1)	1dlc-d1dlc_2 (2_53_2_1)	1gof-d1gof_3 (2_46_1_1)	1ure-d1ifc (2_41_1_2)	1oacb-d1oaca3 (4_14_2_1)	1nsdb-d1nsca (2_45_1_1)	1clc-d1clc_2 (2_1_1_5)
225	1erd-d1erd	1_10_1_1	40	3	0	0	0	0	0	0	1gbs-d1gbs (4_2_1_4)	1sfe-d1sfe_1 (1_4_2_1)	1dhpa-d1dhpa (3_1_3_1)	2liga-d2liga (1_23_2_1)	2baa-d2baa (4_2_1_1)	1lbu-d1lbu_1 (1_19_1_1)	2erl-d1erc (1_10_1_1)	1vin-d1vin_2 (1_59_1_1)	1sly-d1sly_1 (1_84_4_1)
226	1eri-d1eria	3_38_1_1	261	1	0	0	0	0	0	0	1bll-d1lam_2 (3_52_3_3)	1tys-d1tys (4_63_1_1)	1fica-d3fib (4_98_1_1)	1scb-d1csee (3_28_1_1)	1nsdb-d1nsca (2_45_1_1)	1htt-d1htta1 (3_37_1_1)	1taha-d1tahb (3_50_1_8)	1acf-d1acf (4_61_1_1)	1osa-d1osa (1_34_1_5)
227	1erp-d1erp	1_10_1_1	38	3	1	1	0	0	0	0	2erl-d1erc (1_10_1_1)	2abk-d2abk (1_66_1_1)	1sly-d1sly_1 (1_84_4_1)	2tct-d2tct_1 (1_31_1_1)	2cpo-d1cpo_1 (1_34_2_1)	1beo-d1beo (1_96_1_1)	1rpl-d1rpl (5_10_1_1)	1rcd-d1rcd (1_24_1_1)	4xis-d2xis (3_1_12_1)
228	1esd-d1esc	3_13_8_1	302	1	0	0	0	0	0	0	1bdmb-d1bdma2 (4_92_1_1)	1tml-d1tml (3_2_1_1)	1bya-d1byb (3_1_1_2)	2sas-d2sas (1_34_1_5)	1dik-d1dik_1 (3_1_9_2)	1rcd-d1rcd (1_24_1_1)	1nbaa-d1nbaa (3_22_1_1)	1prcl-d1prcl1 (6_5_1_1)	1gym-d1gym (3_1_15_2)
229	1esl-d1esl_1	4_97_1_1	118	2	1	1	0	0	0	0	1rtm2-d1rtm12 (4_97_1_1)	1pii-d1pii_2 (3_1_8_1)	1oaca-d1oaca2 (4_14_2_1)	1vsga-d1vsga (6_3_1_1)	1prhb-d1prha1 (1_65_1_2)	1nsj-d1nsj (3_1_8_1)	2tmda-d2tmda1 (3_1_7_1)	3mdsa-d1mnga2 (4_25_1_1)	2abk-d2abk (1_66_1_1)
230	1esl-d1esl_2	7_3_9_1	39	7	0	0	0	0	0	0	2pnb-d2pnb (4_51_1_1)	1hpca-d1htp (2_59_1_1)	1hcd-d1hcd (2_28_4_1)	1gen-d1gen (2_44_1_1)	4kbpa-d4kbpa2 (4_91_1_1)	1pex-d1pex (2_44_1_1)	1xxca-d1xxaa (4_38_2_1)	1hpm-d1hpm_1 (3_41_1_1)	2tmda-d2tmda2 (3_4_1_1)
231	1etd-d1etd	1_4_3_9	106	2	0	0	0	0	0	0	1fcdc-d1fcdc1 (1_3_1_3)	1prr-d1prr_1 (2_9_1_2)	1phe-d1phb (1_75_1_1)	1vjs-d1bplb1 (2_48_1_1)	1pbn-d1pbn (3_52_1_1)	1nsj-d1nsj (3_1_8_1)	1mioa-d1mioa (3_67_1_1)	1akl-d1kapp2 (4_50_1_6)	1oiba-d1pbp (3_73_1_1)
232	1etpa-d1etpa1	1_3_1_3	92	4	1	1	1	1	0	0	1fcdc-d1fcdc1 (1_3_1_3)	1dvh-d1dvh (1_3_1_1)	1etpa-d1etpa2 (1_3_1_3)	1fcdc-d1fcdc2 (1_3_1_3)	2mtac-d2mtac (1_3_1_1)	1sfta-d1sfta2 (3_1_18_1)	1tml-d1tml (3_2_1_1)	1hrda-d1hrda2 (3_54_1_1)	1occa-d1occa1 (6_5_1_1)
233	1etpa-d1etpa2	1_3_1_3	98	4	1	1	1	1	0	0	1fcdc-d1fcdc1 (1_3_1_3)	1dvh-d1dvh (1_3_1_1)	1fcdc-d1fcdc2 (1_3_1_3)	1etpa-d1etpa1 (1_3_1_3)	2wrpr-d2wrpr (1_78_1_1)	1cem-d1cem (1_73_1_2)	119l-d119l (4_2_1_3)	2aky-d1aky (3_25_1_1)	1tyc-d2ts1_1 (1_54_1_1)
234	1eur-d1eur	2_45_1_1	361	4	1	1	0	0	0	0	2sil-d2sil (2_45_1_1)	3nn9-d3nn9 (2_45_1_1)	2bbkh-d2bbkh (2_46_2_1)	2trcb-d2trcb (2_46_3_1)	1gof-d1gof_3 (2_46_1_1)	1nsdb-d1nsca (2_45_1_1)	1ospo-d1ospo (2_52_1_1)	4aahc-d4aaha (2_47_1_1)	2omf-d2omf (6_7_1_1)
235	1exg-d1exg	2_2_2_1	110	1	0	0	0	0	0	0	1eal-d1eal (2_41_1_2)	1ggt-d1ggta2 (2_1_4_1)	2hmb-d1hms (2_41_1_2)	2pola-d2pola2 (4_76_1_1)	2por-d2por (6_7_1_1)	1ouna-d1ouna (4_14_3_2)	1tnn-d1tnm (2_1_1_4)	4aahc-d4aaha (2_47_1_1)	1seia-d1seia (4_80_1_1)
236	1extb-d1ncfa1	7_22_1_1	60	2	0	0	0	0	0	0	1fna-d1fna (2_1_2_1)	2tmda-d2tmda1 (3_1_7_1)	1chd-d1chd (3_27_1_1)	1dppc-d1dppa (3_73_1_1)	1hcgb-d1hcgb (7_3_9_1)	1p03a-d2alp (2_31_1_1)	1pkp-d1pkp_1 (4_11_1_1)	1qbb-d1qba_2 (2_2_2_3)	1mbt-d1mbb_2 (4_85_1_1)

237	1ezm-d1ezm_1	1_53_1_1	145	1	0	0	0	0	0	0	1flp-d1flp (1_1_1_1)	1dlc-d1dlc_3 (6_1_3_1)	1occa-d1occa1 (6_5_1_1)	1hvd-d1hvd (1_51_1_1)	1itha-d1itha (1_1_1_1)	1lis-d1lis (1_17_1_1)	1cem-d1cem (1_73_1_2)	1gsea-d1gsea1 (1_38_1_1)	1afra-d1afra (1_24_1_2)
238	1ezm-d1ezm_2	4_50_1_2	153	1	0	0	1	1	0	0	1iad-d1ast (4_50_1_4)	1htda-d1atla (4_50_1_5)	1dih-d1dih_2 (4_41_1_2)	1oela-d1grl_2 (3_5_3_1)	1ngi-d1hpm_2 (3_41_1_1)	1xra-d1mxa_2 (4_75_1_1)	1alo-d1alo_7 (4_77_1_1)	1gym-d1gym (3_1_15_2)	1amp-d1amp (3_52_3_4)
239	1faga-d2bmha	1_75_1_1	455	3	1	1	0	0	0	0	1oxa-d1oxa (1_75_1_1)	1phe-d1phb (1_75_1_1)	1occc-d1occc1 (6_5_1_1)	1prcl-d1prcl1 (6_5_1_1)	1afra-d1afra (1_24_1_2)	1mtyd-d1mmod (1_24_1_2)	1ciy-d1ciy_3 (6_1_3_1)	1dlc-d1dlc_3 (6_1_3_1)	1occa-d1occa1 (6_5_1_1)
240	1faq-d1faq	7_38_1_1	52	2	1	1	0	0	0	0	1ptq-d1ptq (7_38_1_1)	1dik-d1dik_2 (3_5_1_1)	1chc-d1chc (7_35_1_1)	1ose-d1ppi_1 (2_48_1_1)	1prch-d1prch1 (2_27_1_1)	1adn-d1adn (7_37_1_1)	1gof-d1gof_3 (2_46_1_1)	1aco-d1aco_1 (3_5_2_1)	1rie-d1rie (7_33_1_2)
241	1fbaa-d1fbaa	3_1_3_1	359	3	0	0	0	0	0	1	1tlfa-d1tlfa (3_72_1_1)	1tpfa-d1tpfa (3_1_11_1)	4xis-d2xis (3_1_12_1)	2bnh-d1dfji (3_7_1_1)	1xyza-d1xyza (3_1_1_3)	1eft-d1eft_3 (3_25_1_3)	2ebn-d2ebn (3_1_1_5)	2dri-d2dri (3_72_1_1)	1miob-d1miob (3_67_1_1)
242	1fbr-d1fbr_1	7_40_1_1	46	3	1	1	0	0	0	0	1fbr-d1fbr_2 (7_40_1_1)	1obpa-d1obpa (2_41_1_1)	2hnp-d2hnp (3_32_1_2)	2bbkh-d2bbkh (2_46_2_1)	1eft-d1eft_2 (2_30_1_1)	1tsra-d1tupa (2_2_3_1)	1gof-d1gof_3 (2_46_1_1)	3pte-d3pte (5_4_1_1)	1iob-d1i1b (2_28_1_2)
243	1fbr-d1fbr_2	7_40_1_1	47	3	1	1	0	0	0	0	1fbr-d1fbr_1 (7_40_1_1)	1tpn-d1tpm (7_40_1_1)	2hmb-d1hms (2_41_1_2)	1bvp1-d1bvp12 (2_14_1_1)	2pia-d2pia_1 (2_29_1_2)	1gof-d1gof_3 (2_46_1_1)	1geua-d1gesa2 (3_4_1_4)	2hnp-d2hnp (3_32_1_2)	1gog-d1gof_1 (2_1_1_5)
244	1fc2c-d1fc2c	1_8_1_1	43	1	0	0	0	0	0	0	3sdha-d3sdha (1_1_1_1)	1lh7-d1lh1 (1_1_1_1)	1scb-d1csee (3_28_1_1)	1prhb-d1prha1 (1_65_1_2)	1cola-d1cola (6_1_1_1)	1hlb-d1hlb (1_1_1_1)	1chra-d2chr_2 (4_31_1_1)	1xsm-d1xsm (1_24_1_2)	1eft-d1eft_3 (3_25_1_3)
245	1fca-d1fca	4_33_1_1	55	1	0	0	1	1	0	0	1fd2-d1fd2 (4_33_1_2)	2fxb-d2fxb (4_33_1_4)	1rof-d1vjw (4_33_1_4)	1fxra-d1fxra (4_33_1_4)	1dkza-d1dkza (5_17_1_1)	1llda-d1llda2 (4_92_1_1)	1emo-d1emo_1 (7_3_9_1)	6ldh-d1ldm_2 (4_92_1_1)	1mcti-d1mcti (7_3_2_1)
246	1fcda-d1fcda3	4_46_1_1	74	5	0	0	0	0	0	0	2ms2a-d2ms2a (4_45_1_1)	1prn-d1prn (6_7_1_1)	1ngi-d1hpm_2 (3_41_1_1)	1btl-d1btl (5_4_1_1)	1poba-d1poa (1_95_1_2)	1gtma-d1gtma2 (3_54_1_1)	1hjra-d1hjra (3_41_3_6)	1chma-d1chma2 (4_72_1_1)	2por-d2por (6_7_1_1)
247	1fcdb-d1fcda2	3_4_1_4	141	6	0	1	0	0	0	0	1yasa-d1yasa (3_50_1_10)	1lbt-d1tca (3_50_1_7)	1geua-d1gesa2 (3_4_1_4)	1art-d1art (3_48_1_1)	1pyda-d1pvda3 (3_24_1_1)	1febb-d2tpra2 (3_4_1_4)	1gln-d1gln_2 (3_15_1_1)	1emd-d2cmd_1 (3_19_1_5)	1eny-d1eny (3_19_1_2)
248	1fcdc-d1fcdc1	1_3_1_3	80	4	0	1	1	1	0	0	1dvh-d1dvh (1_3_1_1)	1etpa-d1etpa2 (1_3_1_3)	1etpa-d1etpa1 (1_3_1_3)	1fcdc-d1fcdc2 (1_3_1_3)	2mtac-d2mtac (1_3_1_1)	2wrpr-d2wrpr (1_78_1_1)	1gtra-d1gtra2 (3_15_1_1)	119l-d119l (4_2_1_3)	1igna-d1igna1 (1_4_1_5)
249	1fcdc-d1fcdc2	1_3_1_3	94	4	1	1	1	1	0	0	1fcdc-d1fcdc1 (1_3_1_3)	1etpa-d1etpa2 (1_3_1_3)	2mtac-d2mtac (1_3_1_1)	1dvh-d1dvh (1_3_1_1)	1etpa-d1etpa1 (1_3_1_3)	1pgo-d2pgd_1 (1_71_1_1)	1cpca-d1cpca (1_1_1_2)	5cyt-d5cytr (1_3_1_1)	1llib-d1llia (1_30_1_2)
250	1fct-d1fct	8_31_1_1	32	1	0	0	0	0	0	0	1mtyd-d1mmod (1_24_1_2)	1vpu-d1vpu (7_25_1_1)	1tfb-d1vola2 (1_59_1_2)	1cpcb-d1cpcb (1_1_1_2)	1alla-d1alla (1_1_1_2)	1cpca-d1cpca (1_1_1_2)	1pmi-d1pmi (2_58_2_1)	1cof-d1ahq (4_60_1_2)	1hxp-d1hxpa2 (4_10_1_2)
251	1fd2-d1fd2	4_33_1_2	106	1	0	0	1	1	0	0	1fca-d1fca (4_33_1_1)	2fxb-d2fxb (4_33_1_4)	2cpo-d1cpo_2 (1_34_2_1)	1eri-d1eria (3_38_1_1)	1fxra-d1fxra (4_33_1_4)	1edt-d1edt (3_1_1_5)	4kbpa-d4kbpa2 (4_91_1_1)	1smna-d1smna (4_71_1_1)	2lbp-d2lbp (3_72_1_1)
252	1fead-d2tpra3	4_46_1_1	125	5	1	1	0	0	0	0	1gesa-d1gesa3 (4_46_1_1)	1lvl-d1lvl_3 (4_46_1_1)	2npx-d1nhp_3 (4_46_1_1)	1btl-d1btl (5_4_1_1)	1alo-d1alo_4 (4_77_1_1)	1xoa-d2trxa (3_33_1_1)	1poh-d1poh (4_52_1_1)	1gtma-d1gtma2 (3_54_1_1)	4aahc-d4aaha (2_47_1_1)
253	1febb-d2tpra2	3_4_1_4	117	6	1	1	1	1	0	0	1geua-d1gesa2 (3_4_1_4)	1nhq-d1nhp_2 (3_4_1_4)	1tde-d1tde_2 (3_4_1_4)	1lvl-d1lvl_2 (3_4_1_4)	2tmda-d2tmda2 (3_4_1_1)	1fcdb-d1fcda2 (3_4_1_4)	1coy-d1coy_1 (3_4_1_2)	1lbu-d1lbu_1 (1_19_1_1)	1pgn-d2pgd_2 (3_19_1_6)
254	1fem-d1hbp	2_41_1_1	174	4	1	1	0	0	0	0	1bbpa-d1bbpa (2_41_1_1)	1bebb-d1beba (2_41_1_1)	1obpa-d1obpa (2_41_1_1)	1ospo-d1ospo (2_52_1_1)	1mpmb-d1mal (6_7_1_2)	2sil-d2sil (2_45_1_1)	2avia-d2avia (2_42_1_1)	1bgl-d1bgla1 (2_1_3_1)	2vik-d2vik (4_60_1_1)
255	1fica-d3fib	4_98_1_1	249	1	0	0	0	0	0	0	1eri-d1eria (3_38_1_1)	1ospo-d1ospo (2_52_1_1)	1fem-d1hbp (2_41_1_1)	1ggt-d1ggta4 (4_3_1_2)	1prn-d1prn (6_7_1_1)	1gdob-d1gdoa (4_88_1_1)	1dona-d1dona (4_7_1_1)	1hpl-d1hpla1 (2_10_2_1)	2pola-d2pola3 (4_76_1_1)
256	1fipa-d1fipa	1_76_1_1	73	1	0	0	0	0	0	0	1cola-d1cola (6_1_1_1)	2wrpr-d2wrpr (1_78_1_1)	1cpcb-d1cpcb (1_1_1_2)	1bfra-d1bcfa (1_24_1_1)	1hvd-d1hvd (1_51_1_1)	119l-d119l (4_2_1_3)	1gtma-d1gtma1 (3_19_1_7)	1ciy-d1ciy_3 (6_1_3_1)	1asu-d1asu (3_41_3_2)
257	1fiva-d1fiva	2_34_1_1	113	3	1	1	0	0	0	0	2mipa-d1idaa (2_34_1_1)	1fmb-d1fmb (2_34_1_1)	1iob-d1i1b (2_28_1_2)	2sil-d2sil (2_45_1_1)	2gpb-d1gpb (3_68_1_2)	1tnra-d1tnra (2_17_1_1)	1eri-d1eria (3_38_1_1)	1p03a-d2alp (2_31_1_1)	1obpa-d1obpa (2_41_1_1)
258	1fjm-d1fjma	4_91_1_2	294	1	0	0	0	0	0	0	1lucb-d1lucb (3_1_13_1)	1broa-d1broa (3_50_1_5)	1wsyb-d1wsyb (3_59_1_1)	1kte-d1kte (3_33_1_1)	1tml-d1tml (3_2_1_1)	2chr-d2chr_1 (3_1_6_2)	1hora-d1deaa (3_23_1_1)	2dri-d2dri (3_72_1_1)	1raca-d1raaa1 (3_58_1_1)
259	1fkg-d1fkd	4_19_1_1	107	1	0	0	0	0	0	0	1prch-d1prch1 (2_27_1_1)	1xva-d1xvaa (3_47_1_2)	1btl-d1btl (5_4_1_1)	1noa-d1noa (2_1_6_1)	1tsra-d1tupa (2_2_3_1)	1eceb-d1ecea (3_1_1_3)	1coy-d1coy_1 (3_4_1_2)	1clc-d1clc_2 (2_1_1_5)	1ciy-d1ciy_2 (2_53_2_1)
260	1fld-d5nul	3_13_4_1	138	3	1	1	0	0	0	0	3fx2-d2fx2 (3_13_4_1)	1rcf-d1rcf (3_13_4_1)	1xyza-d1xyza (3_1_1_3)	1art-d1art (3_48_1_1)	1bncb-d1bnca2 (3_20_1_1)	1wsyb-d1wsyb (3_59_1_1)	1fbaa-d1fbaa (3_1_3_1)	1pgn-d2pgd_2 (3_19_1_6)	1crr-d5p21 (3_25_1_3)
261	1flei-d1flei	7_3_12_1	47	1	0	0	0	0	0	0	1coy-d1coy_1 (3_4_1_2)	1akl-d1kapp2 (4_50_1_6)	1eac-d1eaf (3_30_1_1)	2pec-d2pec (2_56_1_1)	1aocb-d1aoca (7_14_1_5)	1miob-d1miob (3_67_1_1)	1mbt-d1mbb_2 (4_85_1_1)	1dnpa-d1dnpa2 (3_17_1_1)	1gen-d1gen (2_44_1_1)
262	1flp-d1flp	1_1_1_1	142	10	1	1	0	0	0	0	3sdha-d3sdha (1_1_1_1)	1mba-d1mba (1_1_1_1)	2lhb-d2lhb (1_1_1_1)	1itha-d1itha (1_1_1_1)	1hlb-d1hlb (1_1_1_1)	2hbg-d1hbg (1_1_1_1)	1lh7-d1lh1 (1_1_1_1)	1baba-d1baba (1_1_1_1)	2myd-d1mbd (1_1_1_1)
263	1fmb-d1fmb	2_34_1_1	104	3	1	1	0	0	0	0	2mipa-d1idaa (2_34_1_1)	1fiva-d1fiva (2_34_1_1)	1dar-d1dar_3 (4_11_1_1)	2gpb-d1gpb (3_68_1_2)	4aahc-d4aaha (2_47_1_1)	1pfsa-d1pfsa (2_26_4_7)	1bdmb-d1bdma2 (4_92_1_1)	1kxa-d2snv (2_31_1_3)	2sil-d2sil (2_45_1_1)
264	1fna-d1fna	2_1_2_1	91	4	1	1	0	0	0	1	1ten-d1ten (2_1_2_1)	1cfb-d1cfb_1 (2_1_2_1)	1nsdb-d1nsca (2_45_1_1)	1lcl-d1lcl (2_19_1_3)	1cpn-d1cpn (2_19_1_2)	3hhrb-d3hhrb2 (2_1_2_1)	1bgl-d1bgla2 (2_1_3_1)	1occb-d1occb1 (2_5_1_2)	1svb-d1svb_2 (6_10_1_1)
265	1fnc-d1fnb_1	2_29_1_1	136	2	1	1	1	1	0	0	1cnf-d2cnd_1 (2_29_1_1)	2pia-d2pia_1 (2_29_1_2)	2pcd-d2pcda (2_3_3_1)	2prd-d2prd (2_26_5_1)	1ospo-d1ospo (2_52_1_1)	2avia-d2avia (2_42_1_1)	1noa-d1noa (2_1_6_1)	1fem-d1hbp (2_41_1_1)	1bgl-d1bgla1 (2_1_3_1)
266	1fnc-d1fnb_2	3_14_1_1	160	2	1	1	1	1	0	0	1cnf-d2cnd_2 (3_14_1_1)	2pia-d2pia_2 (3_14_1_2)	1eft-d1eft_3 (3_25_1_3)	1tkaa-d1trka1 (3_24_1_2)	1pnr-d1pnra2 (3_72_1_1)	1cowe-d1bmfd3 (3_25_1_6)	1miob-d1miob (3_67_1_1)	1gca-d1gca (3_72_1_1)	2dri-d2dri (3_72_1_1)
267	1fose-d1fose	1_97_2_1	60	4	1	1	0	0	0	0	1fosf-d1fosf (1_97_2_1)	2hmgd-d1htmb (6_2_1_1)	1rcd-d1rcd (1_24_1_1)	1lpe-d1lpe (1_23_1_1)	1afra-d1afra (1_24_1_2)	1bfra-d1bcfa (1_24_1_1)	2liga-d2liga (1_23_2_1)	1mtyd-d1mmod (1_24_1_2)	1vsga-d1vsga (6_3_1_1)
268	1fosf-d1fosf	1_97_2_1	57	4	1	1	0	0	0	0	1fose-d1fose (1_97_2_1)	2hmgd-d1htmb (6_2_1_1)	1vsga-d1vsga (6_3_1_1)	1mtyd-d1mmod (1_24_1_2)	1bfra-d1bcfa (1_24_1_1)	1ryt-d1ryt_1 (1_24_1_1)	1dkza-d1dkza (5_17_1_1)	1rpo-d1rpo (1_27_1_1)	1occc-d1occc1 (6_5_1_1)
269	1fox-d1fow	1_4_4_1	76	1	0	0	0	0	0	0	1mbt-d1mbb_2 (4_85_1_1)	1pbn-d1pbn (3_52_1_1)	1asza-d1asya2 (4_59_1_1)	1vsga-d1vsga (6_3_1_1)	1poxa-d1poxa2 (3_24_1_1)	1dlc-d1dlc_3 (6_1_3_1)	1ngi-d1hpm_2 (3_41_1_1)	1nis-d1aco_2 (3_63_1_1)	1rnl-d1rnl_1 (1_31_1_2)
270	1fps-d1fps	1_91_1_1	348	1	0	0	0	0	0	0	1occa-d1occa1 (6_5_1_1)	1mtyd-d1mmod (1_24_1_2)	1occc-d1occc1 (6_5_1_1)	1dlc-d1dlc_3 (6_1_3_1)	1ciy-d1ciy_3 (6_1_3_1)	1sly-d1sly_1 (1_84_4_1)	1afra-d1afra (1_24_1_2)	1xsm-d1xsm (1_24_1_2)	1mrrb-d1riba (1_24_1_2)
271	1frd-d1frd	4_12_4_1	98	3	1	1	0	0	0	0	2pia-d2pia_3 (4_12_4_1)	1psda-d1psda2 (3_19_1_4)	1gdha-d1gdha2 (3_19_1_4)	1jdya-d3pmga3 (3_64_1_1)	1put-d1put (4_12_4_1)	1hpm-d1hpm_1 (3_41_1_1)	1tpfa-d1tpfa (3_1_11_1)	1mmob-d1mmob (1_24_1_2)	1rgs-d1rgs_1 (2_58_3_2)
272	1froa-d1froa	4_20_1_1	176	1	0	0	0	1	0	0	1eur-d1eur (2_45_1_1)	1han-d1han_2 (4_20_1_3)	1han-d1han_1 (4_20_1_3)	1raca-d1raaa1 (3_58_1_1)	1ctt-d1ctt_2 (3_75_1_1)	1tkaa-d1trka1 (3_24_1_2)	1gph1-d1gph12 (4_88_1_1)	1htda-d1atla (4_50_1_5)	1scua-d1scua1 (3_13_3_1)
273	1fwp-d1fwp	4_33_18_1	69	1	0	0	0	0	0	1	1iba-d1iba (4_53_1_1)	1mpd-d1mpb (3_73_1_1)	1dar-d1dar_4 (4_33_12_1)	1psda-d1psda3 (4_33_15_1)	1xxca-d1xxaa (4_38_2_1)	1pdo-d1pdo (3_40_1_1)	1rvva-d1rvva (3_9_1_1)	1svq-d1svr (4_60_1_1)	1ghj-d1ghk (2_59_1_1)
274	1fxra-d1fxra	4_33_1_4	64	3	1	1	0	1	0	0	2fxb-d2fxb (4_33_1_4)	1rof-d1vjw (4_33_1_4)	2csn-d1csn (5_1_1_1)	1amy-d1amy_2 (3_1_1_1)	1tys-d1tys (4_63_1_1)	1fca-d1fca (4_33_1_1)	1juy-d1adea (3_25_1_5)	1opr-d1opr (3_44_1_1)	1fd2-d1fd2 (4_33_1_2)
275	1gal-d1gal_1	3_4_1_2	322	2	1	1	0	1	0	0	1coy-d1coy_1 (3_4_1_2)	1kcw-d1kcw_5 (2_5_1_3)	4mhta-d1hmy (3_47_1_4)	1geua-d1gesa2 (3_4_1_4)	1tde-d1tde_2 (3_4_1_4)	1dxy-d1dxy_2 (3_19_1_4)	1nhq-d1nhp_2 (3_4_1_4)	1pgo-d2pgd_1 (1_71_1_1)	1gof-d1gof_3 (2_46_1_1)
276	1gal-d1gal_2	4_13_1_4	66	1	0	0	0	0	0	0	1occa-d1occa1 (6_5_1_1)	1tkaa-d1trka1 (3_24_1_2)	1mmob-d1mmob (1_24_1_2)	1buca-d1buca1 (1_23_6_1)	1cem-d1cem (1_73_1_2)	1rvva-d1rvva (3_9_1_1)	1glm-d1glm (1_73_1_1)	1occc-d1occc1 (6_5_1_1)	1hula-d1hula (1_25_1_2)
277	1gat-d1gata	7_30_1_1	60	1	0	0	0	0	0	0	1aco-d1aco_1 (3_5_2_1)	3pmga-d3pmga1 (3_64_1_1)	1aba-d1aba (3_33_1_1)	2ebn-d2ebn (3_1_1_5)	1orb-d1orb_2 (3_60_1_1)	1tbrr-d1tbrr2 (7_12_1_1)	1nai-d1xel (3_19_1_2)	1lst-d1lst (3_73_1_1)	1edt-d1edt (3_1_1_5)
278	1gbs-d1gbs	4_2_1_4	185	1	0	0	1	1	0	0	1sly-d1sly_2 (4_2_1_5)	1hvd-d1hvd (1_51_1_1)	1cpca-d1cpca (1_1_1_2)	1ilk-d1ilk (1_25_1_3)	1bdmb-d1bdma2 (4_92_1_1)	1osa-d1osa (1_34_1_5)	1lh7-d1lh1 (1_1_1_1)	1glm-d1glm (1_73_1_1)	1ytn-d1ypta (3_32_1_2)
279	1gca-d1gca	3_72_1_1	309	6	1	1	0	0	0	0	2dri-d2dri (3_72_1_1)	1tlfa-d1tlfa (3_72_1_1)	1pnr-d1pnra2 (3_72_1_1)	2lbp-d2lbp (3_72_1_1)	1pea-d1pea (3_72_1_1)	1miob-d1miob (3_67_1_1)	2chr-d2chr_1 (3_1_6_2)	1taha-d1tahb (3_50_1_8)	1eny-d1eny (3_19_1_2)
280	1gcb-d1gcb	4_3_1_1	452	2	0	0	0	0	0	0	1afra-d1afra (1_24_1_2)	1mrrb-d1riba (1_24_1_2)	1faga-d2bmha (1_75_1_1)	1ciy-d1ciy_3 (6_1_3_1)	1csr-d1csh (1_74_1_1)	1aep-d1aep (1_49_1_1)	1mrj-d1mrj (4_94_1_1)	1prcl-d1prcl1 (6_5_1_1)	1xsm-d1xsm (1_24_1_2)
281	1gcn-d1gcn	8_8_1_1	29	1	0	0	0	0	0	0	1csr-d1csh (1_74_1_1)	1peh-d1peh (8_13_1_1)	2ifo-d2ifo (1_97_3_1)	1taha-d1tahb (3_50_1_8)	2aaa-d2aaa_2 (3_1_1_1)	1hrda-d1hrda2 (3_54_1_1)	1dlc-d1dlc_3 (6_1_3_1)	1clc-d1clc_1 (1_73_1_2)	2scpa-d2scpa (1_34_1_5)
282	1gcs-d4gcr_1	2_9_1_1	85	3	1	1	1	1	0	0	4gcr-d4gcr_2 (2_9_1_1)	1blba-d2bb2_2 (2_9_1_1)	1prr-d1prr_1 (2_9_1_2)	1prs-d1prr_2 (2_9_1_2)	1pls-d1pls (2_37_1_1)	2btfa-d2btfa1 (3_41_1_1)	1cpn-d1cpn (2_19_1_2)	1tys-d1tys (4_63_1_1)	2trcb-d2trcb (2_46_3_1)
283	1gdha-d1gdha2	3_19_1_4	191	4	1	1	1	1	0	0	2naca-d2naca2 (3_19_1_4)	1psda-d1psda2 (3_19_1_4)	1dxy-d1dxy_2 (3_19_1_4)	1leha-d1leha1 (3_19_1_7)	1gtma-d1gtma1 (3_19_1_7)	1hrda-d1hrda1 (3_19_1_7)	1pgn-d2pgd_2 (3_19_1_6)	3btoa-d2ohxa2 (3_19_1_1)	1bncb-d1bnca2 (3_20_1_1)
284	1gdob-d1gdoa	4_88_1_1	238	2	1	1	0	0	0	0	1gph1-d1gph12 (4_88_1_1)	3chy-d3chy (3_13_2_1)	1asu-d1asu (3_41_3_2)	1lylb-d1lyla1 (2_26_4_1)	1gcb-d1gcb (4_3_1_1)	1ecpa-d1ecpa (3_52_1_1)	1slba-d1slaa (2_19_1_3)	1pgo-d2pgd_1 (1_71_1_1)	1cby-d1cby (4_58_1_1)

285	1gdz-d1ak4c	1_57_1_1	145	1	0	0	0	0	0	0	1fps-d1fps (1_91_1_1)	1occc-d1occc1 (6_5_1_1)	1pbwa-d1pbwa (1_83_1_1)	2ada-d1add (3_1_2_1)	1dlc-d1dlc_3 (6_1_3_1)	1sly-d1sly_1 (1_84_4_1)	1mrrb-d1riba (1_24_1_2)	1hlb-d1hlb (1_1_1_1)	1chka-d1chka (4_2_1_6)
286	1gen-d1gen	2_44_1_1	200	2	1	1	0	0	0	0	1pex-d1pex (2_44_1_1)	2trcb-d2trcb (2_46_3_1)	3nn9-d3nn9 (2_45_1_1)	2sil-d2sil (2_45_1_1)	4aahc-d4aaha (2_47_1_1)	2hmb-d1hms (2_41_1_2)	1ure-d1ifc (2_41_1_2)	1cdcb-d1cdcb (2_1_1_1)	1occb-d1occb1 (2_5_1_2)
287	1gesa-d1gesa3	4_46_1_1	115	5	1	1	0	0	0	0	1fead-d2tpra3 (4_46_1_1)	1lvl-d1lvl_3 (4_46_1_1)	2npx-d1nhp_3 (4_46_1_1)	1alo-d1alo_6 (4_77_1_1)	2ms2a-d2ms2a (4_45_1_1)	1gtma-d1gtma2 (3_54_1_1)	1tsra-d1tupa (2_2_3_1)	1ngi-d1hpm_2 (3_41_1_1)	7cat-d7cata (5_6_1_1)
288	1geua-d1gesa2	3_4_1_4	116	6	1	1	1	1	0	0	1febb-d2tpra2 (3_4_1_4)	1lvl-d1lvl_2 (3_4_1_4)	1nhq-d1nhp_2 (3_4_1_4)	2tmda-d2tmda2 (3_4_1_1)	1tde-d1tde_2 (3_4_1_4)	1coy-d1coy_1 (3_4_1_2)	1psda-d1psda2 (3_19_1_4)	1fcdb-d1fcda2 (3_4_1_4)	1dxy-d1dxy_2 (3_19_1_4)
289	1gf2-d1igl	7_1_1_1	67	1	0	0	0	0	0	0	1vkla-d3pmga2 (3_64_1_1)	1faga-d2bmha (1_75_1_1)	1btl-d1btl (5_4_1_1)	2chr-d2chr_1 (3_1_6_2)	1ntr-d1ntr (3_13_2_1)	2ebn-d2ebn (3_1_1_5)	1gky-d1gky (3_25_1_1)	2myd-d1mbd (1_1_1_1)	1scub-d1scub2 (4_83_1_3)
290	1ggga-d1ggga	3_73_1_1	220	8	1	1	0	0	0	0	1lst-d1lst (3_73_1_1)	1sbp-d1sbp (3_73_1_1)	1oiba-d1pbp (3_73_1_1)	1dxy-d1dxy_2 (3_19_1_4)	1dppc-d1dppa (3_73_1_1)	1jeva-d2olba (3_73_1_1)	1idm-d1idm (3_57_1_1)	3btoa-d2ohxa2 (3_19_1_1)	1cyda-d1cyda (3_19_1_2)
291	1ggt-d1ggta2	2_1_4_1	112	1	0	0	0	0	0	0	1mpmb-d1mal (6_7_1_2)	1ospo-d1ospo (2_52_1_1)	1prn-d1prn (6_7_1_1)	1exg-d1exg (2_2_2_1)	1dlc-d1dlc_2 (2_53_2_1)	2hmb-d1hms (2_41_1_2)	2sil-d2sil (2_45_1_1)	1obpa-d1obpa (2_41_1_1)	1kcw-d1kcw_5 (2_5_1_3)
292	1ggt-d1ggta4	4_3_1_2	325	1	0	0	0	0	0	0	1gof-d1gof_3 (2_46_1_1)	1scha-d1scha (1_65_1_1)	1cowe-d1bmfd3 (3_25_1_6)	2mnr-d2mnr_1 (3_1_6_2)	2lbp-d2lbp (3_72_1_1)	1llda-d1llda2 (4_92_1_1)	2aaa-d2aaa_2 (3_1_1_1)	1coy-d1coy_1 (3_4_1_2)	1dlha-d1dlha1 (2_1_1_2)
293	1ghj-d1ghk	2_59_1_1	79	5	1	1	0	0	1	1	1bdo-d1bdo (2_59_1_1)	1iyu-d1iyu (2_59_1_1)	1lac-d1lac (2_59_1_1)	1hpca-d1htp (2_59_1_1)	1gpr-d1gpr (2_59_3_1)	1hpm-d1hpm_1 (3_41_1_1)	1kcw-d1kcw_1 (2_5_1_3)	1qapa-d1qapa2 (4_23_2_1)	1kob-d1koba (5_1_1_1)
294	1ghr-d1ghr	3_1_1_3	306	5	0	1	0	0	0	1	1nai-d1xel (3_19_1_2)	1xyza-d1xyza (3_1_1_3)	1sfta-d1sfta2 (3_1_18_1)	2ada-d1add (3_1_2_1)	1gca-d1gca (3_72_1_1)	1eceb-d1ecea (3_1_1_3)	4xis-d2xis (3_1_12_1)	2dri-d2dri (3_72_1_1)	1dhr-d1dhr (3_19_1_2)
295	1gks-d1gks	1_3_1_1	78	7	1	1	0	0	0	0	1cc5-d1cc5 (1_3_1_1)	2mtac-d2mtac (1_3_1_1)	1cyj-d1cyi (1_3_1_1)	1beo-d1beo (1_96_1_1)	1afra-d1afra (1_24_1_2)	2sas-d2sas (1_34_1_5)	2gpb-d1gpb (3_68_1_2)	119l-d119l (4_2_1_3)	1sly-d1sly_1 (1_84_4_1)
296	1gky-d1gky	3_25_1_1	185	2	0	0	0	1	0	0	1nfp-d1nfp (3_1_13_2)	1tlfa-d1tlfa (3_72_1_1)	2masa-d2masa (3_51_1_1)	1eft-d1eft_3 (3_25_1_3)	1bya-d1byb (3_1_1_2)	1sfta-d1sfta2 (3_1_18_1)	1nal1-d1nal1 (3_1_3_1)	1jeva-d2olba (3_73_1_1)	1igs-d1igs (3_1_8_1)
297	1glm-d1glm	1_73_1_1	470	1	0	0	1	1	0	0	1cem-d1cem (1_73_1_2)	1occc-d1occc1 (6_5_1_1)	1pgo-d2pgd_1 (1_71_1_1)	1occa-d1occa1 (6_5_1_1)	1sly-d1sly_1 (1_84_4_1)	1ryt-d1ryt_1 (1_24_1_1)	1clc-d1clc_1 (1_73_1_2)	1prcl-d1prcl1 (6_5_1_1)	1hvd-d1hvd (1_51_1_1)
298	1gln-d1gln_1	1_67_1_1	163	1	0	0	0	0	0	0	1pgo-d2pgd_1 (1_71_1_1)	1cpca-d1cpca (1_1_1_2)	1afra-d1afra (1_24_1_2)	1lh7-d1lh1 (1_1_1_1)	1xsm-d1xsm (1_24_1_2)	1osa-d1osa (1_34_1_5)	1flp-d1flp (1_1_1_1)	1itha-d1itha (1_1_1_1)	1cola-d1cola (6_1_1_1)
299	1gln-d1gln_2	3_15_1_1	305	2	1	1	0	0	0	0	1gtra-d1gtra2 (3_15_1_1)	2ada-d1add (3_1_2_1)	1nis-d1aco_2 (3_63_1_1)	1pnr-d1pnra2 (3_72_1_1)	2dri-d2dri (3_72_1_1)	1rvva-d1rvva (3_9_1_1)	1btl-d1btl (5_4_1_1)	2hnp-d2hnp (3_32_1_2)	2ebn-d2ebn (3_1_1_5)
300	1glqa-d1glqa1	1_38_1_1	131	3	1	1	0	0	0	0	3gsta-d2gsta1 (1_38_1_1)	1gsea-d1gsea1 (1_38_1_1)	1prcl-d1prcl1 (6_5_1_1)	1ciy-d1ciy_3 (6_1_3_1)	2myd-d1mbd (1_1_1_1)	1aep-d1aep (1_49_1_1)	1mrrb-d1riba (1_24_1_2)	2hbg-d1hbg (1_1_1_1)	1flp-d1flp (1_1_1_1)
301	1gmqb-d1gmpa	4_1_1_1	96	3	1	1	0	0	0	0	1brnl-d1brnl (4_1_1_1)	2sil-d2sil (2_45_1_1)	1oacb-d1oaca3 (4_14_2_1)	1eur-d1eur (2_45_1_1)	1qba-d1qba_1 (2_1_1_5)	2trcb-d2trcb (2_46_3_1)	1tif-d1tif (4_12_6_1)	1vjs-d1bplb1 (2_48_1_1)	2phl-d2phla1 (2_58_1_1)
302	1gnd-d1gnd_2	4_13_1_5	97	1	0	0	0	0	0	0	1alo-d1alo_5 (4_77_1_1)	7cat-d7cata (5_6_1_1)	1lcl-d1lcl (2_19_1_3)	1bvp1-d1bvp12 (2_14_1_1)	1emd-d2cmd_1 (3_19_1_5)	1imba-d2hhma (5_8_1_2)	1cdaa-d1aly (2_17_1_1)	1pgs-d1pgs_1 (2_11_1_1)	1asu-d1asu (3_41_3_2)
303	1gof-d1gof_2	2_13_1_1	150	1	0	0	0	0	0	0	1plq-d1plq_1 (4_76_1_2)	2pola-d2pola3 (4_76_1_1)	1bgl-d1bgla1 (2_1_3_1)	1ospo-d1ospo (2_52_1_1)	4aahc-d4aaha (2_47_1_1)	1dlc-d1dlc_1 (2_13_1_2)	2cas-d2cas (2_8_1_4)	1bgl-d1bgla2 (2_1_3_1)	1vcaa-d1vcaa1 (2_1_1_3)
304	1gof-d1gof_3	2_46_1_1	387	1	0	0	0	0	1	1	2bbkh-d2bbkh (2_46_2_1)	4aahc-d4aaha (2_47_1_1)	2trcb-d2trcb (2_46_3_1)	1ospo-d1ospo (2_52_1_1)	1eur-d1eur (2_45_1_1)	2sil-d2sil (2_45_1_1)	1cdaa-d1aly (2_17_1_1)	3nn9-d3nn9 (2_45_1_1)	2bfh-d2fgf (2_28_1_1)
305	1gog-d1gof_1	2_1_1_5	102	6	0	1	0	1	1	1	1noa-d1noa (2_1_6_1)	2bbkh-d2bbkh (2_46_2_1)	1clc-d1clc_2 (2_1_1_5)	1hpl-d1hpla1 (2_10_2_1)	1cdcb-d1cdcb (2_1_1_1)	1tcmb-d1cdg_1 (2_1_1_5)	1gof-d1gof_3 (2_46_1_1)	1ospo-d1ospo (2_52_1_1)	1iob-d1i1b (2_28_1_2)
306	1gp2g-d1gp2g	8_47_1_1	54	2	1	1	0	0	0	0	2trcg-d2trcg (8_47_1_1)	1dlc-d1dlc_3 (6_1_3_1)	1wsyb-d1wsyb (3_59_1_1)	1flp-d1flp (1_1_1_1)	1lh7-d1lh1 (1_1_1_1)	1pmi-d1pmi (2_58_2_1)	1jkw-d1jkw_1 (1_59_1_1)	1hula-d1hula (1_25_1_2)	1cola-d1cola (6_1_1_1)
307	1gpc-d1gpc	2_26_4_7	218	3	0	0	0	0	0	0	2npx-d1nhp_3 (4_46_1_1)	1sva5-d1sva1 (2_8_1_4)	1aocb-d1aoca (7_14_1_5)	3rubs-d3rubs (4_37_1_1)	1lrv-d1lrv (1_84_2_1)	1gog-d1gof_1 (2_1_1_5)	1noa-d1noa (2_1_6_1)	1gcb-d1gcb (4_3_1_1)	4mhta-d1hmy (3_47_1_4)
308	1gph1-d1gph11	3_44_1_1	231	3	1	1	0	0	0	0	1opr-d1opr (3_44_1_1)	1hmp-d1hmpa (3_44_1_1)	1tlfa-d1tlfa (3_72_1_1)	1poxa-d1poxa2 (3_24_1_1)	1scub-d1scub1 (3_13_3_1)	3pmga-d3pmga1 (3_64_1_1)	1nal1-d1nal1 (3_1_3_1)	1fbaa-d1fbaa (3_1_3_1)	1dora-d1dora (3_1_7_1)
309	1gph1-d1gph12	4_88_1_1	234	2	1	1	0	0	0	0	1gdob-d1gdoa (4_88_1_1)	4aahc-d4aaha (2_47_1_1)	1eal-d1eal (2_41_1_2)	1hgea-d1hgea (2_14_1_2)	1cyda-d1cyda (3_19_1_2)	1dkza-d1dkza (5_17_1_1)	2pola-d2pola3 (4_76_1_1)	1msk-d1msk (4_99_1_1)	1phk-d1phk (5_1_1_1)
310	1gpma-d1gpma2	3_49_1_1	205	1	0	0	0	0	0	0	3pmga-d3pmga1 (3_64_1_1)	1tiig-d1tiid (2_26_2_1)	1han-d1han_1 (4_20_1_3)	2naca-d2naca2 (3_19_1_4)	1nis-d1aco_2 (3_63_1_1)	1kit-d1kit_1 (2_19_1_6)	1scua-d1scua2 (3_19_1_8)	1seta-d1seta2 (4_59_1_1)	1psda-d1psda2 (3_19_1_4)
311	1gpma-d1gpma3	4_30_1_1	121	1	0	0	0	0	0	0	1hxp-d1hxpa2 (4_10_1_2)	1nai-d1xel (3_19_1_2)	1ihfb-d1ihfb (1_46_1_1)	1eac-d1eaf (3_30_1_1)	1ris-d1ris (4_33_11_1)	1nfp-d1nfp (3_1_13_2)	1xra-d1mxa_2 (4_75_1_1)	1cof-d1ahq (4_60_1_2)	2csn-d1csn (5_1_1_1)
312	1gpr-d1gpr	2_59_3_1	158	1	0	0	0	0	0	1	2trcb-d2trcb (2_46_3_1)	1kxa-d2snv (2_31_1_3)	1iyu-d1iyu (2_59_1_1)	1hcz-d1ctm_2 (2_59_2_2)	1mrj-d1mrj (4_94_1_1)	1qila-d1tssa1 (2_26_2_2)	1febb-d2tpra2 (3_4_1_4)	1bdo-d1bdo (2_59_1_1)	1qapa-d1qapa2 (4_23_2_1)
313	1gps-d1gps	7_3_6_4	47	1	0	0	0	1	0	0	2trcb-d2trcb (2_46_3_1)	1ica-d1ica (7_3_6_3)	1dppc-d1dppa (3_73_1_1)	1fmb-d1fmb (2_34_1_1)	1eac-d1eaf (3_30_1_1)	1vkla-d3pmga2 (3_64_1_1)	1kfd-d1kfd_1 (3_41_3_4)	1qba-d1qba_3 (3_1_1_6)	1cof-d1ahq (4_60_1_2)
314	1grj-d1grj_1	1_2_1_1	78	1	0	0	0	0	0	0	1mrrb-d1riba (1_24_1_2)	1zymb-d1zyma (3_5_1_2)	1bfra-d1bcfa (1_24_1_1)	1occb-d1occb2 (6_5_1_1)	1xsm-d1xsm (1_24_1_2)	1mtyd-d1mmod (1_24_1_2)	1lpe-d1lpe (1_23_1_1)	1bbha-d1bbha (1_23_3_2)	1buca-d1buca1 (1_23_6_1)
315	1gsa-d2glt_1	3_20_1_3	122	1	0	0	0	0	0	0	1kob-d1koba (5_1_1_1)	1han-d1han_1 (4_20_1_3)	1asu-d1asu (3_41_3_2)	1psda-d1psda2 (3_19_1_4)	1pda-d1pda_2 (4_28_2_1)	1kcw-d1kcw_5 (2_5_1_3)	1tkca-d1trka3 (3_34_1_1)	1oela-d1grl_2 (3_5_3_1)	1gln-d1gln_2 (3_15_1_1)
316	1gsea-d1gsea1	1_38_1_1	142	3	1	1	0	0	0	0	1glqa-d1glqa1 (1_38_1_1)	3gsta-d2gsta1 (1_38_1_1)	1prcl-d1prcl1 (6_5_1_1)	1aep-d1aep (1_49_1_1)	1occc-d1occc1 (6_5_1_1)	1rpa-d1rpa (3_43_1_2)	1lpe-d1lpe (1_23_1_1)	1fps-d1fps (1_91_1_1)	1lis-d1lis (1_17_1_1)
317	1gsea-d1gsea2	3_33_1_5	79	3	1	1	0	0	0	0	2glra-d1glqa2 (3_33_1_5)	3gsta-d2gsta2 (3_33_1_5)	1jdya-d3pmga3 (3_64_1_1)	1dnpa-d1dnpa2 (3_17_1_1)	1llo-d1hvq (3_1_1_5)	1lst-d1lst (3_73_1_1)	1wsyb-d1wsyb (3_59_1_1)	1qora-d1qora2 (3_19_1_1)	1egr-d1ego (3_33_1_1)
318	1gtma-d1gtma1	3_19_1_7	239	3	1	1	1	1	0	0	1hrda-d1hrda1 (3_19_1_7)	1leha-d1leha1 (3_19_1_7)	1dxy-d1dxy_2 (3_19_1_4)	2naca-d2naca2 (3_19_1_4)	1psda-d1psda2 (3_19_1_4)	1ciy-d1ciy_3 (6_1_3_1)	1miob-d1miob (3_67_1_1)	1lbd-d1lbd (1_87_1_1)	1mioa-d1mioa (3_67_1_1)
319	1gtma-d1gtma2	3_54_1_1	178	3	1	1	0	0	0	0	1hrda-d1hrda2 (3_54_1_1)	1leha-d1leha2 (3_54_1_1)	1gca-d1gca (3_72_1_1)	2dri-d2dri (3_72_1_1)	2gpb-d1gpb (3_68_1_2)	1nfp-d1nfp (3_1_13_2)	1tml-d1tml (3_2_1_1)	1art-d1art (3_48_1_1)	1ahia-d1fmca (3_19_1_2)
320	1gtqa-d1gtqa	4_54_1_2	138	1	0	0	0	0	0	0	1chra-d2chr_2 (4_31_1_1)	2hnp-d2hnp (3_32_1_2)	7icd-d7icd (3_57_1_1)	2aky-d1aky (3_25_1_1)	1rpa-d1rpa (3_43_1_2)	1art-d1art (3_48_1_1)	3sdha-d3sdha (1_1_1_1)	1nis-d1aco_2 (3_63_1_1)	1lucb-d1lucb (3_1_13_1)
321	1gtra-d1gtra2	3_15_1_1	331	2	1	1	0	0	0	0	1gln-d1gln_2 (3_15_1_1)	1lucb-d1lucb (3_1_13_1)	2dri-d2dri (3_72_1_1)	2ada-d1add (3_1_2_1)	1fbaa-d1fbaa (3_1_3_1)	1gca-d1gca (3_72_1_1)	2bnh-d1dfji (3_7_1_1)	1nal1-d1nal1 (3_1_3_1)	1tlfa-d1tlfa (3_72_1_1)
322	1guab-d1guab	4_12_3_1	76	1	0	0	0	0	1	1	1aara-d1ubi (4_12_2_1)	1put-d1put (4_12_4_1)	1fbaa-d1fbaa (3_1_3_1)	1eac-d1eaf (3_30_1_1)	1nfp-d1nfp (3_1_13_2)	1otfa-d1otfa (4_40_1_1)	2tmda-d2tmda1 (3_1_7_1)	1mbb-d1mbb_1 (4_84_1_2)	1shaa-d1shaa (4_51_1_1)
323	1gur-d1gur	7_3_4_1	33	1	0	0	0	0	0	0	1ahsa-d1ahsa (2_14_1_1)	1npoa-d1npoa (2_7_1_1)	1ihva-d1ihwa (2_21_6_1)	1eal-d1eal (2_41_1_2)	1hcd-d1hcd (2_28_4_1)	1div-d1div (4_82_1_1)	2hmb-d1hms (2_41_1_2)	2blta-d2blta (5_4_1_1)	1mbt-d1mbb_2 (4_85_1_1)
324	1gym-d1gym	3_1_15_2	296	1	0	0	0	0	1	1	1edt-d1edt (3_1_1_5)	1idm-d1idm (3_57_1_1)	1pbn-d1pbn (3_52_1_1)	1esd-d1esc (3_13_8_1)	1eac-d1eaf (3_30_1_1)	7icd-d7icd (3_57_1_1)	1fbaa-d1fbaa (3_1_3_1)	1edg-d1edg (3_1_1_3)	1nai-d1xel (3_19_1_2)
325	1han-d1han_1	4_20_1_3	131	2	1	1	0	0	0	0	1han-d1han_2 (4_20_1_3)	1daaa-d1daaa (5_14_1_1)	1abra-d1abra (4_94_1_1)	1gsa-d2glt_1 (3_20_1_3)	1fld-d5nul (3_13_4_1)	2chr-d2chr_1 (3_1_6_2)	1mtyd-d1mmod (1_24_1_2)	1art-d1art (3_48_1_1)	1onea-d1ebha1 (3_1_6_1)
326	1han-d1han_2	4_20_1_3	156	2	1	1	0	1	0	0	1han-d1han_1 (4_20_1_3)	1iov-d2dln_2 (4_83_1_1)	1froa-d1froa (4_20_1_1)	1gcb-d1gcb (4_3_1_1)	1ecpa-d1ecpa (3_52_1_1)	1div-d1div (4_82_1_1)	1juy-d1adea (3_25_1_5)	1geua-d1gesa2 (3_4_1_4)	1dar-d1dar_3 (4_11_1_1)
327	1hava-d1hava	2_31_1_4	216	1	0	0	0	1	0	0	1pls-d1pls (2_37_1_1)	1p03a-d2alp (2_31_1_1)	2hmb-d1hms (2_41_1_2)	1kiqa-d1vfba (2_1_1_1)	2sil-d2sil (2_45_1_1)	1xnb-d1xnb (2_19_1_8)	3nn9-d3nn9 (2_45_1_1)	1hgea-d1hgea (2_14_1_2)	1kxa-d2snv (2_31_1_3)
328	1hcd-d1hcd	2_28_4_1	118	1	0	0	0	0	0	1	2sil-d2sil (2_45_1_1)	1iob-d1i1b (2_28_1_2)	2bfh-d2fgf (2_28_1_1)	1pex-d1pex (2_44_1_1)	3nn9-d3nn9 (2_45_1_1)	1ure-d1ifc (2_41_1_2)	1ospo-d1ospo (2_52_1_1)	2trcb-d2trcb (2_46_3_1)	2hmb-d1hms (2_41_1_2)
329	1hcgb-d1hcgb	7_3_9_1	51	7	0	1	0	0	0	0	1hfi-d1hfi (7_15_1_1)	1cdcb-d1cdcb (2_1_1_1)	1xnb-d1xnb (2_19_1_8)	1emo-d1emo_1 (7_3_9_1)	1xxca-d1xxaa (4_38_2_1)	2bbvc-d2bbva (2_8_1_3)	1qba-d1qba_1 (2_1_1_5)	1obpa-d1obpa (2_41_1_1)	1dppc-d1dppa (3_73_1_1)
330	1hcr-d1hcra	1_4_1_2	52	2	0	0	0	1	0	1	1rnl-d1rnl_1 (1_31_1_2)	1pdn-d1pdnc (1_4_1_4)	1adr-d1adr (1_30_1_2)	1perl-d1r69 (1_30_1_2)	1dpra-d1dpra1 (1_4_3_11)	2wrpr-d2wrpr (1_78_1_1)	1hddc-d1enh (1_4_1_1)	1fipa-d1fipa (1_76_1_1)	1octc-d1octc2 (1_30_1_1)
331	1hcz-d1ctm_2	2_59_2_2	63	1	0	0	0	0	1	1	1gpr-d1gpr (2_59_3_1)	2trcb-d2trcb (2_46_3_1)	2bbkh-d2bbkh (2_46_2_1)	1eur-d1eur (2_45_1_1)	1occb-d1occb1 (2_5_1_2)	1iyu-d1iyu (2_59_1_1)	1kiqa-d1vfba (2_1_1_1)	1dkza-d1dkza (5_17_1_1)	1qapa-d1qapa2 (4_23_2_1)
332	1hdca-d1hdca	3_19_1_2	253	6	1	1	0	1	0	0	1ahia-d1fmca (3_19_1_2)	1cyda-d1cyda (3_19_1_2)	1eny-d1eny (3_19_1_2)	1dhr-d1dhr (3_19_1_2)	1nai-d1xel (3_19_1_2)	2chr-d2chr_1 (3_1_6_2)	6ldh-d1ldm_1 (3_19_1_5)	1xyza-d1xyza (3_1_1_3)	1pea-d1pea (3_72_1_1)

333	1hddc-d1enh	1_4_1_1	54	5	1	1	0	1	0	0	1octc-d1octc1 (1_4_1_1)	1yrna-d1yrna (1_4_1_1)	1yrnb-d1yrnb (1_4_1_1)	1lfb-d1lfb (1_4_1_1)	1occe-d1occe (1_84_7_1)	2wrpr-d2wrpr (1_78_1_1)	1cxh-d1cdg_4 (3_1_1_1)	1pdn-d1pdnc (1_4_1_4)	1gtma-d1gtma1 (3_19_1_7)
334	1hfh-d1hcc	7_15_1_1	59	2	1	1	0	0	0	0	1hfi-d1hfi (7_15_1_1)	1dlc-d1dlc_2 (2_53_2_1)	1iyu-d1iyu (2_59_1_1)	1han-d1han_2 (4_20_1_3)	1tuia-d1eft_1 (2_29_3_1)	1dfna-d1dfna (7_7_1_1)	1jeva-d2olba (3_73_1_1)	2cas-d2cas (2_8_1_4)	1svb-d1svb_2 (6_10_1_1)
335	1hfi-d1hfi	7_15_1_1	62	2	1	1	0	0	0	0	1hfh-d1hcc (7_15_1_1)	1hcgb-d1hcgb (7_3_9_1)	1dec-d1dec (7_18_1_1)	1sema-d1sema (2_21_2_1)	1xra-d1mxa_2 (4_75_1_1)	1tcmb-d1cdg_1 (2_1_1_5)	1kiqa-d1vfba (2_1_1_1)	1pex-d1pex (2_44_1_1)	1kcw-d1kcw_4 (2_5_1_3)
336	1hgea-d1hgea	2_14_1_2	328	1	0	0	0	0	0	0	2por-d2por (6_7_1_1)	1nsdb-d1nsca (2_45_1_1)	5sgae-d1sgc (2_31_1_1)	1kit-d1kit_2 (2_19_1_6)	1prn-d1prn (6_7_1_1)	3nn9-d3nn9 (2_45_1_1)	2plv-d1pvc1 (2_8_1_4)	1ggt-d1ggta2 (2_1_4_1)	1rsy-d1rsy (2_6_1_2)
337	1hjra-d1hjra	3_41_3_6	158	1	0	0	0	0	1	1	1ngi-d1hpm_2 (3_41_1_1)	2dri-d2dri (3_72_1_1)	1jud-d1jud (5_18_1_1)	1vnc-d1vnc (1_80_1_1)	1gca-d1gca (3_72_1_1)	1dhpa-d1dhpa (3_1_3_1)	1eft-d1eft_3 (3_25_1_3)	1cyda-d1cyda (3_19_1_2)	1cbg-d1cbg (3_1_1_4)
338	1hlb-d1hlb	1_1_1_1	156	10	1	1	1	1	0	0	1mba-d1mba (1_1_1_1)	1itha-d1itha (1_1_1_1)	2myd-d1mbd (1_1_1_1)	2lhb-d2lhb (1_1_1_1)	1flp-d1flp (1_1_1_1)	3sdha-d3sdha (1_1_1_1)	1lh7-d1lh1 (1_1_1_1)	1baba-d1baba (1_1_1_1)	1cpcb-d1cpcb (1_1_1_2)
339	1hlpa-d1hlpa1	3_19_1_5	128	4	1	1	1	1	0	0	6ldh-d1ldm_1 (3_19_1_5)	1llda-d1llda1 (3_19_1_5)	1emd-d2cmd_1 (3_19_1_5)	1ahia-d1fmca (3_19_1_2)	1hdca-d1hdca (3_19_1_2)	1dhr-d1dhr (3_19_1_2)	1reqb-d1reqb2 (3_13_5_2)	2tmda-d2tmda1 (3_1_7_1)	1orta-d1orta2 (3_58_1_1)
340	1hlpa-d1hlpa2	4_92_1_1	175	5	1	1	0	0	0	0	6ldh-d1ldm_2 (4_92_1_1)	1llda-d1llda2 (4_92_1_1)	1bdmb-d1bdma2 (4_92_1_1)	1emd-d2cmd_2 (4_92_1_1)	2tmda-d2tmda1 (3_1_7_1)	2ctb-d2ctb (3_52_3_1)	1gca-d1gca (3_72_1_1)	1nai-d1xel (3_19_1_2)	1lyla-d1lyla2 (4_59_1_1)
341	1hmda-d2hmqa	1_23_4_1	113	1	0	0	0	0	0	1	1aep-d1aep (1_49_1_1)	1dlc-d1dlc_3 (6_1_3_1)	1lpe-d1lpe (1_23_1_1)	1ciy-d1ciy_3 (6_1_3_1)	1glm-d1glm (1_73_1_1)	1occc-d1occc1 (6_5_1_1)	1vsga-d1vsga (6_3_1_1)	1cola-d1cola (6_1_1_1)	1xsm-d1xsm (1_24_1_2)
342	1hme-d1hme	1_20_1_1	77	4	1	1	0	0	0	0	1aab-d1aab (1_20_1_1)	1mmob-d1mmob (1_24_1_2)	1jkw-d1jkw_2 (1_59_1_1)	1ciy-d1ciy_3 (6_1_3_1)	1lbd-d1lbd (1_87_1_1)	2btf-d2btfa2 (3_41_1_1)	1occa-d1occa1 (6_5_1_1)	2hmx-d2hmx (1_58_1_1)	1miob-d1miob (3_67_1_1)
343	1hmp-d1hmpa	3_44_1_1	214	3	1	1	0	0	0	0	1gph1-d1gph11 (3_44_1_1)	1dora-d1dora (3_1_7_1)	1bll-d1lam_2 (3_52_3_3)	1wsyb-d1wsyb (3_59_1_1)	1llo-d1hvq (3_1_1_5)	1pnr-d1pnra2 (3_72_1_1)	1opr-d1opr (3_44_1_1)	1pdo-d1pdo (3_40_1_1)	1mioa-d1mioa (3_67_1_1)
344	1hnr-d1hnr	1_46_1_1	47	3	0	0	0	0	0	0	2baa-d2baa (4_2_1_1)	1gsea-d1gsea1 (1_38_1_1)	1gcs-d4gcr_1 (2_9_1_1)	2blta-d2blta (5_4_1_1)	1dkza-d1dkza (5_17_1_1)	1pea-d1pea (3_72_1_1)	1fld-d5nul (3_13_4_1)	1tme1-d1tme1 (2_8_1_4)	1pii-d1pii_2 (3_1_8_1)
345	1hora-d1deaa	3_23_1_1	266	1	0	0	0	0	0	0	1amp-d1amp (3_52_3_4)	1gtra-d1gtra2 (3_15_1_1)	1cowe-d1bmfd3 (3_25_1_6)	1hjra-d1hjra (3_41_3_6)	1pnr-d1pnra2 (3_72_1_1)	2chr-d2chr_1 (3_1_6_2)	1dnpa-d1dnpa2 (3_17_1_1)	1orta-d1orta1 (3_58_1_1)	1rvva-d1rvva (3_9_1_1)
346	1hpca-d1htp	2_59_1_1	131	5	1	1	0	0	0	1	1iyu-d1iyu (2_59_1_1)	1ghj-d1ghk (2_59_1_1)	1bdo-d1bdo (2_59_1_1)	1nis-d1aco_2 (3_63_1_1)	1lac-d1lac (2_59_1_1)	1btn-d1btn (2_37_1_1)	1qila-d1tssa1 (2_26_2_2)	1oxa-d1oxa (1_75_1_1)	1gpr-d1gpr (2_59_3_1)
347	1hph-d1hph	8_14_1_1	37	1	0	0	0	0	0	0	1div-d1div (4_82_1_1)	3sdha-d3sdha (1_1_1_1)	1eri-d1eria (3_38_1_1)	2myd-d1mbd (1_1_1_1)	1pii-d1pii_2 (3_1_8_1)	1ngi-d1hpm_2 (3_41_1_1)	1lfb-d1lfb (1_4_1_1)	1buca-d1buca2 (5_7_1_1)	4kbpa-d4kbpa2 (4_91_1_1)
348	1hpi-d1hpi	7_27_1_1	71	4	1	1	0	0	0	0	1hrq-d1hrq (7_27_1_1)	2hipa-d2hipa (7_27_1_1)	1isua-d1isua (7_27_1_1)	1mbt-d1mbb_2 (4_85_1_1)	2tmda-d2tmda1 (3_1_7_1)	1kcw-d1kcw_4 (2_5_1_3)	1sva5-d1sva1 (2_8_1_4)	1akl-d1kapp2 (4_50_1_6)	3b5c-d3b5c (4_66_1_1)
349	1hpl-d1hpla1	2_10_2_1	112	1	0	0	0	0	0	0	1dkza-d1dkza (5_17_1_1)	1nsdb-d1nsca (2_45_1_1)	1ospo-d1ospo (2_52_1_1)	1bgl-d1bgla1 (2_1_3_1)	1obpa-d1obpa (2_41_1_1)	1fem-d1hbp (2_41_1_1)	3nn9-d3nn9 (2_45_1_1)	1clc-d1clc_2 (2_1_1_5)	1noa-d1noa (2_1_6_1)
350	1hpm-d1hpm_1	3_41_1_1	185	4	0	0	0	0	0	0	1tlfa-d1tlfa (3_72_1_1)	1pea-d1pea (3_72_1_1)	1coy-d1coy_1 (3_4_1_2)	2ada-d1add (3_1_2_1)	1xyza-d1xyza (3_1_1_3)	1sbp-d1sbp (3_73_1_1)	1orta-d1orta1 (3_58_1_1)	1igs-d1igs (3_1_8_1)	1mbt-d1mbb_2 (4_85_1_1)
351	1hqi-d1hqi	4_79_1_1	90	1	0	0	0	0	0	0	1lyla-d1lyla2 (4_59_1_1)	1lylb-d1lyla1 (2_26_4_1)	2dkb-d2dkb (3_48_1_3)	1dar-d1dar_4 (4_33_12_1)	1plr-d1plq_2 (4_76_1_2)	1hpm-d1hpm_1 (3_41_1_1)	1wsyb-d1wsyb (3_59_1_1)	2pec-d2pec (2_56_1_1)	1pne-d1pne (4_61_1_1)
352	1hrda-d1hrda1	3_19_1_7	255	3	1	1	0	0	0	0	1gtma-d1gtma1 (3_19_1_7)	1leha-d1leha1 (3_19_1_7)	2chr-d2chr_1 (3_1_6_2)	1fbaa-d1fbaa (3_1_3_1)	1art-d1art (3_48_1_1)	1tlfa-d1tlfa (3_72_1_1)	1dlc-d1dlc_3 (6_1_3_1)	2dri-d2dri (3_72_1_1)	1nal1-d1nal1 (3_1_3_1)
353	1hrda-d1hrda2	3_54_1_1	194	3	1	1	0	0	0	0	1gtma-d1gtma2 (3_54_1_1)	1leha-d1leha2 (3_54_1_1)	1taha-d1tahb (3_50_1_8)	1fbaa-d1fbaa (3_1_3_1)	1btl-d1btl (5_4_1_1)	1gca-d1gca (3_72_1_1)	2hnp-d2hnp (3_32_1_2)	1ngi-d1hpm_2 (3_41_1_1)	1mba-d1mba (1_1_1_1)
354	1hrq-d1hrq	7_27_1_1	85	4	1	1	0	0	0	0	1hpi-d1hpi (7_27_1_1)	2hipa-d2hipa (7_27_1_1)	1isua-d1isua (7_27_1_1)	1mbt-d1mbb_2 (4_85_1_1)	2tmda-d2tmda1 (3_1_7_1)	1ezm-d1ezm_1 (1_53_1_1)	1dar-d1dar_1 (2_29_3_1)	1akl-d1kapp2 (4_50_1_6)	1lbd-d1lbd (1_87_1_1)
355	1hry-d1hrza	1_20_1_1	73	4	0	0	0	0	0	0	1knya-d1knya (5_10_1_2)	1bmta-d1bmta1 (1_39_1_1)	1occa-d1occa1 (6_5_1_1)	1phe-d1phb (1_75_1_1)	1art-d1art (3_48_1_1)	1hme-d1hme (1_20_1_1)	2dhd-d1ede (3_50_1_3)	1rpa-d1rpa (3_43_1_2)	1glm-d1glm (1_73_1_1)
356	1hsq-d1hsq	2_21_2_1	71	4	1	1	0	0	1	1	1aey-d1shg (2_21_2_1)	1sema-d1sema (2_21_2_1)	1ihva-d1ihwa (2_21_6_1)	1pht-d1pht (2_21_2_1)	1gen-d1gen (2_44_1_1)	1prhb-d1prha1 (1_65_1_2)	1pky-d1pkya1 (2_40_1_1)	1rie-d1rie (7_33_1_2)	1bia-d1bia_2 (2_21_1_1)
357	1hsta-d1hsta	1_4_3_7	74	1	0	0	0	0	0	0	1vhra-d1vhra (3_32_1_1)	1vin-d1vin_1 (1_59_1_1)	1ytn-d1ypta (3_32_1_2)	1rlr-d1rlr_1 (1_68_1_1)	1cem-d1cem (1_73_1_2)	2ada-d1add (3_1_2_1)	1rpl-d1rpl (5_10_1_1)	1tyc-d2ts1_1 (1_54_1_1)	1lucb-d1lucb (3_1_13_1)
358	1htda-d1atla	4_50_1_5	200	1	0	0	0	0	0	0	4xis-d2xis (3_1_12_1)	1tlfa-d1tlfa (3_72_1_1)	1dhr-d1dhr (3_19_1_2)	1hdca-d1hdca (3_19_1_2)	1mioa-d1mioa (3_67_1_1)	1opr-d1opr (3_44_1_1)	2lbp-d2lbp (3_72_1_1)	1oya-d1oya (3_1_7_1)	1lyla-d1lyla2 (4_59_1_1)
359	1htt-d1htta1	3_37_1_1	99	1	0	0	0	0	0	0	1bmta-d1bmta2 (3_13_5_1)	1oiba-d1pbp (3_73_1_1)	1pii-d1pii_2 (3_1_8_1)	1tlfa-d1tlfa (3_72_1_1)	1qapa-d1qapa1 (3_1_14_1)	1dora-d1dora (3_1_7_1)	1sbp-d1sbp (3_73_1_1)	1leha-d1leha1 (3_19_1_7)	1dhpa-d1dhpa (3_1_3_1)
360	1huea-d1huea	1_46_1_1	90	3	1	1	0	0	0	0	1ihfb-d1ihfb (1_46_1_1)	1miob-d1miob (3_67_1_1)	1lucb-d1lucb (3_1_13_1)	1daaa-d1daaa (5_14_1_1)	6ldh-d1ldm_2 (4_92_1_1)	1buca-d1buca1 (1_23_6_1)	1fbaa-d1fbaa (3_1_3_1)	1hxp-d1hxpa2 (4_10_1_2)	2cpo-d1cpo_1 (1_34_2_1)
361	1hula-d1hula	1_25_1_2	108	3	0	0	0	0	0	0	1tnda-d1tada1 (1_52_1_1)	1xsm-d1xsm (1_24_1_2)	1mdya-d1mdya (1_33_1_1)	1dlc-d1dlc_3 (6_1_3_1)	1hlb-d1hlb (1_1_1_1)	2ccya-d2ccya (1_23_3_2)	2liga-d2liga (1_23_2_1)	1lis-d1lis (1_17_1_1)	1hrda-d1hrda1 (3_19_1_7)
362	1huma-d1huma	4_7_1_1	69	3	1	1	0	0	0	0	1dona-d1dona (4_7_1_1)	1napd-d1napa (4_7_1_1)	1mbb-d1mbb_1 (4_84_1_2)	2dkb-d2dkb (3_48_1_3)	1bove-d1bova (2_26_2_1)	1tkca-d1trka3 (3_34_1_1)	1dyna-d1dyna (2_37_1_1)	2prd-d2prd (2_26_5_1)	1kte-d1kte (3_33_1_1)
363	1hvd-d1hvd	1_51_1_1	313	1	0	0	0	0	0	0	1sly-d1sly_1 (1_84_4_1)	1mrrb-d1riba (1_24_1_2)	1occa-d1occa1 (6_5_1_1)	1mtyd-d1mmod (1_24_1_2)	1pgo-d2pgd_1 (1_71_1_1)	1xsm-d1xsm (1_24_1_2)	1csr-d1csh (1_74_1_1)	1occc-d1occc1 (6_5_1_1)	1afra-d1afra (1_24_1_2)
364	1hxp-d1hxpa2	4_10_1_2	171	1	0	0	1	1	0	0	1kpaa-d1kpaa (4_10_1_1)	1gbs-d1gbs (4_2_1_4)	1llda-d1llda2 (4_92_1_1)	1phk-d1phk (5_1_1_1)	1wsyb-d1wsyb (3_59_1_1)	1gpma-d1gpma3 (4_30_1_1)	2anhb-d2anha (3_56_1_1)	1msk-d1msk (4_99_1_1)	1iov-d2dln_2 (4_83_1_1)
365	1hyp-d1hyp	1_42_1_1	75	2	0	0	0	0	0	0	1sly-d1sly_1 (1_84_4_1)	1mrrb-d1riba (1_24_1_2)	1xsm-d1xsm (1_24_1_2)	1rnl-d1rnl_1 (1_31_1_2)	1bmta-d1bmta1 (1_39_1_1)	1afra-d1afra (1_24_1_2)	2ccya-d2ccya (1_23_3_2)	1glm-d1glm (1_73_1_1)	1nox-d1nox (4_49_1_1)
366	1iad-d1ast	4_50_1_4	200	1	0	0	1	1	0	0	1ezm-d1ezm_2 (4_50_1_2)	1nfp-d1nfp (3_1_13_2)	1akl-d1kapp2 (4_50_1_6)	1ppi-d1ppi_2 (3_1_1_1)	1phe-d1phb (1_75_1_1)	1lucb-d1lucb (3_1_13_1)	1eac-d1eaf (3_30_1_1)	2ctb-d2ctb (3_52_3_1)	1rcf-d1rcf (3_13_4_1)
367	1iba-d1iba	4_53_1_1	78	1	0	0	0	0	0	0	1xxca-d1xxaa (4_38_2_1)	1fwp-d1fwp (4_33_18_1)	1asza-d1asya2 (4_59_1_1)	1fead-d2tpra3 (4_46_1_1)	1cowe-d1bmfd3 (3_25_1_6)	3pte-d3pte (5_4_1_1)	1pkp-d1pkp_1 (4_11_1_1)	1svq-d1svr (4_60_1_1)	1gdob-d1gdoa (4_88_1_1)
368	1ica-d1ica	7_3_6_3	40	1	0	0	1	1	0	0	1gps-d1gps (7_3_6_4)	1igd-d1igd (4_12_1_1)	1aps-d1aps (4_33_10_1)	1ad2-d1ad2 (5_20_1_1)	1lba-d1lba (4_64_1_1)	1ggga-d1ggga (3_73_1_1)	1dar-d1dar_4 (4_33_12_1)	1aba-d1aba (3_33_1_1)	1osa-d1osa (1_34_1_5)
369	1idm-d1idm	3_57_1_1	343	2	1	1	0	0	0	0	7icd-d7icd (3_57_1_1)	1sfta-d1sfta2 (3_1_18_1)	1sbp-d1sbp (3_73_1_1)	2dri-d2dri (3_72_1_1)	1miob-d1miob (3_67_1_1)	1pnr-d1pnra2 (3_72_1_1)	1gca-d1gca (3_72_1_1)	1tlfa-d1tlfa (3_72_1_1)	2ctb-d2ctb (3_52_3_1)
370	1ifi-d1ifj	1_97_3_1	50	4	0	1	0	0	1	1	1fosf-d1fosf (1_97_2_1)	1fose-d1fose (1_97_2_1)	1ifl-d1ifl (1_97_3_1)	1afra-d1afra (1_24_1_2)	1aep-d1aep (1_49_1_1)	1rcd-d1rcd (1_24_1_1)	1occc-d1occc1 (6_5_1_1)	1bfra-d1bcfa (1_24_1_1)	1xsm-d1xsm (1_24_1_2)
371	1ifl-d1ifl	1_97_3_1	53	4	0	0	0	0	0	1	2hmgd-d1htmb (6_2_1_1)	1vsga-d1vsga (6_3_1_1)	1fose-d1fose (1_97_2_1)	1lki-d1lki (1_25_1_1)	1bfra-d1bcfa (1_24_1_1)	1ryt-d1ryt_1 (1_24_1_1)	1occc-d1occc1 (6_5_1_1)	1ifi-d1ifj (1_97_3_1)	1gtma-d1gtma1 (3_19_1_7)
372	1igd-d1igd	4_12_1_1	61	1	0	0	0	0	0	0	1mml-d1mml (5_9_1_2)	1bmfc-d1bmfa3 (3_25_1_6)	1gtma-d1gtma2 (3_54_1_1)	1dora-d1dora (3_1_7_1)	1pne-d1pne (4_61_1_1)	1oaca-d1oaca4 (4_42_1_1)	1han-d1han_2 (4_20_1_3)	1han-d1han_1 (4_20_1_3)	1btn-d1btn (2_37_1_1)
373	1igna-d1igna1	1_4_1_5	86	1	0	0	0	0	0	0	1mmob-d1mmob (1_24_1_2)	1buca-d1buca1 (1_23_6_1)	2ctb-d2ctb (3_52_3_1)	1dpra-d1dpra2 (1_61_1_1)	1apa-d1apa (4_94_1_1)	2hnp-d2hnp (3_32_1_2)	1tcob-d1tcob (1_34_1_5)	1abra-d1abra (4_94_1_1)	2hbg-d1hbg (1_1_1_1)
374	1igs-d1igs	3_1_8_1	247	3	0	1	0	0	1	1	1nal1-d1nal1 (3_1_3_1)	1miob-d1miob (3_67_1_1)	1dora-d1dora (3_1_7_1)	1nsj-d1nsj (3_1_8_1)	1art-d1art (3_48_1_1)	1sfta-d1sfta2 (3_1_18_1)	2mnr-d2mnr_1 (3_1_6_2)	1qapa-d1qapa1 (3_1_14_1)	1tlfa-d1tlfa (3_72_1_1)
375	1ihfb-d1ihfb	1_46_1_1	94	3	1	1	0	0	0	0	1huea-d1huea (1_46_1_1)	1glm-d1glm (1_73_1_1)	1gpma-d1gpma3 (4_30_1_1)	1dnpa-d1dnpa2 (3_17_1_1)	2tmda-d2tmda1 (3_1_7_1)	1rcd-d1rcd (1_24_1_1)	2bpa1-d2bpa1 (2_8_1_1)	1tiig-d1tiid (2_26_2_1)	1prhb-d1prha1 (1_65_1_2)
376	1ihva-d1ihwa	2_21_6_1	52	1	0	0	0	0	1	1	1aey-d1shg (2_21_2_1)	1hsq-d1hsq (2_21_2_1)	1eft-d1eft_2 (2_30_1_1)	1efrd-d1bmfd2 (2_33_1_1)	1cnf-d2cnd_1 (2_29_1_1)	1prhb-d1prha1 (1_65_1_2)	1sema-d1sema (2_21_2_1)	1pex-d1pex (2_44_1_1)	1hava-d1hava (2_31_1_4)
377	1ilk-d1ilk	1_25_1_3	151	2	0	0	0	0	0	0	1mrrb-d1riba (1_24_1_2)	1ciy-d1ciy_3 (6_1_3_1)	1occc-d1occc1 (6_5_1_1)	1lpe-d1lpe (1_23_1_1)	1chka-d1chka (4_2_1_6)	1prcl-d1prcl1 (6_5_1_1)	1occa-d1occa1 (6_5_1_1)	2lhb-d2lhb (1_1_1_1)	1aep-d1aep (1_49_1_1)
378	1imba-d2hhma	5_8_1_2	272	1	0	0	1	1	0	0	1inp-d1inp (5_8_1_3)	1jud-d1jud (5_18_1_1)	2dri-d2dri (3_72_1_1)	1noy-d1noya (3_41_3_5)	1tlfa-d1tlfa (3_72_1_1)	1eft-d1eft_3 (3_25_1_3)	1mrrb-d1riba (1_24_1_2)	1rnl-d1rnl_2 (3_13_2_1)	1slba-d1slaa (2_19_1_3)
379	1inp-d1inp	5_8_1_3	400	1	0	0	1	1	0	0	1imba-d2hhma (5_8_1_2)	1dkza-d1dkza (5_17_1_1)	1apme-d1apme (5_1_1_1)	1ure-d1ifc (2_41_1_2)	1htda-d1atla (4_50_1_5)	2chr-d2chr_1 (3_1_6_2)	4aahc-d4aaha (2_47_1_1)	1mrj-d1mrj (4_94_1_1)	1dppc-d1dppa (3_73_1_1)
380	1iob-d1i1b	2_28_1_2	151	2	1	1	1	1	0	0	1irp-d1ilr1 (2_28_1_2)	2bfh-d2fgf (2_28_1_1)	4aahc-d4aaha (2_47_1_1)	1eal-d1eal (2_41_1_2)	1bebb-d1beba (2_41_1_1)	2sil-d2sil (2_45_1_1)	1tul-d1tul (2_60_4_1)	1abrb-d1abrb1 (2_28_2_1)	1gen-d1gen (2_44_1_1)

381	1iov-d2dln_2	4_83_1_1	210	1	0	0	1	1	0	0	1scub-d1scub2 (4_83_1_3)	1apme-d1apme (5_1_1_1)	1han-d1han_2 (4_20_1_3)	3mdsa-d1mnga2 (4_25_1_1)	1phk-d1phk (5_1_1_1)	1nfp-d1nfp (3_1_13_2)	1dik-d1dik_3 (4_83_1_4)	7icd-d7icd (3_57_1_1)	1ad2-d1ad2 (5_20_1_1)
382	1iow-d2dln_1	3_20_1_2	96	1	0	0	0	0	0	0	1oya-d1oya (3_1_7_1)	2dri-d2dri (3_72_1_1)	2tmda-d2tmda2 (3_4_1_1)	2ada-d1add (3_1_2_1)	1tlfa-d1tlfa (3_72_1_1)	1nipb-d1nipa (3_25_1_5)	1bncb-d1bnca2 (3_20_1_1)	1qrda-d1qrda (3_13_4_2)	1fld-d5nul (3_13_4_1)
383	1irp-d1ilr1	2_28_1_2	145	2	1	1	0	0	0	0	1iob-d1i1b (2_28_1_2)	1tnn-d1tnm (2_1_1_4)	1eal-d1eal (2_41_1_2)	1bbpa-d1bbpa (2_41_1_1)	2trcb-d2trcb (2_46_3_1)	1lcl-d1lcl (2_19_1_3)	1obpa-d1obpa (2_41_1_1)	2sil-d2sil (2_45_1_1)	1ure-d1ifc (2_41_1_2)
384	1irsa-d1irsa	2_37_1_2	112	2	0	0	1	1	0	0	1pls-d1pls (2_37_1_1)	1asza-d1asya2 (4_59_1_1)	1sso-d1sso (4_9_1_1)	1vcaa-d1vcaa2 (2_1_1_4)	2tmda-d2tmda1 (3_1_7_1)	1alo-d1alo_7 (4_77_1_1)	1ytn-d1ypta (3_32_1_2)	1pex-d1pex (2_44_1_1)	1hpca-d1htp (2_59_1_1)
385	1isua-d1isua	7_27_1_1	62	4	1	1	0	0	0	0	1hpi-d1hpi (7_27_1_1)	2hipa-d2hipa (7_27_1_1)	1hrq-d1hrq (7_27_1_1)	1seta-d1seta2 (4_59_1_1)	2blta-d2blta (5_4_1_1)	1tys-d1tys (4_63_1_1)	2bfh-d2fgf (2_28_1_1)	1obr-d1obr (3_52_3_2)	7cat-d7cata (5_6_1_1)
386	1itea-d2int	1_25_1_2	129	3	0	0	0	0	0	0	1dik-d1dik_1 (3_1_9_2)	1glm-d1glm (1_73_1_1)	1mrrb-d1riba (1_24_1_2)	1fps-d1fps (1_91_1_1)	1rcd-d1rcd (1_24_1_1)	2liga-d2liga (1_23_2_1)	1occa-d1occa1 (6_5_1_1)	1lki-d1lki (1_25_1_1)	1occc-d1occc1 (6_5_1_1)
387	1itha-d1itha	1_1_1_1	141	10	1	1	1	1	0	0	1mba-d1mba (1_1_1_1)	2myd-d1mbd (1_1_1_1)	1hlb-d1hlb (1_1_1_1)	1flp-d1flp (1_1_1_1)	3sdha-d3sdha (1_1_1_1)	2lhb-d2lhb (1_1_1_1)	1lh7-d1lh1 (1_1_1_1)	2hbg-d1hbg (1_1_1_1)	1baba-d1baba (1_1_1_1)
388	1iyu-d1iyu	2_59_1_1	79	5	1	1	0	0	1	1	1bdo-d1bdo (2_59_1_1)	1ghj-d1ghk (2_59_1_1)	1lac-d1lac (2_59_1_1)	1hpca-d1htp (2_59_1_1)	1gpr-d1gpr (2_59_3_1)	1dar-d1dar_1 (2_29_3_1)	1tdta-d1tdta (2_57_1_2)	1rgs-d1rgs_2 (2_58_3_2)	1hcz-d1ctm_2 (2_59_2_2)
389	1jdya-d3pmga3	3_64_1_1	117	3	0	0	0	0	0	0	1gsea-d1gsea2 (3_33_1_5)	1miob-d1miob (3_67_1_1)	1jud-d1jud (5_18_1_1)	1vhra-d1vhra (3_32_1_1)	4xis-d2xis (3_1_12_1)	2mnr-d2mnr_1 (3_1_6_2)	1gky-d1gky (3_25_1_1)	1cyda-d1cyda (3_19_1_2)	2cpo-d1cpo_1 (1_34_2_1)
390	1jeva-d2olba	3_73_1_1	517	8	1	1	0	0	0	0	1dppc-d1dppa (3_73_1_1)	2dri-d2dri (3_72_1_1)	1tlfa-d1tlfa (3_72_1_1)	1pea-d1pea (3_72_1_1)	1dik-d1dik_1 (3_1_9_2)	1fbaa-d1fbaa (3_1_3_1)	1sbp-d1sbp (3_73_1_1)	1gky-d1gky (3_25_1_1)	1xyza-d1xyza (3_1_1_3)
391	1jkw-d1jkw_1	1_59_1_1	151	4	0	1	0	0	0	0	2liga-d2liga (1_23_2_1)	119l-d119l (4_2_1_3)	1vin-d1vin_1 (1_59_1_1)	1occa-d1occa1 (6_5_1_1)	1aep-d1aep (1_49_1_1)	1ciy-d1ciy_3 (6_1_3_1)	1sly-d1sly_1 (1_84_4_1)	1buca-d1buca1 (1_23_6_1)	1flp-d1flp (1_1_1_1)
392	1jkw-d1jkw_2	1_59_1_1	126	4	0	0	0	0	0	0	1aep-d1aep (1_49_1_1)	1lis-d1lis (1_17_1_1)	1ciy-d1ciy_3 (6_1_3_1)	3sdha-d3sdha (1_1_1_1)	1hlb-d1hlb (1_1_1_1)	2hbg-d1hbg (1_1_1_1)	1buca-d1buca1 (1_23_6_1)	1dik-d1dik_3 (4_83_1_4)	1cpcb-d1cpcb (1_1_1_2)
393	1jlm-d1ido	3_45_1_1	184	2	1	1	0	0	0	0	1zooa-d1lfaa (3_45_1_1)	1xyza-d1xyza (3_1_1_3)	1pea-d1pea (3_72_1_1)	1lucb-d1lucb (3_1_13_1)	1cyda-d1cyda (3_19_1_2)	1opr-d1opr (3_44_1_1)	2dri-d2dri (3_72_1_1)	1nis-d1aco_2 (3_63_1_1)	1nal1-d1nal1 (3_1_3_1)
394	1jud-d1jud	5_18_1_1	220	1	0	0	0	0	0	0	1tlfa-d1tlfa (3_72_1_1)	1pgo-d2pgd_1 (1_71_1_1)	1miob-d1miob (3_67_1_1)	1sly-d1sly_1 (1_84_4_1)	1hjra-d1hjra (3_41_3_6)	2aky-d1aky (3_25_1_1)	1juy-d1adea (3_25_1_5)	1onea-d1ebha1 (3_1_6_1)	1cola-d1cola (6_1_1_1)
395	1juy-d1adea	3_25_1_5	431	2	0	0	0	0	0	0	1dik-d1dik_3 (4_83_1_4)	1jud-d1jud (5_18_1_1)	1pgo-d2pgd_1 (1_71_1_1)	1dnpa-d1dnpa1 (1_69_1_1)	1hpm-d1hpm_1 (3_41_1_1)	2blta-d2blta (5_4_1_1)	1sly-d1sly_1 (1_84_4_1)	1lucb-d1lucb (3_1_13_1)	1csr-d1csh (1_74_1_1)
396	1kal-d1kal	7_3_3_1	29	1	0	0	0	0	0	0	3nn9-d3nn9 (2_45_1_1)	1obr-d1obr (3_52_3_2)	1scua-d1scua1 (3_13_3_1)	1ppi-d1ppi_2 (3_1_1_1)	1krn-d1pk4 (7_11_1_1)	1poc-d1poc (1_95_1_1)	1efr-d1bmfa2 (2_33_1_1)	1taha-d1tahb (3_50_1_8)	1fcdc-d1fcdc1 (1_3_1_3)
397	1kcw-d1kcw_1	2_5_1_3	192	8	1	1	0	0	0	0	1kcw-d1kcw_5 (2_5_1_3)	1aspb-d1aoza1 (2_5_1_3)	1kcw-d1kcw_6 (2_5_1_3)	1asqa-d1aoza2 (2_5_1_3)	1kcw-d1kcw_4 (2_5_1_3)	1ahsa-d1ahsa (2_14_1_1)	1pgs-d1pgs_2 (2_11_1_1)	1kcw-d1kcw_2 (2_5_1_3)	1eur-d1eur (2_45_1_1)
398	1kcw-d1kcw_2	2_5_1_3	146	8	1	1	0	1	0	0	1kcw-d1kcw_6 (2_5_1_3)	1kcw-d1kcw_4 (2_5_1_3)	1asob-d1aoza3 (2_5_1_3)	1aspb-d1aoza1 (2_5_1_3)	1tlk-d1tlk (2_1_1_4)	2pcy-d1plc (2_5_1_1)	1kcw-d1kcw_5 (2_5_1_3)	2ncm-d2ncm (2_1_1_4)	1kcw-d1kcw_1 (2_5_1_3)
399	1kcw-d1kcw_4	2_5_1_3	152	8	1	1	1	1	0	0	1kcw-d1kcw_6 (2_5_1_3)	1kcw-d1kcw_2 (2_5_1_3)	1asob-d1aoza3 (2_5_1_3)	1aspb-d1aoza1 (2_5_1_3)	1kcw-d1kcw_5 (2_5_1_3)	2pcy-d1plc (2_5_1_1)	1asqa-d1aoza2 (2_5_1_3)	1kcw-d1kcw_1 (2_5_1_3)	1cur-d1cur (2_5_1_1)
400	1kcw-d1kcw_5	2_5_1_3	179	8	1	1	0	0	0	0	1kcw-d1kcw_1 (2_5_1_3)	1kcw-d1kcw_4 (2_5_1_3)	1ggt-d1ggta2 (2_1_4_1)	1gal-d1gal_1 (3_4_1_2)	1aspb-d1aoza1 (2_5_1_3)	1tlk-d1tlk (2_1_1_4)	4kbpb-d4kbpa1 (2_1_9_1)	1dud-d1dupa (2_60_3_1)	1asqa-d1aoza2 (2_5_1_3)
401	1kcw-d1kcw_6	2_5_1_3	149	8	1	1	0	1	0	0	1kcw-d1kcw_4 (2_5_1_3)	1kcw-d1kcw_2 (2_5_1_3)	1aspb-d1aoza1 (2_5_1_3)	1asob-d1aoza3 (2_5_1_3)	1asqa-d1aoza2 (2_5_1_3)	1kcw-d1kcw_1 (2_5_1_3)	1kcw-d1kcw_5 (2_5_1_3)	3nn9-d3nn9 (2_45_1_1)	1nsdb-d1nsca (2_45_1_1)
402	1kdf-d1gzi	2_60_1_1	65	1	0	0	0	0	0	0	1kcw-d1kcw_1 (2_5_1_3)	1scua-d1scua1 (3_13_3_1)	1eft-d1eft_2 (2_30_1_1)	1dlc-d1dlc_2 (2_53_2_1)	2omf-d2omf (6_7_1_1)	1dlha-d1dlha1 (2_1_1_2)	1tib-d1tib (3_50_1_7)	1qila-d1tssa1 (2_26_2_2)	1mioa-d1mioa (3_67_1_1)
403	1kfd-d1kfd_1	3_41_3_4	195	1	0	0	0	0	0	0	1lst-d1lst (3_73_1_1)	2masa-d2masa (3_51_1_1)	1ahia-d1fmca (3_19_1_2)	1cyda-d1cyda (3_19_1_2)	1vsga-d1vsga (6_3_1_1)	1knya-d1knya (5_10_1_2)	1chd-d1chd (3_27_1_1)	1ngi-d1hpm_2 (3_41_1_1)	1miob-d1miob (3_67_1_1)
404	1kifa-d1kifa2	4_13_1_3	93	1	0	0	0	0	0	0	1bebb-d1beba (2_41_1_1)	1obpa-d1obpa (2_41_1_1)	1chra-d2chr_2 (4_31_1_1)	1svb-d1svb_2 (6_10_1_1)	1asu-d1asu (3_41_3_2)	1dar-d1dar_1 (2_29_3_1)	1acf-d1acf (4_61_1_1)	1dih-d1dih_2 (4_41_1_2)	1sfta-d1sfta2 (3_1_18_1)
405	1kiqa-d1vfba	2_1_1_1	107	4	0	1	1	1	0	0	1vcaa-d1vcaa2 (2_1_1_4)	1wit-d1wiu (2_1_1_4)	1tlk-d1tlk (2_1_1_4)	1cid-d1cid_1 (2_1_1_1)	1cdi-d3cd4_2 (2_1_1_3)	1cdcb-d1cdcb (2_1_1_1)	3cd4-d3cd4_1 (2_1_1_1)	2hmb-d1hms (2_41_1_2)	2ncm-d2ncm (2_1_1_4)
406	1kit-d1kit_1	2_19_1_6	192	2	1	1	0	1	0	0	1kit-d1kit_2 (2_19_1_6)	1ospo-d1ospo (2_52_1_1)	1sacb-d1saca (2_19_1_4)	3nn9-d3nn9 (2_45_1_1)	2hmb-d1hms (2_41_1_2)	1lcl-d1lcl (2_19_1_3)	1cpn-d1cpn (2_19_1_2)	1nsdb-d1nsca (2_45_1_1)	1ahsa-d1ahsa (2_14_1_1)
407	1kit-d1kit_2	2_19_1_6	197	2	1	1	1	1	0	0	1kit-d1kit_1 (2_19_1_6)	1lcl-d1lcl (2_19_1_3)	1cpn-d1cpn (2_19_1_2)	1ospo-d1ospo (2_52_1_1)	1mpmb-d1mal (6_7_1_2)	3nn9-d3nn9 (2_45_1_1)	1sacb-d1saca (2_19_1_4)	1eur-d1eur (2_45_1_1)	2bbkh-d2bbkh (2_46_2_1)
408	1knb-d1knb	2_16_1_1	186	1	0	0	0	0	0	0	1ggt-d1ggta2 (2_1_4_1)	1prn-d1prn (6_7_1_1)	2por-d2por (6_7_1_1)	1dih-d1dih_2 (4_41_1_2)	1sva5-d1sva1 (2_8_1_4)	1kit-d1kit_2 (2_19_1_6)	1mpmb-d1mal (6_7_1_2)	1ahsa-d1ahsa (2_14_1_1)	1ospo-d1ospo (2_52_1_1)
409	1knt-d1knt	7_6_1_1	55	3	1	1	0	0	0	0	1mtnh-d1bpi (7_6_1_1)	1bunb-d1bunb (7_6_1_1)	2mipa-d1idaa (2_34_1_1)	1aocb-d1aoca (7_14_1_5)	1clc-d1clc_2 (2_1_1_5)	1phk-d1phk (5_1_1_1)	2prd-d2prd (2_26_5_1)	1pgs-d1pgs_1 (2_11_1_1)	2hnp-d2hnp (3_32_1_2)
410	1knya-d1knya	5_10_1_2	253	1	0	0	0	0	0	0	1pgo-d2pgd_1 (1_71_1_1)	1occa-d1occa1 (6_5_1_1)	1xsm-d1xsm (1_24_1_2)	1mrrb-d1riba (1_24_1_2)	1occc-d1occc1 (6_5_1_1)	1prcl-d1prcl1 (6_5_1_1)	1zymb-d1zyma (3_5_1_2)	1csr-d1csh (1_74_1_1)	1bfra-d1bcfa (1_24_1_1)
411	1kob-d1koba	5_1_1_1	352	4	1	1	0	0	0	0	1phk-d1phk (5_1_1_1)	1apme-d1apme (5_1_1_1)	2csn-d1csn (5_1_1_1)	1qba-d1qba_3 (3_1_1_6)	1htda-d1atla (4_50_1_5)	1pda-d1pda_2 (4_28_2_1)	1gsa-d2glt_1 (3_20_1_3)	1dcha-d1dcoa (4_38_1_1)	1rcd-d1rcd (1_24_1_1)
412	1kpaa-d1kpaa	4_10_1_1	113	1	0	0	1	1	0	0	1hxp-d1hxpa2 (4_10_1_2)	1ltge-d1ltsd (2_26_2_1)	1qrda-d1qrda (3_13_4_2)	1mxb-d1mxa_3 (4_75_1_1)	1dora-d1dora (3_1_7_1)	1kob-d1koba (5_1_1_1)	1gtra-d1gtra2 (3_15_1_1)	1rpl-d1rpl (5_10_1_1)	1orta-d1orta1 (3_58_1_1)
413	1kraa-d2kaua	4_6_1_1	100	1	0	0	0	0	0	0	1mba-d1mba (1_1_1_1)	1vin-d1vin_1 (1_59_1_1)	1gtma-d1gtma1 (3_19_1_7)	1cowe-d1bmfd3 (3_25_1_6)	1occc-d1occc1 (6_5_1_1)	1emd-d2cmd_2 (4_92_1_1)	1buca-d1buca1 (1_23_6_1)	1hrda-d1hrda1 (3_19_1_7)	1eac-d1eaf (3_30_1_1)
414	1krcb-d2kaub	2_60_2_1	101	1	0	0	0	0	0	1	1aspb-d1aoza1 (2_5_1_3)	1duta-d1duta (2_60_3_1)	1kcw-d1kcw_1 (2_5_1_3)	1apa-d1apa (4_94_1_1)	1kcw-d1kcw_5 (2_5_1_3)	1dar-d1dar_1 (2_29_3_1)	1rdd-d2rn2 (3_41_3_1)	1gof-d1gof_3 (2_46_1_1)	2sil-d2sil (2_45_1_1)
415	1krn-d1pk4	7_11_1_1	79	1	0	0	0	0	0	0	1msad-d1msaa (2_54_1_1)	1ray-d2cba (2_50_1_1)	1mbt-d1mbb_2 (4_85_1_1)	1btga-d1bet (7_14_1_3)	1hgea-d1hgea (2_14_1_2)	1udh-d1udg (3_11_1_1)	1pgo-d2pgd_1 (1_71_1_1)	1mrj-d1mrj (4_94_1_1)	1thw-d1thw (2_18_1_1)
416	1kst-d1kst	7_17_1_1	68	1	0	0	0	0	0	0	2prd-d2prd (2_26_5_1)	1prch-d1prch1 (2_27_1_1)	1apa-d1apa (4_94_1_1)	1mbb-d1mbb_1 (4_84_1_2)	1knb-d1knb (2_16_1_1)	2aaa-d2aaa_1 (2_48_1_1)	1kob-d1koba (5_1_1_1)	1juy-d1adea (3_25_1_5)	2mtal-d2bbkl (7_19_1_1)
417	1kte-d1kte	3_33_1_1	105	4	1	1	0	0	0	0	1xoa-d2trxa (3_33_1_1)	1fjm-d1fjma (4_91_1_2)	1rnl-d1rnl_2 (3_13_2_1)	2chr-d2chr_1 (3_1_6_2)	1wsyb-d1wsyb (3_59_1_1)	2lbp-d2lbp (3_72_1_1)	1php-d1php (3_66_1_1)	1nis-d1aco_2 (3_63_1_1)	1gca-d1gca (3_72_1_1)
418	1kuh-d1kuh	4_50_1_1	132	1	0	0	0	1	0	0	1esd-d1esc (3_13_8_1)	1apme-d1apme (5_1_1_1)	2tcl-d1hfc (4_50_1_7)	1llda-d1llda2 (4_92_1_1)	1htda-d1atla (4_50_1_5)	1clc-d1clc_1 (1_73_1_2)	1lucb-d1lucb (3_1_13_1)	1btl-d1btl (5_4_1_1)	1ngi-d1hpm_2 (3_41_1_1)
419	1kxa-d2snv	2_31_1_3	151	1	0	0	0	1	0	0	1ospo-d1ospo (2_52_1_1)	2bbkh-d2bbkh (2_46_2_1)	1smpi-d1smpi (2_42_2_1)	1p03a-d2alp (2_31_1_1)	1arc-d1arb (2_31_1_1)	1gpr-d1gpr (2_59_3_1)	2hnp-d2hnp (3_32_1_2)	1prch-d1prch1 (2_27_1_1)	1gen-d1gen (2_44_1_1)
420	1kzua-d1kzua	6_6_1_1	47	4	1	1	0	0	0	0	1lgha-d1lgha (6_6_1_1)	1esd-d1esc (3_13_8_1)	1rcpa-d1cpq (1_23_3_2)	1mrrb-d1riba (1_24_1_2)	1lpe-d1lpe (1_23_1_1)	1dlc-d1dlc_3 (6_1_3_1)	1vsga-d1vsga (6_3_1_1)	1occc-d1occc1 (6_5_1_1)	1flp-d1flp (1_1_1_1)
421	1kzub-d1kzub	6_6_1_1	41	4	0	0	0	0	0	0	1zymb-d1zyma (3_5_1_2)	1sesa-d1seta1 (1_2_3_1)	1mrrb-d1riba (1_24_1_2)	1div-d1div (4_82_1_1)	2myd-d1mbd (1_1_1_1)	1vin-d1vin_1 (1_59_1_1)	1lki-d1lki (1_25_1_1)	2liga-d2liga (1_23_2_1)	1vsga-d1vsga (6_3_1_1)
422	1lac-d1lac	2_59_1_1	80	5	1	1	0	0	1	1	1iyu-d1iyu (2_59_1_1)	1ghj-d1ghk (2_59_1_1)	1bdo-d1bdo (2_59_1_1)	1hpca-d1htp (2_59_1_1)	1gpr-d1gpr (2_59_3_1)	1ouna-d1ouna (4_14_3_2)	2trcb-d2trcb (2_46_3_1)	1qapa-d1qapa2 (4_23_2_1)	1hpl-d1hpla1 (2_10_2_1)
423	1lam-d1lam_1	3_36_1_1	159	1	0	0	0	0	0	0	1gca-d1gca (3_72_1_1)	1llda-d1llda1 (3_19_1_5)	4xis-d2xis (3_1_12_1)	1lucb-d1lucb (3_1_13_1)	1nis-d1aco_2 (3_63_1_1)	1gky-d1gky (3_25_1_1)	1rvva-d1rvva (3_9_1_1)	2ctb-d2ctb (3_52_3_1)	1tys-d1tys (4_63_1_1)
424	1lba-d1lba	4_64_1_1	146	1	0	0	0	0	0	0	1obr-d1obr (3_52_3_2)	1vhh-d1vhh (4_34_1_2)	2vik-d2vik (4_60_1_1)	1nai-d1xel (3_19_1_2)	1jeva-d2olba (3_73_1_1)	1tiig-d1tiid (2_26_2_1)	1opr-d1opr (3_44_1_1)	1nal1-d1nal1 (3_1_3_1)	1coy-d1coy_1 (3_4_1_2)
425	1lbd-d1lbd	1_87_1_1	238	1	0	0	0	0	0	0	1sly-d1sly_1 (1_84_4_1)	1fps-d1fps (1_91_1_1)	1afra-d1afra (1_24_1_2)	1occa-d1occa1 (6_5_1_1)	1mrrb-d1riba (1_24_1_2)	1csr-d1csh (1_74_1_1)	1mtyd-d1mmod (1_24_1_2)	1occc-d1occc1 (6_5_1_1)	1faga-d2bmha (1_75_1_1)
426	1lbt-d1tca	3_50_1_7	317	3	0	0	1	1	0	0	1yasa-d1yasa (3_50_1_10)	1taha-d1tahb (3_50_1_8)	1broa-d1broa (3_50_1_5)	1xzl-d1cus (3_13_7_1)	2dhd-d1ede (3_50_1_3)	1rvva-d1rvva (3_9_1_1)	1fcdb-d1fcda2 (3_4_1_4)	1llo-d1hvq (3_1_1_5)	1eft-d1eft_3 (3_25_1_3)
427	1lbu-d1lbu_1	1_19_1_1	83	1	0	0	0	0	0	0	1febb-d2tpra2 (3_4_1_4)	2mnr-d2mnr_1 (3_1_6_2)	2chr-d2chr_1 (3_1_6_2)	2sas-d2sas (1_34_1_5)	1srrc-d1srra (3_13_2_1)	1gtma-d1gtma2 (3_54_1_1)	2dri-d2dri (3_72_1_1)	1hrda-d1hrda2 (3_54_1_1)	1osa-d1osa (1_34_1_5)
428	1lbu-d1lbu_2	4_34_1_1	130	1	0	0	1	1	0	0	1vhh-d1vhh (4_34_1_2)	1nox-d1nox (4_49_1_1)	1pnr-d1pnra2 (3_72_1_1)	1asza-d1asya2 (4_59_1_1)	1scha-d1scha (1_65_1_1)	1rcd-d1rcd (1_24_1_1)	4xis-d2xis (3_1_12_1)	1leha-d1leha2 (3_54_1_1)	1tlfa-d1tlfa (3_72_1_1)

429	1lcc-d1lcca	1_30_1_3	51	2	1	1	1	1	0	0	1bdi-d1pnra1 (1_30_1_3)	1llib-d1llia (1_30_1_2)	1dsba-d1dsba1 (1_37_1_1)	1ctf-d1ctf (4_26_1_1)	1rnl-d1rnl_1 (1_31_1_2)	1fps-d1fps (1_91_1_1)	1cxh-d1cdg_4 (3_1_1_1)	1occc-d1occc1 (6_5_1_1)	1ezm-d1ezm_1 (1_53_1_1)
430	1lcl-d1lcl	2_19_1_3	141	2	1	1	1	1	0	0	1slba-d1slaa (2_19_1_3)	1kit-d1kit_2 (2_19_1_6)	1sacb-d1saca (2_19_1_4)	3nn9-d3nn9 (2_45_1_1)	1cpn-d1cpn (2_19_1_2)	1ospo-d1ospo (2_52_1_1)	1kit-d1kit_1 (2_19_1_6)	1ten-d1ten (2_1_2_1)	1nsdb-d1nsca (2_45_1_1)
431	1ldl-d1ajj	7_9_1_1	37	2	1	1	0	0	0	0	1ldl-d1ajj (7_9_1_1)	1lbd-d1lbd (1_87_1_1)	1gym-d1gym (3_1_15_2)	1apme-d1apme (5_1_1_1)	1abrb-d1abrb2 (2_28_2_1)	2bpa1-d2bpa1 (2_8_1_1)	2bfh-d2fgf (2_28_1_1)	1nis-d1aco_2 (3_63_1_1)	1whi-d1whi (2_25_1_1)
432	1ldl-d1ldl	7_9_1_1	46	2	1	1	0	0	0	0	1ldl-d1ajj (7_9_1_1)	2bfh-d2fgf (2_28_1_1)	1whi-d1whi (2_25_1_1)	1nis-d1aco_2 (3_63_1_1)	1lbd-d1lbd (1_87_1_1)	1gym-d1gym (3_1_15_2)	3rnt-d9rnt (4_1_1_1)	1bll-d1lam_2 (3_52_3_3)	1apme-d1apme (5_1_1_1)
433	1lea-d1lea	1_4_3_2	72	1	0	0	0	0	0	0	1vin-d1vin_1 (1_59_1_1)	1vin-d1vin_2 (1_59_1_1)	1nfp-d1nfp (3_1_13_2)	1gln-d1gln_2 (3_15_1_1)	1dik-d1dik_3 (4_83_1_4)	1dpra-d1dpra1 (1_4_3_11)	1alo-d1alo_1 (1_47_1_1)	1wsyb-d1wsyb (3_59_1_1)	1pgo-d2pgd_1 (1_71_1_1)
434	1lef-d1lefa	1_20_1_1	86	4	0	1	0	0	0	0	1mtyd-d1mmod (1_24_1_2)	1aab-d1aab (1_20_1_1)	1mrrb-d1riba (1_24_1_2)	1ciy-d1ciy_3 (6_1_3_1)	1fps-d1fps (1_91_1_1)	1dpra-d1dpra2 (1_61_1_1)	1zymb-d1zyma (3_5_1_2)	1orda-d1orda2 (3_48_1_4)	1lucb-d1lucb (3_1_13_1)
435	1leha-d1leha1	3_19_1_7	230	3	1	1	1	1	0	0	1gtma-d1gtma1 (3_19_1_7)	1hrda-d1hrda1 (3_19_1_7)	2naca-d2naca2 (3_19_1_4)	1gdha-d1gdha2 (3_19_1_4)	1nal1-d1nal1 (3_1_3_1)	1dxy-d1dxy_2 (3_19_1_4)	2gpb-d1gpb (3_68_1_2)	1clc-d1clc_1 (1_73_1_2)	1llda-d1llda1 (3_19_1_5)
436	1leha-d1leha2	3_54_1_1	134	3	1	1	0	0	0	0	1gtma-d1gtma2 (3_54_1_1)	1hrda-d1hrda2 (3_54_1_1)	1tys-d1tys (4_63_1_1)	1pea-d1pea (3_72_1_1)	1ahia-d1fmca (3_19_1_2)	1pnr-d1pnra2 (3_72_1_1)	2ada-d1add (3_1_2_1)	1tlfa-d1tlfa (3_72_1_1)	1nal1-d1nal1 (3_1_3_1)
437	1lfb-d1lfb	1_4_1_1	77	5	1	1	0	0	0	0	1hddc-d1enh (1_4_1_1)	2scpa-d2scpa (1_34_1_5)	7icd-d7icd (3_57_1_1)	3sdha-d3sdha (1_1_1_1)	2liga-d2liga (1_23_2_1)	2cpo-d1cpo_2 (1_34_2_1)	1osa-d1osa (1_34_1_5)	1sly-d1sly_1 (1_84_4_1)	1lis-d1lis (1_17_1_1)
438	1lgha-d1lgha	6_6_1_1	56	4	0	1	0	0	0	0	1bbha-d1bbha (1_23_3_2)	1kzua-d1kzua (6_6_1_1)	1clc-d1clc_1 (1_73_1_2)	1ryt-d1ryt_1 (1_24_1_1)	1rpo-d1rpo (1_27_1_1)	1rcpa-d1cpq (1_23_3_2)	1occc-d1occc1 (6_5_1_1)	1asu-d1asu (3_41_3_2)	1rcd-d1rcd (1_24_1_1)
439	1lghb-d1lghb	6_6_1_1	43	4	0	0	0	0	0	0	1buca-d1buca1 (1_23_6_1)	1ciy-d1ciy_3 (6_1_3_1)	1sesa-d1seta1 (1_2_3_1)	1mof-d1mof (8_42_1_1)	1occc-d1occc1 (6_5_1_1)	1lpe-d1lpe (1_23_1_1)	1pgo-d2pgd_1 (1_71_1_1)	1itha-d1itha (1_1_1_1)	2hmgd-d1htmb (6_2_1_1)
440	1lh7-d1lh1	1_1_1_1	153	10	1	1	1	1	0	0	2myd-d1mbd (1_1_1_1)	1itha-d1itha (1_1_1_1)	1baba-d1baba (1_1_1_1)	1mba-d1mba (1_1_1_1)	3sdha-d3sdha (1_1_1_1)	2hbg-d1hbg (1_1_1_1)	1flp-d1flp (1_1_1_1)	1hlb-d1hlb (1_1_1_1)	2lhb-d2lhb (1_1_1_1)
441	1lis-d1lis	1_17_1_1	131	1	0	0	0	0	0	0	1lpe-d1lpe (1_23_1_1)	1ciy-d1ciy_3 (6_1_3_1)	1mmob-d1mmob (1_24_1_2)	3gsta-d2gsta1 (1_38_1_1)	1lki-d1lki (1_25_1_1)	1dlc-d1dlc_3 (6_1_3_1)	1gsea-d1gsea1 (1_38_1_1)	1sly-d1sly_1 (1_84_4_1)	1osa-d1osa (1_34_1_5)
442	1lki-d1lki	1_25_1_1	172	1	0	0	0	0	0	0	1bfra-d1bcfa (1_24_1_1)	1mrrb-d1riba (1_24_1_2)	1occa-d1occa1 (6_5_1_1)	1rcd-d1rcd (1_24_1_1)	1occc-d1occc1 (6_5_1_1)	1lh7-d1lh1 (1_1_1_1)	1dlc-d1dlc_3 (6_1_3_1)	1afra-d1afra (1_24_1_2)	1ryt-d1ryt_1 (1_24_1_1)
443	1llda-d1llda1	3_19_1_5	143	4	1	1	1	1	0	0	6ldh-d1ldm_1 (3_19_1_5)	1emd-d2cmd_1 (3_19_1_5)	1hlpa-d1hlpa1 (3_19_1_5)	1dhr-d1dhr (3_19_1_2)	1leha-d1leha1 (3_19_1_7)	1cyda-d1cyda (3_19_1_2)	2dri-d2dri (3_72_1_1)	2chr-d2chr_1 (3_1_6_2)	1ahia-d1fmca (3_19_1_2)
444	1llda-d1llda2	4_92_1_1	170	5	1	1	0	0	0	0	6ldh-d1ldm_2 (4_92_1_1)	1hlpa-d1hlpa2 (4_92_1_1)	1bdmb-d1bdma2 (4_92_1_1)	1emd-d2cmd_2 (4_92_1_1)	2tmda-d2tmda1 (3_1_7_1)	1hxp-d1hxpa2 (4_10_1_2)	1hrda-d1hrda1 (3_19_1_7)	4xis-d2xis (3_1_12_1)	1hpm-d1hpm_1 (3_41_1_1)
445	1llib-d1llia	1_30_1_2	89	5	1	1	0	0	0	0	1adr-d1adr (1_30_1_2)	2sas-d2sas (1_34_1_5)	1perl-d1r69 (1_30_1_2)	2abk-d2abk (1_66_1_1)	1osa-d1osa (1_34_1_5)	2wrpr-d2wrpr (1_78_1_1)	1fps-d1fps (1_91_1_1)	1tcob-d1tcob (1_34_1_5)	1yrnb-d1yrnb (1_4_1_1)
446	1llo-d1hvq	3_1_1_5	273	3	0	1	0	0	0	0	1mioa-d1mioa (3_67_1_1)	1tlfa-d1tlfa (3_72_1_1)	1yasa-d1yasa (3_50_1_10)	1tkaa-d1trka1 (3_24_1_2)	1edt-d1edt (3_1_1_5)	1hmp-d1hmpa (3_44_1_1)	1lbt-d1tca (3_50_1_7)	2mnr-d2mnr_1 (3_1_6_2)	1nfp-d1nfp (3_1_13_2)
447	1loi-d1loi	8_45_1_1	25	1	0	0	0	0	0	0	3mdsa-d1mnga2 (4_25_1_1)	1occa-d1occa1 (6_5_1_1)	3pte-d3pte (5_4_1_1)	1inp-d1inp (5_8_1_3)	7cat-d7cata (5_6_1_1)	1zymb-d1zyma (3_5_1_2)	1pne-d1pne (4_61_1_1)	2erl-d1erc (1_10_1_1)	2ada-d1add (3_1_2_1)
448	1lpe-d1lpe	1_23_1_1	144	1	0	0	0	0	0	1	1occc-d1occc1 (6_5_1_1)	1aep-d1aep (1_49_1_1)	1mrrb-d1riba (1_24_1_2)	1bfra-d1bcfa (1_24_1_1)	1buca-d1buca1 (1_23_6_1)	1occa-d1occa1 (6_5_1_1)	2liga-d2liga (1_23_2_1)	1ciy-d1ciy_3 (6_1_3_1)	1afra-d1afra (1_24_1_2)
449	1lpt-d1lpt	1_42_1_1	90	2	0	0	0	0	0	0	1phe-d1phb (1_75_1_1)	2aaa-d2aaa_2 (3_1_1_1)	1mmob-d1mmob (1_24_1_2)	1juy-d1adea (3_25_1_5)	1hry-d1hrza (1_20_1_1)	1cxh-d1cdg_4 (3_1_1_1)	1bmta-d1bmta1 (1_39_1_1)	1lucb-d1lucb (3_1_13_1)	1buca-d1buca2 (5_7_1_1)
450	1lrv-d1lrv	1_84_2_1	233	1	0	0	0	0	0	0	2hbg-d1hbg (1_1_1_1)	1hvd-d1hvd (1_51_1_1)	2scpa-d2scpa (1_34_1_5)	1occa-d1occa1 (6_5_1_1)	2sas-d2sas (1_34_1_5)	1oxa-d1oxa (1_75_1_1)	2mnr-d2mnr_2 (4_31_1_1)	1gbs-d1gbs (4_2_1_4)	2myd-d1mbd (1_1_1_1)
451	1lst-d1lst	3_73_1_1	238	8	1	1	0	0	0	0	1ggga-d1ggga (3_73_1_1)	1qora-d1qora2 (3_19_1_1)	1dxy-d1dxy_2 (3_19_1_4)	1kfd-d1kfd_1 (3_41_3_4)	1oiba-d1pbp (3_73_1_1)	1tlfa-d1tlfa (3_72_1_1)	1dppc-d1dppa (3_73_1_1)	1pgn-d2pgd_2 (3_19_1_6)	1jeva-d2olba (3_73_1_1)
452	1ltge-d1ltsd	2_26_2_1	103	6	1	1	0	0	0	0	1tiig-d1tiid (2_26_2_1)	1ytfa-d1ytba1 (4_74_1_1)	1prtf-d1prtf (2_26_2_1)	2tmda-d2tmda1 (3_1_7_1)	2pola-d2pola3 (4_76_1_1)	1kpaa-d1kpaa (4_10_1_1)	1hjra-d1hjra (3_41_3_6)	1rvea-d1rvaa (3_38_1_2)	1napd-d1napa (4_7_1_1)
453	1lucb-d1lucb	3_1_13_1	320	1	0	0	1	1	0	1	1nfp-d1nfp (3_1_13_2)	1gtra-d1gtra2 (3_15_1_1)	1miob-d1miob (3_67_1_1)	7icd-d7icd (3_57_1_1)	1xyza-d1xyza (3_1_1_3)	1dik-d1dik_3 (4_83_1_4)	1clc-d1clc_1 (1_73_1_2)	1gca-d1gca (3_72_1_1)	2dri-d2dri (3_72_1_1)
454	1lvl-d1lvl_2	3_4_1_4	115	6	1	1	1	1	0	0	1geua-d1gesa2 (3_4_1_4)	2tmda-d2tmda2 (3_4_1_1)	1nhq-d1nhp_2 (3_4_1_4)	1febb-d2tpra2 (3_4_1_4)	1tde-d1tde_2 (3_4_1_4)	1bncb-d1bnca2 (3_20_1_1)	1coy-d1coy_1 (3_4_1_2)	1hrda-d1hrda1 (3_19_1_7)	1scua-d1scua2 (3_19_1_8)
455	1lvl-d1lvl_3	4_46_1_1	123	5	1	1	0	0	0	0	1fead-d2tpra3 (4_46_1_1)	1gesa-d1gesa3 (4_46_1_1)	2npx-d1nhp_3 (4_46_1_1)	2bbkh-d2bbkh (2_46_2_1)	4aahc-d4aaha (2_47_1_1)	1lst-d1lst (3_73_1_1)	1ecpa-d1ecpa (3_52_1_1)	1alo-d1alo_4 (4_77_1_1)	1gdob-d1gdoa (4_88_1_1)
456	1lxa-d1lxa	2_57_1_1	262	1	0	0	1	1	0	0	1tdta-d1tdta (2_57_1_2)	1osa-d1osa (1_34_1_5)	1sly-d1sly_1 (1_84_4_1)	1itha-d1itha (1_1_1_1)	1dik-d1dik_3 (4_83_1_4)	7cat-d7cata (5_6_1_1)	1runb-d1cgpa2 (2_58_3_1)	1gbs-d1gbs (4_2_1_4)	1p03a-d2alp (2_31_1_1)
457	1lxta-d3pmga4	4_74_2_1	141	1	0	0	0	0	0	0	1eac-d1eaf (3_30_1_1)	1ospo-d1ospo (2_52_1_1)	2hnp-d2hnp (3_32_1_2)	2sil-d2sil (2_45_1_1)	1pne-d1pne (4_61_1_1)	1dkza-d1dkza (5_17_1_1)	1alo-d1alo_7 (4_77_1_1)	2hmb-d1hms (2_41_1_2)	1ure-d1ifc (2_41_1_2)
458	1lyla-d1lyla2	4_59_1_1	342	3	1	1	0	0	0	0	1asza-d1asya2 (4_59_1_1)	1fbaa-d1fbaa (3_1_3_1)	1rpl-d1rpl (5_10_1_1)	1ecpa-d1ecpa (3_52_1_1)	1btl-d1btl (5_4_1_1)	1pea-d1pea (3_72_1_1)	1lucb-d1lucb (3_1_13_1)	1dik-d1dik_3 (4_83_1_4)	2ada-d1add (3_1_2_1)
459	1lylb-d1lyla1	2_26_4_1	140	2	1	1	0	0	0	1	1asya-d1asya1 (2_26_4_1)	1gdob-d1gdoa (4_88_1_1)	1qila-d1tssa1 (2_26_2_2)	4aahc-d4aaha (2_47_1_1)	2pola-d2pola3 (4_76_1_1)	1ltge-d1ltsd (2_26_2_1)	1rpa-d1rpa (3_43_1_2)	1chma-d1chma1 (3_41_2_1)	1mpmb-d1mal (6_7_1_2)
460	1lyp-d1lyp	8_16_1_1	32	1	0	0	0	0	0	0	1ifl-d1ifl (1_97_3_1)	6ldh-d1ldm_1 (3_19_1_5)	1mmob-d1mmob (1_24_1_2)	1grj-d1grj_1 (1_2_1_1)	1buca-d1buca1 (1_23_6_1)	1mdya-d1mdya (1_33_1_1)	1pgo-d2pgd_1 (1_71_1_1)	1occc-d1occc1 (6_5_1_1)	1occa-d1occa1 (6_5_1_1)
461	1mai-d1mai	2_37_1_1	119	5	1	1	0	0	0	0	1pls-d1pls (2_37_1_1)	1mpmb-d1mal (6_7_1_2)	1gcb-d1gcb (4_3_1_1)	1gdob-d1gdoa (4_88_1_1)	1dnpa-d1dnpa2 (3_17_1_1)	1asya-d1asya1 (2_26_4_1)	1plq-d1plq_1 (4_76_1_2)	1noy-d1noya (3_41_3_5)	1prn-d1prn (6_7_1_1)
462	1mba-d1mba	1_1_1_1	145	10	1	1	1	1	0	0	2lhb-d2lhb (1_1_1_1)	1itha-d1itha (1_1_1_1)	3sdha-d3sdha (1_1_1_1)	2myd-d1mbd (1_1_1_1)	1hlb-d1hlb (1_1_1_1)	1flp-d1flp (1_1_1_1)	2hbg-d1hbg (1_1_1_1)	1lh7-d1lh1 (1_1_1_1)	1baba-d1baba (1_1_1_1)
463	1mbb-d1mbb_1	4_84_1_2	198	1	0	0	0	0	0	0	1eac-d1eaf (3_30_1_1)	1apa-d1apa (4_94_1_1)	1pne-d1pne (4_61_1_1)	1han-d1han_1 (4_20_1_3)	1gof-d1gof_3 (2_46_1_1)	1acf-d1acf (4_61_1_1)	1gph1-d1gph11 (3_44_1_1)	1chma-d1chma2 (4_72_1_1)	2dri-d2dri (3_72_1_1)
464	1mbk-d1msec2	1_4_1_3	50	2	0	0	0	1	0	0	1hvd-d1hvd (1_51_1_1)	1fipa-d1fipa (1_76_1_1)	1gdz-d1ak4c (1_57_1_1)	1phe-d1phb (1_75_1_1)	1hddc-d1enh (1_4_1_1)	1hrda-d1hrda1 (3_19_1_7)	1bfma-d1bfma (1_21_1_2)	1vid-d1vid (3_47_1_1)	1sly-d1sly_1 (1_84_4_1)
465	1mbt-d1mbb_2	4_85_1_1	142	1	0	0	0	0	0	0	1asza-d1asya2 (4_59_1_1)	1hpm-d1hpm_1 (3_41_1_1)	1bya-d1byb (3_1_1_2)	1fbaa-d1fbaa (3_1_3_1)	2tmda-d2tmda1 (3_1_7_1)	1akl-d1kapp2 (4_50_1_6)	1tlfa-d1tlfa (3_72_1_1)	1onea-d1ebha1 (3_1_6_1)	1seta-d1seta2 (4_59_1_1)
466	1mcti-d1mcti	7_3_2_1	28	1	0	0	0	0	0	0	1kiqa-d1vfba (2_1_1_1)	3rnt-d9rnt (4_1_1_1)	1fca-d1fca (4_33_1_1)	1rcf-d1rcf (3_13_4_1)	1iad-d1ast (4_50_1_4)	1rie-d1rie (7_33_1_2)	1gur-d1gur (7_3_4_1)	2sn3-d2sn3 (7_3_6_1)	1mbb-d1mbb_1 (4_84_1_2)
467	1mdya-d1mdya	1_33_1_1	68	1	0	0	0	0	0	0	1hula-d1hula (1_25_1_2)	1prcl-d1prcl1 (6_5_1_1)	1flp-d1flp (1_1_1_1)	1osa-d1osa (1_34_1_5)	1msk-d1msk (4_99_1_1)	1pgo-d2pgd_1 (1_71_1_1)	1afra-d1afra (1_24_1_2)	1xsm-d1xsm (1_24_1_2)	1aep-d1aep (1_49_1_1)
468	1mek-d1mek	3_33_1_2	120	1	0	0	1	1	0	0	1xoa-d2trxa (3_33_1_1)	1poxa-d1poxa3 (3_24_1_1)	1llo-d1hvq (3_1_1_5)	1pyda-d1pvda3 (3_24_1_1)	1kte-d1kte (3_33_1_1)	1egr-d1ego (3_33_1_1)	1prtf-d1prtf (2_26_2_1)	2dri-d2dri (3_72_1_1)	1cowe-d1bmfd3 (3_25_1_6)
469	1mioa-d1mioa	3_67_1_1	525	2	1	1	0	0	0	0	1miob-d1miob (3_67_1_1)	2bnh-d1dfji (3_7_1_1)	2dri-d2dri (3_72_1_1)	1gca-d1gca (3_72_1_1)	1dhpa-d1dhpa (3_1_3_1)	1gtma-d1gtma1 (3_19_1_7)	1nal1-d1nal1 (3_1_3_1)	1llo-d1hvq (3_1_1_5)	1dora-d1dora (3_1_7_1)
470	1miob-d1miob	3_67_1_1	457	2	1	1	0	0	0	0	1mioa-d1mioa (3_67_1_1)	1gca-d1gca (3_72_1_1)	2dri-d2dri (3_72_1_1)	2bnh-d1dfji (3_7_1_1)	1igs-d1igs (3_1_8_1)	1tlfa-d1tlfa (3_72_1_1)	1lucb-d1lucb (3_1_13_1)	1pnr-d1pnra2 (3_72_1_1)	1nal1-d1nal1 (3_1_3_1)
471	1mit-d1tin	4_22_1_1	68	3	1	1	0	0	0	0	1coa-d1coai (4_22_1_1)	1csei-d1csei (4_22_1_1)	1mrj-d1mrj (4_94_1_1)	2pia-d2pia_1 (2_29_1_2)	1eri-d1eria (3_38_1_1)	2phl-d2phla1 (2_58_1_1)	1esd-d1esc (3_13_8_1)	1eny-d1eny (3_19_1_2)	2bbvc-d2bbva (2_8_1_3)
472	1mjc-d1mjc	2_26_4_4	69	2	0	1	0	0	0	0	1ospo-d1ospo (2_52_1_1)	1sro-d1sro (2_26_4_4)	3ait-d1hoe (2_4_1_1)	1arc-d1arb (2_31_1_1)	1tnj-d1bty (2_31_1_2)	2sil-d2sil (2_45_1_1)	1vmoa-d1vmoa (2_53_1_1)	1p03a-d2alp (2_31_1_1)	1iob-d1i1b (2_28_1_2)
473	1mkaa-d1mkaa	4_21_1_1	171	1	0	0	0	0	0	0	1ouna-d1ouna (4_14_3_2)	1cpn-d1cpn (2_19_1_2)	1mola-d1mola (4_14_1_1)	1bbpa-d1bbpa (2_41_1_1)	1lcl-d1lcl (2_19_1_3)	1ytn-d1ypta (3_32_1_2)	1xva-d1xvaa (3_47_1_2)	1chma-d1chma2 (4_72_1_1)	1cew-d1cewi (4_14_1_2)
474	1mla-d1mla_2	4_33_17_1	70	1	0	0	0	0	0	0	1febb-d2tpra2 (3_4_1_4)	1mxa-d1mxa_1 (4_75_1_1)	1scub-d1scub1 (3_13_3_1)	1oya-d1oya (3_1_7_1)	1hpm-d1hpm_1 (3_41_1_1)	1mml-d1mml (5_9_1_2)	2tmda-d2tmda1 (3_1_7_1)	2dkb-d2dkb (3_48_1_3)	1vhia-d1vhia (4_33_8_1)
475	1mml-d1mml	5_9_1_2	251	1	0	0	0	0	0	0	1emd-d2cmd_1 (3_19_1_5)	3tgl-d3tgl (3_50_1_7)	1nis-d1aco_2 (3_63_1_1)	2dkb-d2dkb (3_48_1_3)	1phk-d1phk (5_1_1_1)	1mba-d1mba (1_1_1_1)	1lyla-d1lyla2 (4_59_1_1)	1art-d1art (3_48_1_1)	1gca-d1gca (3_72_1_1)
476	1mmob-d1mmol	1_24_1_2	384	5	1	1	0	0	0	0	1mtyd-d1mmod (1_24_1_2)	1mrrb-d1riba (1_24_1_2)	1occa-d1occa1 (6_5_1_1)	1xsm-d1xsm (1_24_1_2)	1ciy-d1ciy_3 (6_1_3_1)	1dlc-d1dlc_3 (6_1_3_1)	1fps-d1fps (1_91_1_1)	1afra-d1afra (1_24_1_2)	1sly-d1sly_1 (1_84_4_1)

477	1mne-d1mmd_1	2_21_3_1	46	1	0	0	0	0	0	0	1gdob-d1gdoa (4_88_1_1)	2prd-d2prd (2_26_5_1)	1qila-d1tssa1 (2_26_2_2)	2hnp-d2hnp (3_32_1_2)	1phk-d1phk (5_1_1_1)	1wba-d1wba (2_28_3_1)	1gof-d1gof_3 (2_46_1_1)	1kit-d1kit_2 (2_19_1_6)	2bfh-d2fgf (2_28_1_1)
478	1mnta-d1mnta	1_36_1_1	66	2	0	0	0	0	0	0	1div-d1div (4_82_1_1)	1bip-d1bip (1_42_1_2)	1rgs-d1rgs_2 (2_58_3_2)	1tcob-d1tcob (1_34_1_5)	1rnl-d1rnl_2 (3_13_2_1)	1vin-d1vin_1 (1_59_1_1)	1poc-d1poc (1_95_1_1)	1pdo-d1pdo (3_40_1_1)	1occc-d1occc1 (6_5_1_1)
479	1mof-d1mof	8_42_1_1	53	1	0	0	0	0	0	0	1dkza-d1dkza (5_17_1_1)	1ecma-d1ecma (1_93_1_1)	1lghb-d1lghb (6_6_1_1)	1vsga-d1vsga (6_3_1_1)	1aep-d1aep (1_49_1_1)	1ifi-d1ifj (1_97_3_1)	1fosf-d1fosf (1_97_2_1)	1fose-d1fose (1_97_2_1)	1bfra-d1bcfa (1_24_1_1)
480	1mola-d1mola	4_14_1_1	94	1	0	0	1	1	1	1	1stfi-d1stfi (4_14_1_2)	1cew-d1cewi (4_14_1_2)	1ouna-d1ouna (4_14_3_2)	1mkaa-d1mkaa (4_21_1_1)	1ggt-d1ggta2 (2_1_4_1)	1fem-d1hbp (2_41_1_1)	1xva-d1xvaa (3_47_1_2)	1oaca-d1oaca2 (4_14_2_1)	1seia-d1seia (4_80_1_1)
481	1mpd-d1mpb	3_73_1_1	370	8	1	1	0	0	0	0	1poy1-d1pot (3_73_1_1)	1sbp-d1sbp (3_73_1_1)	2dri-d2dri (3_72_1_1)	1oiba-d1pbp (3_73_1_1)	1rcf-d1rcf (3_13_4_1)	1tkca-d1trka3 (3_34_1_1)	1gca-d1gca (3_72_1_1)	1tlfa-d1tlfa (3_72_1_1)	1cyda-d1cyda (3_19_1_2)
482	1mpmb-d1mal	6_7_1_2	421	1	0	0	1	1	0	0	2omf-d2omf (6_7_1_1)	1ospo-d1ospo (2_52_1_1)	2por-d2por (6_7_1_1)	1prn-d1prn (6_7_1_1)	1con-d1scs (2_19_1_1)	1ggt-d1ggta2 (2_1_4_1)	1obpa-d1obpa (2_41_1_1)	1nsdb-d1nsca (2_45_1_1)	1kit-d1kit_2 (2_19_1_6)
483	1mrj-d1mrj	4_94_1_1	247	3	1	1	0	0	0	0	1abra-d1abra (4_94_1_1)	1apa-d1apa (4_94_1_1)	1gcb-d1gcb (4_3_1_1)	1faga-d2bmha (1_75_1_1)	1prcl-d1prcl1 (6_5_1_1)	2hnp-d2hnp (3_32_1_2)	1oxa-d1oxa (1_75_1_1)	1agre-d1agre (1_64_1_1)	2trcb-d2trcb (2_46_3_1)
484	1mrrb-d1riba	1_24_1_2	340	5	1	1	0	1	0	0	1xsm-d1xsm (1_24_1_2)	1occc-d1occc1 (6_5_1_1)	1mmob-d1mmob (1_24_1_2)	1occa-d1occa1 (6_5_1_1)	1afra-d1afra (1_24_1_2)	1ciy-d1ciy_3 (6_1_3_1)	1mtyd-d1mmod (1_24_1_2)	1hvd-d1hvd (1_51_1_1)	1ryt-d1ryt_1 (1_24_1_1)
485	1msad-d1msaa	2_54_1_1	109	1	0	0	0	0	0	0	2trcb-d2trcb (2_46_3_1)	1ospo-d1ospo (2_52_1_1)	1gof-d1gof_3 (2_46_1_1)	2bfh-d2fgf (2_28_1_1)	4aahc-d4aaha (2_47_1_1)	1scb-d1csee (3_28_1_1)	1qila-d1tssa1 (2_26_2_2)	1noa-d1noa (2_1_6_1)	1tdta-d1tdta (2_57_1_2)
486	1msec-d1msec1	1_4_1_3	55	2	0	0	1	1	0	0	1igna-d1igna1 (1_4_1_5)	2scpa-d2scpa (1_34_1_5)	1tyc-d2ts1_1 (1_54_1_1)	2sas-d2sas (1_34_1_5)	1prhb-d1prha1 (1_65_1_2)	1sbp-d1sbp (3_73_1_1)	1nis-d1aco_2 (3_63_1_1)	1dpra-d1dpra2 (1_61_1_1)	1sly-d1sly_2 (4_2_1_5)
487	1msk-d1msk	4_99_1_1	327	1	0	0	0	0	0	0	1occc-d1occc1 (6_5_1_1)	1dyna-d1dyna (2_37_1_1)	1bfra-d1bcfa (1_24_1_1)	1afra-d1afra (1_24_1_2)	2wrpr-d2wrpr (1_78_1_1)	1prcl-d1prcl1 (6_5_1_1)	1art-d1art (3_48_1_1)	1pgo-d2pgd_1 (1_71_1_1)	1mdya-d1mdya (1_33_1_1)
488	1mtnh-d1bpi	7_6_1_1	58	3	1	1	0	0	0	0	1knt-d1knt (7_6_1_1)	1bunb-d1bunb (7_6_1_1)	1aocb-d1aoca (7_14_1_5)	2hnp-d2hnp (3_32_1_2)	4aahc-d4aaha (2_47_1_1)	1phk-d1phk (5_1_1_1)	1scub-d1scub2 (4_83_1_3)	1eur-d1eur (2_45_1_1)	3tgl-d3tgl (3_50_1_7)
489	1mtyd-d1mmod	1_24_1_2	512	5	1	1	0	0	0	0	1mmob-d1mmob (1_24_1_2)	1xsm-d1xsm (1_24_1_2)	1occa-d1occa1 (6_5_1_1)	1occc-d1occc1 (6_5_1_1)	1mrrb-d1riba (1_24_1_2)	1fps-d1fps (1_91_1_1)	1dlc-d1dlc_3 (6_1_3_1)	1sly-d1sly_1 (1_84_4_1)	1hvd-d1hvd (1_51_1_1)
490	1mtyg-d1mmog	1_22_1_1	162	1	0	0	0	0	0	0	1mmob-d1mmob (1_24_1_2)	2myd-d1mbd (1_1_1_1)	1fps-d1fps (1_91_1_1)	1occc-d1occc1 (6_5_1_1)	1ciy-d1ciy_3 (6_1_3_1)	1alla-d1alla (1_1_1_2)	1occa-d1occa1 (6_5_1_1)	1cpca-d1cpca (1_1_1_2)	1mrrb-d1riba (1_24_1_2)
491	1mut-d1mut	4_62_1_1	129	1	0	0	0	0	0	0	1dlc-d1dlc_2 (2_53_2_1)	1asqa-d1aoza2 (2_5_1_3)	1wit-d1wiu (2_1_1_4)	2pec-d2pec (2_56_1_1)	1asob-d1aoza3 (2_5_1_3)	1qba-d1qba_1 (2_1_1_5)	3nn9-d3nn9 (2_45_1_1)	2omf-d2omf (6_7_1_1)	2bpa1-d2bpa1 (2_8_1_1)
492	1mxa-d1mxa_1	4_75_1_1	101	3	0	0	0	0	0	0	1phk-d1phk (5_1_1_1)	2dri-d2dri (3_72_1_1)	1fcdb-d1fcda2 (3_4_1_4)	1dik-d1dik_3 (4_83_1_4)	1dcha-d1dcoa (4_38_1_1)	7icd-d7icd (3_57_1_1)	1kob-d1koba (5_1_1_1)	2csn-d1csn (5_1_1_1)	1sbp-d1sbp (3_73_1_1)
493	1mxb-d1mxa_3	4_75_1_1	152	3	0	0	0	0	0	0	2ada-d1add (3_1_2_1)	1taha-d1tahb (3_50_1_8)	1fbaa-d1fbaa (3_1_3_1)	1cew-d1cewi (4_14_1_2)	2tmda-d2tmda1 (3_1_7_1)	1obr-d1obr (3_52_3_2)	2chr-d2chr_1 (3_1_6_2)	1ryt-d1ryt_1 (1_24_1_1)	1kpaa-d1kpaa (4_10_1_1)
494	1myka-d1myka	1_36_1_1	47	2	0	0	0	0	0	0	1tfe-d1tfe (4_24_1_1)	1gky-d1gky (3_25_1_1)	1jkw-d1jkw_2 (1_59_1_1)	1gtma-d1gtma1 (3_19_1_7)	1jud-d1jud (5_18_1_1)	1flp-d1flp (1_1_1_1)	2cpo-d1cpo_1 (1_34_2_1)	1gln-d1gln_1 (1_67_1_1)	1chka-d1chka (4_2_1_6)
495	1nai-d1xel	3_19_1_2	338	6	1	1	0	0	0	0	1hdca-d1hdca (3_19_1_2)	1ahia-d1fmca (3_19_1_2)	1cyda-d1cyda (3_19_1_2)	1dhr-d1dhr (3_19_1_2)	4xis-d2xis (3_1_12_1)	1eny-d1eny (3_19_1_2)	2dri-d2dri (3_72_1_1)	2ada-d1add (3_1_2_1)	1tlfa-d1tlfa (3_72_1_1)
496	1nal1-d1nal1	3_1_3_1	291	3	1	1	0	0	1	1	1dhpa-d1dhpa (3_1_3_1)	4xis-d2xis (3_1_12_1)	1igs-d1igs (3_1_8_1)	1art-d1art (3_48_1_1)	1pgo-d2pgd_1 (1_71_1_1)	2mnr-d2mnr_1 (3_1_6_2)	2chr-d2chr_1 (3_1_6_2)	1pii-d1pii_2 (3_1_8_1)	7icd-d7icd (3_57_1_1)
497	1napd-d1napa	4_7_1_1	66	3	1	1	0	0	0	0	1dona-d1dona (4_7_1_1)	1ltge-d1ltsd (2_26_2_1)	1tiig-d1tiid (2_26_2_1)	1eri-d1eria (3_38_1_1)	1kit-d1kit_2 (2_19_1_6)	1huma-d1huma (4_7_1_1)	1ytba-d1ytba2 (4_74_1_1)	1gdob-d1gdoa (4_88_1_1)	1ctt-d1ctt_1 (3_75_1_1)
498	1nbaa-d1nbaa	3_22_1_1	253	1	0	0	0	0	0	0	1miob-d1miob (3_67_1_1)	4xis-d2xis (3_1_12_1)	1esd-d1esc (3_13_8_1)	1orta-d1orta1 (3_58_1_1)	1cyda-d1cyda (3_19_1_2)	1pnr-d1pnra2 (3_72_1_1)	1cxh-d1cdg_4 (3_1_1_1)	2ebn-d2ebn (3_1_1_5)	2dri-d2dri (3_72_1_1)
499	1ncs-d1ncs	7_28_1_1	47	10	0	0	0	0	0	0	1vsga-d1vsga (6_3_1_1)	1alo-d1alo_6 (4_77_1_1)	1hpl-d1hpla1 (2_10_2_1)	1bll-d1lam_2 (3_52_3_3)	2btf-d2btfa2 (3_41_1_1)	1apme-d1apme (5_1_1_1)	1ggt-d1ggta4 (4_3_1_2)	1rof-d1vjw (4_33_1_4)	1ecpa-d1ecpa (3_52_1_1)
500	1neq-d1ner	1_30_1_2	74	5	1	1	0	0	0	0	1llib-d1llia (1_30_1_2)	1bia-d1bia_1 (1_4_3_1)	1adr-d1adr (1_30_1_2)	1rnl-d1rnl_1 (1_31_1_2)	1lvl-d1lvl_3 (4_46_1_1)	1pdn-d1pdnc (1_4_1_4)	2scpa-d2scpa (1_34_1_5)	1copd-d1copd (1_30_1_2)	1lxa-d1lxa (2_57_1_1)
501	1nfp-d1nfp	3_1_13_2	228	1	0	0	1	1	1	1	1lucb-d1lucb (3_1_13_1)	1oya-d1oya (3_1_7_1)	1xyza-d1xyza (3_1_1_3)	1gky-d1gky (3_25_1_1)	1dik-d1dik_3 (4_83_1_4)	4xis-d2xis (3_1_12_1)	1fbaa-d1fbaa (3_1_3_1)	1bfra-d1bcfa (1_24_1_1)	1afra-d1afra (1_24_1_2)
502	1ngi-d1hpm_2	3_41_1_1	193	4	0	1	0	0	1	1	1hjra-d1hjra (3_41_3_6)	1clc-d1clc_1 (1_73_1_2)	2btf-d2btfa2 (3_41_1_1)	2ms2a-d2ms2a (4_45_1_1)	1pxt-d1pxta2 (3_74_1_1)	1dik-d1dik_3 (4_83_1_4)	4xis-d2xis (3_1_12_1)	1lucb-d1lucb (3_1_13_1)	2dri-d2dri (3_72_1_1)
503	1nhq-d1nhp_2	3_4_1_4	123	6	1	1	1	1	0	0	1geua-d1gesa2 (3_4_1_4)	1lvl-d1lvl_2 (3_4_1_4)	1febb-d2tpra2 (3_4_1_4)	2tmda-d2tmda2 (3_4_1_1)	1tde-d1tde_2 (3_4_1_4)	1wsyb-d1wsyb (3_59_1_1)	2dri-d2dri (3_72_1_1)	1dxy-d1dxy_2 (3_19_1_4)	1pgn-d2pgd_2 (3_19_1_6)
504	1nipb-d1nipa	3_25_1_5	283	2	0	0	0	0	0	0	1tlfa-d1tlfa (3_72_1_1)	1ahia-d1fmca (3_19_1_2)	1bya-d1byb (3_1_1_2)	1gca-d1gca (3_72_1_1)	1bll-d1lam_2 (3_52_3_3)	1mpd-d1mpb (3_73_1_1)	1iow-d2dln_1 (3_20_1_2)	1qapa-d1qapa1 (3_1_14_1)	2dri-d2dri (3_72_1_1)
505	1nis-d1aco_2	3_63_1_1	527	1	0	0	0	0	0	0	1tlfa-d1tlfa (3_72_1_1)	2dri-d2dri (3_72_1_1)	1fbaa-d1fbaa (3_1_3_1)	1gln-d1gln_2 (3_15_1_1)	1php-d1php (3_66_1_1)	1art-d1art (3_48_1_1)	1amp-d1amp (3_52_3_4)	1igs-d1igs (3_1_8_1)	2ora-d1orb_1 (3_60_1_1)
506	1noa-d1noa	2_1_6_1	113	1	0	0	0	0	0	1	1ospo-d1ospo (2_52_1_1)	1gog-d1gof_1 (2_1_1_5)	1obpa-d1obpa (2_41_1_1)	1wba-d1wba (2_28_3_1)	2bfh-d2fgf (2_28_1_1)	2avia-d2avia (2_42_1_1)	1fnc-d1fnb_1 (2_29_1_1)	2pcd-d2pcda (2_3_3_1)	1hpl-d1hpla1 (2_10_2_1)
507	1nox-d1nox	4_49_1_1	200	1	0	0	0	0	0	0	1taha-d1tahb (3_50_1_8)	1rcd-d1rcd (1_24_1_1)	1dik-d1dik_1 (3_1_9_2)	1oxa-d1oxa (1_75_1_1)	1pgo-d2pgd_1 (1_71_1_1)	1afra-d1afra (1_24_1_2)	1igs-d1igs (3_1_8_1)	1prcl-d1prcl1 (6_5_1_1)	2gpb-d1gpb (3_68_1_2)
508	1noy-d1noya	3_41_3_5	372	1	0	0	0	0	0	0	7icd-d7icd (3_57_1_1)	1knya-d1knya (5_10_1_2)	1gcb-d1gcb (4_3_1_1)	1leha-d1leha1 (3_19_1_7)	1dik-d1dik_3 (4_83_1_4)	1imba-d2hhma (5_8_1_2)	1lh7-d1lh1 (1_1_1_1)	1hvd-d1hvd (1_51_1_1)	1nal1-d1nal1 (3_1_3_1)
509	1npoa-d1npoa	2_7_1_1	81	1	0	0	0	0	0	0	1jeva-d2olba (3_73_1_1)	2tmda-d2tmda1 (3_1_7_1)	1gur-d1gur (7_3_4_1)	1bll-d1lam_2 (3_52_3_3)	1phe-d1phb (1_75_1_1)	1faga-d2bmha (1_75_1_1)	2bnh-d1dfji (3_7_1_1)	1rcf-d1rcf (3_13_4_1)	1gal-d1gal_1 (3_4_1_2)
510	1nsdb-d1nsca	2_45_1_1	390	4	1	1	0	0	0	0	3nn9-d3nn9 (2_45_1_1)	2sil-d2sil (2_45_1_1)	1eur-d1eur (2_45_1_1)	2bbkh-d2bbkh (2_46_2_1)	1mpmb-d1mal (6_7_1_2)	1ospo-d1ospo (2_52_1_1)	1fna-d1fna (2_1_2_1)	1lcl-d1lcl (2_19_1_3)	1dlc-d1dlc_2 (2_53_2_1)
511	1nsj-d1nsj	3_1_8_1	205	3	1	1	0	0	1	1	1pii-d1pii_2 (3_1_8_1)	1igs-d1igs (3_1_8_1)	2mnr-d2mnr_1 (3_1_6_2)	2chr-d2chr_1 (3_1_6_2)	1qapa-d1qapa1 (3_1_14_1)	1vid-d1vid (3_47_1_1)	1nal1-d1nal1 (3_1_3_1)	3chy-d3chy (3_13_2_1)	1php-d1php (3_66_1_1)
512	1nsp-d1npk	4_33_6_1	150	1	0	0	0	0	0	0	1mioa-d1mioa (3_67_1_1)	1pea-d1pea (3_72_1_1)	1pgn-d2pgd_2 (3_19_1_6)	1jud-d1jud (5_18_1_1)	1pyda-d1pvda3 (3_24_1_1)	1pgo-d2pgd_1 (1_71_1_1)	2ada-d1add (3_1_2_1)	1rcf-d1rcf (3_13_4_1)	1poxa-d1poxa2 (3_24_1_1)
513	1ntr-d1ntr	3_13_2_1	124	4	0	1	0	0	0	0	1mpd-d1mpb (3_73_1_1)	1miob-d1miob (3_67_1_1)	2aaa-d2aaa_2 (3_1_1_1)	3chy-d3chy (3_13_2_1)	1wsyb-d1wsyb (3_59_1_1)	1qora-d1qora2 (3_19_1_1)	1tkca-d1trka3 (3_34_1_1)	1scua-d1scua2 (3_19_1_8)	1dppc-d1dppa (3_73_1_1)
514	1oaca-d1oaca2	4_14_2_1	95	2	0	0	0	0	0	1	2tgi-d1tfg (7_14_1_2)	1apa-d1apa (4_94_1_1)	1udii-d1udii (4_14_4_1)	1mola-d1mola (4_14_1_1)	1rpa-d1rpa (3_43_1_2)	1pea-d1pea (3_72_1_1)	1dik-d1dik_3 (4_83_1_4)	1pex-d1pex (2_44_1_1)	1seia-d1seia (4_80_1_1)
515	1oaca-d1oaca4	4_42_1_1	86	1	0	0	0	0	0	0	2pola-d2pola1 (4_76_1_1)	2hmb-d1hms (2_41_1_2)	1eal-d1eal (2_41_1_2)	1cyx-d1cyx (2_5_1_2)	1imba-d2hhma (5_8_1_2)	1igd-d1igd (4_12_1_1)	1hcz-d1ctm_2 (2_59_2_2)	1pex-d1pex (2_44_1_1)	1tkaa-d1trka1 (3_24_1_2)
516	1oacb-d1oaca3	4_14_2_1	115	2	0	0	0	0	0	0	2trcb-d2trcb (2_46_3_1)	1pgs-d1pgs_1 (2_11_1_1)	1rsy-d1rsy (2_6_1_2)	1bgl-d1bgla2 (2_1_3_1)	4aahc-d4aaha (2_47_1_1)	1bmv1-d1bmv1 (2_8_1_2)	1tsra-d1tupa (2_2_3_1)	2anhb-d2anha (3_56_1_1)	1noy-d1noya (3_41_3_5)
517	1oav-d1iva	7_3_5_2	48	1	0	0	0	0	0	0	1febb-d2tpra2 (3_4_1_4)	1ose-d1ppi_1 (2_48_1_1)	1ciy-d1ciy_2 (2_53_2_1)	1noa-d1noa (2_1_6_1)	1gph1-d1gph12 (4_88_1_1)	1pls-d1pls (2_37_1_1)	1ad2-d1ad2 (5_20_1_1)	1poxa-d1poxa1 (3_21_1_1)	1gof-d1gof_3 (2_46_1_1)
518	1obpa-d1obpa	2_41_1_1	158	4	1	1	0	1	0	0	1bebb-d1beba (2_41_1_1)	1bbpa-d1bbpa (2_41_1_1)	1fem-d1hbp (2_41_1_1)	1ospo-d1ospo (2_52_1_1)	2hmb-d1hms (2_41_1_2)	1prn-d1prn (6_7_1_1)	1eal-d1eal (2_41_1_2)	2bbkh-d2bbkh (2_46_2_1)	1mpmb-d1mal (6_7_1_2)
519	1obr-d1obr	3_52_3_2	323	1	0	0	1	1	0	0	2ctb-d2ctb (3_52_3_1)	1qba-d1qba_3 (3_1_1_6)	1bll-d1lam_2 (3_52_3_3)	1bgl-d1bgla5 (3_1_1_3)	1gca-d1gca (3_72_1_1)	1art-d1art (3_48_1_1)	1asu-d1asu (3_41_3_2)	1lba-d1lba (4_64_1_1)	1wsyb-d1wsyb (3_59_1_1)
520	1occa-d1occa1	6_5_1_1	514	11	0	0	0	0	0	0	1mrrb-d1riba (1_24_1_2)	1mtyd-d1mmod (1_24_1_2)	1sly-d1sly_1 (1_84_4_1)	1fps-d1fps (1_91_1_1)	1mmob-d1mmob (1_24_1_2)	1csr-d1csh (1_74_1_1)	1occc-d1occc1 (6_5_1_1)	1xsm-d1xsm (1_24_1_2)	1pgo-d2pgd_1 (1_71_1_1)
521	1occb-d1occb1	2_5_1_2	137	2	1	1	0	1	0	0	1cyx-d1cyx (2_5_1_2)	1ure-d1ifc (2_41_1_2)	2pcy-d1plc (2_5_1_1)	1gen-d1gen (2_44_1_1)	2hmb-d1hms (2_41_1_2)	2omf-d2omf (6_7_1_1)	1xnb-d1xnb (2_19_1_8)	1cur-d1cur (2_5_1_1)	2bbkh-d2bbkh (2_46_2_1)
522	1occb-d1occb2	6_5_1_1	90	11	0	0	0	0	0	0	1lpe-d1lpe (1_23_1_1)	1buca-d1buca1 (1_23_6_1)	2liga-d2liga (1_23_2_1)	1bfra-d1bcfa (1_24_1_1)	1grj-d1grj_1 (1_2_1_1)	1mtyd-d1mmod (1_24_1_2)	1prcl-d1prcl1 (6_5_1_1)	1clc-d1clc_1 (1_73_1_2)	1occa-d1occa1 (6_5_1_1)
523	1occc-d1occc1	6_5_1_1	261	11	0	0	0	0	0	0	1mrrb-d1riba (1_24_1_2)	1afra-d1afra (1_24_1_2)	1xsm-d1xsm (1_24_1_2)	1mtyd-d1mmod (1_24_1_2)	1bfra-d1bcfa (1_24_1_1)	1occa-d1occa1 (6_5_1_1)	1fps-d1fps (1_91_1_1)	1ciy-d1ciy_3 (6_1_3_1)	1pgo-d2pgd_1 (1_71_1_1)
524	1occd-d1occd1	6_5_1_1	144	11	0	1	0	0	0	0	1occe-d1occe (1_84_7_1)	1sly-d1sly_1 (1_84_4_1)	1osa-d1osa (1_34_1_5)	1csr-d1csh (1_74_1_1)	1occa-d1occa1 (6_5_1_1)	1aep-d1aep (1_49_1_1)	1dlc-d1dlc_3 (6_1_3_1)	1lpe-d1lpe (1_23_1_1)	2sas-d2sas (1_34_1_5)

525	1occe-d1occe	1_84_7_1	109	1	0	0	0	0	0	1	1lpe-d1lpe (1_23_1_1)	1sly-d1sly_1 (1_84_4_1)	1cola-d1cola (6_1_1_1)	1occd-d1occd1 (6_5_1_1)	1lis-d1lis (1_17_1_1)	1bfra-d1bcfa (1_24_1_1)	1dlc-d1dlc_3 (6_1_3_1)	1oxa-d1oxa (1_75_1_1)	1hrda-d1hrda1 (3_19_1_7)
526	1occf-d1occf	7_33_1_1	98	1	0	0	0	0	0	0	2gpb-d1gpb (3_68_1_2)	1tcma-d1cdg_3 (2_48_1_1)	1mrj-d1mrj (4_94_1_1)	1qila-d1tssa1 (2_26_2_2)	1acf-d1acf (4_61_1_1)	2mipa-d1idaa (2_34_1_1)	1kfd-d1kfd_1 (3_41_3_4)	1eal-d1eal (2_41_1_2)	1dlha-d1dlha1 (2_1_1_2)
527	1occg-d1occg1	6_5_1_1	84	11	0	1	0	0	0	0	1cyda-d1cyda (3_19_1_2)	1oxa-d1oxa (1_75_1_1)	1faga-d2bmha (1_75_1_1)	1vsga-d1vsga (6_3_1_1)	1occc-d1occc1 (6_5_1_1)	1nai-d1xel (3_19_1_2)	1pea-d1pea (3_72_1_1)	2liga-d2liga (1_23_2_1)	1rcd-d1rcd (1_24_1_1)
528	1occi-d1occi1	6_5_1_1	73	11	0	0	0	0	0	0	1vsga-d1vsga (6_3_1_1)	1afra-d1afra (1_24_1_2)	1gtma-d1gtma1 (3_19_1_7)	1ryt-d1ryt_1 (1_24_1_1)	1zymb-d1zyma (3_5_1_2)	1occc-d1occc1 (6_5_1_1)	1pgo-d2pgd_1 (1_71_1_1)	1ezm-d1ezm_1 (1_53_1_1)	1bfra-d1bcfa (1_24_1_1)
529	1occj-d1occj1	6_5_1_1	56	11	0	0	0	0	0	0	1lpe-d1lpe (1_23_1_1)	1fps-d1fps (1_91_1_1)	1vnc-d1vnc (1_80_1_1)	1clc-d1clc_1 (1_73_1_2)	2gpb-d1gpb (3_68_1_2)	2masa-d2masa (3_51_1_1)	1glm-d1glm (1_73_1_1)	1dlc-d1dlc_3 (6_1_3_1)	2liga-d2liga (1_23_2_1)
530	1occk-d1occk1	6_5_1_1	49	11	0	0	0	0	0	0	1apme-d1apme (5_1_1_1)	1lyla-d1lyla2 (4_59_1_1)	1asza-d1asya2 (4_59_1_1)	1dik-d1dik_1 (3_1_9_2)	1cyda-d1cyda (3_19_1_2)	2ccya-d2ccya (1_23_3_2)	1rcd-d1rcd (1_24_1_1)	1rvea-d1rvaa (3_38_1_2)	1dik-d1dik_3 (4_83_1_4)
531	1occl-d1occl1	6_5_1_1	47	11	0	0	0	0	0	0	1rpa-d1rpa (3_43_1_2)	1mrj-d1mrj (4_94_1_1)	1taha-d1tahb (3_50_1_8)	1pgo-d2pgd_1 (1_71_1_1)	1lis-d1lis (1_17_1_1)	1osa-d1osa (1_34_1_5)	2liga-d2liga (1_23_2_1)	2gpb-d1gpb (3_68_1_2)	1sly-d1sly_1 (1_84_4_1)
532	1occu-d1occh	1_41_1_1	75	1	0	0	0	0	0	0	1mrrb-d1riba (1_24_1_2)	1nal1-d1nal1 (3_1_3_1)	1lki-d1lki (1_25_1_1)	1hvd-d1hvd (1_51_1_1)	1cola-d1cola (6_1_1_1)	2masa-d2masa (3_51_1_1)	1fipa-d1fipa (1_76_1_1)	1occc-d1occc1 (6_5_1_1)	1dik-d1dik_1 (3_1_9_2)
533	1octc-d1octc1	1_4_1_1	60	5	1	1	0	0	0	0	1hddc-d1enh (1_4_1_1)	1yrna-d1yrna (1_4_1_1)	1yrnb-d1yrnb (1_4_1_1)	1alo-d1alo_1 (1_47_1_1)	2tct-d2tct_1 (1_31_1_1)	1ezm-d1ezm_1 (1_53_1_1)	2wrpr-d2wrpr (1_78_1_1)	1prcl-d1prcl1 (6_5_1_1)	1gtma-d1gtma1 (3_19_1_7)
534	1octc-d1octc2	1_30_1_1	71	1	0	0	0	0	0	0	1osa-d1osa (1_34_1_5)	1sly-d1sly_2 (4_2_1_5)	1prcl-d1prcl1 (6_5_1_1)	1gtma-d1gtma1 (3_19_1_7)	1dlc-d1dlc_3 (6_1_3_1)	1wdcb-d1scmb (1_34_1_5)	1chka-d1chka (4_2_1_6)	1rnl-d1rnl_1 (1_31_1_2)	1jud-d1jud (5_18_1_1)
535	1oef-d1oef	8_35_1_1	24	1	0	0	0	0	0	0	1prcc-d1prcc (1_77_1_1)	1ngi-d1hpm_2 (3_41_1_1)	1fps-d1fps (1_91_1_1)	1crr-d5p21 (3_25_1_3)	1dlh-d1dlha2 (4_15_1_1)	7cat-d7cata (5_6_1_1)	4xis-d2xis (3_1_12_1)	1phk-d1phk (5_1_1_1)	1ltge-d1ltsd (2_26_2_1)
536	1oela-d1grl_2	3_5_3_1	176	1	0	0	0	0	0	0	1bmta-d1bmta2 (3_13_5_1)	1pgn-d2pgd_2 (3_19_1_6)	1mioa-d1mioa (3_67_1_1)	2tmda-d2tmda1 (3_1_7_1)	1orta-d1orta1 (3_58_1_1)	1dora-d1dora (3_1_7_1)	1psda-d1psda3 (4_33_15_1)	1wsyb-d1wsyb (3_59_1_1)	2dri-d2dri (3_72_1_1)
537	1oiba-d1pbp	3_73_1_1	321	8	1	1	0	0	0	0	1mpd-d1mpb (3_73_1_1)	1tlfa-d1tlfa (3_72_1_1)	1sfta-d1sfta2 (3_1_18_1)	1sbp-d1sbp (3_73_1_1)	1ggga-d1ggga (3_73_1_1)	1lst-d1lst (3_73_1_1)	1jeva-d2olba (3_73_1_1)	1htt-d1htta1 (3_37_1_1)	1tys-d1tys (4_63_1_1)
538	1onea-d1ebha1	3_1_6_1	295	1	0	0	1	1	1	1	2chr-d2chr_1 (3_1_6_2)	2mnr-d2mnr_1 (3_1_6_2)	4xis-d2xis (3_1_12_1)	2gpb-d1gpb (3_68_1_2)	1gca-d1gca (3_72_1_1)	2lbp-d2lbp (3_72_1_1)	2bnh-d1dfji (3_7_1_1)	1poxa-d1poxa2 (3_24_1_1)	1tlfa-d1tlfa (3_72_1_1)
539	1onea-d1ebha2	4_31_1_1	141	3	1	1	0	0	0	0	1chra-d2chr_2 (4_31_1_1)	2mnr-d2mnr_2 (4_31_1_1)	1hlb-d1hlb (1_1_1_1)	2myd-d1mbd (1_1_1_1)	1mpd-d1mpb (3_73_1_1)	1dkza-d1dkza (5_17_1_1)	2hmx-d2hmx (1_58_1_1)	1gtma-d1gtma1 (3_19_1_7)	1imba-d2hhma (5_8_1_2)
540	1opc-d1opc	1_4_3_5	99	1	0	0	0	0	1	1	1sfe-d1sfe_1 (1_4_2_1)	1regx-d1regx (4_33_19_1)	1rro-d1rro (1_34_1_4)	1osa-d1osa (1_34_1_5)	1pbwa-d1pbwa (1_83_1_1)	2abk-d2abk (1_66_1_1)	1vin-d1vin_1 (1_59_1_1)	1octc-d1octc2 (1_30_1_1)	1dik-d1dik_3 (4_83_1_4)
541	1opr-d1opr	3_44_1_1	213	3	1	1	0	0	0	0	1gph1-d1gph11 (3_44_1_1)	2ada-d1add (3_1_2_1)	1dora-d1dora (3_1_7_1)	2gpb-d1gpb (3_68_1_2)	1mioa-d1mioa (3_67_1_1)	1amp-d1amp (3_52_3_4)	1pea-d1pea (3_72_1_1)	1gtma-d1gtma1 (3_19_1_7)	1xyza-d1xyza (3_1_1_3)
542	1orb-d1orb_2	3_60_1_1	144	2	1	1	0	0	0	0	2ora-d1orb_1 (3_60_1_1)	1dora-d1dora (3_1_7_1)	1aco-d1aco_1 (3_5_2_1)	1rvva-d1rvva (3_9_1_1)	2bnh-d1dfji (3_7_1_1)	1hora-d1deaa (3_23_1_1)	3pgm-d3pgm (3_43_1_1)	3pmga-d3pmga1 (3_64_1_1)	1fld-d5nul (3_13_4_1)
543	1orda-d1orda1	3_13_6_1	107	1	0	0	0	0	0	1	1cyda-d1cyda (3_19_1_2)	3btoa-d2ohxa2 (3_19_1_1)	1gca-d1gca (3_72_1_1)	1ahia-d1fmca (3_19_1_2)	1rcf-d1rcf (3_13_4_1)	1dxy-d1dxy_1 (3_13_9_1)	1rnl-d1rnl_2 (3_13_2_1)	1nal1-d1nal1 (3_1_3_1)	1igs-d1igs (3_1_8_1)
544	1orda-d1orda2	3_48_1_4	462	1	0	0	1	1	0	0	1art-d1art (3_48_1_1)	2dkb-d2dkb (3_48_1_3)	1miob-d1miob (3_67_1_1)	2mnr-d2mnr_2 (4_31_1_1)	1fps-d1fps (1_91_1_1)	1aab-d1aab (1_20_1_1)	3pmga-d3pmga1 (3_64_1_1)	1zymb-d1zyma (3_5_1_2)	1lef-d1lefa (1_20_1_1)
545	1orda-d1orda3	4_70_1_1	161	1	0	0	0	0	0	0	1ad2-d1ad2 (5_20_1_1)	1phk-d1phk (5_1_1_1)	1phe-d1phb (1_75_1_1)	1oxa-d1oxa (1_75_1_1)	1bmfd-d1bmfd1 (1_55_1_1)	1faga-d2bmha (1_75_1_1)	1jud-d1jud (5_18_1_1)	1tfe-d1tfe (4_24_1_1)	1mxa-d1mxa_1 (4_75_1_1)
546	1orta-d1orta1	3_58_1_1	150	4	1	1	0	0	0	0	1raca-d1raaa1 (3_58_1_1)	2chr-d2chr_1 (3_1_6_2)	1psda-d1psda2 (3_19_1_4)	1mioa-d1mioa (3_67_1_1)	1dhpa-d1dhpa (3_1_3_1)	1nal1-d1nal1 (3_1_3_1)	1tlfa-d1tlfa (3_72_1_1)	1miob-d1miob (3_67_1_1)	1dora-d1dora (3_1_7_1)
547	1orta-d1orta2	3_58_1_1	185	4	1	1	0	0	0	0	1rada-d1raaa2 (3_58_1_1)	1qba-d1qba_3 (3_1_1_6)	1emd-d2cmd_1 (3_19_1_5)	1llda-d1llda1 (3_19_1_5)	1opr-d1opr (3_44_1_1)	1miob-d1miob (3_67_1_1)	2tmda-d2tmda2 (3_4_1_1)	2mnr-d2mnr_1 (3_1_6_2)	1geua-d1gesa2 (3_4_1_4)
548	1osa-d1osa	1_34_1_5	148	5	1	1	1	1	0	0	1tcob-d1tcob (1_34_1_5)	2sas-d2sas (1_34_1_5)	2scpa-d2scpa (1_34_1_5)	1wdcb-d1scmb (1_34_1_5)	1rro-d1rro (1_34_1_4)	1prcl-d1prcl1 (6_5_1_1)	1sra-d1sra (1_34_1_3)	1sly-d1sly_1 (1_84_4_1)	1ciy-d1ciy_3 (6_1_3_1)
549	1ose-d1ppi_1	2_48_1_1	92	6	0	0	0	0	0	0	1eur-d1eur (2_45_1_1)	2bbkh-d2bbkh (2_46_2_1)	1ospo-d1ospo (2_52_1_1)	1dar-d1dar_1 (2_29_3_1)	1sva5-d1sva1 (2_8_1_4)	2trcb-d2trcb (2_46_3_1)	1lcl-d1lcl (2_19_1_3)	1asqa-d1aoza2 (2_5_1_3)	2aaa-d2aaa_1 (2_48_1_1)
550	1ospo-d1ospo	2_52_1_1	251	1	0	0	0	0	0	0	1mpmb-d1mal (6_7_1_2)	2trcb-d2trcb (2_46_3_1)	2bbkh-d2bbkh (2_46_2_1)	1gof-d1gof_3 (2_46_1_1)	4aahc-d4aaha (2_47_1_1)	1ure-d1ifc (2_41_1_2)	1eal-d1eal (2_41_1_2)	1eur-d1eur (2_45_1_1)	1bgl-d1bgla1 (2_1_3_1)
551	1otfa-d1otfa	4_40_1_1	59	1	0	0	0	0	0	0	2chtb-d2chsa (4_39_1_1)	1orta-d1orta1 (3_58_1_1)	1lucb-d1lucb (3_1_13_1)	1gym-d1gym (3_1_15_2)	1bdmb-d1bdma2 (4_92_1_1)	1nfp-d1nfp (3_1_13_2)	1jlm-d1ido (3_45_1_1)	1llda-d1llda2 (4_92_1_1)	1rvva-d1rvva (3_9_1_1)
552	1otga-d1otga	4_40_1_2	125	1	0	0	0	0	0	0	1rpl-d1rpl (5_10_1_1)	1rvva-d1rvva (3_9_1_1)	1zooa-d1lfaa (3_45_1_1)	1qba-d1qba_3 (3_1_1_6)	1ris-d1ris (4_33_11_1)	1eri-d1eria (3_38_1_1)	1cyda-d1cyda (3_19_1_2)	1bmfc-d1bmfa3 (3_25_1_6)	1gym-d1gym (3_1_15_2)
553	1ouna-d1ouna	4_14_3_2	125	1	0	0	0	0	0	1	1mkaa-d1mkaa (4_21_1_1)	1eur-d1eur (2_45_1_1)	1mpmb-d1mal (6_7_1_2)	1mola-d1mola (4_14_1_1)	2hmb-d1hms (2_41_1_2)	1cdaa-d1aly (2_17_1_1)	1lcl-d1lcl (2_19_1_3)	2sil-d2sil (2_45_1_1)	1dih-d1dih_2 (4_41_1_2)
554	1oxa-d1oxa	1_75_1_1	403	3	1	1	0	0	0	0	1phe-d1phb (1_75_1_1)	1faga-d2bmha (1_75_1_1)	1occa-d1occa1 (6_5_1_1)	1mrrb-d1riba (1_24_1_2)	1hvd-d1hvd (1_51_1_1)	1sly-d1sly_1 (1_84_4_1)	1fps-d1fps (1_91_1_1)	1afra-d1afra (1_24_1_2)	1csr-d1csh (1_74_1_1)
555	1oya-d1oya	3_1_7_1	399	3	1	1	0	0	1	1	2tmda-d2tmda1 (3_1_7_1)	2ebn-d2ebn (3_1_1_5)	1nfp-d1nfp (3_1_13_2)	1lucb-d1lucb (3_1_13_1)	2ada-d1add (3_1_2_1)	1bya-d1byb (3_1_1_2)	1rcd-d1rcd (1_24_1_1)	1sbp-d1sbp (3_73_1_1)	1tpfa-d1tpfa (3_1_11_1)
556	1p03a-d2alp	2_31_1_1	198	3	1	1	0	1	0	0	5sgae-d1sgc (2_31_1_1)	1arc-d1arb (2_31_1_1)	1ospo-d1ospo (2_52_1_1)	1cpn-d1cpn (2_19_1_2)	1kxa-d2snv (2_31_1_3)	1hava-d1hava (2_31_1_4)	2trcb-d2trcb (2_46_3_1)	1nsdb-d1nsca (2_45_1_1)	3nn9-d3nn9 (2_45_1_1)
557	1paa-d1paa	7_28_1_1	30	10	1	1	0	0	0	0	1ard-d1ard (7_28_1_1)	1aay-d1zaac3 (7_28_1_1)	1aay-d1zaac2 (7_28_1_1)	1cpca-d1cpca (1_1_1_2)	1ecpa-d1ecpa (3_52_1_1)	1gln-d1gln_2 (3_15_1_1)	1znf-d1znf (7_28_1_1)	1edg-d1edg (3_1_1_3)	1cxh-d1cdg_4 (3_1_1_1)
558	1paw-d1pax_1	1_35_1_1	135	1	0	0	0	0	0	0	1buca-d1buca1 (1_23_6_1)	1occa-d1occa1 (6_5_1_1)	1flp-d1flp (1_1_1_1)	1prcl-d1prcl1 (6_5_1_1)	1lki-d1lki (1_25_1_1)	1fps-d1fps (1_91_1_1)	1ciy-d1ciy_3 (6_1_3_1)	1zymb-d1zyma (3_5_1_2)	2lhb-d2lhb (1_1_1_1)
559	1pax-d1pax_2	4_95_1_2	215	1	0	0	0	1	0	0	1bvp1-d1bvp12 (2_14_1_1)	1mpd-d1mpb (3_73_1_1)	1sgk-d1ddt_2 (4_95_1_1)	1rvva-d1rvva (3_9_1_1)	2sil-d2sil (2_45_1_1)	1iob-d1i1b (2_28_1_2)	1ahsa-d1ahsa (2_14_1_1)	1tsra-d1tupa (2_2_3_1)	1eri-d1eria (3_38_1_1)
560	1pba-d1pba	4_33_3_1	81	1	0	0	0	0	0	1	2dhd-d1ede (3_50_1_3)	1art-d1art (3_48_1_1)	1qapa-d1qapa1 (3_1_14_1)	1dhma-d1dhma (4_33_8_1)	1tib-d1tib (3_50_1_7)	1php-d1php (3_66_1_1)	2bnh-d1dfji (3_7_1_1)	2anhb-d2anha (3_56_1_1)	1nis-d1aco_2 (3_63_1_1)
561	1pbc-d1pbe_2	4_13_1_2	102	1	0	0	0	0	0	0	1prn-d1prn (6_7_1_1)	1jeva-d2olba (3_73_1_1)	1acf-d1acf (4_61_1_1)	1eal-d1eal (2_41_1_2)	1tpfa-d1tpfa (3_1_11_1)	1psda-d1psda3 (4_33_15_1)	1pkp-d1pkp_1 (4_11_1_1)	1mpmb-d1mal (6_7_1_2)	1ad2-d1ad2 (5_20_1_1)
562	1pbn-d1pbn	3_52_1_1	289	2	1	1	0	0	0	1	1ecpa-d1ecpa (3_52_1_1)	2hnp-d2hnp (3_32_1_2)	1gym-d1gym (3_1_15_2)	2ada-d1add (3_1_2_1)	1sfta-d1sfta2 (3_1_18_1)	1bll-d1lam_2 (3_52_3_3)	2mnr-d2mnr_1 (3_1_6_2)	2bnh-d1dfji (3_7_1_1)	1pea-d1pea (3_72_1_1)
563	1pbwa-d1pbwa	1_83_1_1	184	1	0	0	0	0	0	0	1cola-d1cola (6_1_1_1)	1csr-d1csh (1_74_1_1)	1ciy-d1ciy_3 (6_1_3_1)	1mtyg-d1mmog (1_22_1_1)	2hbg-d1hbg (1_1_1_1)	2sas-d2sas (1_34_1_5)	1phe-d1phb (1_75_1_1)	1itha-d1itha (1_1_1_1)	1tcob-d1tcob (1_34_1_5)
564	1pce-d1pce	7_12_1_1	60	5	1	1	0	0	0	0	1tbqr-d1tbrr1 (7_12_1_1)	1tbrr-d1tbrr2 (7_12_1_1)	1tgsi-d1tgsi (7_12_1_1)	1sgpi-d1sgpi (7_12_1_1)	1alo-d1alo_4 (4_77_1_1)	2anhb-d2anha (3_56_1_1)	1btn-d1btn (2_37_1_1)	1noy-d1noya (3_41_3_5)	1oiba-d1pbp (3_73_1_1)
565	1pcn-d1lpba1	7_3_8_1	39	2	0	0	0	0	0	0	1dar-d1dar_1 (2_29_3_1)	1gof-d1gof_3 (2_46_1_1)	1aocb-d1aoca (7_14_1_5)	2pia-d2pia_3 (4_12_4_1)	1ure-d1ifc (2_41_1_2)	7cat-d7cata (5_6_1_1)	1qba-d1qba_3 (3_1_1_6)	1phk-d1phk (5_1_1_1)	1apme-d1apme (5_1_1_1)
566	1pcn-d1lpba2	7_3_8_1	46	2	0	0	0	0	0	0	1hfi-d1hfi (7_15_1_1)	1ulo-d1ulo (2_13_1_4)	1dar-d1dar_3 (4_11_1_1)	2ms2a-d2ms2a (4_45_1_1)	1taha-d1tahb (3_50_1_8)	1hfh-d1hcc (7_15_1_1)	4aahd-d4aahb (8_46_1_1)	2act-d2act (4_3_1_1)	1dcta-d1dcta (3_47_1_4)
567	1pda-d1pda_2	4_28_2_1	88	1	0	0	0	0	0	0	7icd-d7icd (3_57_1_1)	1prtf-d1prtf (2_26_2_1)	2blta-d2blta (5_4_1_1)	1kob-d1koba (5_1_1_1)	1qba-d1qba_1 (2_1_1_5)	1alo-d1alo_6 (4_77_1_1)	3pte-d3pte (5_4_1_1)	1eal-d1eal (2_41_1_2)	6ldh-d1ldm_2 (4_92_1_1)
568	1pdn-d1pdnc	1_4_1_4	123	1	0	0	0	1	0	1	1rnl-d1rnl_1 (1_31_1_2)	1dpra-d1dpra1 (1_4_3_11)	4xis-d2xis (3_1_12_1)	1vola-d1vola1 (1_59_1_2)	1hddc-d1enh (1_4_1_1)	1bia-d1bia_1 (1_4_3_1)	1prcl-d1prcl1 (6_5_1_1)	1tpfa-d1tpfa (3_1_11_1)	1perl-d1r69 (1_30_1_2)
569	1pdo-d1pdo	3_40_1_1	129	1	0	0	0	0	0	0	2dri-d2dri (3_72_1_1)	1gca-d1gca (3_72_1_1)	1wsyb-d1wsyb (3_59_1_1)	1tlfa-d1tlfa (3_72_1_1)	1bmta-d1bmta2 (3_13_5_1)	1dhpa-d1dhpa (3_1_3_1)	1fbaa-d1fbaa (3_1_3_1)	3chy-d3chy (3_13_2_1)	1reqb-d1reqb2 (3_13_5_2)
570	1pea-d1pea	3_72_1_1	368	6	1	1	0	0	0	0	2lbp-d2lbp (3_72_1_1)	2dri-d2dri (3_72_1_1)	1gca-d1gca (3_72_1_1)	1tlfa-d1tlfa (3_72_1_1)	1pnr-d1pnra2 (3_72_1_1)	1miob-d1miob (3_67_1_1)	1dik-d1dik_1 (3_1_9_2)	1nal1-d1nal1 (3_1_3_1)	1bmta-d1bmta2 (3_13_5_1)
571	1peh-d1peh	8_13_1_1	33	1	0	0	0	0	0	0	1leha-d1leha1 (3_19_1_7)	1amp-d1amp (3_52_3_4)	1mmob-d1mmob (1_24_1_2)	1orta-d1orta2 (3_58_1_1)	1clc-d1clc_1 (1_73_1_2)	2gpb-d1gpb (3_68_1_2)	1occa-d1occa1 (6_5_1_1)	1mek-d1mek (3_33_1_2)	1gcn-d1gcn (8_8_1_1)
572	1perl-d1r69	1_30_1_2	63	5	1	1	0	0	0	0	1adr-d1adr (1_30_1_2)	1llib-d1llia (1_30_1_2)	1rnl-d1rnl_1 (1_31_1_2)	1pdn-d1pdnc (1_4_1_4)	1fipa-d1fipa (1_76_1_1)	1dpra-d1dpra1 (1_4_3_11)	1gln-d1gln_1 (1_67_1_1)	1mpd-d1mpb (3_73_1_1)	1seia-d1seia (4_80_1_1)

573	1pex-d1pex	2_44_1_1	192	2	1	1	0	0	0	0	1gen-d1gen (2_44_1_1)	2trcb-d2trcb (2_46_3_1)	2bbkh-d2bbkh (2_46_2_1)	4aahc-d4aaha (2_47_1_1)	2hmb-d1hms (2_41_1_2)	2sil-d2sil (2_45_1_1)	3nn9-d3nn9 (2_45_1_1)	1dlc-d1dlc_2 (2_53_2_1)	1ospo-d1ospo (2_52_1_1)
574	1pfsa-d1pfsa	2_26_4_7	78	3	0	1	0	0	0	0	1aocb-d1aoca (7_14_1_5)	1yhb-d1yhb (2_26_4_7)	2hmb-d1hms (2_41_1_2)	2sil-d2sil (2_45_1_1)	1ouna-d1ouna (4_14_3_2)	1eur-d1eur (2_45_1_1)	4aahc-d4aaha (2_47_1_1)	1lcl-d1lcl (2_19_1_3)	1fem-d1hbp (2_41_1_1)
575	1pft-d1pft	7_32_3_2	50	1	0	0	0	0	0	0	1btl-d1btl (5_4_1_1)	1aco-d1aco_1 (3_5_2_1)	2aky-d1aky (3_25_1_1)	2trcb-d2trcb (2_46_3_1)	1dlc-d1dlc_2 (2_53_2_1)	1gof-d1gof_3 (2_46_1_1)	2bbkh-d2bbkh (2_46_2_1)	1abrb-d1abrb1 (2_28_2_1)	1prch-d1prch1 (2_27_1_1)
576	1pgga-d1prha2	7_3_9_1	41	7	0	0	0	0	0	0	3pte-d3pte (5_4_1_1)	2sil-d2sil (2_45_1_1)	1pmi-d1pmi (2_58_2_1)	1gof-d1gof_3 (2_46_1_1)	1xnb-d1xnb (2_19_1_8)	1stu-d1stu (4_28_1_1)	1iob-d1i1b (2_28_1_2)	1idm-d1idm (3_57_1_1)	4kbpa-d4kbpa2 (4_91_1_1)
577	1pgn-d2pgd_2	3_19_1_6	176	1	0	0	1	1	0	0	1scua-d1scua2 (3_19_1_8)	1emd-d2cmd_1 (3_19_1_5)	1php-d1php (3_66_1_1)	2naca-d2naca2 (3_19_1_4)	1art-d1art (3_48_1_1)	1xva-d1xvaa (3_47_1_2)	1gdha-d1gdha2 (3_19_1_4)	1cyda-d1cyda (3_19_1_2)	1tlfa-d1tlfa (3_72_1_1)
578	1pgo-d2pgd_1	1_71_1_1	297	1	0	0	0	0	0	0	1sly-d1sly_1 (1_84_4_1)	1occc-d1occc1 (6_5_1_1)	1occa-d1occa1 (6_5_1_1)	1hvd-d1hvd (1_51_1_1)	1xsm-d1xsm (1_24_1_2)	1mrrb-d1riba (1_24_1_2)	1afra-d1afra (1_24_1_2)	1ciy-d1ciy_3 (6_1_3_1)	1csr-d1csh (1_74_1_1)
579	1pgs-d1pgs_1	2_11_1_1	137	2	0	0	0	0	0	0	2sil-d2sil (2_45_1_1)	1xnb-d1xnb (2_19_1_8)	1bbpa-d1bbpa (2_41_1_1)	2avia-d2avia (2_42_1_1)	1ttaa-d1eta1 (2_3_2_1)	1rsy-d1rsy (2_6_1_2)	1oacb-d1oaca3 (4_14_2_1)	1eur-d1eur (2_45_1_1)	1dlc-d1dlc_2 (2_53_2_1)
580	1pgs-d1pgs_2	2_11_1_1	174	2	0	0	0	0	0	0	1lcl-d1lcl (2_19_1_3)	1kcw-d1kcw_1 (2_5_1_3)	1ure-d1ifc (2_41_1_2)	1eur-d1eur (2_45_1_1)	2omf-d2omf (6_7_1_1)	1cpn-d1cpn (2_19_1_2)	2sil-d2sil (2_45_1_1)	1eft-d1eft_2 (2_30_1_1)	1inp-d1inp (5_8_1_3)
581	1phe-d1phb	1_75_1_1	405	3	1	1	0	0	0	0	1oxa-d1oxa (1_75_1_1)	1faga-d2bmha (1_75_1_1)	1prcl-d1prcl1 (6_5_1_1)	1afra-d1afra (1_24_1_2)	1occa-d1occa1 (6_5_1_1)	1fps-d1fps (1_91_1_1)	1mrrb-d1riba (1_24_1_2)	1mtyd-d1mmod (1_24_1_2)	1sly-d1sly_1 (1_84_4_1)
582	1phk-d1phk	5_1_1_1	277	4	1	1	0	0	0	0	1apme-d1apme (5_1_1_1)	1kob-d1koba (5_1_1_1)	2csn-d1csn (5_1_1_1)	2hnp-d2hnp (3_32_1_2)	1mxa-d1mxa_1 (4_75_1_1)	1fbaa-d1fbaa (3_1_3_1)	1rpl-d1rpl (5_10_1_1)	1lyla-d1lyla2 (4_59_1_1)	1prhb-d1prha1 (1_65_1_2)
583	1php-d1php	3_66_1_1	394	1	0	0	0	0	0	0	2dri-d2dri (3_72_1_1)	1tlfa-d1tlfa (3_72_1_1)	1nis-d1aco_2 (3_63_1_1)	1nal1-d1nal1 (3_1_3_1)	1pgn-d2pgd_2 (3_19_1_6)	4xis-d2xis (3_1_12_1)	2bnh-d1dfji (3_7_1_1)	1dora-d1dora (3_1_7_1)	2tmda-d2tmda1 (3_1_7_1)
584	1pht-d1pht	2_21_2_1	83	4	1	1	0	0	0	0	1aey-d1shg (2_21_2_1)	1sema-d1sema (2_21_2_1)	1regx-d1regx (4_33_19_1)	1hsq-d1hsq (2_21_2_1)	1dud-d1dupa (2_60_3_1)	1hpm-d1hpm_1 (3_41_1_1)	1fkg-d1fkd (4_19_1_1)	1cxsa-d1cxsa1 (2_35_2_1)	2pcy-d1plc (2_5_1_1)
585	1pii-d1pii_2	3_1_8_1	198	3	1	1	0	0	1	1	1nsj-d1nsj (3_1_8_1)	1nal1-d1nal1 (3_1_3_1)	3chy-d3chy (3_13_2_1)	1igs-d1igs (3_1_8_1)	1dhpa-d1dhpa (3_1_3_1)	2mnr-d2mnr_1 (3_1_6_2)	2chr-d2chr_1 (3_1_6_2)	1sfta-d1sfta2 (3_1_18_1)	1qapa-d1qapa1 (3_1_14_1)
586	1pil-d1pil	4_33_5_1	112	1	0	0	0	0	1	1	1ris-d1ris (4_33_11_1)	1asza-d1asya2 (4_59_1_1)	1msk-d1msk (4_99_1_1)	1dora-d1dora (3_1_7_1)	1scua-d1scua2 (3_19_1_8)	2ms2a-d2ms2a (4_45_1_1)	1rcf-d1rcf (3_13_4_1)	2lbp-d2lbp (3_72_1_1)	1gym-d1gym (3_1_15_2)
587	1pkp-d1pkp_1	4_11_1_1	71	2	0	0	0	0	0	0	1lucb-d1lucb (3_1_13_1)	1hrda-d1hrda1 (3_19_1_7)	1pyda-d1pvda3 (3_24_1_1)	2dri-d2dri (3_72_1_1)	1dora-d1dora (3_1_7_1)	1gdob-d1gdoa (4_88_1_1)	1aco-d1aco_1 (3_5_2_1)	1rnl-d1rnl_2 (3_13_2_1)	1nfp-d1nfp (3_1_13_2)
588	1pkp-d1pkp_2	4_28_1_2	74	1	0	0	0	0	0	0	2vik-d2vik (4_60_1_1)	1gtma-d1gtma2 (3_54_1_1)	1nsdb-d1nsca (2_45_1_1)	1alo-d1alo_6 (4_77_1_1)	1svb-d1svb_2 (6_10_1_1)	1dppc-d1dppa (3_73_1_1)	1otga-d1otga (4_40_1_2)	1btn-d1btn (2_37_1_1)	1fead-d2tpra3 (4_46_1_1)
589	1pky-d1pkya1	2_40_1_1	98	1	0	0	0	0	0	0	1p03a-d2alp (2_31_1_1)	1wit-d1wiu (2_1_1_4)	1sro-d1sro (2_26_4_4)	1dar-d1dar_1 (2_29_3_1)	1kit-d1kit_2 (2_19_1_6)	1asqa-d1aoza2 (2_5_1_3)	2sil-d2sil (2_45_1_1)	1wba-d1wba (2_28_3_1)	1hpca-d1htp (2_59_1_1)
590	1plp-d1plp	8_33_1_1	25	1	0	0	0	0	0	0	1juy-d1adea (3_25_1_5)	1tfb-d1vola2 (1_59_1_2)	2lhb-d2lhb (1_1_1_1)	2hmx-d2hmx (1_58_1_1)	1mrj-d1mrj (4_94_1_1)	1dpra-d1dpra2 (1_61_1_1)	1prcl-d1prcl1 (6_5_1_1)	1hrda-d1hrda2 (3_54_1_1)	1hme-d1hme (1_20_1_1)
591	1plq-d1plq_1	4_76_1_2	126	2	0	1	1	1	0	0	2pola-d2pola3 (4_76_1_1)	1plr-d1plq_2 (4_76_1_2)	1ospo-d1ospo (2_52_1_1)	1ttaa-d1eta1 (2_3_2_1)	2hmb-d1hms (2_41_1_2)	2pola-d2pola1 (4_76_1_1)	1gof-d1gof_2 (2_13_1_1)	1eal-d1eal (2_41_1_2)	1fem-d1hbp (2_41_1_1)
592	1plr-d1plq_2	4_76_1_2	132	2	0	1	1	1	0	0	2pola-d2pola3 (4_76_1_1)	1plq-d1plq_1 (4_76_1_2)	2pola-d2pola2 (4_76_1_1)	2hmb-d1hms (2_41_1_2)	1dcha-d1dcoa (4_38_1_1)	2por-d2por (6_7_1_1)	1iov-d2dln_2 (4_83_1_1)	1eal-d1eal (2_41_1_2)	1chma-d1chma1 (3_41_2_1)
593	1pls-d1pls	2_37_1_1	113	5	1	1	0	1	0	0	1btn-d1btn (2_37_1_1)	1pms-d1pms (2_37_1_1)	1hava-d1hava (2_31_1_4)	1ytfa-d1ytba1 (4_74_1_1)	1irsa-d1irsa (2_37_1_2)	1dyna-d1dyna (2_37_1_1)	1fem-d1hbp (2_41_1_1)	2phl-d2phla1 (2_58_1_1)	1cyx-d1cyx (2_5_1_2)
594	1pmc-d1pmc	7_4_1_1	36	1	0	0	0	0	0	0	1dppc-d1dppa (3_73_1_1)	1mai-d1mai (2_37_1_1)	1hcgb-d1hcgb (7_3_9_1)	2trcb-d2trcb (2_46_3_1)	1qba-d1qba_1 (2_1_1_5)	2aaa-d2aaa_1 (2_48_1_1)	1eac-d1eaf (3_30_1_1)	1dlc-d1dlc_2 (2_53_2_1)	1tdta-d1tdta (2_57_1_2)
595	1pmi-d1pmi	2_58_2_1	440	1	0	0	0	0	0	0	1ciy-d1ciy_1 (2_13_1_2)	1occc-d1occc1 (6_5_1_1)	1dkza-d1dkza (5_17_1_1)	1pgo-d2pgd_1 (1_71_1_1)	1buca-d1buca1 (1_23_6_1)	1occa-d1occa1 (6_5_1_1)	1tfe-d1tfe (4_24_1_1)	1oxa-d1oxa (1_75_1_1)	1alla-d1alla (1_1_1_2)
596	1pms-d1pms	2_37_1_1	135	5	1	1	1	1	0	0	1pls-d1pls (2_37_1_1)	1dyna-d1dyna (2_37_1_1)	1shca-d1shca (2_37_1_2)	1vjs-d1bplb1 (2_48_1_1)	1hgea-d1hgea (2_14_1_2)	2hmb-d1hms (2_41_1_2)	1nsdb-d1nsca (2_45_1_1)	1mai-d1mai (2_37_1_1)	1tnn-d1tnm (2_1_1_4)
597	1pne-d1pne	4_61_1_1	138	2	1	1	0	0	0	0	1acf-d1acf (4_61_1_1)	1chma-d1chma2 (4_72_1_1)	1lxta-d3pmga4 (4_74_2_1)	1gym-d1gym (3_1_15_2)	1dkza-d1dkza (5_17_1_1)	1mbb-d1mbb_1 (4_84_1_2)	3nn9-d3nn9 (2_45_1_1)	2dkb-d2dkb (3_48_1_3)	1hrda-d1hrda2 (3_54_1_1)
598	1pnh-d1pnh	7_3_6_2	31	4	0	0	0	0	0	0	1gen-d1gen (2_44_1_1)	1ptf-d1ptf (4_52_1_1)	1pex-d1pex (2_44_1_1)	1lyla-d1lyla2 (4_59_1_1)	1bove-d1bova (2_26_2_1)	2por-d2por (6_7_1_1)	1gym-d1gym (3_1_15_2)	1yua-d1yua_2 (4_67_1_2)	1dppc-d1dppa (3_73_1_1)
599	1pnr-d1pnra2	3_72_1_1	282	6	1	1	0	0	0	0	1tlfa-d1tlfa (3_72_1_1)	2dri-d2dri (3_72_1_1)	1gca-d1gca (3_72_1_1)	2lbp-d2lbp (3_72_1_1)	1pea-d1pea (3_72_1_1)	1miob-d1miob (3_67_1_1)	1dnpa-d1dnpa2 (3_17_1_1)	2chr-d2chr_1 (3_1_6_2)	1cowe-d1bmfd3 (3_25_1_6)
600	1pnt-d1phr	3_31_1_1	154	1	0	0	0	0	0	0	1pnr-d1pnra2 (3_72_1_1)	1ahia-d1fmca (3_19_1_2)	1tlfa-d1tlfa (3_72_1_1)	1gca-d1gca (3_72_1_1)	1cyda-d1cyda (3_19_1_2)	1rvva-d1rvva (3_9_1_1)	1tml-d1tml (3_2_1_1)	1bya-d1byb (3_1_1_2)	6ldh-d1ldm_1 (3_19_1_5)
601	1poba-d1poa	1_95_1_2	118	1	0	0	0	0	0	0	1regx-d1regx (4_33_19_1)	1mbt-d1mbb_2 (4_85_1_1)	1esd-d1esc (3_13_8_1)	1xyza-d1xyza (3_1_1_3)	1hpm-d1hpm_1 (3_41_1_1)	1mrrb-d1riba (1_24_1_2)	1bdmb-d1bdma2 (4_92_1_1)	1dik-d1dik_3 (4_83_1_4)	1hrda-d1hrda2 (3_54_1_1)
602	1poc-d1poc	1_95_1_1	134	1	0	0	0	0	0	0	1lyla-d1lyla2 (4_59_1_1)	1pgo-d2pgd_1 (1_71_1_1)	1dppc-d1dppa (3_73_1_1)	1lucb-d1lucb (3_1_13_1)	2bnh-d1dfji (3_7_1_1)	1htda-d1atla (4_50_1_5)	1xsm-d1xsm (1_24_1_2)	1mrj-d1mrj (4_94_1_1)	1lis-d1lis (1_17_1_1)
603	1poh-d1poh	4_52_1_1	85	2	1	1	0	0	0	0	1ptf-d1ptf (4_52_1_1)	1scub-d1scub1 (3_13_3_1)	2blta-d2blta (5_4_1_1)	1vid-d1vid (3_47_1_1)	1fead-d2tpra3 (4_46_1_1)	7icd-d7icd (3_57_1_1)	3pte-d3pte (5_4_1_1)	1nai-d1xel (3_19_1_2)	2bnh-d1dfji (3_7_1_1)
604	1poxa-d1poxa1	3_21_1_1	183	1	0	0	0	0	0	0	1oya-d1oya (3_1_7_1)	1art-d1art (3_48_1_1)	1gca-d1gca (3_72_1_1)	1pea-d1pea (3_72_1_1)	1gtma-d1gtma1 (3_19_1_7)	1edt-d1edt (3_1_1_5)	1orta-d1orta2 (3_58_1_1)	1tlfa-d1tlfa (3_72_1_1)	1vhra-d1vhra (3_32_1_1)
605	1poxa-d1poxa2	3_24_1_1	174	3	0	0	0	0	0	0	1tlfa-d1tlfa (3_72_1_1)	1miob-d1miob (3_67_1_1)	1dik-d1dik_3 (4_83_1_4)	1onea-d1ebha1 (3_1_6_1)	1nal1-d1nal1 (3_1_3_1)	1pnr-d1pnra2 (3_72_1_1)	1cowe-d1bmfd3 (3_25_1_6)	1mioa-d1mioa (3_67_1_1)	1art-d1art (3_48_1_1)
606	1poxa-d1poxa3	3_24_1_1	188	3	1	1	1	1	0	0	1pyda-d1pvda3 (3_24_1_1)	1poxa-d1poxa2 (3_24_1_1)	1tkaa-d1trka1 (3_24_1_2)	2dri-d2dri (3_72_1_1)	1nal1-d1nal1 (3_1_3_1)	1dhpa-d1dhpa (3_1_3_1)	1tkca-d1trka2 (3_24_1_2)	1wsyb-d1wsyb (3_59_1_1)	1gky-d1gky (3_25_1_1)
607	1poy1-d1pot	3_73_1_1	322	8	1	1	0	0	0	0	1mpd-d1mpb (3_73_1_1)	1sbp-d1sbp (3_73_1_1)	1gca-d1gca (3_72_1_1)	1cxh-d1cdg_4 (3_1_1_1)	2dri-d2dri (3_72_1_1)	1php-d1php (3_66_1_1)	1pxt-d1pxta2 (3_74_1_1)	1jeva-d2olba (3_73_1_1)	1ahia-d1fmca (3_19_1_2)
608	1ppi-d1ppi_2	3_1_1_1	403	4	1	1	1	1	0	1	1cxh-d1cdg_4 (3_1_1_1)	2aaa-d2aaa_2 (3_1_1_1)	1amy-d1amy_2 (3_1_1_1)	1qba-d1qba_3 (3_1_1_6)	1miob-d1miob (3_67_1_1)	1eceb-d1ecea (3_1_1_3)	1lam-d1lam_1 (3_36_1_1)	1bll-d1lam_2 (3_52_3_3)	1alo-d1alo_6 (4_77_1_1)
609	1ppt-d1ppt	8_5_1_1	36	2	0	0	0	0	0	0	1lucb-d1lucb (3_1_13_1)	1nfp-d1nfp (3_1_13_2)	1acf-d1acf (4_61_1_1)	2dkb-d2dkb (3_48_1_3)	1orta-d1orta1 (3_58_1_1)	1fbaa-d1fbaa (3_1_3_1)	1lghb-d1lghb (6_6_1_1)	1ad2-d1ad2 (5_20_1_1)	1otga-d1otga (4_40_1_2)
610	1prcc-d1prcc	1_77_1_1	332	1	0	0	0	0	0	0	1xyza-d1xyza (3_1_1_3)	1onea-d1ebha1 (3_1_6_1)	1lh7-d1lh1 (1_1_1_1)	1jkw-d1jkw_2 (1_59_1_1)	1lfb-d1lfb (1_4_1_1)	1prch-d1prch2 (6_5_1_1)	1cxh-d1cdg_4 (3_1_1_1)	1cb2b-d1cb2a (3_2_1_1)	1hvd-d1hvd (1_51_1_1)
611	1prch-d1prch1	2_27_1_1	222	1	0	0	0	0	0	0	1apa-d1apa (4_94_1_1)	1rsy-d1rsy (2_6_1_2)	2pcd-d2pcda (2_3_3_1)	1p03a-d2alp (2_31_1_1)	1mrj-d1mrj (4_94_1_1)	2sil-d2sil (2_45_1_1)	2bbkh-d2bbkh (2_46_2_1)	1ospo-d1ospo (2_52_1_1)	1kxa-d2snv (2_31_1_3)
612	1prch-d1prch2	6_5_1_1	35	11	0	1	0	0	0	0	1prcc-d1prcc (1_77_1_1)	1occa-d1occa1 (6_5_1_1)	1prhb-d1prha1 (1_65_1_2)	1xsm-d1xsm (1_24_1_2)	4xis-d2xis (3_1_12_1)	2sas-d2sas (1_34_1_5)	1lyla-d1lyla2 (4_59_1_1)	1grj-d1grj_1 (1_2_1_1)	1cowe-d1bmfd3 (3_25_1_6)
613	1prcl-d1prcl1	6_5_1_1	273	11	1	1	0	0	0	0	1occa-d1occa1 (6_5_1_1)	1faga-d2bmha (1_75_1_1)	1xsm-d1xsm (1_24_1_2)	1occc-d1occc1 (6_5_1_1)	1mrrb-d1riba (1_24_1_2)	1csr-d1csh (1_74_1_1)	1oxa-d1oxa (1_75_1_1)	1pgo-d2pgd_1 (1_71_1_1)	1rpa-d1rpa (3_43_1_2)
614	1preb-d1prea1	4_97_1_2	83	2	1	1	0	0	0	0	1ptoh-d1prtb2 (4_97_1_2)	1apa-d1apa (4_94_1_1)	1pxt-d1pxta2 (3_74_1_1)	1pky-d1pkya1 (2_40_1_1)	1fbaa-d1fbaa (3_1_3_1)	1dhpa-d1dhpa (3_1_3_1)	2aaa-d2aaa_1 (2_48_1_1)	1igna-d1igna1 (1_4_1_5)	1xra-d1mxa_2 (4_75_1_1)
615	1prhb-d1prha1	1_65_1_2	513	1	0	0	0	0	0	0	1lki-d1lki (1_25_1_1)	1occc-d1occc1 (6_5_1_1)	1mtyd-d1mmod (1_24_1_2)	1gsea-d1gsea1 (1_38_1_1)	1ciy-d1ciy_3 (6_1_3_1)	1aep-d1aep (1_49_1_1)	1scb-d1csee (3_28_1_1)	1pgo-d2pgd_1 (1_71_1_1)	1prcl-d1prcl1 (6_5_1_1)
616	1prn-d1prn	6_7_1_1	289	3	1	1	1	1	0	0	2por-d2por (6_7_1_1)	2omf-d2omf (6_7_1_1)	1mpmb-d1mal (6_7_1_2)	1ospo-d1ospo (2_52_1_1)	2hmb-d1hms (2_41_1_2)	1obpa-d1obpa (2_41_1_1)	1qatb-d1djxa2 (2_6_1_1)	1eal-d1eal (2_41_1_2)	2sil-d2sil (2_45_1_1)
617	1prr-d1prr_1	2_9_1_2	90	2	0	1	1	1	0	0	1blba-d2bb2_2 (2_9_1_1)	1prs-d1prr_2 (2_9_1_2)	1gcs-d4gcr_1 (2_9_1_1)	4gcr-d4gcr_2 (2_9_1_1)	1irsa-d1irsa (2_37_1_2)	1kcw-d1kcw_2 (2_5_1_3)	1gtqa-d1gtqa (4_54_1_2)	2bnh-d1dfji (3_7_1_1)	1kcw-d1kcw_4 (2_5_1_3)
618	1prs-d1prr_2	2_9_1_2	83	2	1	1	1	1	0	0	1prr-d1prr_1 (2_9_1_2)	4gcr-d4gcr_2 (2_9_1_1)	1gcs-d4gcr_1 (2_9_1_1)	1blba-d2bb2_2 (2_9_1_1)	1whi-d1whi (2_25_1_1)	1kcw-d1kcw_6 (2_5_1_3)	1kcw-d1kcw_2 (2_5_1_3)	1asya-d1asya1 (2_26_4_1)	2trcb-d2trcb (2_46_3_1)
619	1prtd-d1prtd	2_26_2_1	110	6	1	1	0	0	0	1	1prtf-d1prtf (2_26_2_1)	1lyla-d1lyla2 (4_59_1_1)	1bcpb-d1prtb1 (2_26_2_1)	1fbaa-d1fbaa (3_1_3_1)	1sva5-d1sva1 (2_8_1_4)	1asya-d1asya1 (2_26_4_1)	1hmp-d1hmpa (3_44_1_1)	1ytn-d1ypta (3_32_1_2)	1daaa-d1daaa (5_14_1_1)
620	1prtf-d1prtf	2_26_2_1	98	6	0	1	0	0	0	0	1coy-d1coy_1 (3_4_1_2)	1pda-d1pda_2 (4_28_2_1)	1ltge-d1ltsd (2_26_2_1)	1prtd-d1prtd (2_26_2_1)	1broa-d1broa (3_50_1_5)	1tiig-d1tiid (2_26_2_1)	1chma-d1chma2 (4_72_1_1)	1iov-d2dln_2 (4_83_1_1)	1gtma-d1gtma2 (3_54_1_1)

621	1psda-d1psda2	3_19_1_4	188	4	1	1	1	1	0	0	1gdha-d1gdha2 (3_19_1_4)	1dxy-d1dxy_2 (3_19_1_4)	2naca-d2naca2 (3_19_1_4)	1gtma-d1gtma1 (3_19_1_7)	1bncb-d1bnca2 (3_20_1_1)	2dri-d2dri (3_72_1_1)	1orta-d1orta1 (3_58_1_1)	1raca-d1raaa1 (3_58_1_1)	1scua-d1scua2 (3_19_1_8)
622	1psda-d1psda3	4_33_15_1	84	1	0	0	0	0	1	1	1ris-d1ris (4_33_11_1)	1pea-d1pea (3_72_1_1)	1xxca-d1xxaa (4_38_2_1)	1scub-d1scub1 (3_13_3_1)	1scua-d1scua1 (3_13_3_1)	1oela-d1grl_2 (3_5_3_1)	1tlfa-d1tlfa (3_72_1_1)	1mioa-d1mioa (3_67_1_1)	1hrda-d1hrda1 (3_19_1_7)
623	1psf-d1pse	2_21_5_1	69	1	0	0	0	0	0	0	1eur-d1eur (2_45_1_1)	1pms-d1pms (2_37_1_1)	1akl-d1kapp1 (2_55_1_1)	2bbkh-d2bbkh (2_46_2_1)	1tys-d1tys (4_63_1_1)	1csei-d1csei (4_22_1_1)	1taha-d1tahb (3_50_1_8)	1dkza-d1dkza (5_17_1_1)	1cdaa-d1aly (2_17_1_1)
624	1psm-d1psm	8_17_1_1	38	1	0	0	0	0	0	0	1dora-d1dora (3_1_7_1)	1hme-d1hme (1_20_1_1)	1llib-d1llia (1_30_1_2)	1pbwa-d1pbwa (1_83_1_1)	1fps-d1fps (1_91_1_1)	1lucb-d1lucb (3_1_13_1)	1dhpa-d1dhpa (3_1_3_1)	1lpe-d1lpe (1_23_1_1)	2abk-d2abk (1_66_1_1)
625	1ptf-d1ptf	4_52_1_1	87	2	1	1	0	0	0	0	1poh-d1poh (4_52_1_1)	2blta-d2blta (5_4_1_1)	1scub-d1scub1 (3_13_3_1)	7icd-d7icd (3_57_1_1)	3pte-d3pte (5_4_1_1)	1dhpa-d1dhpa (3_1_3_1)	1bmta-d1bmta2 (3_13_5_1)	1regx-d1regx (4_33_19_1)	1tml-d1tml (3_2_1_1)
626	1ptoh-d1prtb2	4_97_1_2	84	2	1	1	0	0	0	0	1preb-d1prea1 (4_97_1_2)	1iov-d2dln_2 (4_83_1_1)	7icd-d7icd (3_57_1_1)	1orda-d1orda3 (4_70_1_1)	1ytfa-d1ytba1 (4_74_1_1)	1gph1-d1gph12 (4_88_1_1)	2sil-d2sil (2_45_1_1)	2pola-d2pola1 (4_76_1_1)	1nis-d1aco_2 (3_63_1_1)
627	1ptq-d1ptq	7_38_1_1	50	2	1	1	0	0	0	0	1faq-d1faq (7_38_1_1)	1kxa-d2snv (2_31_1_3)	1wgcb-d1wgta4 (7_3_1_1)	1gen-d1gen (2_44_1_1)	1gat-d1gata (7_30_1_1)	1rie-d1rie (7_33_1_2)	2bbkh-d2bbkh (2_46_2_1)	1rahb-d1raab2 (7_32_5_1)	1ppi-d1ppi_2 (3_1_1_1)
628	1puee-d1puee	1_4_3_9	88	2	0	0	0	0	0	0	1jud-d1jud (5_18_1_1)	2ada-d1add (3_1_2_1)	1mmob-d1mmob (1_24_1_2)	1kraa-d2kaua (4_6_1_1)	1ggt-d1ggta4 (4_3_1_2)	1csr-d1csh (1_74_1_1)	2lbp-d2lbp (3_72_1_1)	1prhb-d1prha1 (1_65_1_2)	1lea-d1lea (1_4_3_2)
629	1put-d1put	4_12_4_1	106	3	0	1	0	0	1	1	1guab-d1guab (4_12_3_1)	1aara-d1ubi (4_12_2_1)	1ltge-d1ltsd (2_26_2_1)	2pia-d2pia_3 (4_12_4_1)	1aco-d1aco_1 (3_5_2_1)	1svb-d1svb_2 (6_10_1_1)	1inp-d1inp (5_8_1_3)	1frd-d1frd (4_12_4_1)	1fem-d1hbp (2_41_1_1)
630	1pxt-d1pxta2	3_74_1_1	124	1	0	0	0	0	0	0	1ngi-d1hpm_2 (3_41_1_1)	1rvva-d1rvva (3_9_1_1)	1nox-d1nox (4_49_1_1)	1poy1-d1pot (3_73_1_1)	1bmta-d1bmta2 (3_13_5_1)	2dri-d2dri (3_72_1_1)	1dora-d1dora (3_1_7_1)	1fbaa-d1fbaa (3_1_3_1)	1mrj-d1mrj (4_94_1_1)
631	1pyc-d1pyc	7_29_1_1	41	3	1	1	0	0	0	0	1d66a-d1d66a2 (7_29_1_1)	1pyib-d1pyia2 (7_29_1_1)	1tpfa-d1tpfa (3_1_11_1)	1oela-d1grl_2 (3_5_3_1)	1iow-d2dln_1 (3_20_1_2)	1seta-d1seta2 (4_59_1_1)	1qapa-d1qapa1 (3_1_14_1)	1php-d1php (3_66_1_1)	1nox-d1nox (4_49_1_1)
632	1pyda-d1pyda3	3_24_1_1	196	3	1	1	1	1	0	0	1poxa-d1poxa3 (3_24_1_1)	1tkca-d1trka2 (3_24_1_2)	1wsyb-d1wsyb (3_59_1_1)	2chr-d2chr_1 (3_1_6_2)	1vid-d1vid (3_47_1_1)	1fcdb-d1fcda2 (3_4_1_4)	2ctb-d2ctb (3_52_3_1)	2dkb-d2dkb (3_48_1_3)	1pgn-d2pgd_2 (3_19_1_6)
633	1pyia-d1pyia1	1_97_2_1	46	4	0	0	0	0	0	0	1cbg-d1cbg (3_1_1_4)	1bfra-d1bcfa (1_24_1_1)	1pnt-d1phr (3_31_1_1)	2tmda-d2tmda1 (3_1_7_1)	1lyla-d1lyla2 (4_59_1_1)	1fps-d1fps (1_91_1_1)	1orda-d1orda2 (3_48_1_4)	1gdz-d1ak4c (1_57_1_1)	1qba-d1qba_3 (3_1_1_6)
634	1pyib-d1pyia2	7_29_1_1	42	3	1	1	0	0	0	0	1d66a-d1d66a2 (7_29_1_1)	1pyc-d1pyc (7_29_1_1)	1htt-d1htta1 (3_37_1_1)	1lyla-d1lyla2 (4_59_1_1)	1dik-d1dik_2 (3_5_1_1)	2bbkh-d2bbkh (2_46_2_1)	1scub-d1scub2 (4_83_1_3)	1pii-d1pii_2 (3_1_8_1)	1prtd-d1prtd (2_26_2_1)
635	1qapa-d1qapa1	3_1_14_1	167	1	0	0	0	0	1	1	1igs-d1igs (3_1_8_1)	1nsj-d1nsj (3_1_8_1)	1wsyb-d1wsyb (3_59_1_1)	1qora-d1qora2 (3_19_1_1)	1srrc-d1srra (3_13_2_1)	1nal1-d1nal1 (3_1_3_1)	1miob-d1miob (3_67_1_1)	2dri-d2dri (3_72_1_1)	1pii-d1pii_2 (3_1_8_1)
636	1qapa-d1qapa2	4_23_2_1	122	1	0	0	0	0	0	0	2dkb-d2dkb (3_48_1_3)	1pgo-d2pgd_1 (1_71_1_1)	1ouna-d1ouna (4_14_3_2)	1gpr-d1gpr (2_59_3_1)	1wsyb-d1wsyb (3_59_1_1)	1gcb-d1gcb (4_3_1_1)	1apme-d1apme (5_1_1_1)	1art-d1art (3_48_1_1)	1mbt-d1mbb_2 (4_85_1_1)
637	1qatb-d1djxa2	2_6_1_1	131	1	0	0	1	1	0	0	1rsy-d1rsy (2_6_1_2)	1prn-d1prn (6_7_1_1)	1ospo-d1ospo (2_52_1_1)	1tsra-d1tupa (2_2_3_1)	2pcd-d2pcda (2_3_3_1)	1tcma-d1cdg_3 (2_48_1_1)	2omf-d2omf (6_7_1_1)	1noa-d1noa (2_1_6_1)	1eur-d1eur (2_45_1_1)
638	1qba-d1qba_1	2_1_1_5	105	6	0	0	0	0	0	0	1pda-d1pda_2 (4_28_2_1)	3pte-d3pte (5_4_1_1)	2sil-d2sil (2_45_1_1)	1ospo-d1ospo (2_52_1_1)	1qila-d1tssa1 (2_26_2_2)	1obpa-d1obpa (2_41_1_1)	1rcd-d1rcd (1_24_1_1)	1rvea-d1rvaa (3_38_1_2)	1lvl-d1lvl_2 (3_4_1_4)
639	1qba-d1qba_3	3_1_1_6	443	1	0	0	1	1	1	1	1edg-d1edg (3_1_1_3)	1eceb-d1ecea (3_1_1_3)	1tpfa-d1tpfa (3_1_11_1)	2lbp-d2lbp (3_72_1_1)	1tlfa-d1tlfa (3_72_1_1)	1ahia-d1fmca (3_19_1_2)	1xyza-d1xyza (3_1_1_3)	1cxh-d1cdg_4 (3_1_1_1)	4xis-d2xis (3_1_12_1)
640	1qbb-d1qba_2	2_2_2_3	173	1	0	0	0	0	0	0	1ciy-d1ciy_2 (2_53_2_1)	1sacb-d1saca (2_19_1_4)	1lcl-d1lcl (2_19_1_3)	1hpl-d1hpla1 (2_10_2_1)	1ggt-d1ggta2 (2_1_4_1)	2ncm-d2ncm (2_1_1_4)	1bgl-d1bgla1 (2_1_3_1)	2hmb-d1hms (2_41_1_2)	2trcb-d2trcb (2_46_3_1)
641	1qbb-d1qba_4	4_50_2_1	137	1	0	0	0	0	0	0	2tmda-d2tmda1 (3_1_7_1)	1eac-d1eaf (3_30_1_1)	1pda-d1pda_2 (4_28_2_1)	1gtma-d1gtma2 (3_54_1_1)	3rubs-d3rubs (4_37_1_1)	1asu-d1asu (3_41_3_2)	1nal1-d1nal1 (3_1_3_1)	1idm-d1idm (3_57_1_1)	1stu-d1stu (4_28_1_1)
642	1qila-d1tssa1	2_26_2_2	93	1	0	0	0	0	0	1	2aaa-d2aaa_1 (2_48_1_1)	1lylb-d1lyla1 (2_26_4_1)	2tmda-d2tmda2 (3_4_1_1)	1ospo-d1ospo (2_52_1_1)	3pte-d3pte (5_4_1_1)	1gof-d1gof_3 (2_46_1_1)	1qba-d1qba_1 (2_1_1_5)	1msad-d1msaa (2_54_1_1)	1gpr-d1gpr (2_59_3_1)
643	1qora-d1qora2	3_19_1_1	130	2	1	1	0	1	0	0	3btoa-d2ohxa2 (3_19_1_1)	1sfta-d1sfta2 (3_1_18_1)	1qapa-d1qapa1 (3_1_14_1)	1rnl-d1rnl_2 (3_13_2_1)	1lst-d1lst (3_73_1_1)	1ahia-d1fmca (3_19_1_2)	1cyda-d1cyda (3_19_1_2)	1art-d1art (3_48_1_1)	1nai-d1xel (3_19_1_2)
644	1qrda-d1qrda	3_13_4_2	273	1	0	0	0	0	0	0	1xyza-d1xyza (3_1_1_3)	1tlfa-d1tlfa (3_72_1_1)	1dik-d1dik_1 (3_1_9_2)	1ahia-d1fmca (3_19_1_2)	2tmda-d2tmda1 (3_1_7_1)	1tkca-d1trka3 (3_34_1_1)	1hdca-d1hdca (3_19_1_2)	4xis-d2xis (3_1_12_1)	1cyda-d1cyda (3_19_1_2)
645	1raca-d1raaa1	3_58_1_1	150	4	1	1	0	0	0	0	1orta-d1orta1 (3_58_1_1)	1psda-d1psda2 (3_19_1_4)	1dhpa-d1dhpa (3_1_3_1)	1miob-d1miob (3_67_1_1)	1hrda-d1hrda1 (3_19_1_7)	2dri-d2dri (3_72_1_1)	1scub-d1scub1 (3_13_3_1)	1pea-d1pea (3_72_1_1)	2chr-d2chr_1 (3_1_6_2)
646	1rada-d1raaa2	3_58_1_1	160	4	1	1	0	0	0	0	1orta-d1orta2 (3_58_1_1)	2naca-d2naca2 (3_19_1_4)	1gdha-d1gdha2 (3_19_1_4)	1nai-d1xel (3_19_1_2)	1rvva-d1rvva (3_9_1_1)	3tgl-d3tgl (3_50_1_7)	1wsyb-d1wsyb (3_59_1_1)	1xzl-d1cus (3_13_7_1)	1htt-d1htta1 (3_37_1_1)
647	1rahb-d1raab2	7_32_5_1	53	1	0	0	0	0	0	0	2bfh-d2fgf (2_28_1_1)	4aahc-d4aaha (2_47_1_1)	1dlc-d1dlc_2 (2_53_2_1)	1mne-d1mmd_1 (2_21_3_1)	1idm-d1idm (3_57_1_1)	1gdob-d1gdoa (4_88_1_1)	1udii-d1udii (4_14_4_1)	2prd-d2prd (2_26_5_1)	2pola-d2pola2 (4_76_1_1)
648	1ray-d2cba	2_50_1_1	258	1	0	0	0	0	0	0	1nsdb-d1nsca (2_45_1_1)	1svb-d1svb_2 (6_10_1_1)	1msad-d1msaa (2_54_1_1)	1udii-d1udii (4_14_4_1)	1plq-d1plq_1 (4_76_1_2)	1ciy-d1ciy_2 (2_53_2_1)	1llo-d1hvq (3_1_1_5)	1inp-d1inp (5_8_1_3)	1kit-d1kit_1 (2_19_1_6)
649	1rcd-d1rcd	1_24_1_1	171	3	1	1	1	1	0	0	1bfra-d1bcfa (1_24_1_1)	1ryt-d1ryt_1 (1_24_1_1)	1afra-d1afra (1_24_1_2)	2liga-d2liga (1_23_2_1)	1occa-d1occa1 (6_5_1_1)	1ciy-d1ciy_3 (6_1_3_1)	1occc-d1occc1 (6_5_1_1)	1buca-d1buca1 (1_23_6_1)	1xsm-d1xsm (1_24_1_2)
650	1rcf-d1rcf	3_13_4_1	169	3	1	1	0	0	0	0	1fld-d5nul (3_13_4_1)	3fx2-d2fx2 (3_13_4_1)	1mpd-d1mpb (3_73_1_1)	4mhta-d1hmy (3_47_1_4)	1nai-d1xel (3_19_1_2)	1wsyb-d1wsyb (3_59_1_1)	1dcta-d1dcta (3_47_1_4)	2phy-d2phy (4_61_2_1)	1amp-d1amp (3_52_3_4)
651	1rcpa-d1cpq	1_23_3_2	129	3	1	1	0	0	0	1	1bbha-d1bbha (1_23_3_2)	2ccya-d2ccya (1_23_3_2)	1occc-d1occc1 (6_5_1_1)	1bfra-d1bcfa (1_24_1_1)	1aep-d1aep (1_49_1_1)	2liga-d2liga (1_23_2_1)	1ryt-d1ryt_1 (1_24_1_1)	1afra-d1afra (1_24_1_2)	1glm-d1glm (1_73_1_1)
652	1rdd-d2rn2	3_41_3_1	155	1	0	0	0	0	0	0	2lbp-d2lbp (3_72_1_1)	2dri-d2dri (3_72_1_1)	1scua-d1scua1 (3_13_3_1)	1gtma-d1gtma2 (3_54_1_1)	2chr-d2chr_1 (3_1_6_2)	1cowe-d1bmfd3 (3_25_1_6)	1chma-d1chma2 (4_72_1_1)	1nfp-d1nfp (3_1_13_2)	1miob-d1miob (3_67_1_1)
653	1regx-d1regx	4_33_19_1	122	1	0	0	0	0	0	0	1poba-d1poa (1_95_1_2)	1miob-d1miob (3_67_1_1)	2csn-d1csn (5_1_1_1)	2dri-d2dri (3_72_1_1)	1rpl-d1rpl (5_10_1_1)	1phk-d1phk (5_1_1_1)	1ecpa-d1ecpa (3_52_1_1)	1lucb-d1lucb (3_1_13_1)	1nfp-d1nfp (3_1_13_2)
654	1reqb-d1reqb2	3_13_5_2	163	1	0	0	0	0	0	0	2dri-d2dri (3_72_1_1)	1oya-d1oya (3_1_7_1)	1pea-d1pea (3_72_1_1)	1lucb-d1lucb (3_1_13_1)	1nal1-d1nal1 (3_1_3_1)	1dppc-d1dppa (3_73_1_1)	1pnr-d1pnra2 (3_72_1_1)	3btoa-d2ohxa2 (3_19_1_1)	1tlfa-d1tlfa (3_72_1_1)
655	1ret-d1res	1_4_1_2	43	2	0	1	0	1	0	0	1perl-d1r69 (1_30_1_2)	1rnl-d1rnl_1 (1_31_1_2)	1pdn-d1pdnc (1_4_1_4)	1dik-d1dik_3 (4_83_1_4)	1hcr-d1hcra (1_4_1_2)	1ciy-d1ciy_3 (6_1_3_1)	1llib-d1llia (1_30_1_2)	1octc-d1octc2 (1_30_1_1)	1copd-d1copd (1_30_1_2)
656	1rfba-d1rfba	1_25_1_3	119	2	0	0	0	0	0	0	1fps-d1fps (1_91_1_1)	1pgo-d2pgd_1 (1_71_1_1)	1knya-d1knya (5_10_1_2)	1idm-d1idm (3_57_1_1)	2sas-d2sas (1_34_1_5)	1bip-d1bip (1_42_1_2)	1csr-d1csh (1_74_1_1)	1apa-d1apa (4_94_1_1)	1oxa-d1oxa (1_75_1_1)
657	1rgs-d1rgs_1	2_58_3_2	130	2	1	1	1	1	0	0	1rgs-d1rgs_2 (2_58_3_2)	1runb-d1cgpa2 (2_58_3_1)	1prch-d1prch1 (2_27_1_1)	1ytn-d1ypta (3_32_1_2)	1phe-d1phb (1_75_1_1)	1lcl-d1lcl (2_19_1_3)	1gln-d1gln_2 (3_15_1_1)	1sra-d1sra (1_34_1_3)	1nsdb-d1nsca (2_45_1_1)
658	1rgs-d1rgs_2	2_58_3_2	134	2	1	1	1	1	0	1	1rgs-d1rgs_1 (2_58_3_2)	1runb-d1cgpa2 (2_58_3_1)	1iyu-d1iyu (2_59_1_1)	1csei-d1csei (4_22_1_1)	1ad2-d1ad2 (5_20_1_1)	2phl-d2phla1 (2_58_1_1)	1bbpa-d1bbpa (2_41_1_1)	2hmb-d1hms (2_41_1_2)	1hgea-d1hgea (2_14_1_2)
659	1rie-d1rie	7_33_1_2	127	1	0	0	0	0	0	0	1mrj-d1mrj (4_94_1_1)	1hpm-d1hpm_1 (3_41_1_1)	4fua-d1fua (3_55_1_1)	2bfh-d2fgf (2_28_1_1)	1psda-d1psda3 (4_33_15_1)	1ouna-d1ouna (4_14_3_2)	1cnf-d2cnd_1 (2_29_1_1)	1mpmb-d1mal (6_7_1_2)	1prch-d1prch1 (2_27_1_1)
660	1rip-d1rip	2_26_4_6	81	1	0	0	0	0	0	0	2pec-d2pec (2_56_1_1)	4aahc-d4aaha (2_47_1_1)	1hgea-d1hgea (2_14_1_2)	2act-d2act (4_3_1_1)	1vcaa-d1vcaa1 (2_1_1_3)	1lac-d1lac (2_59_1_1)	1cdcb-d1cdcb (2_1_1_1)	1ten-d1ten (2_1_2_1)	1dlc-d1dlc_2 (2_53_2_1)
661	1ris-d1ris	4_33_11_1	97	1	0	0	0	0	1	1	1psda-d1psda3 (4_33_15_1)	1pil-d1pil (4_33_5_1)	1spbp-d1spbp (4_33_3_2)	1rpl-d1rpl (5_10_1_1)	2ebn-d2ebn (3_1_1_5)	1tys-d1tys (4_63_1_1)	1tig-d1tig (4_35_1_1)	1zooa-d1lfaa (3_45_1_1)	1xra-d1mxa_2 (4_75_1_1)
662	1rldb-d3rubl2	4_33_9_1	121	1	0	0	0	0	0	1	1mbb-d1mbb_1 (4_84_1_2)	1azca-d1aiza (2_5_1_1)	1kte-d1kte (3_33_1_1)	1gtqa-d1gtqa (4_54_1_2)	2bop-d2bopa (4_33_8_1)	1ris-d1ris (4_33_11_1)	1pea-d1pea (3_72_1_1)	1mioa-d1mioa (3_67_1_1)	1lucb-d1lucb (3_1_13_1)
663	1rlr-d1rlr_1	1_68_1_1	212	1	0	0	0	0	0	0	1csr-d1csh (1_74_1_1)	1hvd-d1hvd (1_51_1_1)	1tcob-d1tcob (1_34_1_5)	1faga-d2bmha (1_75_1_1)	1mtyd-d1mmod (1_24_1_2)	1prcl-d1prcl1 (6_5_1_1)	1afra-d1afra (1_24_1_2)	1pgo-d2pgd_1 (1_71_1_1)	2abk-d2abk (1_66_1_1)
664	1rnl-d1rnl_1	1_31_1_2	62	1	0	0	0	0	0	0	1sly-d1sly_1 (1_84_4_1)	1pdn-d1pdnc (1_4_1_4)	2wrpr-d2wrpr (1_78_1_1)	1vola-d1vola1 (1_59_1_2)	1llib-d1llia (1_30_1_2)	1paw-d1pax_1 (1_35_1_1)	1gtma-d1gtma1 (3_19_1_7)	1vin-d1vin_1 (1_59_1_1)	1octc-d1octc2 (1_30_1_1)
665	1rnl-d1rnl_2	3_13_2_1	138	4	1	1	0	0	0	0	1srrc-d1srra (3_13_2_1)	3chy-d3chy (3_13_2_1)	1qora-d1qora2 (3_19_1_1)	2ada-d1add (3_1_2_1)	1tlfa-d1tlfa (3_72_1_1)	1igs-d1igs (3_1_8_1)	2lbp-d2lbp (3_72_1_1)	1vid-d1vid (3_47_1_1)	1bmfc-d1bmfa3 (3_25_1_6)
666	1rof-d1vjw	4_33_1_4	59	3	1	1	1	1	0	0	1fxra-d1fxra (4_33_1_4)	2fxb-d2fxb (4_33_1_4)	1fca-d1fca (4_33_1_1)	1fd2-d1fd2 (4_33_1_2)	1ggt-d1ggta4 (4_3_1_2)	2dkb-d2dkb (3_48_1_3)	3mdsa-d1mnga2 (4_25_1_1)	1tys-d1tys (4_63_1_1)	1ncs-d1ncs (7_28_1_1)
667	1roo-d1roo	7_16_1_1	35	2	0	0	0	0	0	0	1qrda-d1qrda (3_13_4_2)	1etd-d1etd (1_4_3_9)	1ciy-d1ciy_3 (6_1_3_1)	1occa-d1occa1 (6_5_1_1)	1prcl-d1prcl1 (6_5_1_1)	1orda-d1orda2 (3_48_1_4)	1fct-d1fct (8_31_1_1)	1fps-d1fps (1_91_1_1)	1chra-d2chr_2 (4_31_1_1)
668	1rpa-d1rpa	3_43_1_2	342	1	0	0	0	0	0	0	1prcl-d1prcl1 (6_5_1_1)	1dik-d1dik_3 (4_83_1_4)	1csr-d1csh (1_74_1_1)	1gsea-d1gsea1 (1_38_1_1)	1tfe-d1tfe (4_24_1_1)	1faga-d2bmha (1_75_1_1)	1chka-d1chka (4_2_1_6)	1hvd-d1hvd (1_51_1_1)	1occa-d1occa1 (6_5_1_1)

669	1rpf-d7rsa	4_4_1_1	124	1	0	0	0	0	0	0	1clc-d1clc_2 (2_1_1_5)	1jeva-d2olba (3_73_1_1)	2sil-d2sil (2_45_1_1)	2pec-d2pec (2_56_1_1)	1pea-d1pea (3_72_1_1)	1vcc-d1vcc (4_67_1_1)	1ospo-d1ospo (2_52_1_1)	1aco-d1aco_1 (3_5_2_1)	1nis-d1aco_2 (3_63_1_1)
670	1rpl-d1rpl	5_10_1_1	241	1	0	0	0	0	0	0	1asza-d1asya2 (4_59_1_1)	1lyla-d1lyla2 (4_59_1_1)	1otga-d1otga (4_40_1_2)	1phk-d1phk (5_1_1_1)	2dkb-d2dkb (3_48_1_3)	1gtma-d1gtma1 (3_19_1_7)	1eac-d1eaf (3_30_1_1)	7icd-d7icd (3_57_1_1)	1ris-d1ris (4_33_11_1)
671	1rpo-d1rpo	1_27_1_1	61	1	0	0	0	0	0	0	1bfra-d1bcfa (1_24_1_1)	1lpe-d1lpe (1_23_1_1)	1vsga-d1vsga (6_3_1_1)	1lki-d1lki (1_25_1_1)	1bct-d1bct (8_30_1_1)	1ciy-d1ciy_3 (6_1_3_1)	1rcd-d1rcd (1_24_1_1)	1fosf-d1fosf (1_97_2_1)	1sesa-d1seta1 (1_2_3_1)
672	1rro-d1rro	1_34_1_4	108	1	0	0	1	1	0	0	1tcob-d1tcob (1_34_1_5)	1osa-d1osa (1_34_1_5)	2sas-d2sas (1_34_1_5)	1wdcb-d1scmb (1_34_1_5)	2scpa-d2scpa (1_34_1_5)	1sra-d1sra (1_34_1_3)	1tnda-d1tada1 (1_52_1_1)	1sly-d1sly_1 (1_84_4_1)	1bia-d1bia_1 (1_4_3_1)
673	1rsy-d1rsy	2_6_1_2	135	1	0	0	1	1	0	0	1qatb-d1djxa2 (2_6_1_1)	1vcaa-d1vcaa1 (2_1_1_3)	1hgea-d1hgea (2_14_1_2)	1ospo-d1ospo (2_52_1_1)	2trcb-d2trcb (2_46_3_1)	1udii-d1udii (4_14_4_1)	1pgs-d1pgs_1 (2_11_1_1)	1eur-d1eur (2_45_1_1)	1ouna-d1ouna (4_14_3_2)
674	1rtm2-d1rtm12	4_97_1_1	117	2	1	1	0	0	0	0	1esl-d1esl_1 (4_97_1_1)	1srrc-d1srra (3_13_2_1)	2ebn-d2ebn (3_1_1_5)	1nai-d1xel (3_19_1_2)	1cur-d1cur (2_5_1_1)	1hrda-d1hrda1 (3_19_1_7)	1scub-d1scub2 (4_83_1_3)	1gph1-d1gph12 (4_88_1_1)	3dfr-d3dfr (3_53_1_1)
675	1runb-d1cgpa2	2_58_3_1	129	1	0	0	1	1	0	1	1rgs-d1rgs_2 (2_58_3_2)	1rgs-d1rgs_1 (2_58_3_2)	2csn-d1csn (5_1_1_1)	1lxa-d1lxa (2_57_1_1)	1hmda-d2hmqa (1_23_4_1)	1xsm-d1xsm (1_24_1_2)	2phl-d2phla1 (2_58_1_1)	1bfra-d1bcfa (1_24_1_1)	1msk-d1msk (4_99_1_1)
676	1rvea-d1rvaa	3_38_1_2	244	1	0	0	0	0	0	0	1lyla-d1lyla2 (4_59_1_1)	1ltge-d1ltsd (2_26_2_1)	2sil-d2sil (2_45_1_1)	1qba-d1qba_1 (2_1_1_5)	1asza-d1asya2 (4_59_1_1)	1ad2-d1ad2 (5_20_1_1)	1oya-d1oya (3_1_7_1)	2tmda-d2tmda1 (3_1_7_1)	1rcd-d1rcd (1_24_1_1)
677	1rvva-d1rvva	3_9_1_1	154	1	0	0	0	0	0	0	1dppc-d1dppa (3_73_1_1)	1ahia-d1fmca (3_19_1_2)	2dri-d2dri (3_72_1_1)	1cowe-d1bmfd3 (3_25_1_6)	1asu-d1asu (3_41_3_2)	2hnp-d2hnp (3_32_1_2)	1mioa-d1mioa (3_67_1_1)	1tlfa-d1tlfa (3_72_1_1)	1bmfc-d1bmfa3 (3_25_1_6)
678	1ryt-d1ryt_1	1_24_1_1	146	3	1	1	1	1	0	0	1bfra-d1bcfa (1_24_1_1)	1rcd-d1rcd (1_24_1_1)	1mrrb-d1riba (1_24_1_2)	1afra-d1afra (1_24_1_2)	1occc-d1occc1 (6_5_1_1)	2liga-d2liga (1_23_2_1)	1aep-d1aep (1_49_1_1)	1ciy-d1ciy_3 (6_1_3_1)	1lpe-d1lpe (1_23_1_1)
679	1ryt-d1ryt_2	7_32_4_1	44	2	0	0	0	0	0	0	2aky-d1aky (3_25_1_1)	2hnp-d2hnp (3_32_1_2)	1wba-d1wba (2_28_3_1)	1pms-d1pms (2_37_1_1)	1pex-d1pex (2_44_1_1)	1dar-d1dar_1 (2_29_3_1)	1ptq-d1ptq (7_38_1_1)	1alo-d1alo_7 (4_77_1_1)	2qila-d1tssa2 (4_12_5_1)
680	1sacb-d1saca	2_19_1_4	204	1	0	0	1	1	0	0	1lcl-d1lcl (2_19_1_3)	1tnfa-d1tnfa (2_17_1_1)	2bbkh-d2bbkh (2_46_2_1)	1kit-d1kit_1 (2_19_1_6)	2hmb-d1hms (2_41_1_2)	1bbpa-d1bbpa (2_41_1_1)	1kit-d1kit_2 (2_19_1_6)	1ospo-d1ospo (2_52_1_1)	3nn9-d3nn9 (2_45_1_1)
681	1sala-d1olga	1_44_1_1	42	1	0	0	0	0	0	0	1ysac-d2ztaa (1_97_2_1)	1occd-d1occd1 (6_5_1_1)	1tfe-d1tfe (4_24_1_1)	1tys-d1tys (4_63_1_1)	2bop-d2bopa (4_33_8_1)	1kob-d1koba (5_1_1_1)	1grj-d1grj_1 (1_2_1_1)	1apme-d1apme (5_1_1_1)	1rvea-d1rvaa (3_38_1_2)
682	1sbp-d1sbp	3_73_1_1	309	8	1	1	0	0	0	0	1poy1-d1pot (3_73_1_1)	1mpd-d1mpb (3_73_1_1)	1ggga-d1ggga (3_73_1_1)	1tlfa-d1tlfa (3_72_1_1)	2dri-d2dri (3_72_1_1)	1idm-d1idm (3_57_1_1)	1oya-d1oya (3_1_7_1)	2lbp-d2lbp (3_72_1_1)	1oiba-d1pbp (3_73_1_1)
683	1scb-d1csee	3_28_1_1	274	1	0	0	0	0	0	0	1eceb-d1ecea (3_1_1_3)	1scua-d1scua2 (3_19_1_8)	1prhb-d1prha1 (1_65_1_2)	3chy-d3chy (3_13_2_1)	1gln-d1gln_2 (3_15_1_1)	1xva-d1xvaa (3_47_1_2)	1tlfa-d1tlfa (3_72_1_1)	1bgl-d1bgla5 (3_1_1_3)	1xyza-d1xyza (3_1_1_3)
684	1scea-d1scea	4_55_1_1	97	1	0	0	0	0	0	0	1msk-d1msk (4_99_1_1)	1kcw-d1kcw_1 (2_5_1_3)	2anhb-d2anha (3_56_1_1)	1plq-d1plq_1 (4_76_1_2)	1pyda-d1pvda3 (3_24_1_1)	1phk-d1phk (5_1_1_1)	1rldb-d3rubl2 (4_33_9_1)	2phy-d2phy (4_61_2_1)	2ebn-d2ebn (3_1_1_5)
685	1scha-d1scha	1_65_1_1	292	1	0	0	0	0	0	0	3sdha-d3sdha (1_1_1_1)	1sly-d1sly_1 (1_84_4_1)	1cem-d1cem (1_73_1_2)	2hbg-d1hbg (1_1_1_1)	2gpb-d1gpb (3_68_1_2)	1occa-d1occa1 (6_5_1_1)	1glm-d1glm (1_73_1_1)	1gcb-d1gcb (4_3_1_1)	1clc-d1clc_1 (1_73_1_2)
686	1scua-d1scua1	3_13_3_1	167	2	1	1	0	0	0	0	1scub-d1scub1 (3_13_3_1)	1bll-d1lam_2 (3_52_3_3)	1mioa-d1mioa (3_67_1_1)	1tlfa-d1tlfa (3_72_1_1)	1nal1-d1nal1 (3_1_3_1)	1psda-d1psda3 (4_33_15_1)	1nhq-d1nhp_2 (3_4_1_4)	2lbp-d2lbp (3_72_1_1)	2bnh-d1dfji (3_7_1_1)
687	1scua-d1scua2	3_19_1_8	121	1	0	0	1	1	0	0	1pgn-d2pgd_2 (3_19_1_6)	1nai-d1xel (3_19_1_2)	1psda-d1psda2 (3_19_1_4)	2lbp-d2lbp (3_72_1_1)	1reqb-d1reqb2 (3_13_5_2)	1scb-d1csee (3_28_1_1)	1tlfa-d1tlfa (3_72_1_1)	1ecpa-d1ecpa (3_52_1_1)	1sfta-d1sfta2 (3_1_18_1)
688	1scub-d1scub1	3_13_3_1	144	2	1	1	0	0	1	1	1scua-d1scua1 (3_13_3_1)	1bmta-d1bmta2 (3_13_5_1)	1miob-d1miob (3_67_1_1)	2lbp-d2lbp (3_72_1_1)	2dri-d2dri (3_72_1_1)	1tlfa-d1tlfa (3_72_1_1)	1cowe-d1bmfd3 (3_25_1_6)	1pnr-d1pnra2 (3_72_1_1)	1nal1-d1nal1 (3_1_3_1)
689	1scub-d1scub2	4_83_1_3	244	1	0	0	1	1	0	0	1iov-d2dln_2 (4_83_1_1)	1eft-d1eft_3 (3_25_1_3)	2tmda-d2tmda1 (3_1_7_1)	1bmta-d1bmta2 (3_13_5_1)	1igs-d1igs (3_1_8_1)	1hrda-d1hrda1 (3_19_1_7)	1dik-d1dik_3 (4_83_1_4)	1srsa-d1srsa (4_47_1_1)	3tgl-d3tgl (3_50_1_7)
690	1sea-d1esfa2	4_12_5_1	113	2	1	1	0	0	0	0	2qila-d1tssa2 (4_12_5_1)	1bcpb-d1prtb1 (2_26_2_1)	1udii-d1udii (4_14_4_1)	3pte-d3pte (5_4_1_1)	1noa-d1noa (2_1_6_1)	1noy-d1noya (3_41_3_5)	1ngi-d1hpm_2 (3_41_1_1)	1cowe-d1bmfd3 (3_25_1_6)	1ciy-d1ciy_2 (2_53_2_1)
691	1seia-d1seia	4_80_1_1	130	1	0	0	0	0	0	0	2gpb-d1gpb (3_68_1_2)	1mpd-d1mpb (3_73_1_1)	1jeva-d2olba (3_73_1_1)	1taha-d1tahb (3_50_1_8)	1scb-d1csee (3_28_1_1)	1rpa-d1rpa (3_43_1_2)	1daaa-d1daaa (5_14_1_1)	1nal1-d1nal1 (3_1_3_1)	1dppc-d1dppa (3_73_1_1)
692	1sema-d1sema	2_21_2_1	58	4	1	1	0	0	0	1	1aey-d1shg (2_21_2_1)	1hsq-d1hsq (2_21_2_1)	1pht-d1pht (2_21_2_1)	1sty-d1sty (2_26_1_1)	1ihva-d1ihwa (2_21_6_1)	1hfi-d1hfi (7_15_1_1)	1hcd-d1hcd (2_28_4_1)	1tnj-d1bty (2_31_1_2)	1ecpa-d1ecpa (3_52_1_1)
693	1sesa-d1seta1	1_2_3_1	110	1	0	0	0	0	0	0	1afra-d1afra (1_24_1_2)	1lpe-d1lpe (1_23_1_1)	1bfra-d1bcfa (1_24_1_1)	1occc-d1occc1 (6_5_1_1)	1vsga-d1vsga (6_3_1_1)	1dlc-d1dlc_3 (6_1_3_1)	1dkza-d1dkza (5_17_1_1)	1aep-d1aep (1_49_1_1)	1rcd-d1rcd (1_24_1_1)
694	1seta-d1seta2	4_59_1_1	311	3	0	1	0	0	0	0	1tys-d1tys (4_63_1_1)	1asza-d1asya2 (4_59_1_1)	1lyla-d1lyla2 (4_59_1_1)	1oiba-d1pbp (3_73_1_1)	1mbt-d1mbb_2 (4_85_1_1)	1jeva-d2olba (3_73_1_1)	1mioa-d1mioa (3_67_1_1)	1cyda-d1cyda (3_19_1_2)	1eac-d1eaf (3_30_1_1)
695	1sfe-d1sfe_1	1_4_2_1	84	1	0	0	0	0	0	1	1glm-d1glm (1_73_1_1)	1opc-d1opc (1_4_3_5)	1tpfa-d1tpfa (3_1_11_1)	1occe-d1occe (1_84_7_1)	1sra-d1sra (1_34_1_3)	1xyza-d1xyza (3_1_1_3)	1bll-d1lam_2 (3_52_3_3)	1lea-d1lea (1_4_3_2)	1dhr-d1dhr (3_19_1_2)
696	1sfta-d1sfta2	3_1_18_1	233	1	0	0	0	0	0	1	1idm-d1idm (3_57_1_1)	1igs-d1igs (3_1_8_1)	1tlfa-d1tlfa (3_72_1_1)	1art-d1art (3_48_1_1)	1qora-d1qora2 (3_19_1_1)	1ghr-d1ghr (3_1_1_3)	1dora-d1dora (3_1_7_1)	2dri-d2dri (3_72_1_1)	1oiba-d1pbp (3_73_1_1)
697	1sgk-d1ddt_2	4_95_1_1	187	1	0	0	1	1	0	0	1pax-d1pax_2 (4_95_1_2)	1eft-d1eft_3 (3_25_1_3)	2phl-d2phla1 (2_58_1_1)	1amp-d1amp (3_52_3_4)	1gen-d1gen (2_44_1_1)	1eac-d1eaf (3_30_1_1)	2pcd-d2pcda (2_3_3_1)	1vjs-d1bplb1 (2_48_1_1)	1nal1-d1nal1 (3_1_3_1)
698	1sgpi-d1sgpi	7_12_1_1	51	5	1	1	0	0	0	0	1tbrr-d1tbrr2 (7_12_1_1)	1tbqr-d1tbrr1 (7_12_1_1)	1tgsi-d1tgsi (7_12_1_1)	1pce-d1pce (7_12_1_1)	1nal1-d1nal1 (3_1_3_1)	1art-d1art (3_48_1_1)	3pmga-d3pmga1 (3_64_1_1)	1gky-d1gky (3_25_1_1)	1dhpa-d1dhpa (3_1_3_1)
699	1shaa-d1shaa	4_51_1_1	103	3	1	1	0	0	0	0	2plda-d2plda (4_51_1_1)	1wba-d1wba (2_28_3_1)	2csn-d1csn (5_1_1_1)	1obpa-d1obpa (2_41_1_1)	1con-d1scs (2_19_1_1)	1tys-d1tys (4_63_1_1)	1nal1-d1nal1 (3_1_3_1)	1xva-d1xvaa (3_47_1_2)	1ctt-d1ctt_1 (3_75_1_1)
700	1shca-d1shca	2_37_1_2	195	2	0	0	0	1	0	0	1nsdb-d1nsca (2_45_1_1)	1asya-d1asya1 (2_26_4_1)	1pms-d1pms (2_37_1_1)	1nfp-d1nfp (3_1_13_2)	1kit-d1kit_1 (2_19_1_6)	2trcb-d2trcb (2_46_3_1)	1sva5-d1sva1 (2_8_1_4)	7icd-d7icd (3_57_1_1)	2pec-d2pec (2_56_1_1)
701	1slba-d1slaa	2_19_1_3	134	2	1	1	0	1	0	0	1lcl-d1lcl (2_19_1_3)	2hmb-d1hms (2_41_1_2)	1sacb-d1saca (2_19_1_4)	4aahc-d4aaha (2_47_1_1)	2omf-d2omf (6_7_1_1)	1prn-d1prn (6_7_1_1)	1ospo-d1ospo (2_52_1_1)	1ure-d1ifc (2_41_1_2)	2trcb-d2trcb (2_46_3_1)
702	1sly-d1sly_1	1_84_4_1	450	1	0	0	0	0	0	0	1occa-d1occa1 (6_5_1_1)	1pgo-d2pgd_1 (1_71_1_1)	1csr-d1csh (1_74_1_1)	1hvd-d1hvd (1_51_1_1)	1occc-d1occc1 (6_5_1_1)	1mrrb-d1riba (1_24_1_2)	1afra-d1afra (1_24_1_2)	1ciy-d1ciy_3 (6_1_3_1)	1xsm-d1xsm (1_24_1_2)
703	1sly-d1sly_2	4_2_1_5	168	1	0	0	1	1	0	0	1gbs-d1gbs (4_2_1_4)	2wrpr-d2wrpr (1_78_1_1)	119l-d119l (4_2_1_3)	1sly-d1sly_1 (1_84_4_1)	2myd-d1mbd (1_1_1_1)	1tfe-d1tfe (4_24_1_1)	1chka-d1chka (4_2_1_6)	1afra-d1afra (1_24_1_2)	1xsm-d1xsm (1_24_1_2)
704	1smna-d1smna	4_71_1_1	241	1	0	0	0	0	0	0	1mkaa-d1mkaa (4_21_1_1)	3dfr-d3dfr (3_53_1_1)	1scub-d1scub2 (4_83_1_3)	1bgl-d1bgla5 (3_1_1_3)	1eny-d1eny (3_19_1_2)	1ahsa-d1ahsa (2_14_1_1)	1ytn-d1ypta (3_32_1_2)	1chma-d1chma2 (4_72_1_1)	2bfh-d2fgf (2_28_1_1)
705	1smpi-d1smpi	2_42_2_1	100	1	0	0	0	0	0	0	1kxa-d2snv (2_31_1_3)	2sil-d2sil (2_45_1_1)	1ospo-d1ospo (2_52_1_1)	1wit-d1wiu (2_1_1_4)	1bgl-d1bgla1 (2_1_3_1)	1pls-d1pls (2_37_1_1)	1tlk-d1tlk (2_1_1_4)	2trcb-d2trcb (2_46_3_1)	1jeva-d2olba (3_73_1_1)
706	1smvc-d1smva	2_8_1_2	196	4	0	1	1	1	0	0	2bbvc-d2bbva (2_8_1_3)	2plv-d1pvc1 (2_8_1_4)	1art-d1art (3_48_1_1)	2stv-d2stv (2_8_1_2)	2cas-d2cas (2_8_1_4)	1kfd-d1kfd_1 (3_41_3_4)	1daaa-d1daaa (5_14_1_1)	2gd1o-d1gd1o2 (4_41_1_1)	1mml-d1mml (5_9_1_2)
707	1spbp-d1spbp	4_33_3_2	71	1	0	0	0	0	1	1	1ris-d1ris (4_33_11_1)	1scua-d1scua1 (3_13_3_1)	1scub-d1scub1 (3_13_3_1)	1gph1-d1gph11 (3_44_1_1)	1dhpa-d1dhpa (3_1_3_1)	1psda-d1psda3 (4_33_15_1)	1eny-d1eny (3_19_1_2)	1hrda-d1hrda1 (3_19_1_7)	2tmda-d2tmda1 (3_1_7_1)
708	1spf-d1spf	8_30_1_1	35	2	0	0	0	0	0	0	1rpo-d1rpo (1_27_1_1)	1asu-d1asu (3_41_3_2)	1occc-d1occc1 (6_5_1_1)	1aep-d1aep (1_49_1_1)	2hmgd-d1htmb (6_2_1_1)	1clc-d1clc_1 (1_73_1_2)	1bfra-d1bcfa (1_24_1_1)	1fipa-d1fipa (1_76_1_1)	1div-d1div (4_82_1_1)
709	1sra-d1sra	1_34_1_3	151	1	0	0	1	1	0	0	2scpa-d2scpa (1_34_1_5)	1tcob-d1tcob (1_34_1_5)	1osa-d1osa (1_34_1_5)	2sas-d2sas (1_34_1_5)	1wdcb-d1scmb (1_34_1_5)	1pbwa-d1pbwa (1_83_1_1)	1rro-d1rro (1_34_1_4)	1csr-d1csh (1_74_1_1)	1cola-d1cola (6_1_1_1)
710	1sro-d1sro	2_26_4_4	76	2	1	1	0	0	0	1	1mjc-d1mjc (2_26_4_4)	1pex-d1pex (2_44_1_1)	1pky-d1pkya1 (2_40_1_1)	1qila-d1tssa1 (2_26_2_2)	1gen-d1gen (2_44_1_1)	1dlc-d1dlc_2 (2_53_2_1)	1tcmb-d1cdg_1 (2_1_1_5)	2prd-d2prd (2_26_5_1)	1cpn-d1cpn (2_19_1_2)
711	1srrc-d1srra	3_13_2_1	119	4	1	1	0	0	1	1	3chy-d3chy (3_13_2_1)	1rnl-d1rnl_2 (3_13_2_1)	1scub-d1scub1 (3_13_3_1)	1igs-d1igs (3_1_8_1)	1nsj-d1nsj (3_1_8_1)	1ahia-d1fmca (3_19_1_2)	1dppc-d1dppa (3_73_1_1)	1tlfa-d1tlfa (3_72_1_1)	6ldh-d1ldm_1 (3_19_1_5)
712	1srsa-d1srsa	4_47_1_1	84	1	0	0	0	0	0	0	1dik-d1dik_3 (4_83_1_4)	1itea-d2int (1_25_1_2)	1gcb-d1gcb (4_3_1_1)	1scub-d1scub2 (4_83_1_3)	1ctt-d1ctt_2 (3_75_1_1)	1mrj-d1mrj (4_94_1_1)	1hdca-d1hdca (3_19_1_2)	1cyda-d1cyda (3_19_1_2)	1seta-d1seta2 (4_59_1_1)
713	1sso-d1sso	4_9_1_1	62	1	0	0	0	0	0	0	1noy-d1noya (3_41_3_5)	1irsa-d1irsa (2_37_1_2)	1eal-d1eal (2_41_1_2)	3pte-d3pte (5_4_1_1)	3nn9-d3nn9 (2_45_1_1)	1smpi-d1smpi (2_42_2_1)	1ouna-d1ouna (4_14_3_2)	1gof-d1gof_3 (2_46_1_1)	1ospo-d1ospo (2_52_1_1)
714	1stfi-d1stfi	4_14_1_2	98	2	0	0	1	1	0	0	1mola-d1mola (4_14_1_1)	1obpa-d1obpa (2_41_1_1)	1pda-d1pda_2 (4_28_2_1)	3tgl-d3tgl (3_50_1_7)	1febb-d2tpra2 (3_4_1_4)	1dih-d1dih_2 (4_41_1_2)	1mkaa-d1mkaa (4_21_1_1)	1dcha-d1dcoa (4_38_1_1)	1chma-d1chma1 (3_41_2_1)
715	1stu-d1stu	4_28_1_1	68	1	0	0	0	0	0	1	1qbb-d1qba_4 (4_50_2_1)	1han-d1han_1 (4_20_1_3)	1pda-d1pda_2 (4_28_2_1)	1lyla-d1lyla2 (4_59_1_1)	1rdd-d2rn2 (3_41_3_1)	1asu-d1asu (3_41_3_2)	1tys-d1tys (4_63_1_1)	1chma-d1chma2 (4_72_1_1)	1asza-d1asya2 (4_59_1_1)
716	1sty-d1sty	2_26_1_1	137	1	0	0	0	0	0	0	1gph1-d1gph11 (3_44_1_1)	1llo-d1hvq (3_1_1_5)	1nfp-d1nfp (3_1_13_2)	1chd-d1chd (3_27_1_1)	1vhia-d1vhia (4_33_8_1)	1amp-d1amp (3_52_3_4)	1vhh-d1vhh (4_34_1_2)	1xyza-d1xyza (3_1_1_3)	1daaa-d1daaa (5_14_1_1)

717	1sva5-d1sva1	2_8_1_4	347	5	0	1	0	0	0	0	1mpmb-d1mal (6_7_1_2)	1ospo-d1ospo (2_52_1_1)	2cas-d2cas (2_8_1_4)	1obpa-d1obpa (2_41_1_1)	1akl-d1kapp1 (2_55_1_1)	7cat-d7cata (5_6_1_1)	3nn9-d3nn9 (2_45_1_1)	1cpn-d1cpn (2_19_1_2)	1noa-d1noa (2_1_6_1)
718	1svb-d1svb_1	2_1_1_5	92	6	0	0	0	0	0	0	2hmb-d1hms (2_41_1_2)	1ospo-d1ospo (2_52_1_1)	1tnfa-d1tnfa (2_17_1_1)	4aahc-d4aaha (2_47_1_1)	1asob-d1aoza3 (2_5_1_3)	1tlk-d1tlk (2_1_1_4)	1nsdb-d1nsca (2_45_1_1)	1ure-d1ifc (2_41_1_2)	1gen-d1gen (2_44_1_1)
719	1svb-d1svb_2	6_10_1_1	302	1	0	0	0	0	0	0	1ospo-d1ospo (2_52_1_1)	3nn9-d3nn9 (2_45_1_1)	1pmi-d1pmi (2_58_2_1)	5sgae-d1sgc (2_31_1_1)	1bbpa-d1bbpa (2_41_1_1)	1eur-d1eur (2_45_1_1)	1bmv1-d1bmv1 (2_8_1_2)	1kit-d1kit_2 (2_19_1_6)	1bvp1-d1bvp12 (2_14_1_1)
720	1svq-d1svr	4_60_1_1	94	2	0	1	0	0	0	0	1mml-d1mml (5_9_1_2)	2vik-d2vik (4_60_1_1)	7icd-d7icd (3_57_1_1)	4kbpa-d4kbpa2 (4_91_1_1)	2tmda-d2tmda1 (3_1_7_1)	1vhh-d1vhh (4_34_1_2)	1qrda-d1qrda (3_13_4_2)	1xzl-d1cus (3_13_7_1)	1ezm-d1ezm_2 (4_50_1_2)
721	1sxl-d1sxl	4_33_7_1	97	2	1	1	0	0	0	0	1urna-d1urna (4_33_7_1)	1hmp-d1hmpa (3_44_1_1)	1tbd-d1tbd (4_48_1_1)	1scub-d1scub2 (4_83_1_3)	1llo-d1hvq (3_1_1_5)	1gph1-d1gph12 (4_88_1_1)	1fwp-d1fwp (4_33_18_1)	2sici-d2sici (4_44_1_1)	1qapa-d1qapa1 (3_1_14_1)
722	1taha-d1tahb	3_50_1_8	318	1	0	0	1	1	0	0	2dhd-d1ede (3_50_1_3)	1broa-d1broa (3_50_1_5)	1lbt-d1tca (3_50_1_7)	1yasa-d1yasa (3_50_1_10)	1gca-d1gca (3_72_1_1)	1cowe-d1bmfd3 (3_25_1_6)	2dri-d2dri (3_72_1_1)	1nox-d1nox (4_49_1_1)	1hrda-d1hrda2 (3_54_1_1)
723	1tbd-d1tbd	4_48_1_1	134	1	0	0	0	0	0	0	1psda-d1psda3 (4_33_15_1)	1plq-d1plq_1 (4_76_1_2)	1cew-d1cewi (4_14_1_2)	1nhq-d1nhp_2 (3_4_1_4)	1nsj-d1nsj (3_1_8_1)	1gph1-d1gph12 (4_88_1_1)	1jlm-d1ido (3_45_1_1)	1bebb-d1beba (2_41_1_1)	1dik-d1dik_1 (3_1_9_2)
724	1tbqr-d1tbrr1	7_12_1_1	51	5	1	1	0	0	0	0	1tbrr-d1tbrr2 (7_12_1_1)	1tgsi-d1tgsi (7_12_1_1)	1sgpi-d1sgpi (7_12_1_1)	1pce-d1pce (7_12_1_1)	1lyla-d1lyla2 (4_59_1_1)	1geua-d1gesa2 (3_4_1_4)	1tys-d1tys (4_63_1_1)	1oiba-d1pbp (3_73_1_1)	1art-d1art (3_48_1_1)
725	1tbrr-d1tbrr2	7_12_1_1	52	5	1	1	0	0	0	0	1tbqr-d1tbrr1 (7_12_1_1)	1sgpi-d1sgpi (7_12_1_1)	1tgsi-d1tgsi (7_12_1_1)	1pce-d1pce (7_12_1_1)	1aba-d1aba (3_33_1_1)	1gdob-d1gdoa (4_88_1_1)	1vcc-d1vcc (4_67_1_1)	1gat-d1gata (7_30_1_1)	1froa-d1froa (4_20_1_1)
726	1tcma-d1cdg_3	2_48_1_1	113	6	1	1	0	0	0	0	2aaa-d2aaa_1 (2_48_1_1)	1qatb-d1djxa2 (2_6_1_1)	1vjs-d1bplb1 (2_48_1_1)	1acf-d1acf (4_61_1_1)	1ospo-d1ospo (2_52_1_1)	1lvl-d1lvl_2 (3_4_1_4)	1cby-d1cby (4_58_1_1)	3nn9-d3nn9 (2_45_1_1)	1mpmb-d1mal (6_7_1_2)
727	1tcmb-d1cdg_1	2_1_1_5	86	6	0	1	0	0	1	1	1noa-d1noa (2_1_6_1)	1gog-d1gof_1 (2_1_1_5)	1msad-d1msaa (2_54_1_1)	2phl-d2phla1 (2_58_1_1)	1sro-d1sro (2_26_4_4)	1iob-d1i1b (2_28_1_2)	1cdcb-d1cdcb (2_1_1_1)	1bgl-d1bgla5 (3_1_1_3)	1fna-d1fna (2_1_2_1)
728	1tcob-d1tcob	1_34_1_5	169	5	1	1	1	1	0	0	1osa-d1osa (1_34_1_5)	2sas-d2sas (1_34_1_5)	1wdcb-d1scmb (1_34_1_5)	2scpa-d2scpa (1_34_1_5)	1rro-d1rro (1_34_1_4)	1sra-d1sra (1_34_1_3)	1rlr-d1rlr_1 (1_68_1_1)	2abk-d2abk (1_66_1_1)	1pbwa-d1pbwa (1_83_1_1)
729	1tcp-d1tcp	7_6_1_2	60	1	0	0	0	1	0	0	2sil-d2sil (2_45_1_1)	1ure-d1ifc (2_41_1_2)	1knt-d1knt (7_6_1_1)	1sso-d1sso (4_9_1_1)	1bgl-d1bgla2 (2_1_3_1)	1chma-d1chma2 (4_72_1_1)	1han-d1han_2 (4_20_1_3)	2blta-d2blta (5_4_1_1)	1chra-d2chr_2 (4_31_1_1)
730	1tde-d1tde_2	3_4_1_4	126	6	1	1	0	1	0	0	1febb-d2tpra2 (3_4_1_4)	1geua-d1gesa2 (3_4_1_4)	1nhq-d1nhp_2 (3_4_1_4)	1lvl-d1lvl_2 (3_4_1_4)	1miob-d1miob (3_67_1_1)	1gal-d1gal_1 (3_4_1_2)	2tmda-d2tmda2 (3_4_1_1)	1gtma-d1gtma1 (3_19_1_7)	1php-d1php (3_66_1_1)
731	1tdta-d1tdta	2_57_1_2	256	1	0	0	1	1	0	0	1lxa-d1lxa (2_57_1_1)	1ospo-d1ospo (2_52_1_1)	1mrrb-d1riba (1_24_1_2)	3pmga-d3pmga1 (3_64_1_1)	1p03a-d2alp (2_31_1_1)	1iyu-d1iyu (2_59_1_1)	1gky-d1gky (3_25_1_1)	1sacb-d1saca (2_19_1_4)	1occa-d1occa1 (6_5_1_1)
732	1ten-d1ten	2_1_2_1	88	4	1	1	0	0	0	1	1fna-d1fna (2_1_2_1)	1lcl-d1lcl (2_19_1_3)	1cfb-d1cfb_1 (2_1_2_1)	3hhrb-d3hhrb2 (2_1_2_1)	4kbpb-d4kbpa1 (2_1_9_1)	1wit-d1wiu (2_1_1_4)	1kit-d1kit_2 (2_19_1_6)	2por-d2por (6_7_1_1)	2omf-d2omf (6_7_1_1)
733	1tfb-d1vola2	1_59_1_2	109	2	1	1	0	0	0	0	1vola-d1vola1 (1_59_1_2)	1wsyb-d1wsyb (3_59_1_1)	1rlr-d1rlr_1 (1_68_1_1)	1octc-d1octc2 (1_30_1_1)	1gtra-d1gtra2 (3_15_1_1)	1dik-d1dik_3 (4_83_1_4)	1cowa-d1bmfa1 (1_55_1_1)	1afra-d1afra (1_24_1_2)	1vin-d1vin_1 (1_59_1_1)
734	1tfe-d1tfe	4_24_1_1	142	1	0	0	0	0	0	0	1rpa-d1rpa (3_43_1_2)	1mba-d1mba (1_1_1_1)	1buca-d1buca1 (1_23_6_1)	1hlb-d1hlb (1_1_1_1)	1sly-d1sly_2 (4_2_1_5)	1ecma-d1ecma (1_93_1_1)	1vin-d1vin_1 (1_59_1_1)	1pmi-d1pmi (2_58_2_1)	1oxa-d1oxa (1_75_1_1)
735	1tfi-d1tfi	7_32_3_1	50	1	0	0	0	0	0	0	1udii-d1udii (4_14_4_1)	2hnp-d2hnp (3_32_1_2)	1vmoa-d1vmoa (2_53_1_1)	1ure-d1ifc (2_41_1_2)	1con-d1scs (2_19_1_1)	1mrj-d1mrj (4_94_1_1)	1sacb-d1saca (2_19_1_4)	1bbpa-d1bbpa (2_41_1_1)	1lcl-d1lcl (2_19_1_3)
736	1tgsi-d1tgsi	7_12_1_1	56	5	1	1	0	0	0	0	1tbrr-d1tbrr2 (7_12_1_1)	1tbqr-d1tbrr1 (7_12_1_1)	1sgpi-d1sgpi (7_12_1_1)	1pce-d1pce (7_12_1_1)	1vmoa-d1vmoa (2_53_1_1)	1hpm-d1hpm_1 (3_41_1_1)	1gdob-d1gdoa (4_88_1_1)	1shaa-d1shaa (4_51_1_1)	2hmx-d2hmx (1_58_1_1)
737	1thw-d1thw	2_18_1_1	207	1	0	0	0	0	0	0	1eur-d1eur (2_45_1_1)	1ospo-d1ospo (2_52_1_1)	1asqa-d1aoza2 (2_5_1_3)	2pcy-d1plc (2_5_1_1)	1xnb-d1xnb (2_19_1_8)	1eri-d1eria (3_38_1_1)	4aahc-d4aaha (2_47_1_1)	1iob-d1i1b (2_28_1_2)	1gof-d1gof_3 (2_46_1_1)
738	1tib-d1tib	3_50_1_7	269	3	1	1	0	0	0	0	3tgl-d3tgl (3_50_1_7)	1cyda-d1cyda (3_19_1_2)	1mioa-d1mioa (3_67_1_1)	1xzl-d1cus (3_13_7_1)	1wsyb-d1wsyb (3_59_1_1)	1tml-d1tml (3_2_1_1)	1cnf-d2cnd_2 (3_14_1_1)	2chr-d2chr_1 (3_1_6_2)	2mnr-d2mnr_1 (3_1_6_2)
739	1tif-d1tif	4_12_6_1	76	1	0	0	0	0	0	0	1xyza-d1xyza (3_1_1_3)	1asza-d1asya2 (4_59_1_1)	1art-d1art (3_48_1_1)	1tlfa-d1tlfa (3_72_1_1)	1mbt-d1mbb_2 (4_85_1_1)	1qba-d1qba_3 (3_1_1_6)	2anhb-d2anha (3_56_1_1)	1bfra-d1bcfa (1_24_1_1)	1acf-d1acf (4_61_1_1)
740	1tig-d1tig	4_35_1_1	88	1	0	0	0	0	0	0	1cbg-d1cbg (3_1_1_4)	2dri-d2dri (3_72_1_1)	1eceb-d1ecea (3_1_1_3)	1fbaa-d1fbaa (3_1_3_1)	1dnpa-d1dnpa2 (3_17_1_1)	7icd-d7icd (3_57_1_1)	1xyza-d1xyza (3_1_1_3)	1rcd-d1rcd (1_24_1_1)	1dik-d1dik_1 (3_1_9_2)
741	1tiig-d1tiid	2_26_2_1	98	6	1	1	0	0	0	0	1ltge-d1ltsd (2_26_2_1)	1bll-d1lam_2 (3_52_3_3)	1gpma-d1gpma2 (3_49_1_1)	2tmda-d2tmda1 (3_1_7_1)	1dora-d1dora (3_1_7_1)	1ytba-d1ytba2 (4_74_1_1)	1febb-d2tpra2 (3_4_1_4)	1prtf-d1prtf (2_26_2_1)	1asu-d1asu (3_41_3_2)
742	1tkaa-d1trka1	3_24_1_2	335	2	0	0	0	1	0	0	4xis-d2xis (3_1_12_1)	1nal1-d1nal1 (3_1_3_1)	1wsyb-d1wsyb (3_59_1_1)	1poxa-d1poxa3 (3_24_1_1)	2dri-d2dri (3_72_1_1)	1fnc-d1fnb_2 (3_14_1_1)	1llo-d1hvq (3_1_1_5)	1art-d1art (3_48_1_1)	1alo-d1alo_6 (4_77_1_1)
743	1tkca-d1trka2	3_24_1_2	197	2	0	0	1	1	0	0	1pyda-d1pvda3 (3_24_1_1)	1poxa-d1poxa2 (3_24_1_1)	1miob-d1miob (3_67_1_1)	1poxa-d1poxa3 (3_24_1_1)	1tkca-d1trka3 (3_34_1_1)	1dora-d1dora (3_1_7_1)	2lbp-d2lbp (3_72_1_1)	2dri-d2dri (3_72_1_1)	1vid-d1vid (3_47_1_1)
744	1tkca-d1trka3	3_34_1_1	146	1	0	0	0	0	0	0	1mpd-d1mpb (3_73_1_1)	1cyda-d1cyda (3_19_1_2)	1tkca-d1trka2 (3_24_1_2)	1dppc-d1dppa (3_73_1_1)	1wsyb-d1wsyb (3_59_1_1)	1jeva-d2olba (3_73_1_1)	1qrda-d1qrda (3_13_4_2)	1art-d1art (3_48_1_1)	1hdca-d1hdca (3_19_1_2)
745	1tle-d1klo_2	7_3_9_2	56	1	0	0	0	0	0	0	2npx-d1nhp_3 (4_46_1_1)	1gtra-d1gtra2 (3_15_1_1)	2prd-d2prd (2_26_5_1)	1ata-d1ata (7_21_1_1)	1edg-d1edg (3_1_1_3)	1amy-d1amy_2 (3_1_1_1)	2trcb-d2trcb (2_46_3_1)	1scea-d1scea (4_55_1_1)	1abra-d1abra (4_94_1_1)
746	1tlfa-d1tlfa	3_72_1_1	296	6	1	1	0	0	0	0	1pnr-d1pnra2 (3_72_1_1)	2dri-d2dri (3_72_1_1)	1gca-d1gca (3_72_1_1)	2lbp-d2lbp (3_72_1_1)	1pea-d1pea (3_72_1_1)	1fbaa-d1fbaa (3_1_3_1)	1miob-d1miob (3_67_1_1)	1ecpa-d1ecpa (3_52_1_1)	1dnpa-d1dnpa2 (3_17_1_1)
747	1tlk-d1tlk	2_1_1_4	103	5	1	1	1	1	0	0	2ncm-d2ncm (2_1_1_4)	1vcaa-d1vcaa2 (2_1_1_4)	1wit-d1wiu (2_1_1_4)	1cdcb-d1cdcb (2_1_1_1)	1eal-d1eal (2_41_1_2)	1tnn-d1tnm (2_1_1_4)	1kiqa-d1vfba (2_1_1_1)	3cd4-d3cd4_1 (2_1_1_1)	1ospo-d1ospo (2_52_1_1)
748	1tme1-d1tme1	2_8_1_4	256	5	1	1	1	1	0	0	2mev1-d2mev1 (2_8_1_4)	2plv-d1pvc1 (2_8_1_4)	2bbvc-d2bbva (2_8_1_3)	5sgae-d1sgc (2_31_1_1)	2phl-d2phla1 (2_58_1_1)	1kiqa-d1vfba (2_1_1_1)	1ospo-d1ospo (2_52_1_1)	1ciy-d1ciy_2 (2_53_2_1)	1eri-d1eria (3_38_1_1)
749	1tml-d1tml	3_2_1_1	286	2	1	1	0	0	0	0	1cb2b-d1cb2a (3_2_1_1)	1xyza-d1xyza (3_1_1_3)	2dri-d2dri (3_72_1_1)	1fbaa-d1fbaa (3_1_3_1)	1edg-d1edg (3_1_1_3)	1miob-d1miob (3_67_1_1)	1dnpa-d1dnpa2 (3_17_1_1)	1cowe-d1bmfd3 (3_25_1_6)	1oya-d1oya (3_1_7_1)
750	1tnda-d1tada1	1_52_1_1	121	1	0	0	0	0	0	0	1ecma-d1ecma (1_93_1_1)	1prcl-d1prcl1 (6_5_1_1)	1hula-d1hula (1_25_1_2)	1phe-d1phb (1_75_1_1)	1chka-d1chka (4_2_1_6)	1vsga-d1vsga (6_3_1_1)	2liga-d2liga (1_23_2_1)	1xsm-d1xsm (1_24_1_2)	1occa-d1occa1 (6_5_1_1)
751	1tnfa-d1tnfa	2_17_1_1	152	3	1	1	0	0	0	0	1cdaa-d1aly (2_17_1_1)	1tnra-d1tnra (2_17_1_1)	1sacb-d1saca (2_19_1_4)	1mpmb-d1mal (6_7_1_2)	2omf-d2omf (6_7_1_1)	2sil-d2sil (2_45_1_1)	1ggt-d1ggta2 (2_1_4_1)	1dlc-d1dlc_1 (2_13_1_2)	1vcaa-d1vcaa1 (2_1_1_3)
752	1tnj-d1bty	2_31_1_2	223	1	0	0	1	1	0	0	1arc-d1arb (2_31_1_1)	5sgae-d1sgc (2_31_1_1)	1p03a-d2alp (2_31_1_1)	4aahc-d4aaha (2_47_1_1)	1bgl-d1bgla1 (2_1_3_1)	1eft-d1eft_2 (2_30_1_1)	2sil-d2sil (2_45_1_1)	2bpa1-d2bpa1 (2_8_1_1)	2cas-d2cas (2_8_1_4)
753	1tnn-d1tnm	2_1_1_4	91	5	1	1	0	1	0	0	1wit-d1wiu (2_1_1_4)	1tlk-d1tlk (2_1_1_4)	1vcaa-d1vcaa2 (2_1_1_4)	1eal-d1eal (2_41_1_2)	1nsdb-d1nsca (2_45_1_1)	1kiqa-d1vfba (2_1_1_1)	1ure-d1ifc (2_41_1_2)	1exg-d1exg (2_2_2_1)	1cdi-d3cd4_2 (2_1_1_3)
754	1tnra-d1tnra	2_17_1_1	144	3	1	1	0	0	0	0	1tnfa-d1tnfa (2_17_1_1)	1cdaa-d1aly (2_17_1_1)	1mpmb-d1mal (6_7_1_2)	1vcaa-d1vcaa1 (2_1_1_3)	2por-d2por (6_7_1_1)	1dlha-d1dlha1 (2_1_1_2)	4kbpb-d4kbpa1 (2_1_9_1)	3nn9-d3nn9 (2_45_1_1)	1prn-d1prn (6_7_1_1)
755	1tnrr-d1ncfa2	7_22_1_1	44	2	0	0	0	0	0	0	2cpo-d1cpo_2 (1_34_2_1)	1prch-d1prch1 (2_27_1_1)	1gph1-d1gph12 (4_88_1_1)	1cbh-d1cbh (7_3_7_1)	1mioa-d1mioa (3_67_1_1)	1nis-d1aco_2 (3_63_1_1)	1kxa-d2snv (2_31_1_3)	2bpa1-d2bpa1 (2_8_1_1)	2blta-d2blta (5_4_1_1)
756	1tns-d1tns	1_6_1_1	76	1	0	0	0	0	0	0	1aara-d1ubi (4_12_2_1)	1iov-d2dln_2 (4_83_1_1)	451c-d351c (1_3_1_1)	1nfp-d1nfp (3_1_13_2)	1dpra-d1dpra2 (1_61_1_1)	1qapa-d1qapa1 (3_1_14_1)	1fjm-d1fjma (4_91_1_2)	1psda-d1psda3 (4_33_15_1)	1rldb-d3rubl2 (4_33_9_1)
757	1toxa-d1ddt_1	2_2_1_1	155	1	0	0	0	0	0	0	1nsdb-d1nsca (2_45_1_1)	2hmb-d1hms (2_41_1_2)	1kit-d1kit_2 (2_19_1_6)	1wit-d1wiu (2_1_1_4)	1kcw-d1kcw_5 (2_5_1_3)	1ure-d1ifc (2_41_1_2)	1bgl-d1bgla2 (2_1_3_1)	3nn9-d3nn9 (2_45_1_1)	1mpmb-d1mal (6_7_1_2)
758	1tpfa-d1tpfa	3_1_11_1	250	1	0	0	0	0	1	1	1fbaa-d1fbaa (3_1_3_1)	1nal1-d1nal1 (3_1_3_1)	1cyda-d1cyda (3_19_1_2)	1miob-d1miob (3_67_1_1)	1oya-d1oya (3_1_7_1)	1qba-d1qba_3 (3_1_1_6)	1gtma-d1gtma1 (3_19_1_7)	1nis-d1aco_2 (3_63_1_1)	1dhpa-d1dhpa (3_1_3_1)
759	1tpn-d1tpm	7_40_1_1	50	3	1	1	0	0	0	0	1fbr-d1fbr_2 (7_40_1_1)	1gof-d1gof_3 (2_46_1_1)	1iob-d1i1b (2_28_1_2)	1bvp1-d1bvp12 (2_14_1_1)	1occb-d1occb1 (2_5_1_2)	2bbkh-d2bbkh (2_46_2_1)	2hmb-d1hms (2_41_1_2)	1bbpa-d1bbpa (2_41_1_1)	1ospo-d1ospo (2_52_1_1)
760	1tsg-d1tsg	4_97_1_3	98	1	0	0	0	0	0	0	1con-d1scs (2_19_1_1)	1gym-d1gym (3_1_15_2)	2pia-d2pia_1 (2_29_1_2)	1hsq-d1hsq (2_21_2_1)	1gof-d1gof_3 (2_46_1_1)	2tmda-d2tmda2 (3_4_1_1)	1art-d1art (3_48_1_1)	2sh1-d1sh1 (7_7_1_1)	1tyc-d2ts1_1 (1_54_1_1)
761	1tsra-d1tupa	2_2_3_1	196	1	0	0	0	0	0	0	1obpa-d1obpa (2_41_1_1)	1bgl-d1bgla1 (2_1_3_1)	1ospo-d1ospo (2_52_1_1)	1qatb-d1djxa2 (2_6_1_1)	2mtal-d2bbkl (7_19_1_1)	2prd-d2prd (2_26_5_1)	1dlc-d1dlc_2 (2_53_2_1)	2bbkh-d2bbkh (2_46_2_1)	1bgl-d1bgla2 (2_1_3_1)
762	1ttaa-d1eta1	2_3_2_1	127	1	0	0	0	0	0	0	1ospo-d1ospo (2_52_1_1)	1plq-d1plq_1 (4_76_1_2)	1prn-d1prn (6_7_1_1)	1p03a-d2alp (2_31_1_1)	1pgs-d1pgs_1 (2_11_1_1)	1udii-d1udii (4_14_4_1)	2hmb-d1hms (2_41_1_2)	1fem-d1hbp (2_41_1_1)	2prd-d2prd (2_26_5_1)
763	1tuia-d1eft_1	2_29_3_1	100	2	1	1	0	0	0	1	1dar-d1dar_1 (2_29_3_1)	5sgae-d1sgc (2_31_1_1)	1efrd-d1bmfd2 (2_33_1_1)	1efr-d1bmfa2 (2_33_1_1)	2pia-d2pia_1 (2_29_1_2)	1asqa-d1aoza2 (2_5_1_3)	1ulo-d1ulo (2_13_1_4)	2act-d2act (4_3_1_1)	1wba-d1wba (2_28_3_1)
764	1tul-d1tul	2_60_4_1	102	1	0	0	0	0	0	0	1cpn-d1cpn (2_19_1_2)	3nn9-d3nn9 (2_45_1_1)	1wit-d1wiu (2_1_1_4)	1iob-d1i1b (2_28_1_2)	1ospo-d1ospo (2_52_1_1)	1ciy-d1ciy_2 (2_53_2_1)	4aahc-d4aaha (2_47_1_1)	1ure-d1ifc (2_41_1_2)	1duta-d1duta (2_60_3_1)

765	1tvs-d1tvs	8_36_1_1	75	1	0	0	0	0	0	0	1fjm-d1fjma (4_91_1_2)	2ada-d1add (3_1_2_1)	1orda-d1orda3 (4_70_1_1)	1hlb-d1hlb (1_1_1_1)	1orda-d1orda2 (3_48_1_4)	3b5c-d3b5c (4_66_1_1)	1ggga-d1ggga (3_73_1_1)	1idm-d1idm (3_57_1_1)	1dik-d1dik_2 (3_5_1_1)
766	1txm-d1txm	7_3_6_2	34	4	0	0	0	0	0	0	1fwp-d1fwp (4_33_18_1)	1smpi-d1smpi (2_42_2_1)	2btfa-d2btfa1 (3_41_1_1)	1tbd-d1tbd (4_48_1_1)	1qora-d1qora2 (3_19_1_1)	1mioa-d1mioa (3_67_1_1)	1dnpa-d1dnpa2 (3_17_1_1)	1han-d1han_1 (4_20_1_3)	1cyx-d1cyx (2_5_1_2)
767	1tyc-d2ts1_1	1_54_1_1	92	1	0	0	0	0	0	0	1csr-d1csh (1_74_1_1)	7cat-d7cata (5_6_1_1)	2abk-d2abk (1_66_1_1)	1zymb-d1zyma (3_5_1_2)	2myd-d1mbd (1_1_1_1)	1hvd-d1hvd (1_51_1_1)	1sly-d1sly_1 (1_84_4_1)	1octc-d1octc2 (1_30_1_1)	1lh7-d1lh1 (1_1_1_1)
768	1tys-d1tys	4_63_1_1	264	1	0	0	0	0	0	0	1leha-d1leha2 (3_54_1_1)	1seta-d1seta2 (4_59_1_1)	1fbaa-d1fbaa (3_1_3_1)	1eri-d1eria (3_38_1_1)	1oiba-d1pbp (3_73_1_1)	1gtma-d1gtma2 (3_54_1_1)	1hpm-d1hpm_1 (3_41_1_1)	1dhr-d1dhr (3_19_1_2)	1lam-d1lam_1 (3_36_1_1)
769	1udh-d1udg	3_11_1_1	228	1	0	0	0	0	0	0	1taha-d1tahb (3_50_1_8)	1daaa-d1daaa (5_14_1_1)	1art-d1art (3_48_1_1)	1emd-d2cmd_1 (3_19_1_5)	3rubs-d3rubs (4_37_1_1)	2bnh-d1dfji (3_7_1_1)	1gca-d1gca (3_72_1_1)	1edt-d1edt (3_1_1_5)	1miob-d1miob (3_67_1_1)
770	1udii-d1udii	4_14_4_1	83	1	0	0	0	0	0	1	1vmoa-d1vmoa (2_53_1_1)	1obpa-d1obpa (2_41_1_1)	1ospo-d1ospo (2_52_1_1)	1con-d1scs (2_19_1_1)	1oaca-d1oaca2 (4_14_2_1)	1ttaa-d1eta1 (2_3_2_1)	1rsy-d1rsy (2_6_1_2)	1hpl-d1hpla1 (2_10_2_1)	1gof-d1gof_3 (2_46_1_1)
771	1ulo-d1ulo	2_13_1_4	152	1	0	0	0	0	0	0	1mpmb-d1mal (6_7_1_2)	1akl-d1kapp1 (2_55_1_1)	1eal-d1eal (2_41_1_2)	4kbpb-d4kbpa1 (2_1_9_1)	1ure-d1ifc (2_41_1_2)	1ospo-d1ospo (2_52_1_1)	1xnb-d1xnb (2_19_1_8)	1p03a-d2alp (2_31_1_1)	1obpa-d1obpa (2_41_1_1)
772	1ure-d1ifc	2_41_1_2	131	3	1	1	0	1	0	0	2hmb-d1hms (2_41_1_2)	1eal-d1eal (2_41_1_2)	1ospo-d1ospo (2_52_1_1)	2por-d2por (6_7_1_1)	2sil-d2sil (2_45_1_1)	1bbpa-d1bbpa (2_41_1_1)	2phl-d2phla1 (2_58_1_1)	1con-d1scs (2_19_1_1)	1prn-d1prn (6_7_1_1)
773	1urna-d1urna	4_33_7_1	96	2	1	1	0	0	0	1	1sxl-d1sxl (4_33_7_1)	1zooa-d1lfaa (3_45_1_1)	2pec-d2pec (2_56_1_1)	2bop-d2bopa (4_33_8_1)	1bll-d1lam_2 (3_52_3_3)	1tkca-d1trka2 (3_24_1_2)	1dora-d1dora (3_1_7_1)	1ecpa-d1ecpa (3_52_1_1)	1dhpa-d1dhpa (3_1_3_1)
774	1vcaa-d1vcaa1	2_1_1_3	109	3	0	0	1	1	1	1	1cdcb-d1cdcb (2_1_1_1)	1bgl-d1bgla1 (2_1_3_1)	1nsdb-d1nsca (2_45_1_1)	3nn9-d3nn9 (2_45_1_1)	1dlha-d1dlha1 (2_1_1_2)	1tnra-d1tnra (2_17_1_1)	1cyx-d1cyx (2_5_1_2)	1bgl-d1bgla2 (2_1_3_1)	1prn-d1prn (6_7_1_1)
775	1vcaa-d1vcaa2	2_1_1_4	90	5	1	1	1	1	0	1	1wit-d1wiu (2_1_1_4)	2ncm-d2ncm (2_1_1_4)	1kiqa-d1vfba (2_1_1_1)	1tlk-d1tlk (2_1_1_4)	1cdi-d3cd4_2 (2_1_1_3)	3cd4-d3cd4_1 (2_1_1_1)	1ospo-d1ospo (2_52_1_1)	2hmb-d1hms (2_41_1_2)	1eal-d1eal (2_41_1_2)
776	1vcc-d1vcc	4_67_1_1	77	1	0	0	0	0	0	0	1mrj-d1mrj (4_94_1_1)	1ltge-d1ltsd (2_26_2_1)	1rpf-d7rsa (4_4_1_1)	1eri-d1eria (3_38_1_1)	4fua-d1fua (3_55_1_1)	2sil-d2sil (2_45_1_1)	1napd-d1napa (4_7_1_1)	1han-d1han_2 (4_20_1_3)	1kit-d1kit_2 (2_19_1_6)
777	1vhh-d1vhh	4_34_1_2	157	1	0	0	1	1	0	0	1lbu-d1lbu_2 (4_34_1_1)	1asza-d1asya2 (4_59_1_1)	1lyla-d1lyla2 (4_59_1_1)	1lba-d1lba (4_64_1_1)	1ris-d1ris (4_33_11_1)	2dkb-d2dkb (3_48_1_3)	1pea-d1pea (3_72_1_1)	1orta-d1orta1 (3_58_1_1)	4kbpa-d4kbpa2 (4_91_1_1)
778	1vhia-d1vhia	4_33_8_1	139	3	0	1	0	0	0	0	1lyla-d1lyla2 (4_59_1_1)	2bop-d2bopa (4_33_8_1)	1pnt-d1phr (3_31_1_1)	1sty-d1sty (2_26_1_1)	1esd-d1esc (3_13_8_1)	1nipb-d1nipa (3_25_1_5)	1tpfa-d1tpfa (3_1_11_1)	1scb-d1csee (3_28_1_1)	1ghr-d1ghr (3_1_1_3)
779	1vhra-d1vhra	3_32_1_1	178	1	0	0	1	1	0	0	2hnp-d2hnp (3_32_1_2)	1tlfa-d1tlfa (3_72_1_1)	1gca-d1gca (3_72_1_1)	1wsyb-d1wsyb (3_59_1_1)	1vin-d1vin_1 (1_59_1_1)	1hsta-d1hsta (1_4_3_7)	2dri-d2dri (3_72_1_1)	1ytn-d1ypta (3_32_1_2)	1itha-d1itha (1_1_1_1)
780	1vid-d1vid	3_47_1_1	213	1	0	0	1	1	0	0	1xva-d1xvaa (3_47_1_2)	2dri-d2dri (3_72_1_1)	1wsyb-d1wsyb (3_59_1_1)	1nsj-d1nsj (3_1_8_1)	1ecpa-d1ecpa (3_52_1_1)	1rnl-d1rnl_2 (3_13_2_1)	4mhta-d1hmy (3_47_1_4)	1tlfa-d1tlfa (3_72_1_1)	2lbp-d2lbp (3_72_1_1)
781	1vii-d1vii	1_14_1_1	36	1	0	0	0	0	0	0	2csn-d1csn (5_1_1_1)	1nipb-d1nipa (3_25_1_5)	1lbd-d1lbd (1_87_1_1)	1ret-d1res (1_4_1_2)	1jlm-d1ido (3_45_1_1)	1vtm-d1vtmp (1_23_5_1)	1noy-d1noya (3_41_3_5)	1idm-d1idm (3_57_1_1)	2hmx-d2hmx (1_58_1_1)
782	1vin-d1vin_1	1_59_1_1	128	4	0	1	1	1	0	0	1vola-d1vola1 (1_59_1_2)	1jkw-d1jkw_1 (1_59_1_1)	2myd-d1mbd (1_1_1_1)	2sas-d2sas (1_34_1_5)	1cowa-d1bmfa1 (1_55_1_1)	1baba-d1baba (1_1_1_1)	3sdha-d3sdha (1_1_1_1)	1zymb-d1zyma (3_5_1_2)	1sly-d1sly_1 (1_84_4_1)
783	1vin-d1vin_2	1_59_1_1	124	4	0	1	0	0	0	0	1lh7-d1lh1 (1_1_1_1)	2wrpr-d2wrpr (1_78_1_1)	1rlr-d1rlr_1 (1_68_1_1)	1tfe-d1tfe (4_24_1_1)	1jkw-d1jkw_1 (1_59_1_1)	1dlc-d1dlc_3 (6_1_3_1)	1hlb-d1hlb (1_1_1_1)	2sas-d2sas (1_34_1_5)	1vin-d1vin_1 (1_59_1_1)
784	1vjs-d1bplb1	2_48_1_1	109	6	1	1	0	0	0	0	2aaa-d2aaa_1 (2_48_1_1)	4aahc-d4aaha (2_47_1_1)	1tcma-d1cdg_3 (2_48_1_1)	1ospo-d1ospo (2_52_1_1)	1ouna-d1ouna (4_14_3_2)	3cd4-d3cd4_1 (2_1_1_1)	1con-d1scs (2_19_1_1)	3ait-d1hoe (2_4_1_1)	1ure-d1ifc (2_41_1_2)
785	1vkla-d3pmga2	3_64_1_1	113	3	0	0	0	0	0	0	1rvva-d1rvva (3_9_1_1)	1seia-d1seia (4_80_1_1)	1pea-d1pea (3_72_1_1)	1gf2-d1igl (7_1_1_1)	1gln-d1gln_2 (3_15_1_1)	1llda-d1llda1 (3_19_1_5)	2bnh-d1dfji (3_7_1_1)	1cof-d1ahq (4_60_1_2)	1plq-d1plq_1 (4_76_1_2)
786	1vmoa-d1vmoa	2_53_1_1	163	1	0	0	0	0	1	1	1dlc-d1dlc_2 (2_53_2_1)	1udii-d1udii (4_14_4_1)	1ospo-d1ospo (2_52_1_1)	2bbkh-d2bbkh (2_46_2_1)	1eur-d1eur (2_45_1_1)	1gof-d1gof_3 (2_46_1_1)	1cpn-d1cpn (2_19_1_2)	1fem-d1hbp (2_41_1_1)	4aahc-d4aaha (2_47_1_1)
787	1vnc-d1vnc	1_80_1_1	576	1	0	0	0	0	0	0	1prcl-d1prcl1 (6_5_1_1)	1faga-d2bmha (1_75_1_1)	1tlfa-d1tlfa (3_72_1_1)	1phe-d1phb (1_75_1_1)	1occa-d1occa1 (6_5_1_1)	1dik-d1dik_3 (4_83_1_4)	1ciy-d1ciy_3 (6_1_3_1)	1hjra-d1hjra (3_41_3_6)	1pgo-d2pgd_1 (1_71_1_1)
788	1vola-d1vola1	1_59_1_2	95	2	0	1	1	1	0	0	1vin-d1vin_1 (1_59_1_1)	1jkw-d1jkw_1 (1_59_1_1)	1tfb-d1vola2 (1_59_1_2)	1gtma-d1gtma1 (3_19_1_7)	1mba-d1mba (1_1_1_1)	1rnl-d1rnl_1 (1_31_1_2)	1abra-d1abra (4_94_1_1)	1phe-d1phb (1_75_1_1)	1osa-d1osa (1_34_1_5)
789	1vpu-d1vpu	7_25_1_1	45	1	0	0	0	0	0	0	1gpc-d1gpc (2_26_4_7)	1fct-d1fct (8_31_1_1)	1mbk-d1msec2 (1_4_1_3)	1tbrr-d1tbrr2 (7_12_1_1)	1noy-d1noya (3_41_3_5)	1hlpa-d1hlpa2 (4_92_1_1)	2tcl-d1hfc (4_50_1_7)	2bbvc-d2bbva (2_8_1_3)	1pmi-d1pmi (2_58_2_1)
790	1vsga-d1vsga	6_3_1_1	362	1	0	0	0	0	0	0	1occa-d1occa1 (6_5_1_1)	1aep-d1aep (1_49_1_1)	2liga-d2liga (1_23_2_1)	1ciy-d1ciy_3 (6_1_3_1)	1dkza-d1dkza (5_17_1_1)	1dlc-d1dlc_3 (6_1_3_1)	1cpcb-d1cpcb (1_1_1_2)	1occc-d1occc1 (6_5_1_1)	1bfra-d1bcfa (1_24_1_1)
791	1vtm-d1vtmp	1_23_5_1	158	1	0	0	0	0	0	0	1csr-d1csh (1_74_1_1)	2sas-d2sas (1_34_1_5)	2blta-d2blta (5_4_1_1)	2scpa-d2scpa (1_34_1_5)	1pbn-d1pbn (3_52_1_1)	1dnpa-d1dnpa1 (1_69_1_1)	1dhr-d1dhr (3_19_1_2)	2bnh-d1dfji (3_7_1_1)	1vnc-d1vnc (1_80_1_1)
792	1wap-d1wapa	2_58_5_1	68	1	0	0	0	0	0	0	1seia-d1seia (4_80_1_1)	1ospo-d1ospo (2_52_1_1)	2bpa1-d2bpa1 (2_8_1_1)	1imba-d2hhma (5_8_1_2)	1hcz-d1ctm_2 (2_59_2_2)	3nn9-d3nn9 (2_45_1_1)	4aahc-d4aaha (2_47_1_1)	1gdob-d1gdoa (4_88_1_1)	1obpa-d1obpa (2_41_1_1)
793	1wba-d1wba	2_28_3_1	171	1	0	0	0	0	0	0	3nn9-d3nn9 (2_45_1_1)	1eur-d1eur (2_45_1_1)	1ospo-d1ospo (2_52_1_1)	1noa-d1noa (2_1_6_1)	1kxa-d2snv (2_31_1_3)	1p03a-d2alp (2_31_1_1)	2bfh-d2fgf (2_28_1_1)	1gof-d1gof_3 (2_46_1_1)	2bbkh-d2bbkh (2_46_2_1)
794	1wdcb-d1scmb	1_34_1_5	138	5	1	1	0	1	0	0	1osa-d1osa (1_34_1_5)	1tcob-d1tcob (1_34_1_5)	2scpa-d2scpa (1_34_1_5)	2sas-d2sas (1_34_1_5)	1hvd-d1hvd (1_51_1_1)	2abk-d2abk (1_66_1_1)	1rro-d1rro (1_34_1_4)	1sra-d1sra (1_34_1_3)	1jkw-d1jkw_1 (1_59_1_1)
795	1wfba-d1wfba	8_20_1_1	37	1	0	0	0	0	0	0	1sesa-d1seta1 (1_2_3_1)	1vsga-d1vsga (6_3_1_1)	2hmgd-d1htmb (6_2_1_1)	1fosf-d1fosf (1_97_2_1)	1dkza-d1dkza (5_17_1_1)	1occb-d1occb2 (6_5_1_1)	1occj-d1occj1 (6_5_1_1)	1lpe-d1lpe (1_23_1_1)	1fose-d1fose (1_97_2_1)
796	1wgcb-d1wgta4	7_3_1_1	42	3	1	1	0	0	0	0	1wgta-d1wgta3 (7_3_1_1)	1wgta-d1wgta2 (7_3_1_1)	1ptq-d1ptq (7_38_1_1)	1prch-d1prch1 (2_27_1_1)	1rie-d1rie (7_33_1_2)	1ata-d1ata (7_21_1_1)	119l-d119l (4_2_1_3)	1ttaa-d1eta1 (2_3_2_1)	1tbqr-d1tbrr1 (7_12_1_1)
797	1wgta-d1wgta2	7_3_1_1	34	3	1	1	0	0	0	0	1wgta-d1wgta3 (7_3_1_1)	1wgcb-d1wgta4 (7_3_1_1)	1rie-d1rie (7_33_1_2)	1tbrr-d1tbrr2 (7_12_1_1)	119l-d119l (4_2_1_3)	1ttaa-d1eta1 (2_3_2_1)	1prch-d1prch1 (2_27_1_1)	1ospo-d1ospo (2_52_1_1)	1apa-d1apa (4_94_1_1)
798	1wgta-d1wgta3	7_3_1_1	43	3	1	1	0	0	0	0	1wgcb-d1wgta4 (7_3_1_1)	1wgta-d1wgta2 (7_3_1_1)	1vmoa-d1vmoa (2_53_1_1)	1rie-d1rie (7_33_1_2)	1apa-d1apa (4_94_1_1)	1tuia-d1eft_1 (2_29_3_1)	1ttaa-d1eta1 (2_3_2_1)	1ptq-d1ptq (7_38_1_1)	1prch-d1prch1 (2_27_1_1)
799	1whi-d1whi	2_25_1_1	122	1	0	0	0	0	0	0	1noa-d1noa (2_1_6_1)	2pcd-d2pcda (2_3_3_1)	1p03a-d2alp (2_31_1_1)	1fnc-d1fnb_1 (2_29_1_1)	1msad-d1msaa (2_54_1_1)	1qila-d1tssa1 (2_26_2_2)	1dar-d1dar_1 (2_29_3_1)	1clc-d1clc_2 (2_1_1_5)	1fem-d1hbp (2_41_1_1)
800	1wit-d1wiu	2_1_1_4	93	5	1	1	1	1	0	0	1vcaa-d1vcaa2 (2_1_1_4)	1tnn-d1tnm (2_1_1_4)	2ncm-d2ncm (2_1_1_4)	1tlk-d1tlk (2_1_1_4)	1kiqa-d1vfba (2_1_1_1)	1cid-d1cid_1 (2_1_1_1)	3cd4-d3cd4_1 (2_1_1_1)	1eal-d1eal (2_41_1_2)	1ospo-d1ospo (2_52_1_1)
801	1wsyb-d1wsyb	3_59_1_1	385	1	0	0	0	0	0	0	2dri-d2dri (3_72_1_1)	1art-d1art (3_48_1_1)	1tlfa-d1tlfa (3_72_1_1)	1nai-d1xel (3_19_1_2)	1pdo-d1pdo (3_40_1_1)	2chr-d2chr_1 (3_1_6_2)	1qapa-d1qapa1 (3_1_14_1)	2dkb-d2dkb (3_48_1_3)	1dora-d1dora (3_1_7_1)
802	1xnb-d1xnb	2_19_1_8	185	1	0	0	0	0	0	0	1ospo-d1ospo (2_52_1_1)	2omf-d2omf (6_7_1_1)	2sil-d2sil (2_45_1_1)	3nn9-d3nn9 (2_45_1_1)	1pgs-d1pgs_1 (2_11_1_1)	1cpn-d1cpn (2_19_1_2)	1kit-d1kit_2 (2_19_1_6)	1occb-d1occb1 (2_5_1_2)	1svb-d1svb_2 (6_10_1_1)
803	1xoa-d2trxa	3_33_1_1	108	4	0	1	1	1	0	0	1mek-d1mek (3_33_1_2)	1kte-d1kte (3_33_1_1)	1chma-d1chma2 (4_72_1_1)	1oela-d1grl_2 (3_5_3_1)	1cnf-d2cnd_2 (3_14_1_1)	1fead-d2tpra3 (4_46_1_1)	1pyda-d1pvda3 (3_24_1_1)	1poxa-d1poxa1 (3_21_1_1)	1tys-d1tys (4_63_1_1)
804	1xra-d1mxa_2	4_75_1_1	124	3	0	0	0	0	0	0	1chma-d1chma2 (4_72_1_1)	1bgl-d1bgla2 (2_1_3_1)	1ctt-d1ctt_1 (3_75_1_1)	1eny-d1eny (3_19_1_2)	1dik-d1dik_3 (4_83_1_4)	1ris-d1ris (4_33_11_1)	1rpl-d1rpl (5_10_1_1)	1phk-d1phk (5_1_1_1)	1tdta-d1tdta (2_57_1_2)
805	1xsm-d1xsm	1_24_1_2	288	5	1	1	0	0	0	0	1mrrb-d1riba (1_24_1_2)	1mtyd-d1mmod (1_24_1_2)	1occc-d1occc1 (6_5_1_1)	1occa-d1occa1 (6_5_1_1)	1mmob-d1mmob (1_24_1_2)	1ciy-d1ciy_3 (6_1_3_1)	1afra-d1afra (1_24_1_2)	1fps-d1fps (1_91_1_1)	1dlc-d1dlc_3 (6_1_3_1)
806	1xsoa-d1xsoa	2_1_7_1	150	1	0	0	0	0	0	0	2sil-d2sil (2_45_1_1)	1exg-d1exg (2_2_2_1)	1xnb-d1xnb (2_19_1_8)	1cdaa-d1aly (2_17_1_1)	1kxa-d2snv (2_31_1_3)	4kbpa-d4kbpa2 (4_91_1_1)	1tme1-d1tme1 (2_8_1_4)	2bbkh-d2bbkh (2_46_2_1)	1fna-d1fna (2_1_2_1)
807	1xula-d1hcna	7_14_1_4	85	2	1	1	0	0	0	0	1xulb-d1hcnb (7_14_1_4)	1hgea-d1hgea (2_14_1_2)	1daaa-d1daaa (5_14_1_1)	1ten-d1ten (2_1_2_1)	1pbn-d1pbn (3_52_1_1)	1kfd-d1kfd_1 (3_41_3_4)	1ytn-d1ypta (3_32_1_2)	1kob-d1koba (5_1_1_1)	1eft-d1eft_2 (2_30_1_1)
808	1xulb-d1hcnb	7_14_1_4	110	2	0	0	0	0	0	0	1asqa-d1aoza2 (2_5_1_3)	1svb-d1svb_2 (6_10_1_1)	2prd-d2prd (2_26_5_1)	1wba-d1wba (2_28_3_1)	1cpn-d1cpn (2_19_1_2)	1exg-d1exg (2_2_2_1)	2avia-d2avia (2_42_1_1)	1xula-d1hcna (7_14_1_4)	1gof-d1gof_2 (2_13_1_1)
809	1xva-d1xvaa	3_47_1_2	292	1	0	0	1	1	0	0	1vid-d1vid (3_47_1_1)	4mhta-d1hmy (3_47_1_4)	1pgn-d2pgd_2 (3_19_1_6)	1dcta-d1dcta (3_47_1_4)	2anhb-d2anha (3_56_1_1)	1chd-d1chd (3_27_1_1)	2lbp-d2lbp (3_72_1_1)	1scb-d1csee (3_28_1_1)	1glm-d1glm (1_73_1_1)
810	1xxca-d1xxaa	4_38_2_1	71	1	0	0	0	0	0	0	1psda-d1psda3 (4_33_15_1)	1iba-d1iba (4_53_1_1)	2dkb-d2dkb (3_48_1_3)	1fwp-d1fwp (4_33_18_1)	2bnh-d1dfji (3_7_1_1)	6ldh-d1ldm_1 (3_19_1_5)	1bncb-d1bnca2 (3_20_1_1)	1orda-d1orda2 (3_48_1_4)	1broa-d1broa (3_50_1_5)
811	1xyza-d1xyza	3_1_1_3	320	5	0	1	0	1	1	1	1lucb-d1lucb (3_1_13_1)	1tml-d1tml (3_2_1_1)	1cbg-d1cbg (3_1_1_4)	1ghr-d1ghr (3_1_1_3)	1nai-d1xel (3_19_1_2)	1nfp-d1nfp (3_1_13_2)	4xis-d2xis (3_1_12_1)	1fbaa-d1fbaa (3_1_3_1)	1tlfa-d1tlfa (3_72_1_1)
812	1xzl-d1cus	3_13_7_1	197	1	0	0	0	0	0	0	1lbt-d1tca (3_50_1_7)	1broa-d1broa (3_50_1_5)	1tib-d1tib (3_50_1_7)	3tgl-d3tgl (3_50_1_7)	1wsyb-d1wsyb (3_59_1_1)	1nis-d1aco_2 (3_63_1_1)	1tlfa-d1tlfa (3_72_1_1)	2dhd-d1ede (3_50_1_3)	1fnc-d1fnb_2 (3_14_1_1)

813	1yasa-d1yasa	3_50_1_10	256	1	0	0	1	1	0	0	2dhd-d1ede (3_50_1_3)	1broa-d1broa (3_50_1_5)	1lbt-d1tca (3_50_1_7)	1taha-d1tahb (3_50_1_8)	1nal1-d1nal1 (3_1_3_1)	1llo-d1hvq (3_1_1_5)	1wsyb-d1wsyb (3_59_1_1)	1fcdb-d1fcda2 (3_4_1_4)	1art-d1art (3_48_1_1)
814	1yhb-d1yhb	2_26_4_7	87	3	0	1	0	0	0	0	1fem-d1hbp (2_41_1_1)	1pfsa-d1pfsa (2_26_4_7)	1mrj-d1mrj (4_94_1_1)	2bbkh-d2bbkh (2_46_2_1)	2avia-d2avia (2_42_1_1)	2trcb-d2trcb (2_46_3_1)	2pcd-d2pcda (2_3_3_1)	1wba-d1wba (2_28_3_1)	1eft-d1eft_2 (2_30_1_1)
815	1yrna-d1yrna	1_4_1_1	49	5	1	1	0	0	0	0	1octc-d1octc1 (1_4_1_1)	1hddc-d1enh (1_4_1_1)	1prhb-d1prha1 (1_65_1_2)	1osa-d1osa (1_34_1_5)	1occe-d1occe (1_84_7_1)	1rnl-d1rnl_1 (1_31_1_2)	1alo-d1alo_1 (1_47_1_1)	1ecma-d1ecma (1_93_1_1)	1sly-d1sly_1 (1_84_4_1)
816	1yrnb-d1yrnb	1_4_1_1	78	5	1	1	0	0	0	0	1octc-d1octc1 (1_4_1_1)	1hddc-d1enh (1_4_1_1)	1llib-d1llia (1_30_1_2)	1gtma-d1gtma1 (3_19_1_7)	1fbaa-d1fbaa (3_1_3_1)	1oxa-d1oxa (1_75_1_1)	1mba-d1mba (1_1_1_1)	1dlc-d1dlc_3 (6_1_3_1)	1yrna-d1yrna (1_4_1_1)
817	1ysac-d2ztaa	1_97_2_1	31	4	0	0	0	0	0	0	1mmob-d1mmob (1_24_1_2)	1bfma-d1bfma (1_21_1_2)	1vsga-d1vsga (6_3_1_1)	1occc-d1occc1 (6_5_1_1)	1buca-d1buca1 (1_23_6_1)	1lki-d1lki (1_25_1_1)	1sala-d1olga (1_44_1_1)	1occj-d1occj1 (6_5_1_1)	1imba-d2hhma (5_8_1_2)
818	1ytba-d1ytba2	4_74_1_1	85	2	1	1	0	0	0	0	1ytfa-d1ytba1 (4_74_1_1)	1tiig-d1tiid (2_26_2_1)	2pola-d2pola3 (4_76_1_1)	1ctt-d1ctt_2 (3_75_1_1)	2bbkh-d2bbkh (2_46_2_1)	1ltge-d1ltsd (2_26_2_1)	1napd-d1napa (4_7_1_1)	2bfh-d2fgf (2_28_1_1)	1tys-d1tys (4_63_1_1)
819	1ytfa-d1ytba1	4_74_1_1	95	2	1	1	0	0	0	0	1ytba-d1ytba2 (4_74_1_1)	1pls-d1pls (2_37_1_1)	1ltge-d1ltsd (2_26_2_1)	1hrda-d1hrda2 (3_54_1_1)	1tiig-d1tiid (2_26_2_1)	1phk-d1phk (5_1_1_1)	1hpm-d1hpm_1 (3_41_1_1)	2hnp-d2hnp (3_32_1_2)	1tys-d1tys (4_63_1_1)
820	1ytfb-d1ytfb1	1_28_1_1	46	2	0	0	0	0	0	0	1gtma-d1gtma1 (3_19_1_7)	2myd-d1mbd (1_1_1_1)	1dlc-d1dlc_3 (6_1_3_1)	1cola-d1cola (6_1_1_1)	1ryt-d1ryt_1 (1_24_1_1)	1buca-d1buca1 (1_23_6_1)	2wrpr-d2wrpr (1_78_1_1)	1fipa-d1fipa (1_76_1_1)	2hmgd-d1htmb (6_2_1_1)
821	1ytfc-d1ytfc1	2_38_1_1	46	1	0	0	0	0	0	0	2hmb-d1hms (2_41_1_2)	1sacb-d1saca (2_19_1_4)	1mkaa-d1mkaa (4_21_1_1)	1juy-d1adea (3_25_1_5)	1eal-d1eal (2_41_1_2)	1vcaa-d1vcaa1 (2_1_1_3)	1exg-d1exg (2_2_2_1)	3nn9-d3nn9 (2_45_1_1)	1toxa-d1ddt_1 (2_2_1_1)
822	1ytfd-d1ytfd1	1_28_1_1	50	2	0	0	0	0	0	0	1ciy-d1ciy_3 (6_1_3_1)	1occc-d1occc1 (6_5_1_1)	1zymb-d1zyma (3_5_1_2)	2myd-d1mbd (1_1_1_1)	2hbg-d1hbg (1_1_1_1)	1idm-d1idm (3_57_1_1)	1leha-d1leha1 (3_19_1_7)	1vnc-d1vnc (1_80_1_1)	2cpo-d1cpo_1 (1_34_2_1)
823	1ytn-d1ypta	3_32_1_2	278	2	1	1	0	0	0	0	2hnp-d2hnp (3_32_1_2)	1buca-d1buca1 (1_23_6_1)	1sly-d1sly_1 (1_84_4_1)	1csr-d1csh (1_74_1_1)	1occa-d1occa1 (6_5_1_1)	1xsm-d1xsm (1_24_1_2)	1gbs-d1gbs (4_2_1_4)	1afra-d1afra (1_24_1_2)	1mmob-d1mmob (1_24_1_2)
824	1yua-d1yua_1	4_67_1_2	65	2	0	0	0	0	0	0	1aey-d1shg (2_21_2_1)	1wba-d1wba (2_28_3_1)	1pgga-d1prha2 (7_3_9_1)	1iob-d1i1b (2_28_1_2)	1kxa-d2snv (2_31_1_3)	1duta-d1duta (2_60_3_1)	1sema-d1sema (2_21_2_1)	1oya-d1oya (3_1_7_1)	1poba-d1poa (1_95_1_2)
825	1yua-d1yua_2	4_67_1_2	57	2	0	0	0	0	0	0	2hmb-d1hms (2_41_1_2)	1ure-d1ifc (2_41_1_2)	1mtyd-d1mmod (1_24_1_2)	1ospo-d1ospo (2_52_1_1)	1apme-d1apme (5_1_1_1)	1udii-d1udii (4_14_4_1)	1stfi-d1stfi (4_14_1_2)	1pfsa-d1pfsa (2_26_4_7)	2sil-d2sil (2_45_1_1)
826	1zaac-d1zaac1	7_28_1_1	29	10	1	1	0	0	0	0	1aay-d1zaac2 (7_28_1_1)	1ard-d1ard (7_28_1_1)	1sbp-d1sbp (3_73_1_1)	1dxy-d1dxy_2 (3_19_1_4)	1tdta-d1tdta (2_57_1_2)	2dri-d2dri (3_72_1_1)	1kfd-d1kfd_1 (3_41_3_4)	1zfd-d1zfd (7_28_1_1)	1pax-d1pax_2 (4_95_1_2)
827	1zfd-d1zfd	7_28_1_1	32	10	0	0	0	0	0	0	1dyna-d1dyna (2_37_1_1)	1tpfa-d1tpfa (3_1_11_1)	1sbp-d1sbp (3_73_1_1)	2masa-d2masa (3_51_1_1)	2hmx-d2hmx (1_58_1_1)	1dik-d1dik_1 (3_1_9_2)	2lbp-d2lbp (3_72_1_1)	1ytn-d1ypta (3_32_1_2)	2dhd-d1ede (3_50_1_3)
828	1znf-d1znf	7_28_1_1	24	10	1	1	0	0	0	0	1aay-d1zaac2 (7_28_1_1)	1aay-d1zaac3 (7_28_1_1)	1ard-d1ard (7_28_1_1)	2anhb-d2anha (3_56_1_1)	1paa-d1paa (7_28_1_1)	1igs-d1igs (3_1_8_1)	1cby-d1cby (4_58_1_1)	1tns-d1tns (1_6_1_1)	1sgk-d1ddt_2 (4_95_1_1)
829	1zooa-d1lfaa	3_45_1_1	183	2	1	1	0	0	0	0	1jlm-d1ido (3_45_1_1)	1lucb-d1lucb (3_1_13_1)	1hmp-d1hmpa (3_44_1_1)	1wsyb-d1wsyb (3_59_1_1)	2dri-d2dri (3_72_1_1)	1ris-d1ris (4_33_11_1)	1otga-d1otga (4_40_1_2)	1lyla-d1lyla2 (4_59_1_1)	1idm-d1idm (3_57_1_1)
830	1zto-d1zto	8_11_1_1	36	1	0	0	0	0	0	0	1cowe-d1bmfd3 (3_25_1_6)	2ccya-d2ccya (1_23_3_2)	2gpb-d1gpb (3_68_1_2)	1rfba-d1rfba (1_25_1_3)	1edt-d1edt (3_1_1_5)	1hora-d1deaa (3_23_1_1)	2myd-d1mbd (1_1_1_1)	1llo-d1hvq (3_1_1_5)	1fps-d1fps (1_91_1_1)
831	1zymb-d1zyma	3_5_1_2	247	1	0	0	0	0	0	0	1mtyd-d1mmod (1_24_1_2)	1occa-d1occa1 (6_5_1_1)	1vsga-d1vsga (6_3_1_1)	1prcl-d1prcl1 (6_5_1_1)	1ciy-d1ciy_3 (6_1_3_1)	1mrrb-d1riba (1_24_1_2)	1occc-d1occc1 (6_5_1_1)	1afra-d1afra (1_24_1_2)	2liga-d2liga (1_23_2_1)
832	2aaa-d2aaa_1	2_48_1_1	103	6	1	1	0	0	0	0	1tcma-d1cdg_3 (2_48_1_1)	2bbkh-d2bbkh (2_46_2_1)	1vjs-d1bplb1 (2_48_1_1)	1qila-d1tssa1 (2_26_2_2)	1ospo-d1ospo (2_52_1_1)	1gof-d1gof_3 (2_46_1_1)	2hmb-d1hms (2_41_1_2)	1nsdb-d1nsca (2_45_1_1)	3nn9-d3nn9 (2_45_1_1)
833	2aaa-d2aaa_2	3_1_1_1	353	4	1	1	0	1	1	1	1cxh-d1cdg_4 (3_1_1_1)	1amy-d1amy_2 (3_1_1_1)	1ppi-d1ppi_2 (3_1_1_1)	1oya-d1oya (3_1_7_1)	1dik-d1dik_1 (3_1_9_2)	1qba-d1qba_3 (3_1_1_6)	1wsyb-d1wsyb (3_59_1_1)	1bgl-d1bgla5 (3_1_1_3)	1taha-d1tahb (3_50_1_8)
834	2abk-d2abk	1_66_1_1	211	1	0	0	0	0	0	0	1csr-d1csh (1_74_1_1)	1hvd-d1hvd (1_51_1_1)	2sas-d2sas (1_34_1_5)	1wdcb-d1scmb (1_34_1_5)	1osa-d1osa (1_34_1_5)	1tcob-d1tcob (1_34_1_5)	1rlr-d1rlr_1 (1_68_1_1)	1lis-d1lis (1_17_1_1)	1fps-d1fps (1_91_1_1)
835	2act-d2act	4_3_1_1	218	2	1	1	0	0	0	0	1gcb-d1gcb (4_3_1_1)	1p03a-d2alp (2_31_1_1)	1miob-d1miob (3_67_1_1)	1rvva-d1rvva (3_9_1_1)	1fnc-d1fnb_1 (2_29_1_1)	1bmfc-d1bmfa3 (3_25_1_6)	2aky-d1aky (3_25_1_1)	1taha-d1tahb (3_50_1_8)	1hvd-d1hvd (1_51_1_1)
836	2ada-d1add	3_1_2_1	349	1	0	0	0	0	0	1	1gln-d1gln_2 (3_15_1_1)	4xis-d2xis (3_1_12_1)	1dhpa-d1dhpa (3_1_3_1)	1miob-d1miob (3_67_1_1)	1nai-d1xel (3_19_1_2)	2dri-d2dri (3_72_1_1)	1eceb-d1ecea (3_1_1_3)	1gtra-d1gtra2 (3_15_1_1)	1nal1-d1nal1 (3_1_3_1)
837	2aky-d1aky	3_25_1_1	218	2	0	0	0	0	0	0	1miob-d1miob (3_67_1_1)	1art-d1art (3_48_1_1)	1jud-d1jud (5_18_1_1)	1csr-d1csh (1_74_1_1)	1cpcb-d1cpcb (1_1_1_2)	1leha-d1leha1 (3_19_1_7)	1tlfa-d1tlfa (3_72_1_1)	1fbaa-d1fbaa (3_1_3_1)	2dri-d2dri (3_72_1_1)
838	2amg-d1jdc_1	2_48_1_1	61	6	0	0	0	0	0	0	2bbkh-d2bbkh (2_46_2_1)	1tbd-d1tbd (4_48_1_1)	1bebb-d1beba (2_41_1_1)	1tnn-d1tnm (2_1_1_4)	1lcl-d1lcl (2_19_1_3)	1apa-d1apa (4_94_1_1)	1udii-d1udii (4_14_4_1)	1tme1-d1tme1 (2_8_1_4)	1dlc-d1dlc_2 (2_53_2_1)
839	2anhb-d2anha	3_56_1_1	446	1	0	0	0	0	0	0	1art-d1art (3_48_1_1)	1ryt-d1ryt_1 (1_24_1_1)	1xva-d1xvaa (3_47_1_2)	1lucb-d1lucb (3_1_13_1)	1eac-d1eaf (3_30_1_1)	1vhra-d1vhra (3_32_1_1)	1occc-d1occc1 (6_5_1_1)	1asza-d1asya2 (4_59_1_1)	1bfra-d1bcfa (1_24_1_1)
840	2avia-d2avia	2_42_1_1	121	1	0	0	0	0	0	0	1ospo-d1ospo (2_52_1_1)	1fem-d1hbp (2_41_1_1)	1eur-d1eur (2_45_1_1)	2pcd-d2pcda (2_3_3_1)	1obpa-d1obpa (2_41_1_1)	1mpmb-d1mal (6_7_1_2)	1gof-d1gof_3 (2_46_1_1)	1pgs-d1pgs_1 (2_11_1_1)	2phl-d2phla1 (2_58_1_1)
841	2baa-d2baa	4_2_1_1	243	1	0	0	0	1	0	0	1jkw-d1jkw_1 (1_59_1_1)	1juy-d1adea (3_25_1_5)	1scha-d1scha (1_65_1_1)	1occe-d1occe (1_84_7_1)	1sly-d1sly_2 (4_2_1_5)	1phe-d1phb (1_75_1_1)	1gdz-d1ak4c (1_57_1_1)	1wsyb-d1wsyb (3_59_1_1)	1mba-d1mba (1_1_1_1)
842	2bbkh-d2bbkh	2_46_2_1	355	1	0	0	0	0	1	1	2trcb-d2trcb (2_46_3_1)	1gof-d1gof_3 (2_46_1_1)	1eur-d1eur (2_45_1_1)	4aahc-d4aaha (2_47_1_1)	1nsdb-d1nsca (2_45_1_1)	1ospo-d1ospo (2_52_1_1)	1pex-d1pex (2_44_1_1)	1sacb-d1saca (2_19_1_4)	2sil-d2sil (2_45_1_1)
843	2bbvc-d2bbva	2_8_1_3	308	1	0	0	1	1	0	0	1smvc-d1smva (2_8_1_2)	2plv-d1pvc1 (2_8_1_4)	2cas-d2cas (2_8_1_4)	1tsra-d1tupa (2_2_3_1)	1hgea-d1hgea (2_14_1_2)	1dlc-d1dlc_2 (2_53_2_1)	1rsy-d1rsy (2_6_1_2)	1tme1-d1tme1 (2_8_1_4)	1asqa-d1aoza2 (2_5_1_3)
844	2bfh-d2fgf	2_28_1_1	126	1	0	0	0	0	0	0	2bbkh-d2bbkh (2_46_2_1)	1ospo-d1ospo (2_52_1_1)	1eur-d1eur (2_45_1_1)	1gof-d1gof_3 (2_46_1_1)	2pia-d2pia_1 (2_29_1_2)	1iob-d1i1b (2_28_1_2)	2trcb-d2trcb (2_46_3_1)	1abrb-d1abrb2 (2_28_2_1)	1msad-d1msaa (2_54_1_1)
845	2blta-d2blta	5_4_1_1	359	3	1	1	0	0	0	0	3pte-d3pte (5_4_1_1)	1btl-d1btl (5_4_1_1)	1ptf-d1ptf (4_52_1_1)	1juy-d1adea (3_25_1_5)	1pda-d1pda_2 (4_28_2_1)	1dik-d1dik_3 (4_83_1_4)	1dkza-d1dkza (5_17_1_1)	1leha-d1leha2 (3_54_1_1)	1wdcb-d1scmb (1_34_1_5)
846	2bnh-d1dfji	3_7_1_1	456	1	0	0	0	0	0	0	1miob-d1miob (3_67_1_1)	2chr-d2chr_1 (3_1_6_2)	1fbaa-d1fbaa (3_1_3_1)	1mioa-d1mioa (3_67_1_1)	2lbp-d2lbp (3_72_1_1)	2dri-d2dri (3_72_1_1)	1gtra-d1gtra2 (3_15_1_1)	1pea-d1pea (3_72_1_1)	1gca-d1gca (3_72_1_1)
847	2bop-d2bopa	4_33_8_1	85	3	1	1	0	0	0	1	1dhma-d1dhma (4_33_8_1)	1nfp-d1nfp (3_1_13_2)	1vhia-d1vhia (4_33_8_1)	1lucb-d1lucb (3_1_13_1)	1urna-d1urna (4_33_7_1)	2gd1o-d1gd1o2 (4_41_1_1)	1tlfa-d1tlfa (3_72_1_1)	1dppc-d1dppa (3_73_1_1)	1gpma-d1gpma3 (4_30_1_1)
848	2bpa1-d2bpa1	2_8_1_1	426	1	0	0	0	0	0	0	2por-d2por (6_7_1_1)	1tnra-d1tnra (2_17_1_1)	1bgl-d1bgla2 (2_1_3_1)	2phl-d2phla1 (2_58_1_1)	1ggt-d1ggta2 (2_1_4_1)	2gpb-d1gpb (3_68_1_2)	1ouna-d1ouna (4_14_3_2)	1chma-d1chma2 (4_72_1_1)	1lxta-d3pmga4 (4_74_2_1)
849	2btfa-d2btfa1	3_41_1_1	144	4	1	1	0	0	0	0	1ngi-d1hpm_2 (3_41_1_1)	1gln-d1gln_1 (1_67_1_1)	1pgo-d2pgd_1 (1_71_1_1)	2sas-d2sas (1_34_1_5)	1jeva-d2olba (3_73_1_1)	1miob-d1miob (3_67_1_1)	1vid-d1vid (3_47_1_1)	1oya-d1oya (3_1_7_1)	1poxa-d1poxa3 (3_24_1_1)
850	2btf-d2btfa2	3_41_1_1	229	4	0	0	0	0	0	0	1bmta-d1bmta2 (3_13_5_1)	1miob-d1miob (3_67_1_1)	1tlfa-d1tlfa (3_72_1_1)	1pea-d1pea (3_72_1_1)	1cowe-d1bmfd3 (3_25_1_6)	1cbg-d1cbg (3_1_1_4)	1fbaa-d1fbaa (3_1_3_1)	1edt-d1edt (3_1_1_5)	1dnpa-d1dnpa1 (1_69_1_1)
851	2cas-d2cas	2_8_1_4	548	5	0	1	0	1	0	0	1arc-d1arb (2_31_1_1)	2sil-d2sil (2_45_1_1)	1sva5-d1sva1 (2_8_1_4)	2por-d2por (6_7_1_1)	1dlc-d1dlc_2 (2_53_2_1)	2bbvc-d2bbva (2_8_1_3)	1nsdb-d1nsca (2_45_1_1)	2gpb-d1gpb (3_68_1_2)	1sacb-d1saca (2_19_1_4)
852	2cbp-d2cbp	2_5_1_1	96	4	0	1	1	1	0	0	1aspb-d1aoza1 (2_5_1_3)	1cur-d1cur (2_5_1_1)	2pcy-d1plc (2_5_1_1)	1kcw-d1kcw_4 (2_5_1_3)	3nn9-d3nn9 (2_45_1_1)	1vcaa-d1vcaa2 (2_1_1_4)	1kcw-d1kcw_2 (2_5_1_3)	1wit-d1wiu (2_1_1_4)	2ncm-d2ncm (2_1_1_4)
853	2ccya-d2ccya	1_23_3_2	127	3	1	1	0	0	0	0	1bbha-d1bbha (1_23_3_2)	1rcpa-d1cpq (1_23_3_2)	1ciy-d1ciy_3 (6_1_3_1)	1afra-d1afra (1_24_1_2)	1occc-d1occc1 (6_5_1_1)	1cpca-d1cpca (1_1_1_2)	2myd-d1mbd (1_1_1_1)	2lhb-d2lhb (1_1_1_1)	1mba-d1mba (1_1_1_1)
854	2chr-d2chr_1	3_1_6_2	244	2	1	1	1	1	0	1	2mnr-d2mnr_1 (3_1_6_2)	1onea-d1ebha1 (3_1_6_1)	2bnh-d1dfji (3_7_1_1)	1nal1-d1nal1 (3_1_3_1)	1dora-d1dora (3_1_7_1)	1gca-d1gca (3_72_1_1)	2dri-d2dri (3_72_1_1)	1hrda-d1hrda1 (3_19_1_7)	1tlfa-d1tlfa (3_72_1_1)
855	2chtb-d2chsa	4_39_1_1	114	1	0	0	0	0	0	0	1otfa-d1otfa (4_40_1_1)	1hora-d1deaa (3_23_1_1)	1eac-d1eaf (3_30_1_1)	2aaa-d2aaa_2 (3_1_1_1)	7icd-d7icd (3_57_1_1)	1edg-d1edg (3_1_1_3)	1fbaa-d1fbaa (3_1_3_1)	1gym-d1gym (3_1_15_2)	4xis-d2xis (3_1_12_1)
856	2cpl-d2cpl	2_43_1_1	164	1	0	0	0	0	0	0	1chma-d1chma2 (4_72_1_1)	1tys-d1tys (4_63_1_1)	1bcpb-d1prtb1 (2_26_2_1)	1eac-d1eaf (3_30_1_1)	2por-d2por (6_7_1_1)	1xxca-d1xxaa (4_38_2_1)	2qila-d1tssa2 (4_12_5_1)	1lyla-d1lyla2 (4_59_1_1)	1dik-d1dik_3 (4_83_1_4)
857	2cpo-d1cpo_1	1_34_2_1	119	2	0	0	0	0	0	0	1cxh-d1cdg_4 (3_1_1_1)	2myd-d1mbd (1_1_1_1)	1llib-d1llia (1_30_1_2)	1hddc-d1enh (1_4_1_1)	1dnpa-d1dnpa1 (1_69_1_1)	1noy-d1noya (3_41_3_5)	1dik-d1dik_3 (4_83_1_4)	1gtra-d1gtra2 (3_15_1_1)	1lucb-d1lucb (3_1_13_1)
858	2cpo-d1cpo_2	1_34_2_1	179	2	0	0	0	0	0	1	2abk-d2abk (1_66_1_1)	2sas-d2sas (1_34_1_5)	1miob-d1miob (3_67_1_1)	1cola-d1cola (6_1_1_1)	1art-d1art (3_48_1_1)	1sbp-d1sbp (3_73_1_1)	1lfb-d1lfb (1_4_1_1)	1vin-d1vin_1 (1_59_1_1)	1hvd-d1hvd (1_51_1_1)
859	2csn-d1csn	5_1_1_1	293	4	1	1	0	0	0	0	1kob-d1koba (5_1_1_1)	1phk-d1phk (5_1_1_1)	1apme-d1apme (5_1_1_1)	1csr-d1csh (1_74_1_1)	1lucb-d1lucb (3_1_13_1)	1akl-d1kapp2 (4_50_1_6)	3sdha-d3sdha (1_1_1_1)	1cbg-d1cbg (3_1_1_4)	1gbs-d1gbs (4_2_1_4)
860	2ctb-d2ctb	3_52_3_1	307	1	0	0	1	1	0	0	1obr-d1obr (3_52_3_2)	1idm-d1idm (3_57_1_1)	1dnpa-d1dnpa2 (3_17_1_1)	1rvva-d1rvva (3_9_1_1)	1asu-d1asu (3_41_3_2)	1nfp-d1nfp (3_1_13_2)	1xyza-d1xyza (3_1_1_3)	1hrda-d1hrda2 (3_54_1_1)	4xis-d2xis (3_1_12_1)

861	2cy3-d2cy3	7_26_1_1	118	1	0	0	0	0	0	0	1occj-d1occj1 (6_5_1_1)	1edg-d1edg (3_1_1_3)	1vnc-d1vnc (1_80_1_1)	1csr-d1csh (1_74_1_1)	1fps-d1fps (1_91_1_1)	1aep-d1aep (1_49_1_1)	2myd-d1mbd (1_1_1_1)	1qrda-d1qrda (3_13_4_2)	1ppi-d1ppi_2 (3_1_1_1)
862	2dhd-d1ede	3_50_1_3	310	1	0	0	1	1	0	0	1broa-d1broa (3_50_1_5)	1yasa-d1yasa (3_50_1_10)	1taha-d1tahb (3_50_1_8)	1lbt-d1tca (3_50_1_7)	1ahia-d1fmca (3_19_1_2)	1faga-d2bmha (1_75_1_1)	3tgl-d3tgl (3_50_1_7)	2bnh-d1dfji (3_7_1_1)	1eft-d1eft_3 (3_25_1_3)
863	2dkb-d2dkb	3_48_1_3	431	1	0	0	1	1	0	0	1art-d1art (3_48_1_1)	1fbaa-d1fbaa (3_1_3_1)	1wsyb-d1wsyb (3_59_1_1)	1lucb-d1lucb (3_1_13_1)	2lbp-d2lbp (3_72_1_1)	1miob-d1miob (3_67_1_1)	1mioa-d1mioa (3_67_1_1)	1gca-d1gca (3_72_1_1)	2gpb-d1gpb (3_68_1_2)
864	2dri-d2dri	3_72_1_1	271	6	1	1	0	0	0	0	1gca-d1gca (3_72_1_1)	1tlfa-d1tlfa (3_72_1_1)	1pnr-d1pnra2 (3_72_1_1)	2lbp-d2lbp (3_72_1_1)	1pea-d1pea (3_72_1_1)	1art-d1art (3_48_1_1)	1miob-d1miob (3_67_1_1)	2mnr-d2mnr_1 (3_1_6_2)	1dora-d1dora (3_1_7_1)
865	2dtb-d1dtc	8_7_1_1	26	1	0	0	0	0	0	0	1lis-d1lis (1_17_1_1)	1ilk-d1ilk (1_25_1_3)	1oxa-d1oxa (1_75_1_1)	1gsea-d1gsea1 (1_38_1_1)	1qba-d1qba_1 (2_1_1_5)	1chka-d1chka (4_2_1_6)	1mioa-d1mioa (3_67_1_1)	1dlc-d1dlc_3 (6_1_3_1)	1occi-d1occi1 (6_5_1_1)
866	2ebn-d2ebn	3_1_1_5	285	3	1	1	0	1	1	1	1edt-d1edt (3_1_1_5)	1fbaa-d1fbaa (3_1_3_1)	1oya-d1oya (3_1_7_1)	1eceb-d1ecea (3_1_1_3)	1qba-d1qba_3 (3_1_1_6)	1gln-d1gln_2 (3_15_1_1)	1xyza-d1xyza (3_1_1_3)	1cbg-d1cbg (3_1_1_4)	1cyda-d1cyda (3_19_1_2)
867	2end-d2end	1_16_1_1	137	1	0	0	0	0	0	0	1hdca-d1hdca (3_19_1_2)	4xis-d2xis (3_1_12_1)	2gpb-d1gpb (3_68_1_2)	1bfra-d1bcfa (1_24_1_1)	1nai-d1xel (3_19_1_2)	1eceb-d1ecea (3_1_1_3)	1cbg-d1cbg (3_1_1_4)	1xyza-d1xyza (3_1_1_3)	2ebn-d2ebn (3_1_1_5)
868	2erl-d1erc	1_10_1_1	40	3	0	0	0	0	0	0	1sly-d1sly_1 (1_84_4_1)	1lh7-d1lh1 (1_1_1_1)	1mmob-d1mmob (1_24_1_2)	1rcd-d1rcd (1_24_1_1)	1mtyd-d1mmod (1_24_1_2)	2tct-d2tct_1 (1_31_1_1)	1erp-d1erp (1_10_1_1)	119l-d119l (4_2_1_3)	2sas-d2sas (1_34_1_5)
869	2fxb-d2fxb	4_33_1_4	81	3	1	1	1	1	0	0	1fxra-d1fxra (4_33_1_4)	1rof-d1vjw (4_33_1_4)	1fd2-d1fd2 (4_33_1_2)	1fca-d1fca (4_33_1_1)	4fua-d1fua (3_55_1_1)	2dkb-d2dkb (3_48_1_3)	2trcb-d2trcb (2_46_3_1)	2bnh-d1dfji (3_7_1_1)	1hpca-d1htp (2_59_1_1)
870	2gd1o-d1gd1o2	4_41_1_1	163	1	0	0	0	0	0	0	1scb-d1csee (3_28_1_1)	1gym-d1gym (3_1_15_2)	1llda-d1llda1 (3_19_1_5)	1tig-d1tig (4_35_1_1)	1chma-d1chma2 (4_72_1_1)	2ctb-d2ctb (3_52_3_1)	1eri-d1eria (3_38_1_1)	1xoa-d2trxa (3_33_1_1)	2vik-d2vik (4_60_1_1)
871	2glra-d1glqa2	3_33_1_5	78	3	1	1	1	1	0	0	3gsta-d2gsta2 (3_33_1_5)	1gsea-d1gsea2 (3_33_1_5)	1egr-d1ego (3_33_1_1)	1ghr-d1ghr (3_1_1_3)	2dri-d2dri (3_72_1_1)	1rvva-d1rvva (3_9_1_1)	1llo-d1hvq (3_1_1_5)	3chy-d3chy (3_13_2_1)	1kte-d1kte (3_33_1_1)
872	2gmfa-d1csga	1_25_1_2	120	3	1	1	0	0	0	0	1hula-d1hula (1_25_1_2)	1occa-d1occa1 (6_5_1_1)	1occb-d1occb2 (6_5_1_1)	2end-d2end (1_16_1_1)	1gca-d1gca (3_72_1_1)	1ecpa-d1ecpa (3_52_1_1)	2dkb-d2dkb (3_48_1_3)	1pdo-d1pdo (3_40_1_1)	1cem-d1cem (1_73_1_2)
873	2gpb-d1gpb	3_68_1_2	823	1	0	0	0	0	0	0	1occa-d1occa1 (6_5_1_1)	1art-d1art (3_48_1_1)	1prcl-d1prcl1 (6_5_1_1)	1onea-d1ebha1 (3_1_6_1)	1dora-d1dora (3_1_7_1)	1buca-d1buca1 (1_23_6_1)	1pgo-d2pgd_1 (1_71_1_1)	1mba-d1mba (1_1_1_1)	1hvd-d1hvd (1_51_1_1)
874	2hbg-d1hbg	1_1_1_1	147	10	1	1	1	1	0	0	1baba-d1baba (1_1_1_1)	2myd-d1mbd (1_1_1_1)	1mba-d1mba (1_1_1_1)	2lhb-d2lhb (1_1_1_1)	3sdha-d3sdha (1_1_1_1)	1itha-d1itha (1_1_1_1)	1flp-d1flp (1_1_1_1)	1lh7-d1lh1 (1_1_1_1)	1hlb-d1hlb (1_1_1_1)
875	2hipa-d2hipa	7_27_1_1	71	4	1	1	0	0	0	0	1hpi-d1hpi (7_27_1_1)	1isua-d1isua (7_27_1_1)	1hrq-d1hrq (7_27_1_1)	6ldh-d1ldm_1 (3_19_1_5)	1obr-d1obr (3_52_3_2)	1oxa-d1oxa (1_75_1_1)	1poxa-d1poxa1 (3_21_1_1)	1alo-d1alo_4 (4_77_1_1)	1dxy-d1dxy_1 (3_13_9_1)
876	2hmb-d1hms	2_41_1_2	131	3	1	1	0	1	0	0	1ure-d1ifc (2_41_1_2)	1eal-d1eal (2_41_1_2)	1ospo-d1ospo (2_52_1_1)	1bbpa-d1bbpa (2_41_1_1)	1obpa-d1obpa (2_41_1_1)	2sil-d2sil (2_45_1_1)	2por-d2por (6_7_1_1)	1mpmb-d1mal (6_7_1_2)	3nn9-d3nn9 (2_45_1_1)
877	2hmgd-d1htmb	6_2_1_1	114	1	0	0	0	0	0	0	1rcd-d1rcd (1_24_1_1)	1bfra-d1bcfa (1_24_1_1)	1dkza-d1dkza (5_17_1_1)	1afra-d1afra (1_24_1_2)	1fosf-d1fosf (1_97_2_1)	1fose-d1fose (1_97_2_1)	1ryt-d1ryt_1 (1_24_1_1)	1buca-d1buca1 (1_23_6_1)	1vsga-d1vsga (6_3_1_1)
878	2hmx-d2hmx	1_58_1_1	133	1	0	0	0	0	0	0	1vin-d1vin_1 (1_59_1_1)	1occa-d1occa1 (6_5_1_1)	1miob-d1miob (3_67_1_1)	1xsm-d1xsm (1_24_1_2)	1rlr-d1rlr_1 (1_68_1_1)	1onea-d1ebha2 (4_31_1_1)	1flp-d1flp (1_1_1_1)	1oxa-d1oxa (1_75_1_1)	1cola-d1cola (6_1_1_1)
879	2hnp-d2hnp	3_32_1_2	278	2	1	1	0	1	0	0	1ytn-d1ypta (3_32_1_2)	1rvva-d1rvva (3_9_1_1)	1vhra-d1vhra (3_32_1_1)	1apa-d1apa (4_94_1_1)	1alo-d1alo_7 (4_77_1_1)	1mrj-d1mrj (4_94_1_1)	1hrda-d1hrda2 (3_54_1_1)	1gln-d1gln_2 (3_15_1_1)	1phk-d1phk (5_1_1_1)
880	2ifm-d1ifm	1_97_3_1	46	4	0	1	0	0	0	1	1vsga-d1vsga (6_3_1_1)	1bfra-d1bcfa (1_24_1_1)	1ifl-d1ifl (1_97_3_1)	1fosf-d1fosf (1_97_2_1)	1ifi-d1ifj (1_97_3_1)	1fose-d1fose (1_97_2_1)	2dkb-d2dkb (3_48_1_3)	1ryt-d1ryt_1 (1_24_1_1)	2ccya-d2ccya (1_23_3_2)
881	2ifo-d2ifo	1_97_3_1	46	4	0	0	0	0	0	1	1mmob-d1mmob (1_24_1_2)	1faga-d2bmha (1_75_1_1)	1occc-d1occc1 (6_5_1_1)	1mrrb-d1riba (1_24_1_2)	1fosf-d1fosf (1_97_2_1)	2liga-d2liga (1_23_2_1)	1fose-d1fose (1_97_2_1)	1vsga-d1vsga (6_3_1_1)	1phe-d1phb (1_75_1_1)
882	2lbp-d2lbp	3_72_1_1	346	6	1	1	0	0	0	0	1pea-d1pea (3_72_1_1)	2dri-d2dri (3_72_1_1)	1gca-d1gca (3_72_1_1)	1tlfa-d1tlfa (3_72_1_1)	1pnr-d1pnra2 (3_72_1_1)	1ahia-d1fmca (3_19_1_2)	1dnpa-d1dnpa2 (3_17_1_1)	1nal1-d1nal1 (3_1_3_1)	1miob-d1miob (3_67_1_1)
883	2lhb-d2lhb	1_1_1_1	149	10	1	1	1	1	0	0	1mba-d1mba (1_1_1_1)	2myd-d1mbd (1_1_1_1)	3sdha-d3sdha (1_1_1_1)	1flp-d1flp (1_1_1_1)	1hlb-d1hlb (1_1_1_1)	1baba-d1baba (1_1_1_1)	1itha-d1itha (1_1_1_1)	2hbg-d1hbg (1_1_1_1)	1lh7-d1lh1 (1_1_1_1)
884	2liga-d2liga	1_23_2_1	157	1	0	0	0	0	0	0	1rcd-d1rcd (1_24_1_1)	1dlc-d1dlc_3 (6_1_3_1)	1bfra-d1bcfa (1_24_1_1)	1ciy-d1ciy_3 (6_1_3_1)	1ryt-d1ryt_1 (1_24_1_1)	1occc-d1occc1 (6_5_1_1)	1afra-d1afra (1_24_1_2)	1buca-d1buca1 (1_23_6_1)	1aep-d1aep (1_49_1_1)
885	2masa-d2masa	3_51_1_1	313	1	0	0	0	0	0	0	1gky-d1gky (3_25_1_1)	1art-d1art (3_48_1_1)	1lbt-d1tca (3_50_1_7)	1broa-d1broa (3_50_1_5)	1scub-d1scub1 (3_13_3_1)	1gln-d1gln_2 (3_15_1_1)	1kfd-d1kfd_1 (3_41_3_4)	2bnh-d1dfji (3_7_1_1)	1orta-d1orta1 (3_58_1_1)
886	2mev1-d2mev1	2_8_1_4	268	5	1	1	0	0	0	0	1tme1-d1tme1 (2_8_1_4)	2plv-d1pvc1 (2_8_1_4)	1kiqa-d1vfba (2_1_1_1)	1pmi-d1pmi (2_58_2_1)	1bbpa-d1bbpa (2_41_1_1)	2hmb-d1hms (2_41_1_2)	1nsdb-d1nsca (2_45_1_1)	1lcl-d1lcl (2_19_1_3)	2ncm-d2ncm (2_1_1_4)
887	2mipa-d1idaa	2_34_1_1	99	3	1	1	0	0	0	0	1fmb-d1fmb (2_34_1_1)	1fiva-d1fiva (2_34_1_1)	1nsdb-d1nsca (2_45_1_1)	2gpb-d1gpb (3_68_1_2)	1asqa-d1aoza2 (2_5_1_3)	2sil-d2sil (2_45_1_1)	1iob-d1i1b (2_28_1_2)	4aahc-d4aaha (2_47_1_1)	1dlha-d1dlha1 (2_1_1_2)
888	2mlta-d2mlta	8_18_1_1	26	1	0	0	0	0	0	0	1ciy-d1ciy_3 (6_1_3_1)	1occb-d1occb2 (6_5_1_1)	2anhb-d2anha (3_56_1_1)	1occa-d1occa1 (6_5_1_1)	1dlc-d1dlc_3 (6_1_3_1)	1bmta-d1bmta1 (1_39_1_1)	1bct-d1bct (8_30_1_1)	1tkaa-d1trka1 (3_24_1_2)	1ecma-d1ecma (1_93_1_1)
889	2mnr-d2mnr_1	3_1_6_2	227	2	1	1	1	1	1	1	2chr-d2chr_1 (3_1_6_2)	1onea-d1ebha1 (3_1_6_1)	1nal1-d1nal1 (3_1_3_1)	2dri-d2dri (3_72_1_1)	1dora-d1dora (3_1_7_1)	1nsj-d1nsj (3_1_8_1)	1igs-d1igs (3_1_8_1)	1tlfa-d1tlfa (3_72_1_1)	1eft-d1eft_3 (3_25_1_3)
890	2mnr-d2mnr_2	4_31_1_1	130	3	1	1	0	0	0	0	1chra-d2chr_2 (4_31_1_1)	1tfe-d1tfe (4_24_1_1)	1dkza-d1dkza (5_17_1_1)	1sly-d1sly_1 (1_84_4_1)	1onea-d1ebha2 (4_31_1_1)	1lbd-d1lbd (1_87_1_1)	1asu-d1asu (3_41_3_2)	1orda-d1orda2 (3_48_1_4)	1cpcb-d1cpcb (1_1_1_2)
891	2ms2a-d2ms2a	4_45_1_1	129	1	0	0	0	0	0	0	1ngi-d1hpm_2 (3_41_1_1)	1eac-d1eaf (3_30_1_1)	1dkza-d1dkza (5_17_1_1)	1mpmb-d1mal (6_7_1_2)	2por-d2por (6_7_1_1)	1seta-d1seta2 (4_59_1_1)	1bgl-d1bgla1 (2_1_3_1)	1obpa-d1obpa (2_41_1_1)	1mkaa-d1mkaa (4_21_1_1)
892	2mtac-d2mtac	1_3_1_1	147	7	0	1	0	1	0	0	1cem-d1cem (1_73_1_2)	1gks-d1gks (1_3_1_1)	1cyj-d1cyi (1_3_1_1)	1fcdc-d1fcdc2 (1_3_1_3)	1dvh-d1dvh (1_3_1_1)	1fcdc-d1fcdc1 (1_3_1_3)	1etpa-d1etpa1 (1_3_1_3)	1ngi-d1hpm_2 (3_41_1_1)	1qba-d1qba_3 (3_1_1_6)
893	2mtal-d2bbkl	7_19_1_1	125	1	0	0	0	0	0	0	1tsra-d1tupa (2_2_3_1)	1apme-d1apme (5_1_1_1)	1mpmb-d1mal (6_7_1_2)	2csn-d1csn (5_1_1_1)	1aocb-d1aoca (7_14_1_5)	5sgae-d1sgc (2_31_1_1)	4aahc-d4aaha (2_47_1_1)	2hnp-d2hnp (3_32_1_2)	1prn-d1prn (6_7_1_1)
894	2myd-d1mbd	1_1_1_1	153	10	1	1	1	1	0	0	1baba-d1baba (1_1_1_1)	2lhb-d2lhb (1_1_1_1)	1mba-d1mba (1_1_1_1)	1itha-d1itha (1_1_1_1)	1lh7-d1lh1 (1_1_1_1)	1hlb-d1hlb (1_1_1_1)	2hbg-d1hbg (1_1_1_1)	3sdha-d3sdha (1_1_1_1)	1cpca-d1cpca (1_1_1_2)
895	2naca-d2naca2	3_19_1_4	188	4	1	1	1	1	0	0	1gdha-d1gdha2 (3_19_1_4)	1psda-d1psda2 (3_19_1_4)	1dxy-d1dxy_2 (3_19_1_4)	1leha-d1leha1 (3_19_1_7)	1gtma-d1gtma1 (3_19_1_7)	1pgn-d2pgd_2 (3_19_1_6)	1hrda-d1hrda1 (3_19_1_7)	1rada-d1raaa2 (3_58_1_1)	1nis-d1aco_2 (3_63_1_1)
896	2ncm-d2ncm	2_1_1_4	99	5	1	1	1	1	0	1	1vcaa-d1vcaa2 (2_1_1_4)	1tlk-d1tlk (2_1_1_4)	3cd4-d3cd4_1 (2_1_1_1)	1wit-d1wiu (2_1_1_4)	1ospo-d1ospo (2_52_1_1)	1cdi-d3cd4_2 (2_1_1_3)	1kiqa-d1vfba (2_1_1_1)	1cdcb-d1cdcb (2_1_1_1)	1eal-d1eal (2_41_1_2)
897	2npx-d1nhp_3	4_46_1_1	126	5	1	1	0	0	0	0	1lvl-d1lvl_3 (4_46_1_1)	1fead-d2tpra3 (4_46_1_1)	1gesa-d1gesa3 (4_46_1_1)	1ospo-d1ospo (2_52_1_1)	1asza-d1asya2 (4_59_1_1)	1alo-d1alo_7 (4_77_1_1)	1gpc-d1gpc (2_26_4_7)	2por-d2por (6_7_1_1)	2ada-d1add (3_1_2_1)
898	2omf-d2omf	6_7_1_1	340	3	1	1	1	1	0	0	1prn-d1prn (6_7_1_1)	2por-d2por (6_7_1_1)	1mpmb-d1mal (6_7_1_2)	2sil-d2sil (2_45_1_1)	1ospo-d1ospo (2_52_1_1)	1eur-d1eur (2_45_1_1)	1con-d1scs (2_19_1_1)	1xnb-d1xnb (2_19_1_8)	1obpa-d1obpa (2_41_1_1)
899	2ora-d1orb_1	3_60_1_1	149	2	1	1	0	0	0	0	1orb-d1orb_2 (3_60_1_1)	1nis-d1aco_2 (3_63_1_1)	1dora-d1dora (3_1_7_1)	1sfta-d1sfta2 (3_1_18_1)	1tlfa-d1tlfa (3_72_1_1)	2dri-d2dri (3_72_1_1)	1rcf-d1rcf (3_13_4_1)	1qba-d1qba_3 (3_1_1_6)	1rnl-d1rnl_2 (3_13_2_1)
900	2pcd-d2pcda	2_3_3_1	200	1	0	0	0	0	0	0	2avia-d2avia (2_42_1_1)	1fnc-d1fnb_1 (2_29_1_1)	1hpm-d1hpm_1 (3_41_1_1)	1fem-d1hbp (2_41_1_1)	1qatb-d1djxa2 (2_6_1_1)	1noa-d1noa (2_1_6_1)	2sil-d2sil (2_45_1_1)	2bfh-d2fgf (2_28_1_1)	4aahc-d4aaha (2_47_1_1)
901	2pcy-d1plc	2_5_1_1	99	4	0	1	1	1	0	0	1kcw-d1kcw_4 (2_5_1_3)	1occb-d1occb1 (2_5_1_2)	1kcw-d1kcw_2 (2_5_1_3)	2cbp-d2cbp (2_5_1_1)	1kcw-d1kcw_6 (2_5_1_3)	1aspb-d1aoza1 (2_5_1_3)	1asob-d1aoza3 (2_5_1_3)	1kcw-d1kcw_5 (2_5_1_3)	1thw-d1thw (2_18_1_1)
902	2pec-d2pec	2_56_1_1	352	1	0	0	0	0	0	0	1llo-d1hvq (3_1_1_5)	1shca-d1shca (2_37_1_2)	7cat-d7cata (5_6_1_1)	1svb-d1svb_2 (6_10_1_1)	1leha-d1leha1 (3_19_1_7)	1xnb-d1xnb (2_19_1_8)	1akl-d1kapp1 (2_55_1_1)	2trcb-d2trcb (2_46_3_1)	6ldh-d1ldm_1 (3_19_1_5)
903	2phl-d2phla1	2_58_1_1	200	1	0	0	0	0	0	0	1eal-d1eal (2_41_1_2)	1ure-d1ifc (2_41_1_2)	2hmb-d1hms (2_41_1_2)	1ospo-d1ospo (2_52_1_1)	1ggt-d1ggta2 (2_1_4_1)	3nn9-d3nn9 (2_45_1_1)	1prn-d1prn (6_7_1_1)	4kbpb-d4kbpa1 (2_1_9_1)	2omf-d2omf (6_7_1_1)
904	2phy-d2phy	4_61_2_1	125	1	0	0	0	0	0	0	1rcf-d1rcf (3_13_4_1)	1bebb-d1beba (2_41_1_1)	1lvl-d1lvl_2 (3_4_1_4)	7icd-d7icd (3_57_1_1)	1ctf-d1ctf (4_26_1_1)	4mhta-d1hmy (3_47_1_4)	2anhb-d2anha (3_56_1_1)	1tnra-d1tnra (2_17_1_1)	1mbt-d1mbb_2 (4_85_1_1)
905	2pia-d2pia_1	2_29_1_2	103	1	0	0	1	1	0	0	1cnf-d2cnd_1 (2_29_1_1)	1fnc-d1fnb_1 (2_29_1_1)	2bfh-d2fgf (2_28_1_1)	1gof-d1gof_3 (2_46_1_1)	1eur-d1eur (2_45_1_1)	1fem-d1hbp (2_41_1_1)	1bgl-d1bgla2 (2_1_3_1)	1cyx-d1cyx (2_5_1_2)	3pte-d3pte (5_4_1_1)
906	2pia-d2pia_2	3_14_1_2	120	1	0	0	1	1	0	0	1fnc-d1fnb_2 (3_14_1_1)	1cnf-d2cnd_2 (3_14_1_1)	1rvva-d1rvva (3_9_1_1)	1tkca-d1trka2 (3_24_1_2)	3btoa-d2ohxa2 (3_19_1_1)	1gph1-d1gph12 (4_88_1_1)	1pyda-d1pvda3 (3_24_1_1)	1psda-d1psda2 (3_19_1_4)	1hora-d1deaa (3_23_1_1)
907	2pia-d2pia_3	4_12_4_1	98	3	1	1	0	0	0	1	1frd-d1frd (4_12_4_1)	1eac-d1eaf (3_30_1_1)	2bfh-d2fgf (2_28_1_1)	7icd-d7icd (3_57_1_1)	1tif-d1tif (4_12_6_1)	1sgk-d1ddt_2 (4_95_1_1)	1seia-d1seia (4_80_1_1)	1put-d1put (4_12_4_1)	4aahc-d4aaha (2_47_1_1)
908	2plda-d2plda	4_51_1_1	105	3	1	1	0	0	0	0	1shaa-d1shaa (4_51_1_1)	1ouna-d1ouna (4_14_3_2)	1vcaa-d1vcaa2 (2_1_1_4)	1gph1-d1gph12 (4_88_1_1)	1gdob-d1gdoa (4_88_1_1)	1hpm-d1hpm_1 (3_41_1_1)	1mrj-d1mrj (4_94_1_1)	1amp-d1amp (3_52_3_4)	1onea-d1ebha2 (4_31_1_1)

909	2plv-d1pvc1	2_8_1_4	279	5	1	1	1	1	0	0	1tme1-d1tme1 (2_8_1_4)	2mev1-d2mev1 (2_8_1_4)	1smvc-d1smva (2_8_1_2)	1hgea-d1hgea (2_14_1_2)	2bbvc-d2bbva (2_8_1_3)	1bmv1-d1bmv1 (2_8_1_2)	1ggt-d1ggta2 (2_1_4_1)	1dlc-d1dlc_2 (2_53_2_1)	1ouna-d1ouna (4_14_3_2)
910	2pnb-d2pnb	4_51_1_1	104	3	0	0	0	0	0	0	1pex-d1pex (2_44_1_1)	1hpm-d1hpm_1 (3_41_1_1)	2pola-d2pola1 (4_76_1_1)	1vcaa-d1vcaa2 (2_1_1_4)	1shca-d1shca (2_37_1_2)	1plr-d1plq_2 (4_76_1_2)	2plda-d2plda (4_51_1_1)	1bbpa-d1bbpa (2_41_1_1)	1ure-d1ifc (2_41_1_2)
911	2pola-d2pola1	4_76_1_1	122	3	1	1	0	1	0	0	2pola-d2pola2 (4_76_1_1)	2pola-d2pola3 (4_76_1_1)	2hmb-d1hms (2_41_1_2)	1plq-d1plq_1 (4_76_1_2)	1eal-d1eal (2_41_1_2)	1pex-d1pex (2_44_1_1)	1hgea-d1hgea (2_14_1_2)	3nn9-d3nn9 (2_45_1_1)	2sil-d2sil (2_45_1_1)
912	2pola-d2pola2	4_76_1_1	122	3	1	1	1	1	0	0	2pola-d2pola1 (4_76_1_1)	2pola-d2pola3 (4_76_1_1)	1plr-d1plq_2 (4_76_1_2)	2hmb-d1hms (2_41_1_2)	1ure-d1ifc (2_41_1_2)	2por-d2por (6_7_1_1)	1eal-d1eal (2_41_1_2)	1bbpa-d1bbpa (2_41_1_1)	1exg-d1exg (2_2_2_1)
913	2pola-d2pola3	4_76_1_1	122	3	0	1	1	1	0	0	1plr-d1plq_2 (4_76_1_2)	2pola-d2pola2 (4_76_1_1)	1plq-d1plq_1 (4_76_1_2)	2pola-d2pola1 (4_76_1_1)	2hmb-d1hms (2_41_1_2)	1ospo-d1ospo (2_52_1_1)	1obpa-d1obpa (2_41_1_1)	1apa-d1apa (4_94_1_1)	1gph1-d1gph12 (4_88_1_1)
914	2por-d2por	6_7_1_1	301	3	1	1	1	1	0	0	1prn-d1prn (6_7_1_1)	2omf-d2omf (6_7_1_1)	1mpmb-d1mal (6_7_1_2)	2sil-d2sil (2_45_1_1)	1bgl-d1bgla1 (2_1_3_1)	2hmb-d1hms (2_41_1_2)	1ure-d1ifc (2_41_1_2)	1hgea-d1hgea (2_14_1_2)	1con-d1scs (2_19_1_1)
915	2prd-d2prd	2_26_5_1	174	1	0	0	0	0	0	0	4aahc-d4aaha (2_47_1_1)	2trcb-d2trcb (2_46_3_1)	1gtma-d1gtma2 (3_54_1_1)	2bbkh-d2bbkh (2_46_2_1)	1fnc-d1fnb_1 (2_29_1_1)	1tsra-d1tupa (2_2_3_1)	1fbaa-d1fbaa (3_1_3_1)	2gpb-d1gpb (3_68_1_2)	1gym-d1gym (3_1_15_2)
916	2pspa-d1pspa2	7_13_1_1	52	1	0	0	0	0	0	0	1jud-d1jud (5_18_1_1)	1psda-d1psda2 (3_19_1_4)	2anhb-d2anha (3_56_1_1)	1dik-d1dik_2 (3_5_1_1)	1zymb-d1zyma (3_5_1_2)	1gcb-d1gcb (4_3_1_1)	1tpfa-d1tpfa (3_1_11_1)	1oiba-d1pbp (3_73_1_1)	1lvl-d1lvl_2 (3_4_1_4)
917	2qila-d1tssa2	4_12_5_1	101	2	1	1	0	0	0	0	1sea-d1esfa2 (4_12_5_1)	1ngi-d1hpm_2 (3_41_1_1)	1apa-d1apa (4_94_1_1)	1chma-d1chma2 (4_72_1_1)	3tgl-d3tgl (3_50_1_7)	1xoa-d2trxa (3_33_1_1)	1han-d1han_2 (4_20_1_3)	1mrj-d1mrj (4_94_1_1)	1raca-d1raaa1 (3_58_1_1)
918	2reb-d2reb_2	4_27_1_1	60	1	0	0	0	0	0	0	1mml-d1mml (5_9_1_2)	1kte-d1kte (3_33_1_1)	1mrrb-d1riba (1_24_1_2)	1itea-d2int (1_25_1_2)	1bfra-d1bcfa (1_24_1_1)	1bdmb-d1bdma2 (4_92_1_1)	1rcd-d1rcd (1_24_1_1)	1orda-d1orda2 (3_48_1_4)	1faga-d2bmha (1_75_1_1)
919	2sas-d2sas	1_34_1_5	185	5	1	1	0	1	0	0	2scpa-d2scpa (1_34_1_5)	1osa-d1osa (1_34_1_5)	1tcob-d1tcob (1_34_1_5)	1flp-d1flp (1_1_1_1)	1wdcb-d1scmb (1_34_1_5)	1sra-d1sra (1_34_1_3)	1cpca-d1cpca (1_1_1_2)	1cpcb-d1cpcb (1_1_1_2)	2abk-d2abk (1_66_1_1)
920	2scpa-d2scpa	1_34_1_5	174	5	1	1	1	1	0	0	2sas-d2sas (1_34_1_5)	1osa-d1osa (1_34_1_5)	1tcob-d1tcob (1_34_1_5)	1sra-d1sra (1_34_1_3)	1wdcb-d1scmb (1_34_1_5)	1itha-d1itha (1_1_1_1)	2abk-d2abk (1_66_1_1)	1occa-d1occa1 (6_5_1_1)	3sdha-d3sdha (1_1_1_1)
921	2sh1-d1sh1	7_7_1_1	48	5	1	1	0	0	0	0	1bnb-d1bnb (7_7_1_1)	1gym-d1gym (3_1_15_2)	2omf-d2omf (6_7_1_1)	1afp-d1afp (7_23_1_1)	1gcb-d1gcb (4_3_1_1)	1tsg-d1tsg (4_97_1_3)	1udh-d1udg (3_11_1_1)	1hsq-d1hsq (2_21_2_1)	1vhra-d1vhra (3_32_1_1)
922	2sici-d2sici	4_44_1_1	107	1	0	0	0	0	0	0	1mola-d1mola (4_14_1_1)	1ecpa-d1ecpa (3_52_1_1)	1kcw-d1kcw_5 (2_5_1_3)	1geua-d1gesa2 (3_4_1_4)	1pda-d1pda_2 (4_28_2_1)	1dlha-d1dlha1 (2_1_1_2)	1ouna-d1ouna (4_14_3_2)	1xva-d1xvaa (3_47_1_2)	1scub-d1scub1 (3_13_3_1)
923	2sil-d2sil	2_45_1_1	381	4	1	1	0	0	0	0	1eur-d1eur (2_45_1_1)	3nn9-d3nn9 (2_45_1_1)	1nsdb-d1nsca (2_45_1_1)	2trcb-d2trcb (2_46_3_1)	4aahc-d4aaha (2_47_1_1)	1ospo-d1ospo (2_52_1_1)	2por-d2por (6_7_1_1)	2omf-d2omf (6_7_1_1)	2hmb-d1hms (2_41_1_2)
924	2sn3-d2sn3	7_3_6_1	65	1	0	0	0	0	0	0	1bll-d1lam_2 (3_52_3_3)	1noy-d1noya (3_41_3_5)	1prtd-d1prtd (2_26_2_1)	1han-d1han_2 (4_20_1_3)	1gky-d1gky (3_25_1_1)	1amy-d1amy_2 (3_1_1_1)	1mpd-d1mpb (3_73_1_1)	1htda-d1atla (4_50_1_5)	1tbd-d1tbd (4_48_1_1)
925	2stv-d2stv	2_8_1_2	184	4	0	0	0	0	0	0	1tnfa-d1tnfa (2_17_1_1)	1toxa-d1ddt_1 (2_2_1_1)	1obpa-d1obpa (2_41_1_1)	1mpmb-d1mal (6_7_1_2)	2omf-d2omf (6_7_1_1)	1svb-d1svb_2 (6_10_1_1)	2bpa1-d2bpa1 (2_8_1_1)	1dkza-d1dkza (5_17_1_1)	1cdaa-d1aly (2_17_1_1)
926	2tcl-d1hfc	4_50_1_7	157	1	0	0	1	1	0	0	1akl-d1kapp2 (4_50_1_6)	1kuh-d1kuh (4_50_1_1)	1htda-d1atla (4_50_1_5)	2vik-d2vik (4_60_1_1)	1kob-d1koba (5_1_1_1)	2scpa-d2scpa (1_34_1_5)	1raca-d1raaa1 (3_58_1_1)	1nfp-d1nfp (3_1_13_2)	1iad-d1ast (4_50_1_4)
927	2tct-d2tct_1	1_31_1_1	66	1	0	0	0	0	0	0	1vola-d1vola1 (1_59_1_2)	119l-d119l (4_2_1_3)	1mtyg-d1mmog (1_22_1_1)	1octc-d1octc1 (1_4_1_1)	2sas-d2sas (1_34_1_5)	2hnp-d2hnp (3_32_1_2)	1llib-d1llia (1_30_1_2)	1zymb-d1zyma (3_5_1_2)	1rnl-d1rnl_1 (1_31_1_2)
928	2tgi-d1tfg	7_14_1_2	112	1	0	0	0	0	0	0	1oaca-d1oaca2 (4_14_2_1)	1acf-d1acf (4_61_1_1)	3rubs-d3rubs (4_37_1_1)	2cas-d2cas (2_8_1_4)	1eri-d1eria (3_38_1_1)	2plda-d2plda (4_51_1_1)	1nsdb-d1nsca (2_45_1_1)	1lucb-d1lucb (3_1_13_1)	1gpc-d1gpc (2_26_4_7)
929	2tmda-d2tmda1	3_1_7_1	340	3	1	1	0	0	1	1	1oya-d1oya (3_1_7_1)	4xis-d2xis (3_1_12_1)	1cb2b-d1cb2a (3_2_1_1)	2chr-d2chr_1 (3_1_6_2)	1php-d1php (3_66_1_1)	1bya-d1byb (3_1_1_2)	2mnr-d2mnr_1 (3_1_6_2)	1onea-d1ebha1 (3_1_6_1)	1dora-d1dora (3_1_7_1)
930	2tmda-d2tmda2	3_4_1_1	156	1	0	0	1	1	0	0	1lvl-d1lvl_2 (3_4_1_4)	1geua-d1gesa2 (3_4_1_4)	1nhq-d1nhp_2 (3_4_1_4)	1febb-d2tpra2 (3_4_1_4)	1coy-d1coy_1 (3_4_1_2)	1ure-d1ifc (2_41_1_2)	1tpfa-d1tpfa (3_1_11_1)	1iow-d2dln_1 (3_20_1_2)	1orta-d1orta2 (3_58_1_1)
931	2trcb-d2trcb	2_46_3_1	340	1	0	0	0	0	1	1	2bbkh-d2bbkh (2_46_2_1)	4aahc-d4aaha (2_47_1_1)	1ospo-d1ospo (2_52_1_1)	1gof-d1gof_3 (2_46_1_1)	1eur-d1eur (2_45_1_1)	3nn9-d3nn9 (2_45_1_1)	2sil-d2sil (2_45_1_1)	1pex-d1pex (2_44_1_1)	1msad-d1msaa (2_54_1_1)
932	2trcg-d2trcg	8_47_1_1	68	2	1	1	0	0	0	0	1gp2g-d1gp2g (8_47_1_1)	1tfe-d1tfe (4_24_1_1)	1vin-d1vin_1 (1_59_1_1)	1mba-d1mba (1_1_1_1)	1glm-d1glm (1_73_1_1)	1flp-d1flp (1_1_1_1)	1rpl-d1rpl (5_10_1_1)	1lucb-d1lucb (3_1_13_1)	1jkw-d1jkw_1 (1_59_1_1)
933	2utga-d1utg	1_72_1_1	70	1	0	0	0	0	0	0	1dlc-d1dlc_3 (6_1_3_1)	1csr-d1csh (1_74_1_1)	2ada-d1add (3_1_2_1)	1flp-d1flp (1_1_1_1)	1ecma-d1ecma (1_93_1_1)	2wrpr-d2wrpr (1_78_1_1)	1zymb-d1zyma (3_5_1_2)	1occe-d1occe (1_84_7_1)	2liga-d2liga (1_23_2_1)
934	2vik-d2vik	4_60_1_1	126	2	0	0	0	1	0	0	1fem-d1hbp (2_41_1_1)	1bbpa-d1bbpa (2_41_1_1)	1cof-d1ahq (4_60_1_2)	1dkza-d1dkza (5_17_1_1)	1obpa-d1obpa (2_41_1_1)	1lyla-d1lyla2 (4_59_1_1)	2ebn-d2ebn (3_1_1_5)	1eri-d1eria (3_38_1_1)	1ciy-d1ciy_2 (2_53_2_1)
935	2wrpr-d2wrpr	1_78_1_1	104	1	0	0	0	0	0	0	1bfra-d1bcfa (1_24_1_1)	1xsm-d1xsm (1_24_1_2)	1sly-d1sly_2 (4_2_1_5)	1occc-d1occc1 (6_5_1_1)	1lis-d1lis (1_17_1_1)	1buca-d1buca1 (1_23_6_1)	1sesa-d1seta1 (1_2_3_1)	1rcd-d1rcd (1_24_1_1)	1mrrb-d1riba (1_24_1_2)
936	3ait-d1hoe	2_4_1_1	74	1	0	0	0	0	0	0	1ospo-d1ospo (2_52_1_1)	1vjs-d1bplb1 (2_48_1_1)	1p03a-d2alp (2_31_1_1)	1mjc-d1mjc (2_26_4_4)	1noa-d1noa (2_1_6_1)	1runb-d1cgpa2 (2_58_3_1)	1gdob-d1gdoa (4_88_1_1)	1fnc-d1fnb_1 (2_29_1_1)	1qila-d1tssa1 (2_26_2_2)
937	3b5c-d3b5c	4_66_1_1	85	1	0	0	0	0	0	0	1smpi-d1smpi (2_42_2_1)	1prhb-d1prha1 (1_65_1_2)	1itha-d1itha (1_1_1_1)	1pbn-d1pbn (3_52_1_1)	1cid-d1cid_1 (2_1_1_1)	2sil-d2sil (2_45_1_1)	2btf-d2btfa2 (3_41_1_1)	1ospo-d1ospo (2_52_1_1)	1poba-d1poa (1_95_1_2)
938	3btoa-d2ohxa2	3_19_1_1	150	2	1	1	1	1	0	0	1qora-d1qora2 (3_19_1_1)	1cyda-d1cyda (3_19_1_2)	1nal1-d1nal1 (3_1_3_1)	1pnr-d1pnra2 (3_72_1_1)	1igs-d1igs (3_1_8_1)	1reqb-d1reqb2 (3_13_5_2)	1psda-d1psda2 (3_19_1_4)	1pgn-d2pgd_2 (3_19_1_6)	1bncb-d1bnca2 (3_20_1_1)
939	3cd4-d3cd4_1	2_1_1_1	97	4	0	1	1	1	0	0	2ncm-d2ncm (2_1_1_4)	1vcaa-d1vcaa2 (2_1_1_4)	1cid-d1cid_1 (2_1_1_1)	1tlk-d1tlk (2_1_1_4)	1wit-d1wiu (2_1_1_4)	1kiqa-d1vfba (2_1_1_1)	2hmb-d1hms (2_41_1_2)	1ospo-d1ospo (2_52_1_1)	1cdcb-d1cdcb (2_1_1_1)
940	3chy-d3chy	3_13_2_1	128	4	1	1	0	0	0	0	1srrc-d1srra (3_13_2_1)	1rnl-d1rnl_2 (3_13_2_1)	1pii-d1pii_2 (3_1_8_1)	1nal1-d1nal1 (3_1_3_1)	1igs-d1igs (3_1_8_1)	2dri-d2dri (3_72_1_1)	1rvva-d1rvva (3_9_1_1)	1dora-d1dora (3_1_7_1)	1tml-d1tml (3_2_1_1)
941	3dfr-d3dfr	3_53_1_1	162	1	0	0	0	0	0	0	1poxa-d1poxa3 (3_24_1_1)	2bnh-d1dfji (3_7_1_1)	1pbn-d1pbn (3_52_1_1)	1han-d1han_1 (4_20_1_3)	2phl-d2phla1 (2_58_1_1)	1scub-d1scub2 (4_83_1_3)	1smna-d1smna (4_71_1_1)	1mioa-d1mioa (3_67_1_1)	1gky-d1gky (3_25_1_1)
942	3fx2-d2fx2	3_13_4_1	147	3	1	1	0	0	0	0	1fld-d5nul (3_13_4_1)	1rcf-d1rcf (3_13_4_1)	2ada-d1add (3_1_2_1)	2dri-d2dri (3_72_1_1)	1tlfa-d1tlfa (3_72_1_1)	1rvva-d1rvva (3_9_1_1)	1mioa-d1mioa (3_67_1_1)	1wsyb-d1wsyb (3_59_1_1)	1gca-d1gca (3_72_1_1)
943	3gsta-d2gsta1	1_38_1_1	133	3	1	1	0	0	0	0	1glqa-d1glqa1 (1_38_1_1)	1gsea-d1gsea1 (1_38_1_1)	2hbg-d1hbg (1_1_1_1)	1prcl-d1prcl1 (6_5_1_1)	1lis-d1lis (1_17_1_1)	1xsm-d1xsm (1_24_1_2)	1occc-d1occc1 (6_5_1_1)	1knya-d1knya (5_10_1_2)	2lhb-d2lhb (1_1_1_1)
944	3gsta-d2gsta2	3_33_1_5	84	3	1	1	1	1	0	0	2glra-d1glqa2 (3_33_1_5)	1gsea-d1gsea2 (3_33_1_5)	1egr-d1ego (3_33_1_1)	1ghr-d1ghr (3_1_1_3)	1gym-d1gym (3_1_15_2)	1kte-d1kte (3_33_1_1)	1xoa-d2trxa (3_33_1_1)	1fbaa-d1fbaa (3_1_3_1)	1hrda-d1hrda1 (3_19_1_7)
945	3hhrb-d3hhrb2	2_1_2_1	104	4	1	1	0	0	0	0	1ten-d1ten (2_1_2_1)	1fna-d1fna (2_1_2_1)	1svb-d1svb_2 (6_10_1_1)	2bpa1-d2bpa1 (2_8_1_1)	1cfb-d1cfb_1 (2_1_2_1)	3nn9-d3nn9 (2_45_1_1)	1kcw-d1kcw_5 (2_5_1_3)	1dkza-d1dkza (5_17_1_1)	2plv-d1pvc1 (2_8_1_4)
946	3mdsa-d1mnga1	1_2_4_1	92	1	0	0	0	0	0	1	1mba-d1mba (1_1_1_1)	1oxa-d1oxa (1_75_1_1)	1grj-d1grj_1 (1_2_1_1)	1lbd-d1lbd (1_87_1_1)	1rpa-d1rpa (3_43_1_2)	1occa-d1occa1 (6_5_1_1)	1mtyd-d1mmod (1_24_1_2)	1itha-d1itha (1_1_1_1)	1mrrb-d1riba (1_24_1_2)
947	3mdsa-d1mnga2	4_25_1_1	111	1	0	0	0	0	0	0	1iov-d2dln_2 (4_83_1_1)	1geua-d1gesa2 (3_4_1_4)	1apa-d1apa (4_94_1_1)	2tmda-d2tmda1 (3_1_7_1)	1dlh-d1dlha2 (4_15_1_1)	1abra-d1abra (4_94_1_1)	1ctt-d1ctt_1 (3_75_1_1)	1ppi-d1ppi_2 (3_1_1_1)	1obr-d1obr (3_52_3_2)
948	3nn9-d3nn9	2_45_1_1	388	4	1	1	0	0	0	0	1nsdb-d1nsca (2_45_1_1)	1eur-d1eur (2_45_1_1)	2sil-d2sil (2_45_1_1)	2trcb-d2trcb (2_46_3_1)	1ospo-d1ospo (2_52_1_1)	4aahc-d4aaha (2_47_1_1)	1lcl-d1lcl (2_19_1_3)	1gof-d1gof_3 (2_46_1_1)	2hmb-d1hms (2_41_1_2)
949	3pgm-d3pgm	3_43_1_1	230	1	0	0	0	0	0	0	1miob-d1miob (3_67_1_1)	2lbp-d2lbp (3_72_1_1)	2dri-d2dri (3_72_1_1)	1gca-d1gca (3_72_1_1)	4xis-d2xis (3_1_12_1)	1orb-d1orb_2 (3_60_1_1)	1asu-d1asu (3_41_3_2)	2chr-d2chr_1 (3_1_6_2)	1tlfa-d1tlfa (3_72_1_1)
950	3pmga-d3pmga1	3_64_1_1	190	3	0	0	0	0	0	0	1nal1-d1nal1 (3_1_3_1)	1tlfa-d1tlfa (3_72_1_1)	1reqb-d1reqb2 (3_13_5_2)	1yasa-d1yasa (3_50_1_10)	1pnr-d1pnra2 (3_72_1_1)	1art-d1art (3_48_1_1)	1pea-d1pea (3_72_1_1)	3tgl-d3tgl (3_50_1_7)	1gpma-d1gpma2 (3_49_1_1)
951	3pte-d3pte	5_4_1_1	347	3	1	1	0	0	0	0	2blta-d2blta (5_4_1_1)	1btl-d1btl (5_4_1_1)	1ctt-d1ctt_1 (3_75_1_1)	1onea-d1ebha1 (3_1_6_1)	1ospo-d1ospo (2_52_1_1)	1juy-d1adea (3_25_1_5)	1gtra-d1gtra2 (3_15_1_1)	2trcb-d2trcb (2_46_3_1)	1leha-d1leha2 (3_54_1_1)
952	3rnt-d9rnt	4_1_1_1	104	3	1	1	0	0	0	0	1brnl-d1brnl (4_1_1_1)	1eur-d1eur (2_45_1_1)	1wba-d1wba (2_28_3_1)	1kxa-d2snv (2_31_1_3)	1gpr-d1gpr (2_59_3_1)	1cmc-d1cmba (1_36_1_2)	1nfp-d1nfp (3_1_13_2)	1prcl-d1prcl1 (6_5_1_1)	1emd-d2cmd_2 (4_92_1_1)
953	3rubs-d3rubs	4_37_1_1	123	1	0	0	0	0	0	0	1eceb-d1ecea (3_1_1_3)	1bll-d1lam_2 (3_52_3_3)	1amp-d1amp (3_52_3_4)	1udh-d1udg (3_11_1_1)	1poxa-d1poxa2 (3_24_1_1)	1wsyb-d1wsyb (3_59_1_1)	1qrda-d1qrda (3_13_4_2)	1nai-d1xel (3_19_1_2)	2aaa-d2aaa_2 (3_1_1_1)
954	3sdha-d3sdha	1_1_1_1	145	10	1	1	1	1	0	0	1mba-d1mba (1_1_1_1)	2lhb-d2lhb (1_1_1_1)	1flp-d1flp (1_1_1_1)	1itha-d1itha (1_1_1_1)	2myd-d1mbd (1_1_1_1)	1lh7-d1lh1 (1_1_1_1)	2hbg-d1hbg (1_1_1_1)	1hlb-d1hlb (1_1_1_1)	1cpca-d1cpca (1_1_1_2)
955	3tgl-d3tgl	3_50_1_7	265	3	1	1	0	1	0	0	1tib-d1tib (3_50_1_7)	1crr-d5p21 (3_25_1_3)	1xyza-d1xyza (3_1_1_3)	1gca-d1gca (3_72_1_1)	2dhd-d1ede (3_50_1_3)	1apme-d1apme (5_1_1_1)	1mml-d1mml (5_9_1_2)	1tml-d1tml (3_2_1_1)	3pmga-d3pmga1 (3_64_1_1)
956	451c-d351c	1_3_1_1	82	7	1	1	0	0	0	0	1cyj-d1cyi (1_3_1_1)	1pbwa-d1pbwa (1_83_1_1)	1raca-d1raaa1 (3_58_1_1)	1fbaa-d1fbaa (3_1_3_1)	1fps-d1fps (1_91_1_1)	1paw-d1pax_1 (1_35_1_1)	1lucb-d1lucb (3_1_13_1)	1gdz-d1ak4c (1_57_1_1)	1ctf-d1ctf (4_26_1_1)

957	4aahc-d4aaha	2_47_1_1	571	1	0	0	0	0	0	0	2trcb-d2trcb (2_46_3_1)	1gof-d1gof_3 (2_46_1_1)	2bbkh-d2bbkh (2_46_2_1)	1ospo-d1ospo (2_52_1_1)	2sil-d2sil (2_45_1_1)	1eur-d1eur (2_45_1_1)	3nn9-d3nn9 (2_45_1_1)	1pex-d1pex (2_44_1_1)	2prd-d2prd (2_26_5_1)
958	4aahd-d4aahb	8_46_1_1	69	1	0	0	0	0	0	0	1cpcb-d1cpcb (1_1_1_2)	1bbha-d1bbha (1_23_3_2)	1ezm-d1ezm_1 (1_53_1_1)	1ghr-d1ghr (3_1_1_3)	1dcha-d1dcoa (4_38_1_1)	1hvd-d1hvd (1_51_1_1)	1pgo-d2pgd_1 (1_71_1_1)	1gbs-d1gbs (4_2_1_4)	1ryt-d1ryt_1 (1_24_1_1)
959	4fua-d1fua	3_55_1_1	206	1	0	0	0	0	0	0	1ytn-d1ypta (3_32_1_2)	1wsyb-d1wsyb (3_59_1_1)	1tlfa-d1tlfa (3_72_1_1)	2sas-d2sas (1_34_1_5)	1bll-d1lam_2 (3_52_3_3)	1dhr-d1dhr (3_19_1_2)	1bfra-d1bcfa (1_24_1_1)	1osa-d1osa (1_34_1_5)	1xsm-d1xsm (1_24_1_2)
960	4gcr-d4gcr_2	2_9_1_1	89	3	1	1	1	1	0	0	1gcs-d4gcr_1 (2_9_1_1)	1blba-d2bb2_2 (2_9_1_1)	1prr-d1prr_1 (2_9_1_2)	1prs-d1prr_2 (2_9_1_2)	1vid-d1vid (3_47_1_1)	1gof-d1gof_2 (2_13_1_1)	1kcw-d1kcw_2 (2_5_1_3)	3nn9-d3nn9 (2_45_1_1)	2trcb-d2trcb (2_46_3_1)
961	4kbpa-d4kbpa2	4_91_1_1	312	1	0	0	0	0	0	0	1gen-d1gen (2_44_1_1)	2hmb-d1hms (2_41_1_2)	1vhh-d1vhh (4_34_1_2)	1eft-d1eft_3 (3_25_1_3)	1bll-d1lam_2 (3_52_3_3)	1dcta-d1dcta (3_47_1_4)	1tys-d1tys (4_63_1_1)	1wsyb-d1wsyb (3_59_1_1)	1rcf-d1rcf (3_13_4_1)
962	4kbpb-d4kbpa1	2_1_9_1	112	1	0	0	0	0	0	1	1kcw-d1kcw_5 (2_5_1_3)	1ciy-d1ciy_2 (2_53_2_1)	2phl-d2phla1 (2_58_1_1)	1tnra-d1tnra (2_17_1_1)	1ten-d1ten (2_1_2_1)	2hmb-d1hms (2_41_1_2)	2gpb-d1gpb (3_68_1_2)	1ospo-d1ospo (2_52_1_1)	1sacb-d1saca (2_19_1_4)
963	4kmb2-d1rtm11	1_97_1_1	32	1	0	0	0	0	1	1	1fose-d1fose (1_97_2_1)	1ecma-d1ecma (1_93_1_1)	1fosf-d1fosf (1_97_2_1)	1div-d1div (4_82_1_1)	1art-d1art (3_48_1_1)	1bct-d1bct (8_30_1_1)	1rcd-d1rcd (1_24_1_1)	1ifi-d1ifj (1_97_3_1)	1ciy-d1ciy_3 (6_1_3_1)
964	4mhta-d1hmy	3_47_1_4	327	2	1	1	1	1	0	0	1dcta-d1dcta (3_47_1_4)	1xva-d1xvaa (3_47_1_2)	1gtma-d1gtma1 (3_19_1_7)	1leha-d1leha1 (3_19_1_7)	1rcf-d1rcf (3_13_4_1)	1vid-d1vid (3_47_1_1)	1bya-d1byb (3_1_1_2)	1cyda-d1cyda (3_19_1_2)	1cowe-d1bmfd3 (3_25_1_6)
965	4sgbi-d4sgbi	7_12_1_2	51	1	0	0	0	0	0	0	1prch-d1prch1 (2_27_1_1)	1abrb-d1abrb2 (2_28_2_1)	1ospo-d1ospo (2_52_1_1)	1cem-d1cem (1_73_1_2)	1bll-d1lam_2 (3_52_3_3)	1kxa-d2snv (2_31_1_3)	1vmoa-d1vmoa (2_53_1_1)	1ggt-d1ggta4 (4_3_1_2)	4fua-d1fua (3_55_1_1)
966	4tgf-d2tgf	7_3_9_1	50	7	1	1	0	0	0	0	1eph-d1egf (7_3_9_1)	1edt-d1edt (3_1_1_5)	1mbb-d1mbb_1 (4_84_1_2)	1hcz-d1ctm_2 (2_59_2_2)	2ebn-d2ebn (3_1_1_5)	1clc-d1clc_2 (2_1_1_5)	1prs-d1prr_2 (2_9_1_2)	1dlc-d1dlc_2 (2_53_2_1)	1gof-d1gof_3 (2_46_1_1)
967	4xis-d2xis	3_1_12_1	385	1	0	0	0	0	1	1	1nal1-d1nal1 (3_1_3_1)	1fbaa-d1fbaa (3_1_3_1)	2ada-d1add (3_1_2_1)	1nai-d1xel (3_19_1_2)	1xyza-d1xyza (3_1_1_3)	1tkaa-d1trka1 (3_24_1_2)	2dri-d2dri (3_72_1_1)	1onea-d1ebha1 (3_1_6_1)	1dhr-d1dhr (3_19_1_2)
968	5cyt-d5cytr	1_3_1_1	102	7	0	0	0	1	0	0	1emd-d2cmd_2 (4_92_1_1)	1fcdc-d1fcdc2 (1_3_1_3)	1dik-d1dik_3 (4_83_1_4)	1itea-d2int (1_25_1_2)	1nfp-d1nfp (3_1_13_2)	1gcb-d1gcb (4_3_1_1)	1pgo-d2pgd_1 (1_71_1_1)	1occa-d1occa1 (6_5_1_1)	1art-d1art (3_48_1_1)
969	5sgae-d1sgc	2_31_1_1	181	3	1	1	0	1	0	0	1p03a-d2alp (2_31_1_1)	1arc-d1arb (2_31_1_1)	1hgea-d1hgea (2_14_1_2)	1svb-d1svb_2 (6_10_1_1)	1tnj-d1bty (2_31_1_2)	4aahc-d4aaha (2_47_1_1)	1bvp1-d1bvp12 (2_14_1_1)	1dar-d1dar_1 (2_29_3_1)	2sil-d2sil (2_45_1_1)
970	5znf-d5znf	7_28_1_1	30	10	0	0	0	0	0	0	7icd-d7icd (3_57_1_1)	1faga-d2bmha (1_75_1_1)	1cxh-d1cdg_4 (3_1_1_1)	2ada-d1add (3_1_2_1)	1ytba-d1ytba2 (4_74_1_1)	2cpo-d1cpo_1 (1_34_2_1)	1sxl-d1sxl (4_33_7_1)	1hxp-d1hxpa2 (4_10_1_2)	1cbg-d1cbg (3_1_1_4)
971	6ldh-d1ldm_1	3_19_1_5	159	4	1	1	1	1	0	0	1llda-d1llda1 (3_19_1_5)	1emd-d2cmd_1 (3_19_1_5)	1hlpa-d1hlpa1 (3_19_1_5)	1dhr-d1dhr (3_19_1_2)	1hdca-d1hdca (3_19_1_2)	1ahia-d1fmca (3_19_1_2)	1tlfa-d1tlfa (3_72_1_1)	2bnh-d1dfji (3_7_1_1)	1cyda-d1cyda (3_19_1_2)
972	6ldh-d1ldm_2	4_92_1_1	169	5	1	1	0	0	0	0	1hlpa-d1hlpa2 (4_92_1_1)	1llda-d1llda2 (4_92_1_1)	1bdmb-d1bdma2 (4_92_1_1)	1emd-d2cmd_2 (4_92_1_1)	2tmda-d2tmda1 (3_1_7_1)	1scha-d1scha (1_65_1_1)	1pda-d1pda_2 (4_28_2_1)	7icd-d7icd (3_57_1_1)	1lyla-d1lyla2 (4_59_1_1)
973	6lyt-d193l	4_2_1_2	129	1	0	0	1	1	0	0	1sly-d1sly_2 (4_2_1_5)	1gbs-d1gbs (4_2_1_4)	1asza-d1asya2 (4_59_1_1)	1esd-d1esc (3_13_8_1)	1gln-d1gln_1 (1_67_1_1)	1jeva-d2olba (3_73_1_1)	1pii-d1pii_2 (3_1_8_1)	1occd-d1occd1 (6_5_1_1)	1nfp-d1nfp (3_1_13_2)
974	7cat-d7cata	5_6_1_1	498	1	0	0	0	0	0	0	1tyc-d2ts1_1 (1_54_1_1)	2npx-d1nhp_3 (4_46_1_1)	2myd-d1mbd (1_1_1_1)	1ctt-d1ctt_1 (3_75_1_1)	1ciy-d1ciy_3 (6_1_3_1)	1prhb-d1prha1 (1_65_1_2)	1occa-d1occa1 (6_5_1_1)	2sas-d2sas (1_34_1_5)	2gpb-d1gpb (3_68_1_2)
975	7icd-d7icd	3_57_1_1	414	2	1	1	0	0	0	0	1idm-d1idm (3_57_1_1)	1nal1-d1nal1 (3_1_3_1)	1lucb-d1lucb (3_1_13_1)	1pnr-d1pnra2 (3_72_1_1)	2lbp-d2lbp (3_72_1_1)	1dhpa-d1dhpa (3_1_3_1)	2dri-d2dri (3_72_1_1)	1noy-d1noya (3_41_3_5)	1tlfa-d1tlfa (3_72_1_1)
976	7rxn-d8rxna	7_32_4_1	52	2	0	0	0	0	0	0	1gof-d1gof_3 (2_46_1_1)	1ggt-d1ggta2 (2_1_4_1)	1eur-d1eur (2_45_1_1)	2bbkh-d2bbkh (2_46_2_1)	1igd-d1igd (4_12_1_1)	2hnp-d2hnp (3_32_1_2)	1ctt-d1ctt_2 (3_75_1_1)	1asu-d1asu (3_41_3_2)	1ryt-d1ryt_2 (7_32_4_1)
